# Ser/Thr/Tyr Protein Phosphorylation in the Archaeon *Halobacterium salinarum*—A Representative of the Third Domain of Life

**DOI:** 10.1371/journal.pone.0004777

**Published:** 2009-03-10

**Authors:** Michalis Aivaliotis, Boris Macek, Florian Gnad, Peter Reichelt, Matthias Mann, Dieter Oesterhelt

**Affiliations:** 1 Department of Membrane Biochemistry, Max Planck Institute of Biochemistry, Martinsried, Germany; 2 Department of Proteomics and Signal Transduction, Max Planck Institute of Biochemistry, Martinsried, Germany; University of Würzburg, Germany

## Abstract

In the quest for the origin and evolution of protein phosphorylation, the major regulatory post-translational modification in eukaryotes, the members of archaea, the “third domain of life”, play a protagonistic role. A plethora of studies have demonstrated that archaeal proteins are subject to post-translational modification by covalent phosphorylation, but little is known concerning the identities of the proteins affected, the impact on their functionality, the physiological roles of archaeal protein phosphorylation/dephosphorylation, and the protein kinases/phosphatases involved. These limited studies led to the initial hypothesis that archaea, similarly to other prokaryotes, use mainly histidine/aspartate phosphorylation, in their two-component systems representing a paradigm of prokaryotic signal transduction, while eukaryotes mostly use Ser/Thr/Tyr phosphorylation for creating highly sophisticated regulatory networks. In antithesis to the above hypothesis, several studies showed that Ser/Thr/Tyr phosphorylation is also common in the bacterial cell, and here we present the first genome-wide phosphoproteomic analysis of the model organism of archaea, *Halobacterium salinarum*, proving the existence/conservation of Ser/Thr/Tyr phosphorylation in the “third domain” of life, allowing a better understanding of the origin and evolution of the so-called “Nature's premier” mechanism for regulating the functional properties of proteins.

## Introduction

The reversible protein phosphorylation on serine, threonine, and tyrosine (Ser/Thr/Tyr) is a key post-translational modification in eukaryotes with stunning regulatory and signalling potential [1]. The importance of Ser/Thr/Tyr kinases and phosphatases for cell physiology has been widely documented in eukaryotes ranging from yeast to human [2], [3], where 2–3% of the open reading frames in their genomes code for known or potential protein kinases and protein phosphatases [4]–[6]. On the contrary, protein phosphorylation in prokaryotes (archaea and bacteria) is less intensively studied, thus specific information is lacking about its extent and function in these domains of life. Initial studies had shown that procaryotes use histidine/aspartate phosphorylation, mainly in their two-component systems, which represent a paradigm of prokaryotic signal transduction [7], [8]. Therefore, the hypothesis that eukaryotes mostly use Ser/Thr/Tyr phosphorylation, while prokaryotes use primarly histidine/aspartate phosphorylation, was set forth. However, in the last two decades more evidence has emerged for the prominent role of Ser/Thr/Tyr phosphorylation in prokaryotes [9]–[11].

Preliminary studies showed that bacteria possess kinases and phosphatases that structurally resemble their eukaryotic counterparts but have also developed “bacterial” kinases and phosphatases with very different domain organizations from the known homologues in eukaryotes [12]–[18]. Besides, “looking” into several archaeal genomes has revealed the widespread presence of several “eukaryotic” and “bacterial” protein kinases and phosphatases, suggesting that this versatile molecular regulatory mechanism emerged at an early point in development of “life as we know it” [13], [17], [18]. The first evidence for Ser/Thr/Tyr protein phosphorylation in a member of the “third domain of life” was reported in the extreme halophilic archaeon *H. salinarum* (then *H. halobium*) in 1980, using ^32^P radiolabeling [19]. In 1984 and 1989, with two subsequent studies, Skorko established the existence of protein phosphorylation in a second member of the archaea, the extreme acidothermophile *Sulfolobus acidocaldarius*, when a polypeptide of approximate size 40 kDa became phosphorylated on threonine when an isolated ribosomal fraction from *S. acidocaldarius* was incubated with ^32^P polyphosphate [20], [21]. Utilizing two-dimensional electrophoresis, Osorio and Jerez observed more than 20 ^32^P-labelled proteins in cells grown in the presence of ^32^P phosphate [22]. In 1997, the range of archaeons in which protein phosphorylation had been detected was extended further to include the extreme acidothermophile (*Sulfolobus solfataricus*), the extreme halophile (*Haloferax volcanii*) and the anaerobic methanogen (*Methanosarcina thermophila* TM-1). These studies employed phosphoamino acid-directed antibodies to provide the first direct evidence for the presence of phosphotyrosine in archaeal proteins. Jeon et al. extracted three tyrosine-phosphorylated polypeptides from a lysate of the hyperthermophile *T. kodakaraensis* using a substrate-trapping mutant of a potential protein tyrosine phosphatase (PTP), Tk-PTP [23], [24].

Although the above studies provide strong evidence that proteins within a broad spectrum of archaeons can be phosphorylated, little progress has been made in ascertaining precisely which archaeal proteins are phosphorylated, which kinases/phosphatases are involved, and what cellular processes are targeted by this covalent modification process. The first archaeal phosphoproteins of any type to be identified were CheY and CheA homologs in *H. salinarum* [25], [26], and the methyltransferase activation protein (MAP), from the methanogenic archaeon *Methanosarcina barkeri* [27], [28]. Unfortunately, since then, only a few archaeal phosphoproteins have been identified to date [29], [30] (Table 1). In particular, looking through the present bibliography, only 17 archaeal proteins are reported to be phosphorylated (Table 1) without any suggestion about the cellular impact of this phosphorylation, except of the case of a putative phosphohexomutase (sso0207) from *S. solfataricus* P2, in which the *in vivo* phosphorylation of Ser309 seems to regulate its catalytic activity [31]. In addition, in phosphorylation site database (http://vigen.biochem.vt.edu/xpd/xpdindex.htm), [32] 7 records were found for protein phosphorylation in archaea, including the previously mentioned putative phosphohexomutase (sso0207) from *S. solfataricus* P2; Beta-1 subunit of 20S proteasome, psmB1, from *H. volcanii* [29]; cell division control protein 6, homologs 1 and 2, mthCdc6-1 and mthCdc6-2, respectively, from *Methanothermobacter thermoautotrophicus* [33]; protein serine kinase, Rio1, from *Archaeoglobus fulgidus* [34]; protein serine kinase, SsoPK2, and protein serine/threonine kinase, SsoPK3, from *S. solfataricus* P2 [35].

**Table 1:** List of the archaeal proteins reported to be phosphorylated up to date.

No	Protein	Organism	Residue	Evidence for phosphorylation	Reference
**1**	CheA	*H. salinarum*	His	^32^P incorporation	Rudolph, J., Oesterhelt.D., 1995
**2**	CheY	*H. salinarum*	Asp	^32^P incorporation	Rudolph, J., et al., 1995
**3**	Methyltransferase-activating protein	*M. barkeri*	Ser,Thr or Tyr	^32^P incorporation	Daas, P. J. H., et al., 1996
**4**	Cdc6	*M. thermoautotrophicum*	Ser	^32^P incorporation	Grabowski, B., Kelman, Z., 2001
**5**	Cdc6	*P. aerophilum*	Ser	^32^P incorporation	Grabowski, B., Kelman, Z., 2001
**6**	Cdc6	*S. solfataricus*	Ser	^32^P incorporation	De Felice, M., et al., 2003
**7**	Q9HH97, Glycogen synthase	*S. acidocaldarius*	Ser or Thr	^32^P incorporation	Cardona, S., et al., 2001
**8**	aIF2	*P. horikoshii*	Ser48	^32^P incorporation (in vitro)	Tahara, M., et al., 2004
**9**	Phenylalanyl-tRNA synthetase β-chain	*T. kodakaraensis KOD1*	Tyr	Anti-phosphotyrosine antibodies	Jeon, S.-J., et al., 2002
**10**	Phosphomannomutase, pmm	*T. kodakaraensis KOD1*	Tyr	Anti-phosphotyrosine antibodies	Jeon, S.-J., et al., 2002
**11**	RtcB, RNA 3′-terminal-phosphate cyclase	*T. kodakaraensis KOD1*	Tyr	Anti-phosphotyrosine antibodies	Jeon, S.-J., et al., 2002
**12**	APE2, Leucyl aminopeptidase	*S. solfataricus*	Ser or Thr	^32^P incorporation	Condo, I., et al., 1998
**13**	SsoPK2, putative protein serine kinase	*S. solfataricus*	Ser	^32^P incorporation	Lower, B. H., Kennelly, P. J., 2003
**14**	SsoPK3, putative protein serine kinase	*S. solfataricus*	Thr	^32^P incorporation/Mass Spectrometry	Lower, B. H., et al., 2004
**15**	sso0207, putative phosphohexomutase	*S. solfataricus* P1	Ser	^32^P incorporation/Mass Spectrometry	Solow, B. et al. 1998/Ray, W. K. et al. 2005
**16**	O28471, putative protein serine kinase	*A. fulgidus*	Ser108	^32^P incorporation/Edman sequencing	LaRonde-LeBlanc, N., et al., 2005
**17**	Beta-1 subunit of 20S proteasome	*H. volcanii*	Ser129	Mass Spectrometry	Humbard, M. A., et al., 2006

In order to fill this gap in our knowledge, we have initiated a systematic study of the identities and functional roles of the major phosphoproteins in the extreme halophilic archaeon *H. salinarum*. The first step in the exploration of protein phosphorylation/dephosphorylation in the member of archaea, which we present here, is a genome-wide, gel-free, and site-specific phosphoproteome analysis of *H. salinarum* strain R1, and its phenotypically identical deletion mutant *ΔserB* - which lacks the only predicted phosphoserine phosphatase (*serB*, OE4405R [36], www.halolex.de) - using high accuracy mass spectrometry in combination with biochemical enrichment of phosphopeptides from digested cell lysates. The total outcome was the identification of 90 unique phosphopeptides from 69 *H. salinarum* proteins and the determination of 81 phosphorylation sites. Detected phosphoproteins are involved in a wide variety of cellular processes but are enriched in metabolism and translation. This set of archaeal proteins phosphorylated on Ser/Thr/Tyr residues is the largest available to date, supporting the emerging view that protein phosphorylation is a general and fundamental regulatory process, not restricted only to eukaryotes and bacteria, and opens the way for its detailed functional and evolutionary analysis in archaea and prokaryotes in general.

## Results and Discussion

### Ser/Thr/Tyr phosphoproteome of the halophilic archaeon *H. salinarum* strain R1

**Figure 1:**
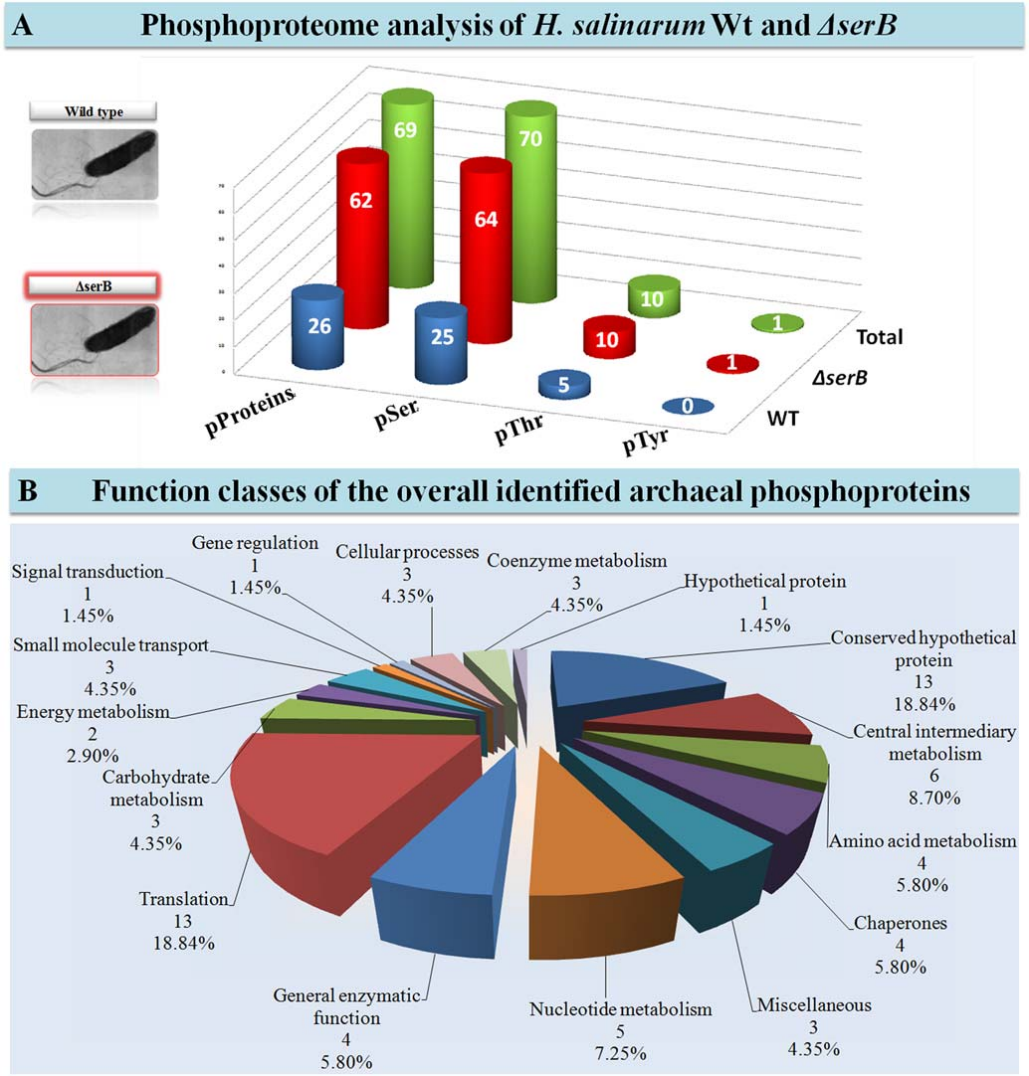
Statistical analysis of the results of the phosphoproteome analysis of *H. salinarum*. A. The number of phosphoproteins, phosphoserines, phosphothreonines and phosphotyrosines identified in Wt *H. salinarum* strain R1, *ΔserB*, and overall. B. The function classes distribution of the phosphoproteins identified in *H. salinarum*.

In the wild type (Wt), we identified 42 phosphopeptides from 26 *H. salinarum* proteins, and reliably determined 31 phosphorylation sites (Table S1). In the identified phosphopeptides, a total of 26 serines, 5 threonines and no tyrosines were phosphorylated, yielding a Ser/Thr/Tyr phosphorylation ratio of 84/16/0%, respectively (Figure 1A). The phosphoproteome analysis of *ΔserB* mutant revealed a striking increase of Ser protein phosphorylation: 100 phosphopeptides from 62 proteins were identified, and 75 phosphorylation sites reliably determined (Table S1). In the identified phosphopeptides, a total of 64 serines, 10 threonines, and one tyrosine were found to be phosphorylated, yielding a Ser/Thr/Tyr phosphorylation ratio of 86/13/1%, respectively (Figure 1A). In total, we identified 90 unique phosphopeptides from 69 *H. salinarum* proteins, and determined 81 phosphorylation sites: 70 (86%) on serine, 10 (12%) on threonine, and one (1%) on tyrosine (Table S1). It has to be noted that the sole phosphotyrosine-containing peptide, measured with 3.6 ppm mass deviation, was an outlier in the dataset and additional experiments will be needed to confirm the presence of tyrosine phosphorylation on this protein.. Detected phosphoproteins are involved in a wide variety of cellular processes but are enriched in metabolism (23, 33.3%) and translation (13, 18.8%), while many conserved hypothetical proteins (13, 18.8%), with interesting characteristics (homologies, functional domains, protein interactions etc), were found to be phosphorylated. This set of archaeal proteins phosphorylated on Ser/Thr/Tyr residues is the largest available to date.

### Verification of the role of serB as a functional phosphoserine phosphatase

Protein Ser/Thr phosphatase activity has been detected in extracts of *H. salinarum* and *H. volcanii*, but the enzymes responsible have not been identified and characterized experimentally [37], [38]. Analyzing the genome of *H. salinarum* strain R1, we detected one predicted phosphoserine phosphatase (*serB*, OE4405R) with 30% sequence identity to the human phosphoserine phosphatase (*serB*, SwissProt ID P78330). In order to determine whether this is a functional phosphoserine phosphatase, and - if so - to enhance protein phosphorylation in *H. salinarum*, we knocked-out the protein OE4405R from its genome, constructing the deletion mutant *ΔserB*, which showed an identical to the Wt phenotype during chemotaxis and phototaxis experiments (data not shown) [39]. Phosphoproteome analysis of *ΔserB* revealed a three-fold increase of Ser phosphorylation in comparison to the Wt (Figure 1A), representing the first *in vivo* experimental characterisation of a phosphoserine phosphatase in the phylogenetic domain of archaea. In particular, 49 serine phosphorylation sites were reliably and uniquely identified in *ΔserB* which is a strong indication that their dephosphorylation depends exclusively on serB. In addition to serines, 4 threonine phosphorylation sites were reliably and uniquely identified in *ΔserB*, which may imply the bi-functionality of serB as a Ser/Thr phosphatase.

### Function classes of the archaeal phosphoproteins

The identified archaeal phosphoproteins belong to 16 protein functional classes, representing a wide range of cellular processes (Figure 1B). The majority of them (33.3%) are involved in a variety of metabolic pathways such as central intermediary metabolism (8.7%), nucleotide metabolism (7.3%), amino acid metabolism (5.8%), carbohydrate metabolism (4.4%), coenzyme metabolism (4.4%), and energy metabolism (2.9%). Many identified phosphoproteins are involved in translation (18.8%), such as ribosomal proteins and translation initiation factors, whereas a significant fraction of the identified phosphoproteins have unknown cellular role (30.4%).

#### Metabolism

Key enzymes of the archaeal metabolism such as pyruvate kinase, pyruvate water dikinase, pyruvate-ferredoxin oxidoreductase, succinate dehydrogenase subunit B, isocitrate dehydrogenase, ATP synthase, subunits E and H, nucleoside-diphosphate kinase, were found to be phosphorylated, implying their possible functional regulation via phosphorylation. Pyruvate water dikinase (OE1500R), a key enzyme in gluconeogenesis, catalyzing the transfer of a phosphate group from ATP to pyruvate yielding phosphoenolpyruvate via the intermediate phosphorylation of His394, is found to be phosphorylated on Thr392, Ser393 and Ser399, located in its active centre. Isocitrate dehydrogenase is a citric acid cycle catalyst and it was shown that its human protein homolog idh1 can be phosphorylated [40]. In *E. coli*, the enzyme is completely inactivated by the phosphorylation of Ser113 (corresponds to Ser111 of archaeal icd), by action of isocitrate dehydrogenase kinase/phosphorylase [41]. In the present study, the only isocitrate dehydrogenase (OE3634F) predicted in *H. salinarum* was found to be phosphorylated on the Ser2 residue (conserved among all domains of life) at the protein N-terminus, where the initial methionine was cleaved off. Considering that there is not a predicted isocitrate dehydrogenase kinase/phosphorylase in *H. salinarum*, and that the phosphorylation occurred when serB was knocked-out, we speculate that the dephosphorylation of icd in *H. salinarum* depends on serB. Nucleoside-diphosphate kinase (OE2667F) is an important enzyme in nucleic synthesis, lipid synthesis, polysaccharide synthesis, protein elongation and signal transduction. We previously solved the 3D structure of this protein [42] and in the present study we detected phosphorylation on the Ser121 residue. This residue is conserved in all domains of life and located close to the His119, involved in the catalytic mechanism of the protein. Interestingly, Ser121 corresponds to Ser120 of the human ndkA (51% sequence identity), which is mutated to Gly120 in neuroblastoma [43]. As in the other phosphoproteomics studies of prokaryotes, the phosphoserine intermediates of four phosphomutases, which perform substrate phosphorylation via a phosphoserine intermediate [44], were identified. The phosphoserine intermediate (Ser59) of the active site of phosphoglycerate mutase (OE3653R), conserved in archaea and bacteria, which participates in the Embden-Meyerhof pathway and in gluconeogenesis, was identified. The phosphoserine intermediate of three *H. salinarum's* phosphohexomutases, OE2318R, OE4094F, and OE4190F were identified, which are conserved in all domains of life. Interestingly, the threonine residue located at −2 position from the intermediate phosphoserine, is also phosphorylated in two of the three phosphohexomutases (OE4094F and OE4190F).

#### Transcrption and Translation

Among 14 phosphorylated proteins involved in transcription and translation in *H. salinarum*, only one archaeal transcription regulator, sirR (OE1797R), which is conserved in archaea, was found to be phosphorylated on four possible residues: Thr26, Ser27, Thr28 or Tyr32. Of the 13 phosphorylated proteins involved in translation, the tryptophanyl-tRNA synthetase (OE4101R), is phosphorylated either on Ser395, 396 or 397. In the human homologue trpRS, the corresponding serines 360 and 362 are not phosphorylated, while Ser467 (not present in *H. salinarum*), known to be involved in different types of cancer [40], is phosphorylated. Two translation initiation factors and one elongation factor were found to be phosphorylated. The probable archaeal translation initiation factor SUI1 (OE4626R) is phosphorylated on Ser58, conserved in archaea and bacteria. The eukaryotic homologue of SUI1, involved in directing the ribosome to the proper start site of translation by functioning in concert with eIF-2 and the initiator tRNA-Met [45], was found to be phosphorylated on Thr15 in yeast [46], and Tyr30 in human [47] (both residues do not occur in *H. salinarum*). Interestingly, previous *in vitro* studies on the hyperthermophilic archaeon *Pyrococcus horikoshii* OT3, showed that the initiation factor aIF-2a (not found to be phosphorylated in *H. salinarum*) can be phosphorylated by a putative aIF-2a protein kinase [48] which has a 36% sequence identity to the conserved hypothetical protein OE1298R of *H. salinarum*. The alpha subunit of translation elongation factor aEF-1 (OE4721R), known to promote the GTP-dependent binding of aminoacyl-tRNA to the A-site of ribosomes during protein biosynthesis [49], is phosphorylated on Ser155 and Ser162, and possibly on Tyr152 or Tyr157. The eukaryotic translation elongation factor eEF-1 is multiply phosphorylated on several serine and threonine residues in yeast, and on tyrosine residues in humans. The phosphorylated Ser155 of *H. salinarum's* aEF-1 corresponds to the phosphorylated Ser155 of yeast's eEF-1, while the tyrosines 152 and 157, possible phosphorylated in *H. salinarum's* aEF-1, correspond to tyrosines 162 and 167 in human eEF-1 where only the Tyr162 is phosphorylated [50]. The archaeal translation elongation factor aEF-2 (OE4729R), which promotes the GTP-dependent translocation of the nascent protein chain from the A-site to the P-site of the ribosome, found to be phosphorylated on Ser383 (conserved in prokaryotes) and Ser634 (conserved in archaea). It is known that the bacterial and eukaryal EF-2 are multiply phosphorylated [10], [50], [51] on serine and threonine residues which are not conserved in archaeal EF-2. The nine phosphorylated archaeal ribosomal proteins, mainly involved in translation initiation, support translational regulation in archaea. Suggestively, the ribosomal protein S15 (OE2165R), which is mostly conserved in eukaryotes and archaea, can be phosphorylated either on Ser11, 12, 14 or Thr18 (the exact phosphosite could not be determined). Serines 12 and 14 are conserved in human rps13 (36% sequence identity to archaeal rps15), while Thr18 is replaced by Tyr18 in human rps13. The human rps13 is phosphorylated on Ser30 (not conserved in OE2165R), and on three tyrosine residues, 128, 129 (correspond to tyrosines 132 and 133, respectively, in OE2165R) and 38 [51], [52]. In *E. coli*, this protein binds to 16S ribosomal RNA and functions in early steps of ribosome assembly [53]. The ribosomal protein S11 (OE2629F), which is conserved in all domains of life and plays an essential role in selecting the correct tRNA in protein biosynthesis, was found phosphorylated on Ser125 and Thr119. The latter phosphosite is conserved and phosphorylated in the human homologue rps14 (Thr140, 49% sequence identity) [50]. The ribosomal protein S12 (OE4736R), conserved in all domains of life, plays an important role in translational initiation and is located in the interface of the 30S and 50S subunits [54]. We found it to be phosphorylated on the Ser36 residue, which is conserved in all domains of life, but it was never found phosphorylated before in any other organism. The ribosomal protein L3 (OE3388F), also conserved in most organisms, is known to bind to the 23S rRNA, and may participate in the formation of the peptidyltransferase center of the ribosome [55]. We found Ser11 to be phosphorylated in a region of the protein where the amino acid motif may be recognized by many known eukaryotic protein kinases. This serine corresponds to the Ser13 of the human homologue of rpl3 (36% sequence identity) which is not phosphorylated. Instead, the human rpl3 is phosphorylated on Tyr307 which is not conserved in archaeal rlp3. The ribosomal protein L5 (OE3407F), conserved in all domains of life, is one of the proteins that binds and probably mediates the attachment of the 5S RNA into the large ribosomal subunit, where it forms part of the central protuberance. It contacts the P site tRNA, where the 5S rRNA facilitates the stabilize positioning of ribosome-bound tRNAs. In our study, we found Ser2 and Ser55 to be phosphorylated. Consistently with our previous findings, Ser2 was also acetylated [56]. The above findings suggest that these phosphorylated proteins might play a role in the regulation of transcription and translation in archaea through their phosphorylation and dephosphorylation.

#### Conserved hypothetical proteins

A significant fraction (13) of the identified archaeal phosphoproteins are classified by genome annotation [36] (www.Halolex.de) as conserved hypothetical proteins among the domain of archaea and the other phyla. The exact cellular role of these proteins in archaeal life is not known, indicating the need for additional experiments for their characterization, which will facilitate a better understanding of archaea and the evolution of life. Most of these proteins were found to be expressed, and in some cases regulated in *H. salinarum* under different growth conditions [57] (Aivaliotis and Tebbe unpublished data), underpinning a possible significant cellular role. In addition, eight of them contain protein domains with known function and/or show homology with functional characterized proteins, such as DNA/RNA binding proteins, archaeal-type phosphoenolpyruvate carboxylases, phosphoserine phosphatases etc. In particular, the conserved hypothetical protein OE3815R was found to be phosphorylated and partially acetylated on Ser2 (conserved in archaea), the residue which in our previous studies was found to be fully N^α^-acetylated [56]. This protein contains a domain found in archaea and bacteria, which is believed to bind double-stranded DNA. The same domain is found in species ranging from yeast to mice including a human protein encoded by TFAR19 which is up-regulated in the tumor cells undergoing apoptosis [58]. The protein OE3943R, uncharacterized but conserved in archaea, was found to be phosphorylated on Thr13 and Thr18 (conserved in archaea) close to its N-terminus. It contains the oligonucleotide/oligosaccharide-binding fold which is found in all three kingdoms and its common architecture presents a binding face that has adapted to bind nucleic acids including the anti-codon binding domain of lysyl, aspartyl, and asparaginyl-tRNA synthetase [59]. This domain is found in RecG helicase involved in DNA repair [60] and at the C-terminus of bacterial DNA polymerase III alpha chain. In interaction proteomics experiments on *H. salinarum* in our lab, it was found that OE3943R interacts with several Che proteins involved in the chemotactic response of *H. salinarum* (Schlesner unpublished data). These are only two examples out of the 13 conserved hypothetical phosphoproteins of *H. salinarum* which are going to be under close inspection in future experiments.

#### Signal transduction

**Figure 2:**
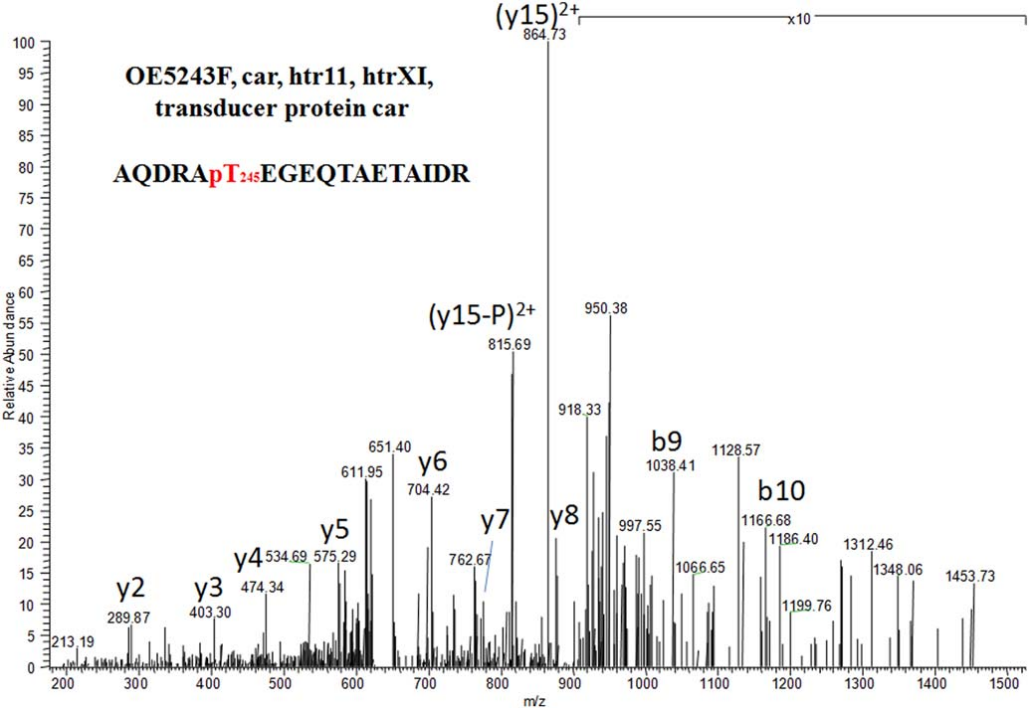
MS/MS spectrum of the phosphopeptide AQDRApTEGEQTAETAIDR of the cytoplasmic arginine transducer protein Car (OE5243F). The phosphorylation site (Thr245) is located on the methyl-accepting chemotaxis protein (MCP) signal domain of the protein. This is the first time that a MCP is reported to be phosphorylated.

In the present study, the specific cytoplasmic arginine transducer protein Car (OE5243F) was found to be phosphorylated on Thr245 (Figure 2), which is located on the methyl-accepting chemotaxis protein (MCP) signal domain of the protein, and its surrounding consensus sequence fits with the phosphorylation motif of known kinases (see Table S1). This is the first time that a MCP is reported to be phosphorylated. Car was found phosphorylated on *ΔserB* implying that the serB might be responsible for its dephosphorylation.

### Comparison of archaeal and bacterial phosphoproteomes

**Figure 3:**
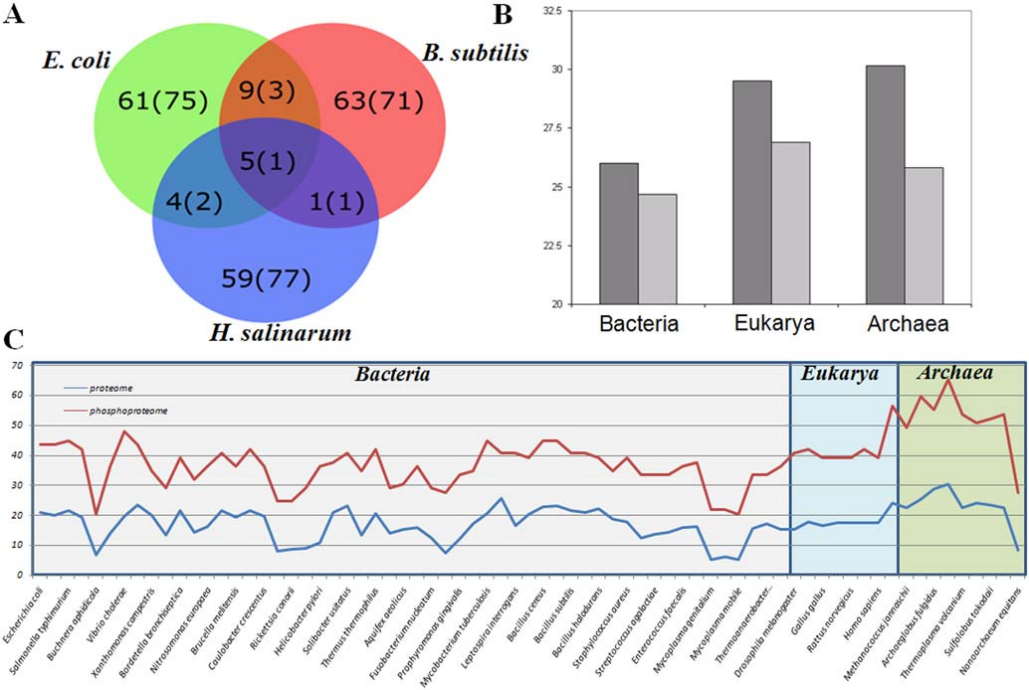
Conservation of protein phosphorylation. A. Overlap of phosphoproteins detected in *B. subtilis*, *E.coli* and *H. salinarum*. B. Average conservation of phosphorylated (dark-gray) and non-phosphorylated (light-gray) serines that occur in the loop regions. C. Conservation of the identified *H. salinarum* phosphoproteins in comparison to the whole proteome conservation among the three domains of life.

The detection of 81 phosphorylation sites on 69 archaeal proteins in this study clearly establishes the existence of Ser/Thr/Tyr phosphorylation in this domain and provides a valuable resource for further functional analysis and comparison to previously described phosphoproteomes of prokaryotes from the domain bacteria. The Wt strains of the model bacteria *B. subtilis* and *E. coli*, analyzed previously using the same analytical approach, revealed about 80 phosphoproteins in each organism [10], [11]. This is in contrast to the Wt strain of *H. salinarum* R1, where this number was about four times lower (25). However, the overall number of phosphoproteins detected in both Wt and *ΔserB* strains, approaches the number observed in bacteria, which clearly shows that this organism possesses potentially robust phosphorylation mechanisms. Analysis of orthologous phosphoproteins detected in the three prokaryotic organisms revealed only a modest overlap (Figure 3A): ten *H. salinarum* phosphoproteins were detected in either *B. subtilis* or *E. coli*, and only five phosphoproteins were detected in all three organisms (Table S2), pointing to an evolutionary conserved and potentially vital role of phosphorylation in their function. These proteins are pyruvate kinase, nucleoside-diphosphate kinase, phosphoglycerate mutase, probable phosphomannomutase, and translation elongation factor aEF-2. Although there is no available data on the essentiality of the proteins in *H. salinarum*, these conserved phosphoproteins are essential in organisms that show the corresponding orthologs. Notably, the two phosphomutases detected in all three organisms are phosphorylated on the same serine residues, which are known to have a crucial role in the active site of these enzymes. Modest overlap between phosphoproteomes of *B. subtilis*, *E. coli* and *H. salinarum* may reflect their phylogenetic distance and drastically different lifestyles, however it has to be noted that the phosphoproteomes of the model bacteria would have to be analyzed in the context of Ser/Thr phosphatase knock-outs in order to make a fair comparison. It also has to be taken into account that non-phosphorylated proteins that show homology to phosphorylated proteins of *H. salinarum* might prove to be phosphorylated on the basis of upcoming projects due to different strains, experimental designs, or applied technology.

### Evolutionary conservation of archaeal phosphoproteins/phosphorylation sites

The identified phosphoproteins and phosphorylation sites of *H. salinarum* were uploaded to the PHOSIDA [61], the phosphorylation site database (http://www.phosida.com), where they are publicly available. In addition to *H. salinarum*, PHOSIDA contains phosphorylation sites of bacteria such as *B. subtilis* [10], *E. coli* [11], *L. lactis* [62], and eukarya such as *S. cerevisiae*, *M. musculus* and *H. sapiens*, which makes it one of the most comprehensive database of phosphorylation sites. In addition to the mere presentation of phosphoproteomic data, PHOSIDA provides insights into evolutionary relationships at the protein and phosphorylation site level between detected phosphoproteins and their orthologs in more than 70 species. Pertinent to this study, proteins that have been identified to be phosphorylated in *H. salinarum* show a significantly higher conservation than non-phosphorylated proteins (Figure 3C), and phosphorylated residues are also more conserved than their non-phosphorylated counterparts (Figure 3B). This is in agreement with the observations on the evolutionary preservation of the *E. coli* phosphoproteome [11]. The higher degree of conservation both on the phosphoprotein and phosphorylation site level indicates that phosphorylation events occur on vital proteins within functionally important regions that are preserved in evolution. However, it has to be noted that there is no evidence that residues that were found to be phosphorylated in *H. salinarum* and are conserved throughout a variety of other species, are also phosphorylated in these species.

### Phosphorylation motif analysis

We tested the occurrence of significantly overrepresented consensus sequences surrounding phosphorylation sites. First we checked whether established sequence motifs, which have been proven to build up target sequences for certain eukaryotic kinases, match significantly with phosphorylation sites of *H. salinarum*. As in the previously analysed prokaryotic organisms, several eukaryotic kinase target motifs matched the sequences surrounding the phosphorylation sites, however all of them were random and therefore not statistically significant (Table S1). To check the general incidence of significantly overrepresented consensus sequences, we also tried to extract motifs on the basis of Motif-X [63]. However, this approach led to the same outcome, as it did not find evidence for any preferred sequence pattern. These results are in agreement with previous studies on the bacterial phosphoproteome, where no consensus phosphorylation sequences could be found [10], [11].

## Materials and Methods

### Strains, culture conditions and cell lysis

Cells of *H. salinarum* strain R1 and strain *ΔserB*, which lacks the predicted phosphoserine phosphatase *serB*, were grown aerobically in 1 L of complete medium, in the dark, as described before [64], to an optical density of 1.0 at 578 nm, which corresponds to the stationary phase. The strain *ΔserB* was constructed according to Koch et al. 2005 [65] (Figure S1) and the cell lysis is described in detail in Appendix S1.

### Protein digestion and phosphopeptide enrichment

About 20 mg of protein extract was dissolved in denaturation solution (6 M urea, 2 M thiourea, 1% n-octylglucoside in 20 mM ammonium bicarbonate), and prepared for phosphopeptide enrichment using a combination of strong cation exchange (SCX) chromatography and titanium oxide (TiO_2_) beads, as described previously [10], [66] (Appendix S1).

### Liquid chromatography – mass spectrometry analysis

Liquid chromatography was performed on an 1100 nano-HPLC (Agilent Technologies) coupled to the LTQ-Orbitrap mass spectrometer (Thermo Fisher Scientific), using a nano-electrospray interface (Proxeon Biosystems) as described previously [10] (Appendix S1).

### Data processing and validation

Raw MS spectra were processed using MaxQuant software v. 1.0.6.4 [67], [68] and peak lists were searched using the Mascot search engine (Matrix Science) against a concatenated forward and reversed *H. salinarum* protein database (www.halolex.de), containing 5642 entries. The search criteria which were employed are described in Appendix S1. All phosphopeptide spectra identified by Mascot were further processed and validated using the MaxQuant software. Stringent acceptance criteria were applied, which included maximum mass deviation of the precursor ion of 5 ppm, and rejection of peptides shorter than seven amino acids. Peptides identified by Mascot as phosphorylated on His or Asp, were manually checked and rejected in case no fragment ions confirming their exact location were observed. All hits to the reversed *H. salinarum* protein sequences were also rejected and the false positive rate of the reported dataset at the peptide level is estimated to be 1%. The probabilities for phosphorylation at each potential site on a peptide were calculated from the PTM scores, as described previously [51]. A BLAST analysis of all detected phosphopeptides against the complete NCBI protein database was performed to exclude the possibility of detection of low abundant phosphopeptides of eukaryotic origin present in the reagents used in sample preparation. Annotated MS/MS spectra of all identified phosphopeptides are presented in Figure S2.

### Bioinformatics analysis

In order to explore the possibility of over-representation of different protein classes among those phosphopeptides identified in our experiments, an enrichment analysis of their gene ontology (GO) terms was implemented as previously described [10]. Information on detected phosphopeptides and phosphoproteins were uploaded to the phosphorylation site database PHOSIDA (www.phosida.com), and the evolutionary analysis was performed as previously described [10], [61], [69] (Appendix S1). The derived evolutionary relationships were used to check the overall conservation of phosphorylated proteins and phosphorylated sites between *H. salinarum*, *B. subtilis* [10] and *E. coli* [11].

### Motif Analysis

The identified phosphorylation sites were screened for significant matches to known sequence motifs: We employed the χ2-test to analyze whether phosphosites in the third domain of life match significantly to established motifs of various eukaryotic kinases ranging from PKA to CKI. In addition, we used Motif-X [63] to derive candidate motifs on the basis of all phosphorylation sites and their surrounding+/−6 residues. We applied the Motif-X method to phosphorylated threonines and serines separately and used the entire *H. salinarum* database (www.halolex.de) as a background set. Due to the relatively low number of instances, we set the requiring parameters to be quite loose (minimum occurrences = 5, significance level = 0.01).

## Supporting Information

Appendix S1 Experimental part
(0.04 MB DOC)

Table S1 List of the identified phosphoproteins in H. salinarum. The H. salinarum phosphopeptides identified from Wt and ΔserB are presented with information regarding their Mascot score, identified phosphosite (if could be determined), protein and site conservation among all domains of life, and function or/and pathway in which the phosphoproteins are involved. pX: determined phosphosite; p[…]: possible phosphosite; (M): cleaved initial methionine; Ac: N-terminal acetylation.
(0.07 MB XLS)

Table S2 Phosphoproteome conservation of H.salinarium. On the basis of two-directional BLASTP alignments, we defined orthologous proteins that are phosphorylated in H.salinarium as well as in E.coli or B.subtilis. Phosphorylation sites of conserved phosphoproteins are illustrated in brackets. Conserved Phosphosites are marked in bold.
(0.02 MB XLS)

Figure S1 The primers and the plasmid pMA101. They were used for the deletion of OE4405R - serB - from the genome of H.salinarium.
(1.43 MB TIF)

Figure S2 MS/MS spectra of all identified phosphopeptides. Note that each phosphopeptide is presented with two spectra: the lower is the raw spectrum, the upper is the processed spectrum, containing only peaks submitted to database search. Assigned fragment ions are annotated in bold letters. Fragment ions arising from the neutral loss of phosphoric acid are marked with an asterisk.
(24.11 MB PDF)
