# Supplementary material for: Ser/Thr/Tyr Protein Phosphorylation in the Archaeon Halobacterium salinarum—A Representative of the Third Domain of Life
Source: PLoS One. 2009 Mar 10;4(3):e4777. doi: 10.1371/journal.pone.0004777 (PMC2652253; doi:10.1371/journal.pone.0004777)
Supplement: Appendix S1 — Experimental part (0.04 MB DOC) [file pone.0004777.s001.doc]

***Cell lysis***

Cells of *H. salinarum* were pelleted by centrifugation (10 min at 6500g, at 4°C) and resuspended in lysis buffer containing 50 mM Tris-HCl, pH 7.5, 5 mM of each of the following phosphatase inhibitors: sodium fluoride, 2-glycerol phosphate, sodium vanadate and sodium pyrophosphate, and one tablet of protease inhibitor coctail (Complete, EDTA-free, Roche). Cell lysis was performed by sonication (10x15 sec activation with 45 sec pause, Branson sonifier 450, 1/2 inch disruptor horn, level 8). DNAse I (100 μg/ml) (Sigma) and N-octylglucoside detergent (final concentration of 1%) were added to the lysate and incubated 30 min at 37° C, for more efficient extraction and solubilization of membrane proteins. Cellular debris were removed by centrifugation at 25000g for 30 min, at 4°C. The crude protein extract was extensively dialyzed against deionized water and concentrated with Amicon filters (cut-off 10 kDa).

***Data processing and validation-Mascot search criteria***

Full tryptic specificity was required; 3 missed cleavages were allowed; carbamidomethylation was set as fixed modification; oxidation (M), N-acetylation (protein), and phosphorylation (STY) were set as variable modifications; allowed maximum mass deviation in database search was 7 ppm for the precursor ion, and 0.5 Da for fragment ions. A separate search was preformed with carbamidomethylation as fixed and phosphorylation (H), (D) as variable modifications.

***Bioinformatics analysis-Evolutionary analysis***

Briefly, phylogenetic relationships between phosphorylated proteins of *H. salinarum* and proteins of 70 other species ranging from archaea to human were derived on the basis of two-directional BLASTP result [59], and the global alignments of homologous proteins were generated by the Needle software [68]. The phylogeny of each phosphorylated protein, as well as the conservation of each phosphorylated residue is illustrated at the evolutionary section of the PHOSIDA database. Furthermore, for each phosphorylated residue, its predicted structural environment along with the corresponding identified phosphopeptides is displayed.

***Protein digestion and phosphopeptide enrichment***

Briefly, the proteins were reduced and alkylated, digested in-solution for 3 hrs with endoproteinase Lys-C (1/100 w/w) (Waco), diluted four times with 20 mM ammonium bicarbonate and digested overnight with sequencing grade modified trypsin (1/100 w/w) (Promega). The first stage of phosphopeptide enrichment was performed using SCX as described previously [10]. Sixteen SCX fractions were collected, including the flow-through, and subjected to the second stage of phosphopeptide enrichment using TiO_2_ as described previously [10]. The peptides were eluted from the beads with 0.5% ammonium hydroxide solution in 40% acetonitrile (pH 10.5), dried nearly to completeness in a vacuum-centrifuge and resuspended to 10 μL with 1% TFA/2% acetonitrile in water for LC-MS/MS analysis.

***Liquid chromatography – mass spectrometry analysis***

The LTQ-Orbitrap was operated in the positive ion mode, with acquisition cycle consisting of a full scan recorded in the orbitrap analyzer at resolution R=60000, followed by collision-induced dissociation of the five most-intense peptide ions in the LTQ analyzer. Up to 500 ions were dynamically excluded for 90 s upon sequencing to improve the experimental dynamic range of peptide identification. "Multistage activation" at -97.97, -48.99 and -32.66 Thompson (Th) relative to the precursor ion was enabled in all MS/MS events to improve fragmentation spectra of phosphopeptides and the full scans were re-calibrated on-the-fly using the "lock mass" option as described previously [66].
