# Supplementary figures and images for: Ser/Thr/Tyr Protein Phosphorylation in the Archaeon Halobacterium salinarum—A Representative of the Third Domain of Life
Source: PLoS One. 2009 Mar 10;4(3):e4777. doi: 10.1371/journal.pone.0004777 (PMC2652253; doi:10.1371/journal.pone.0004777)

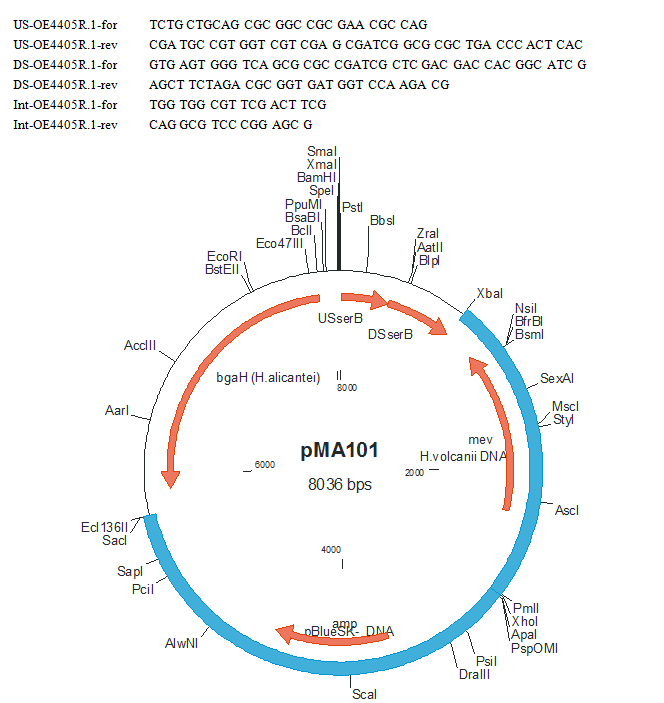

Supplement: Figure S1 — The primers and the plasmid pMA101. They were used for the deletion of OE4405R - serB - from the genome of H.salinarium. (1.43 MB TIF) [file pone.0004777.s004.tif]
