# Supplementary material for: Ser/Thr/Tyr Protein Phosphorylation in the Archaeon Halobacterium salinarum—A Representative of the Third Domain of Life
Source: PLoS One. 2009 Mar 10;4(3):e4777. doi: 10.1371/journal.pone.0004777 (PMC2652253; doi:10.1371/journal.pone.0004777)
Supplement: Figure S2 — MS/MS spectra of all identified phosphopeptides. Note that each phosphopeptide is presented with two spectra: the lower is the raw spectrum, the upper is the processed spectrum, containing only peaks submitted to database search. Assigned fragment ions are annotated in bold letters. Fragment ions arising from the neutral loss of phosphoric acid are marked with an asterisk. (24.11 MB PDF) [file pone.0004777.s005.pdf]

Supplemental Data

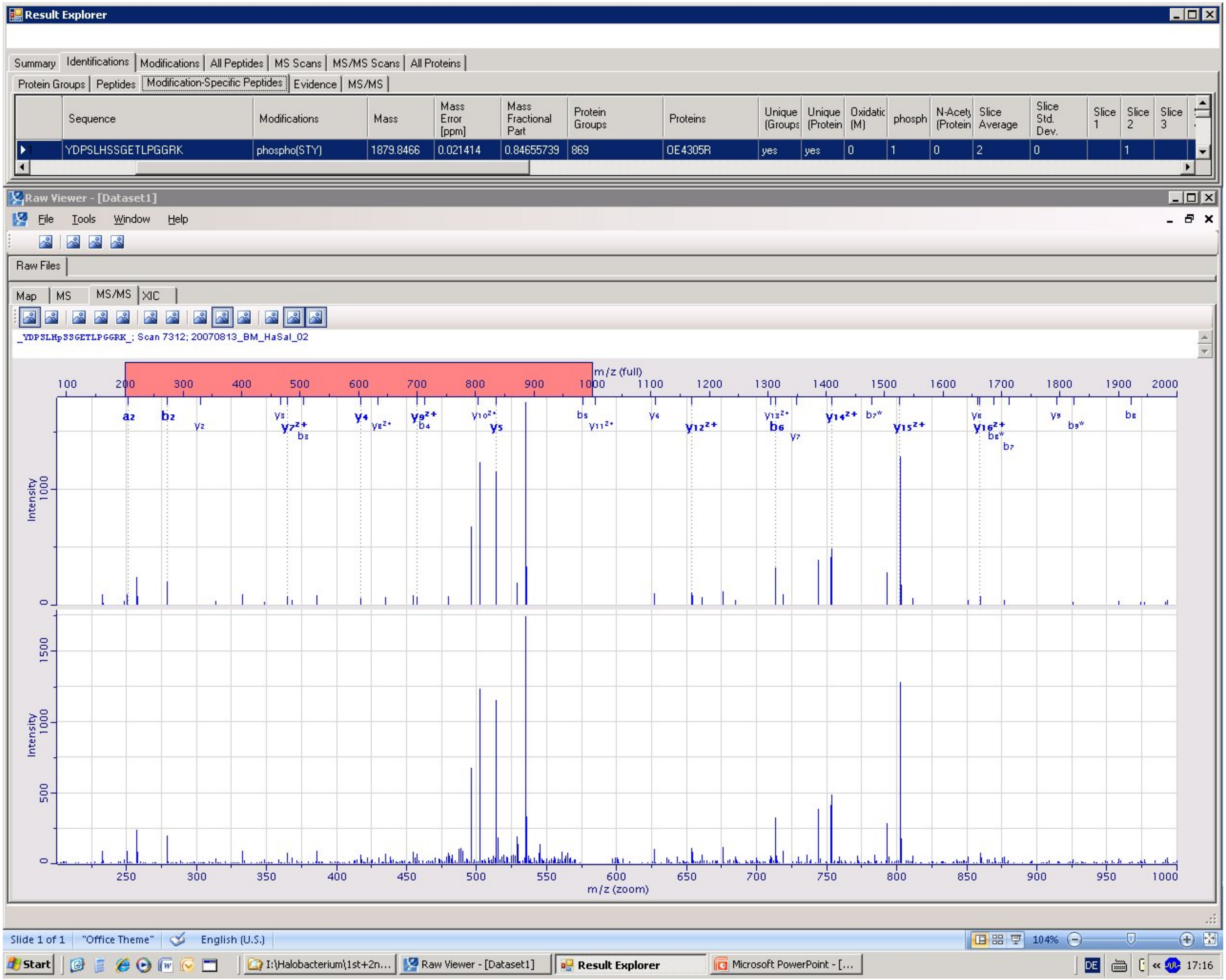

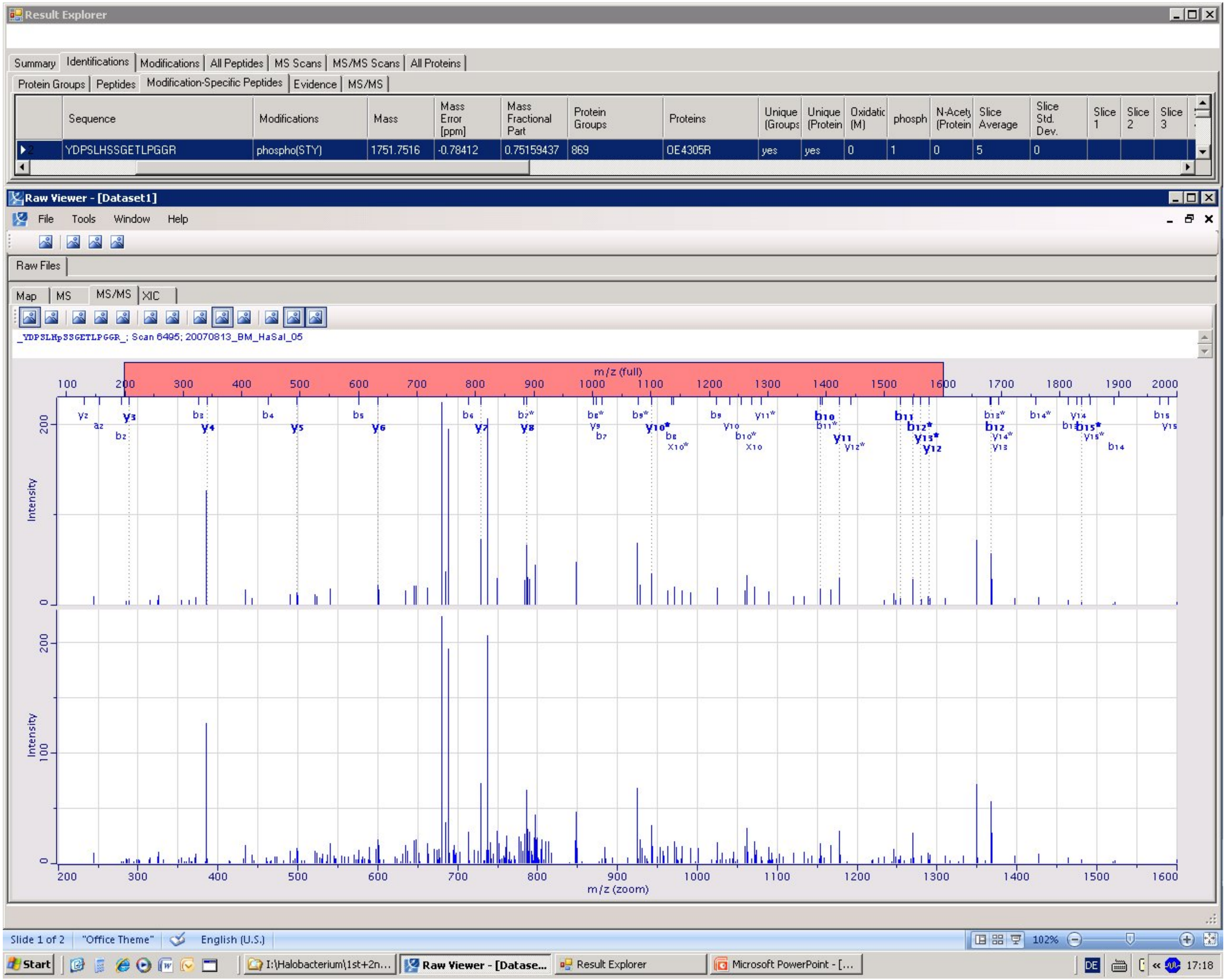
Result Explorer
Summary
Identifications
Modifications
All Peptides
MS Scans
MS/MS Scans
All Proteins
Protein Groups
Peptides
Modification-Specific Peptides
Evidence
MS/MS
Sequence
Modifications
Mass
Mass Error [ppm]
Mass Fractional Part
Protein Groups
Proteins
YDPSLHSSGETLPGGR
phospho(STY)
1751.7516
-0.78412
0.75159437
869
OE4305R
yes
Raw Viewer - [Dataset1]
File
Tools
Window
Help
Raw Files
Map
MS
MS/MS
XIC
YDPSLHpSSGETLPGGR; Scan 6495; 20070813_BM_HaSal_05
m/z (full)
Intensity
m/z (zoom)
Slide 1 of 2
"Office Theme"
English (U.S.)
Start
Raw Viewer - [Datase...
Result Explorer
Microsoft PowerPoint - [...
17:18

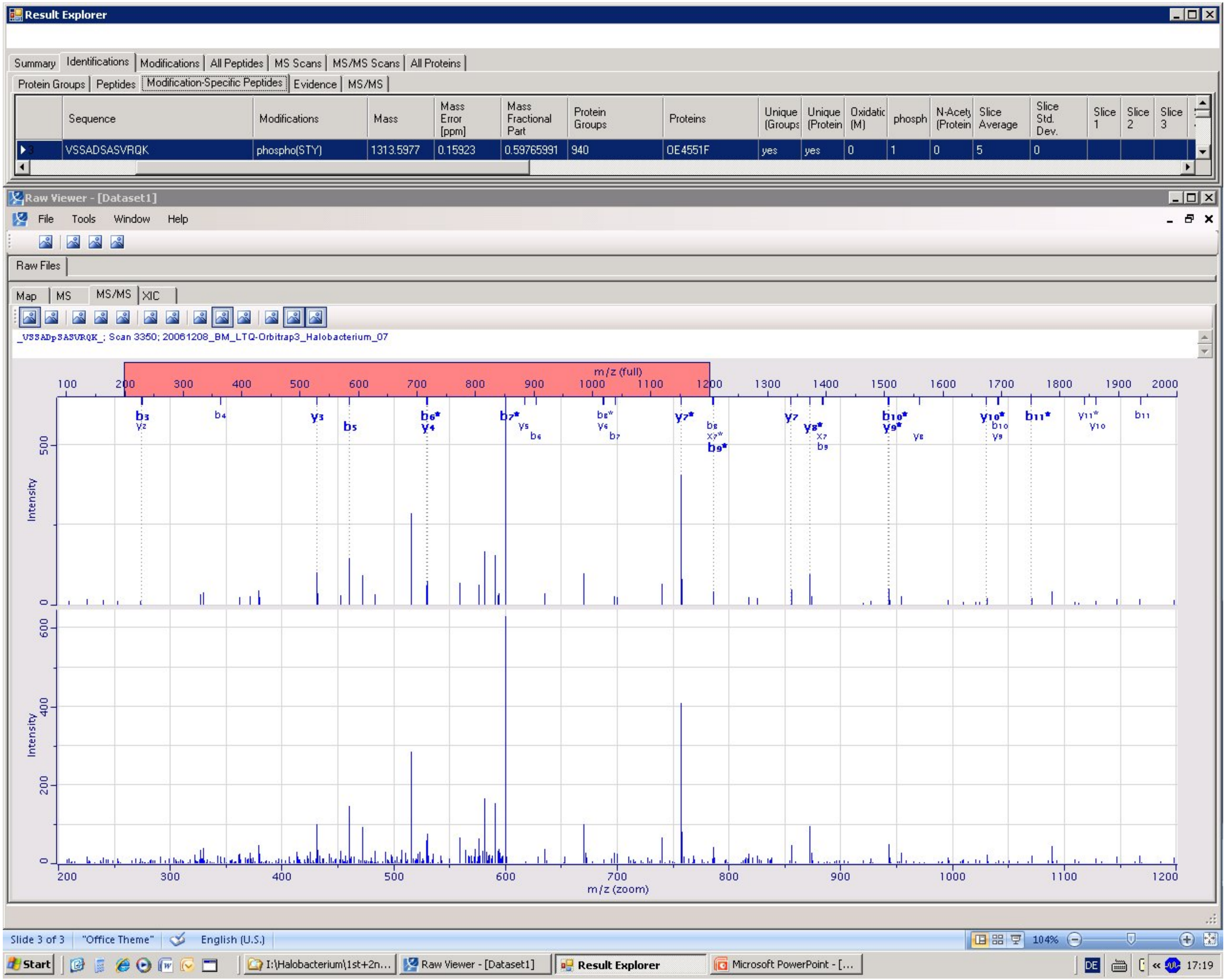
Result Explorer
Summary
Identifications
Modifications
All Peptides
MS Scans
MS/MS Scans
All Proteins
Protein Groups
Peptides
Modification-Specific Peptides
Evidence
MS/MS
Sequence | Modifications | Mass | Mass Error [ppm] | Mass Fractional Part | Protein Groups | Proteins | Unique (Groups | Unique (Protein | Oxidatic (M) | phosph | N-Acety (Protein | Slice Average | Slice Std. Dev. | Slice 1 | Slice 2 | Slice 3
VSSADSASVRQK | phospho(STY) | 1313.5977 | 0.15923 | 0.59765991 | 940 | OE4551F | yes | yes | 0 | 1 | 0 | 5 | 0
Raw Viewer - [Dataset1]
File
Tools
Window
Help
Raw Files
Map
MS
MS/MS
XIC
VSSADpSASVRQK; Scan 3350; 20061208_BM_LTQ-Orbitrap3_Halobacterium_07
m/z (full)
Intensity
m/z (zoom)
Slide 3 of 3
"Office Theme"
English (U.S.)
104%
Start
I:\Halobacterium\1st+2n...
Raw Viewer - [Dataset1]
Result Explorer
Microsoft PowerPoint - [...
17:19

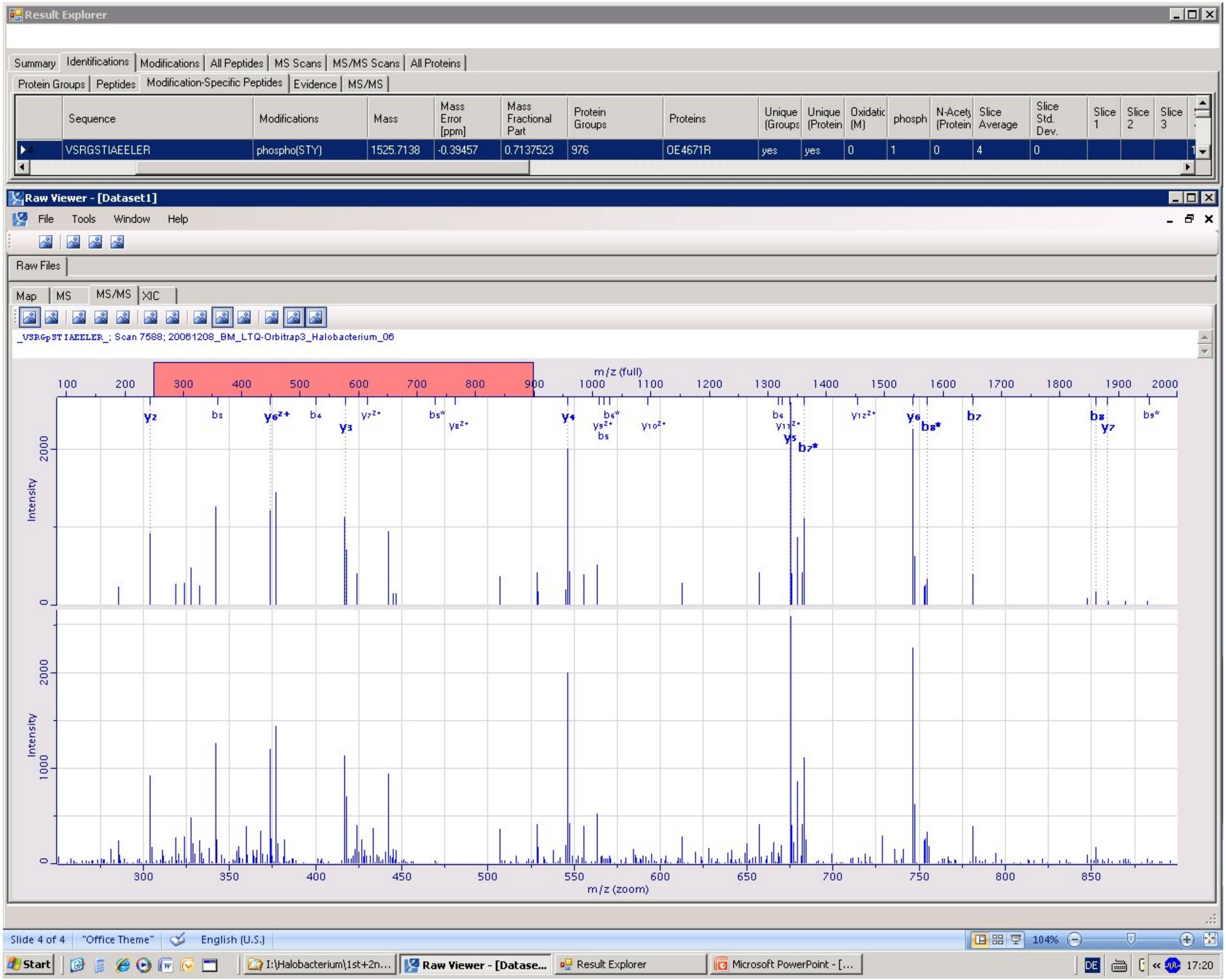
Result Explorer
Summary | Identifications | Modifications | All Peptides | MS Scans | MS/MS Scans | All Proteins
Protein Groups | Peptides | Modification-Specific Peptides | Evidence | MS/MS
Sequence | Modifications | Mass | Mass Error [ppm] | Mass Fractional Part | Protein Groups | Proteins | Unique (Groups | Unique (Protein | Oxidatic (M) | phosph | N-Acety (Protein | Slice Average | Slice Std. Dev. | Slice 1 | Slice 2 | Slice 3
VSRGSTIAEELER | phospho(STY) | 1525.7138 | -0.39457 | 0.7137523 | 976 | OE4671R | yes | yes | 0 | 1 | 0 | 4 | 0
Raw Viewer - [Dataset1]
File Tools Window Help
Raw Files
Map MS MS/MS XIC
VSRGpSTIAEELER; Scan 7588; 20061208_BM_LTQ-Orbitrap3_Halobacterium_06
m/z (full)
Intensity
m/z (zoom)
Slide 4 of 4 "Office Theme" English (U.S.)
Start
Raw Viewer - [Datase...
Result Explorer
Microsoft PowerPoint - [...
17:20

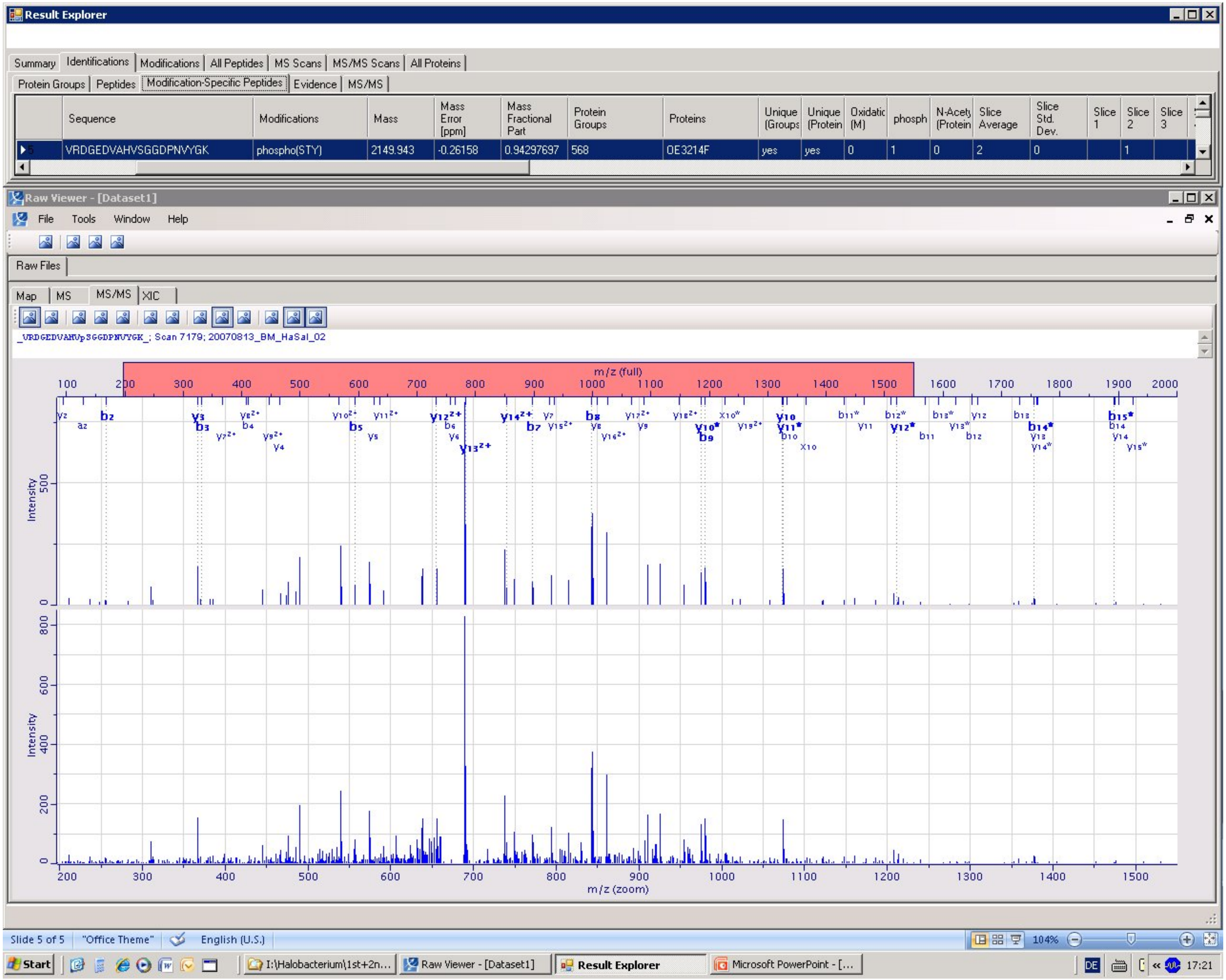
Result Explorer
Summary | Identifications | Modifications | All Peptides | MS Scans | MS/MS Scans | All Proteins
Protein Groups | Peptides | Modification-Specific Peptides | Evidence | MS/MS
Sequence | Modifications | Mass | Mass Error [ppm] | Mass Fractional Part | Protein Groups | Proteins | Unique (Groups | Unique (Protein | Oxidatic (M) | phosph | N-Acety (Protein | Slice Average | Slice Std. Dev. | Slice 1 | Slice 2 | Slice 3
VRDGEDVAHVSGGDPNVYGK | phospho(STY) | 2149.943 | -0.26158 | 0.94297697 | 568 | OE3214F | yes | yes | 0 | 1 | 0 | 2 | 0 | | 1
Raw Viewer - [Dataset1]
File Tools Window Help
Raw Files
Map MS MS/MS XIC
VRDGEDVAHVpSGGDPNVYGK; Scan 7179; 20070813_BM_HaSal_02
m/z (full)
Intensity
m/z (zoom)
Slide 5 of 5 "Office Theme" English (U.S.) 104%
Start I:\Halobacterium\1st+2n... Raw Viewer - [Dataset1] Result Explorer Microsoft PowerPoint - [... DE 17:21

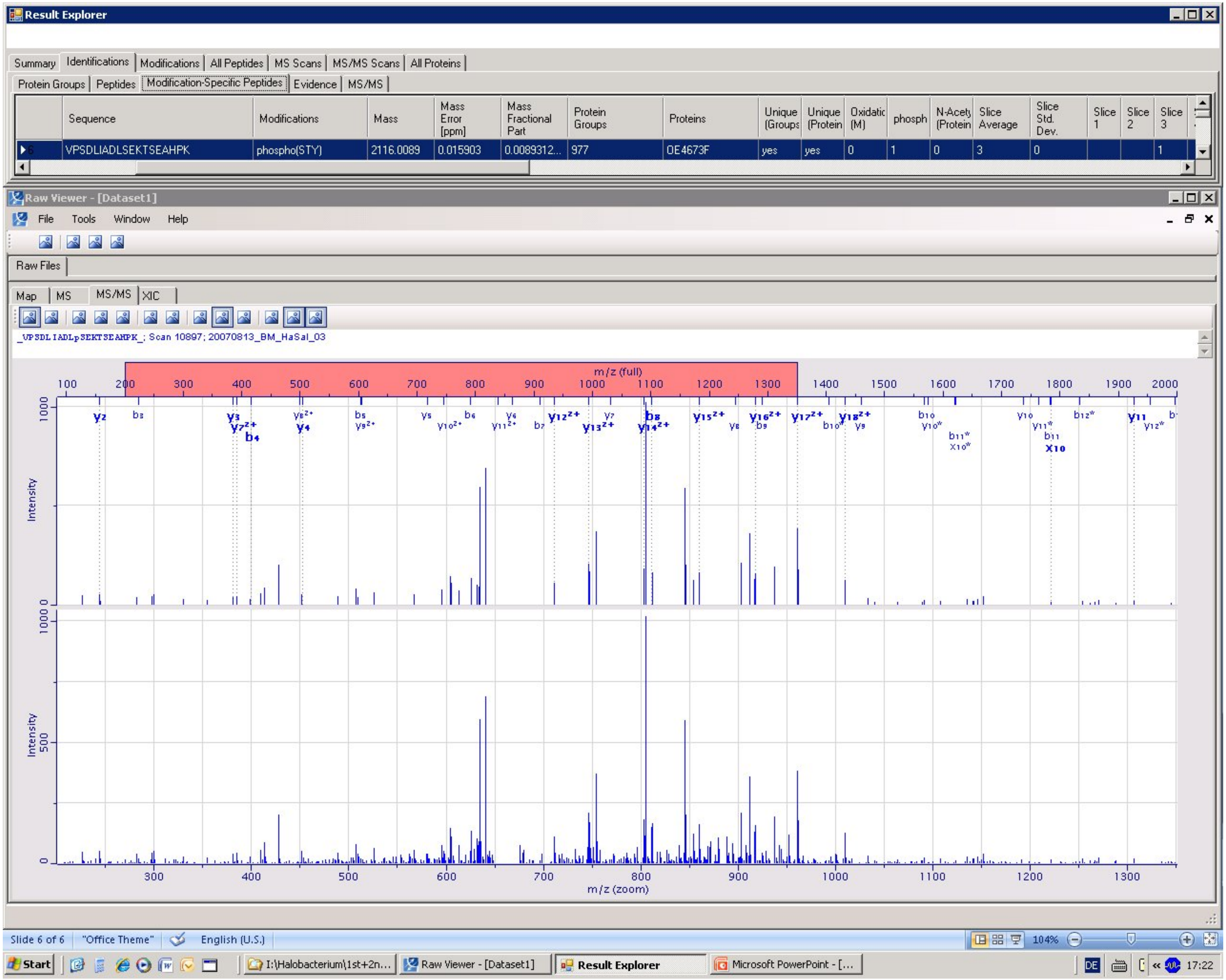
Result Explorer
Summary
Identifications
Modifications
All Peptides
MS Scans
MS/MS Scans
All Proteins
Protein Groups
Peptides
Modification-Specific Peptides
Evidence
MS/MS
Sequence
Modifications
Mass
Mass Error [ppm]
Mass Fractional Part
Protein Groups
Proteins
Slice Average
VPSDLIADLSEKTSEAHPK
phospho(STY)
2116.0089
0.015903
977
OE4673F
yes
Raw Viewer - [Dataset1]
File
Tools
Window
Help
Raw Files
Map
MS
MS/MS
XIC
VPSDLIADLpSEKTSEAHPK; Scan 10897; 20070813_BM_HaSal_03
m/z (full)
Intensity
m/z (zoom)
Slide 6 of 6
"Office Theme"
English (U.S.)
104%
Start
Raw Viewer - [Dataset1]
Result Explorer
17:22

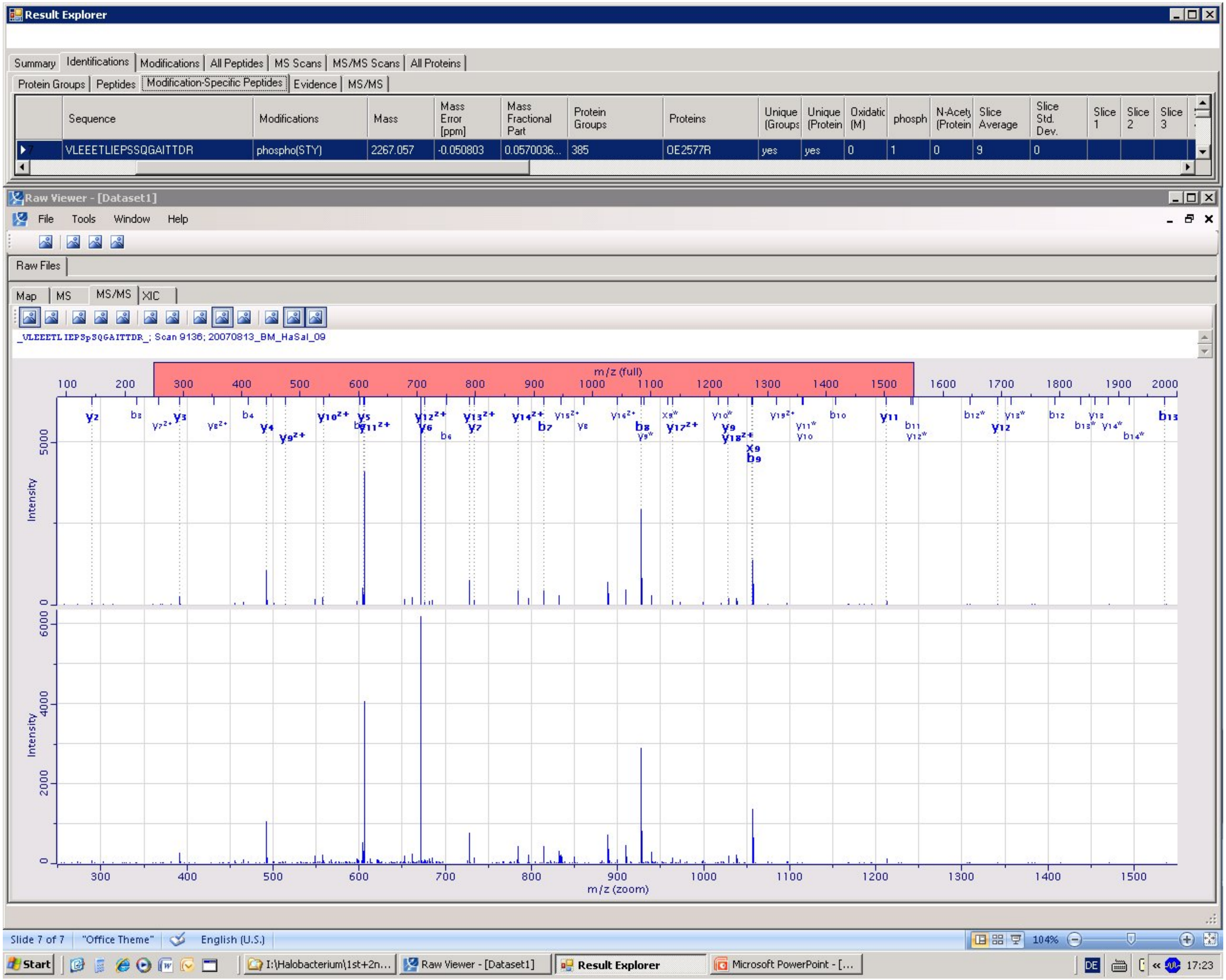
Result Explorer
Summary
Identifications
Modifications
All Peptides
MS Scans
MS/MS Scans
All Proteins
Protein Groups
Peptides
Modification-Specific Peptides
Evidence
MS/MS
Sequence
Modifications
Mass
Mass Error [ppm]
Mass Fractional Part
Protein Groups
Proteins
Slice Average
Slice Std. Dev.
VLEEETLIEPSSQGAITTDR
phospho(STY)
2267.057
-0.050803
0.0570036...
385
OE2577R
yes
yes
Raw Viewer - [Dataset1]
File
Tools
Window
Help
Raw Files
Map
MS
MS/MS
XIC
VLEEETLIEPSpSQGAITTDR; Scan 9136; 20070813_BM_HaSal_09
m/z (full)
Intensity
m/z (zoom)
Slide 7 of 7
"Office Theme"
English (U.S.)
104%
Start
Raw Viewer - [Dataset1]
Result Explorer
Microsoft PowerPoint - [...
17:23

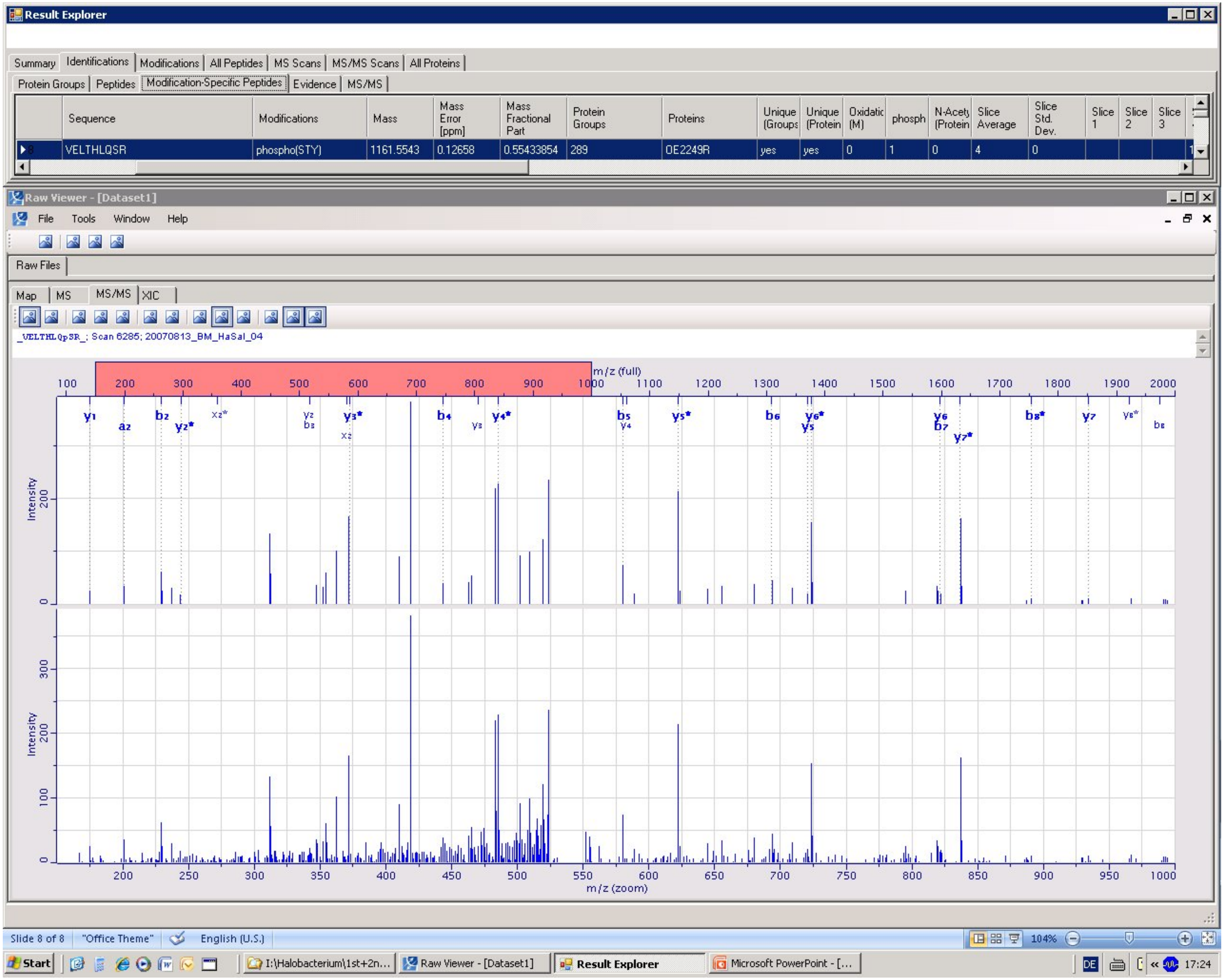
Result Explorer
Summary
Identifications
Modifications
All Peptides
MS Scans
MS/MS Scans
All Proteins
Protein Groups
Peptides
Modification-Specific Peptides
Evidence
MS/MS
Sequence
Modifications
Mass
Mass Error [ppm]
Mass Fractional Part
Protein Groups
Proteins
VELTHLQSR
phospho(STY)
1161.5543
0.12658
0.55433854
289
OE2249R
Raw Viewer - [Dataset1]
File
Tools
Window
Help
Raw Files
Map
MS
MS/MS
XIC
VELTHLQpSR; Scan 6285; 20070813_BM_HaSal_04
m/z (full)
Intensity
m/z (zoom)
Slide 8 of 8
"Office Theme"
English (U.S.)
Start
Raw Viewer - [Dataset1]
Result Explorer
Microsoft PowerPoint - [...
17:24

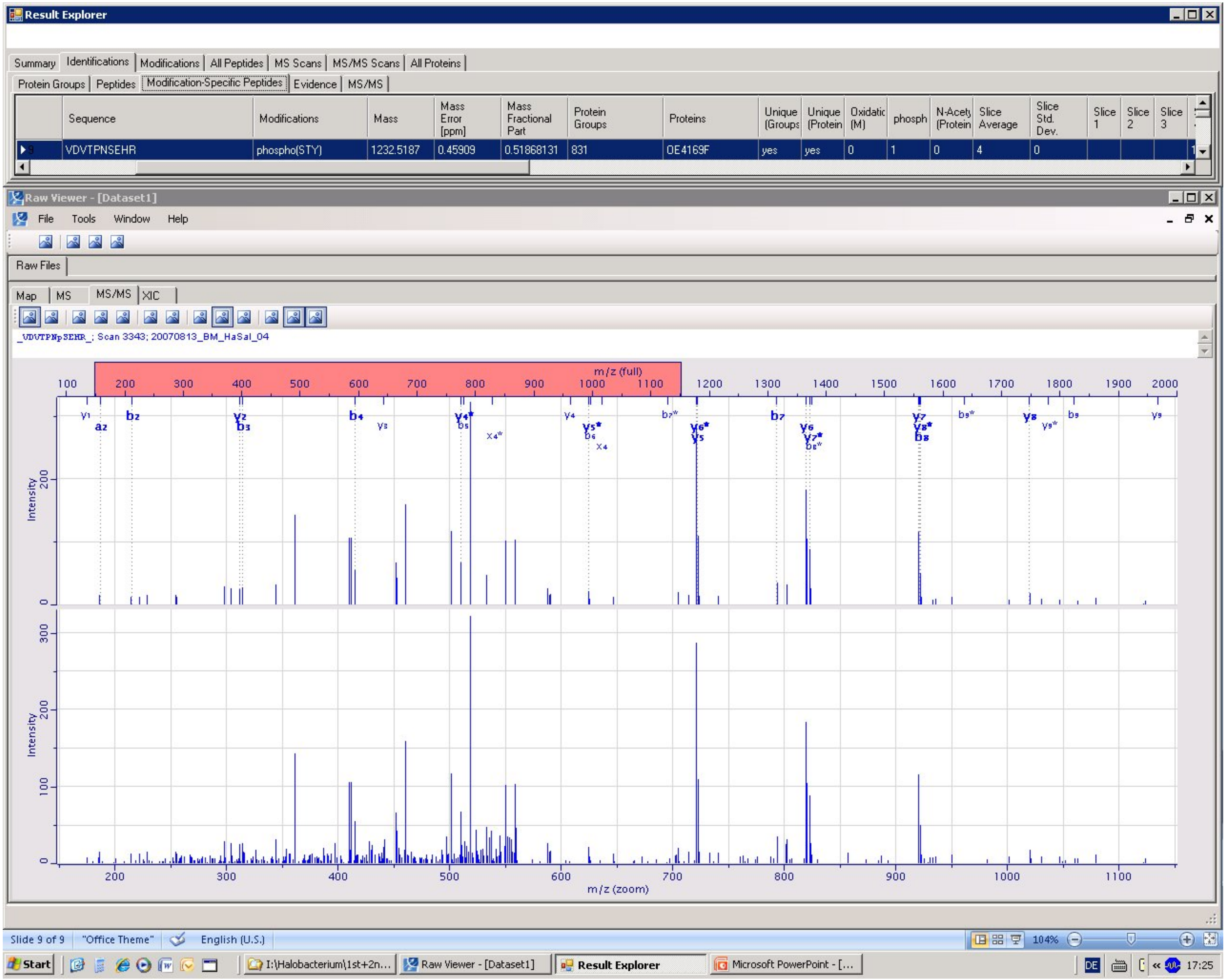
Result Explorer
Summary
Identifications
Modifications
All Peptides
MS Scans
MS/MS Scans
All Proteins
Protein Groups
Peptides
Modification-Specific Peptides
Evidence
MS/MS
Sequence
Modifications
Mass
Mass Error [ppm]
Mass Fractional Part
Protein Groups
Proteins
VDVTPNSEHR
phospho(STY)
1232.5187
0.45909
0.51868131
831
OE4169F
yes
Raw Viewer - [Dataset1]
File
Tools
Window
Help
Raw Files
Map
MS
MS/MS
XIC
VDVTPNpSEHR; Scan 3343; 20070813_BM_HaSal_04
m/z (full)
Intensity
m/z (zoom)
Slide 9 of 9
"Office Theme"
English (U.S.)
104%
Start
Raw Viewer - [Dataset1]
Result Explorer
Microsoft PowerPoint - [...
17:25

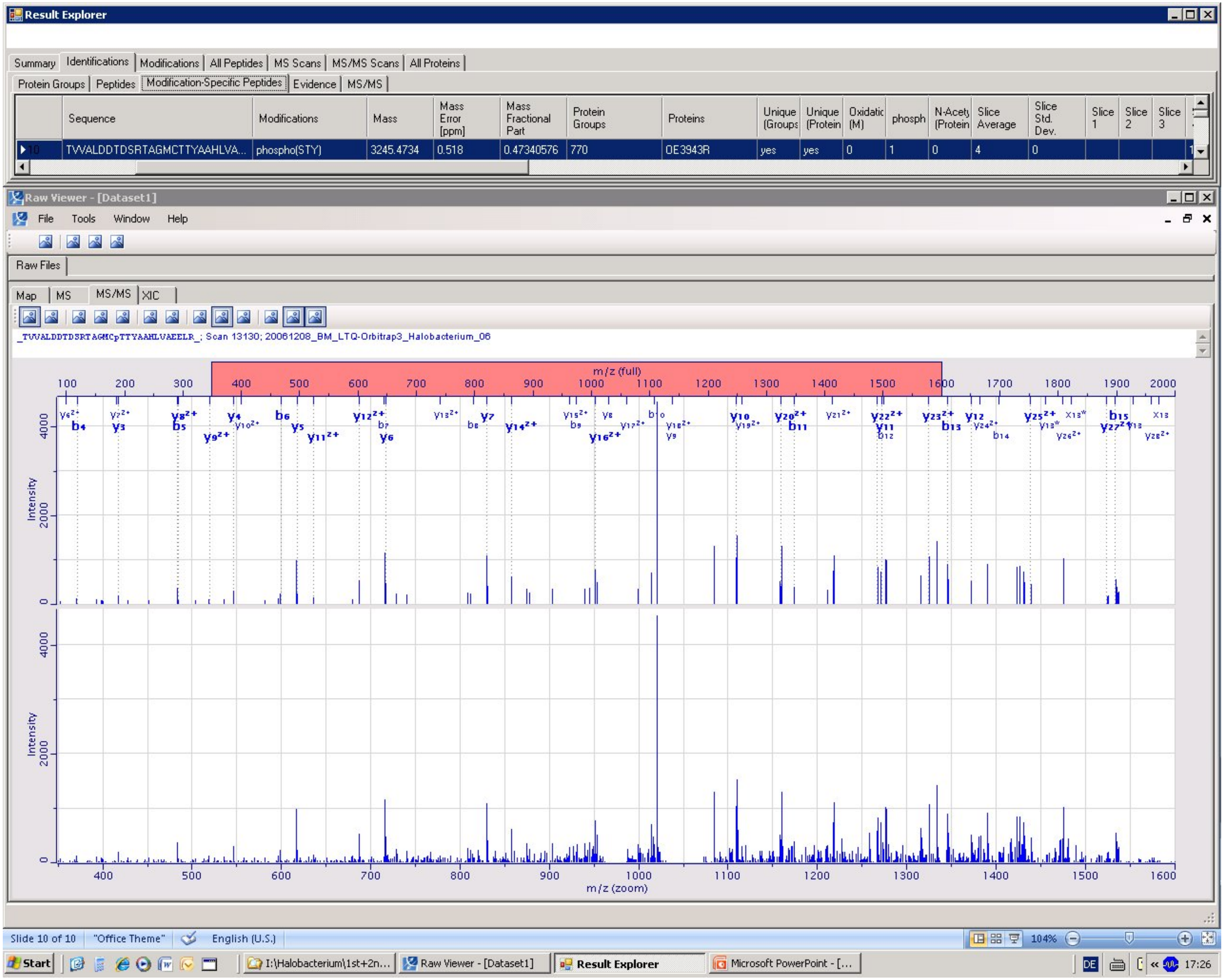
Result Explorer
Summary | Identifications | Modifications | All Peptides | MS Scans | MS/MS Scans | All Proteins
Protein Groups | Peptides | Modification-Specific Peptides | Evidence | MS/MS
Sequence | Modifications | Mass | Mass Error [ppm] | Mass Fractional Part | Protein Groups | Proteins | Unique (Groups | Unique (Protein | Oxidatio (M) | phosph | N-Acety (Protein | Slice Average | Slice Std. Dev. | Slice 1 | Slice 2 | Slice 3
TVVALDDTDSRTAGMCTTYAAHLVA... | phospho(STY) | 3245.4734 | 0.518 | 0.47340576 | 770 | OE3943R | yes | yes | 0 | 1 | 0 | 4 | 0
Raw Viewer - [Dataset1]
File Tools Window Help
Raw Files
Map MS MS/MS XIC
TVVALDDTDSRTAGMCpTTYAAHLVAEELR; Scan 13130; 20061208_BM_LTQ-Orbitrap3_Halobacterium_06
m/z (full)
Intensity
m/z (zoom)
Slide 10 of 10 "Office Theme" English (U.S.)
Start
I:\Halobacterium\1st+2n...
Raw Viewer - [Dataset1]
Result Explorer
Microsoft PowerPoint - [...
17:26

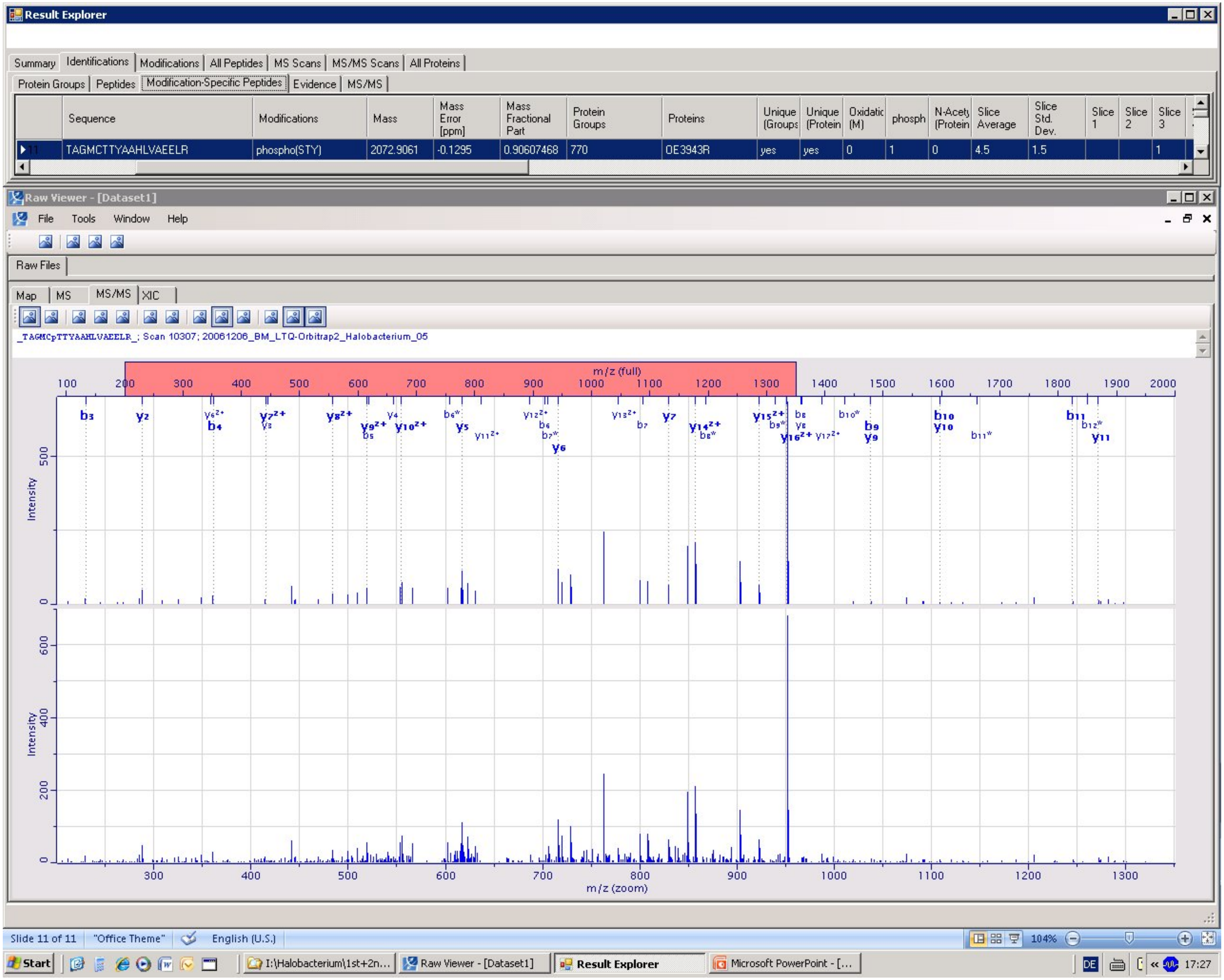
Result Explorer
Summary
Identifications
Modifications
All Peptides
MS Scans
MS/MS Scans
All Proteins
Protein Groups
Peptides
Modification-Specific Peptides
Evidence
MS/MS
Sequence
Modifications
Mass
Mass Error [ppm]
Mass Fractional Part
Protein Groups
Proteins
TAGMCTTYAAHLVAEELR
phospho(STY)
2072.9061
-0.1295
0.90607468
770
OE3943R
Raw Viewer - [Dataset1]
File
Tools
Window
Help
Raw Files
Map
MS
MS/MS
XIC
TAGMCpTTYAAHLVAEELR; Scan 10307; 20061206_BM_LTQ-Orbitrap2_Halobacterium_05
m/z (full)
Intensity
m/z (zoom)
Slide 11 of 11
"Office Theme"
English (U.S.)
Start
Raw Viewer - [Dataset1]
Result Explorer
Microsoft PowerPoint - [...
17:27

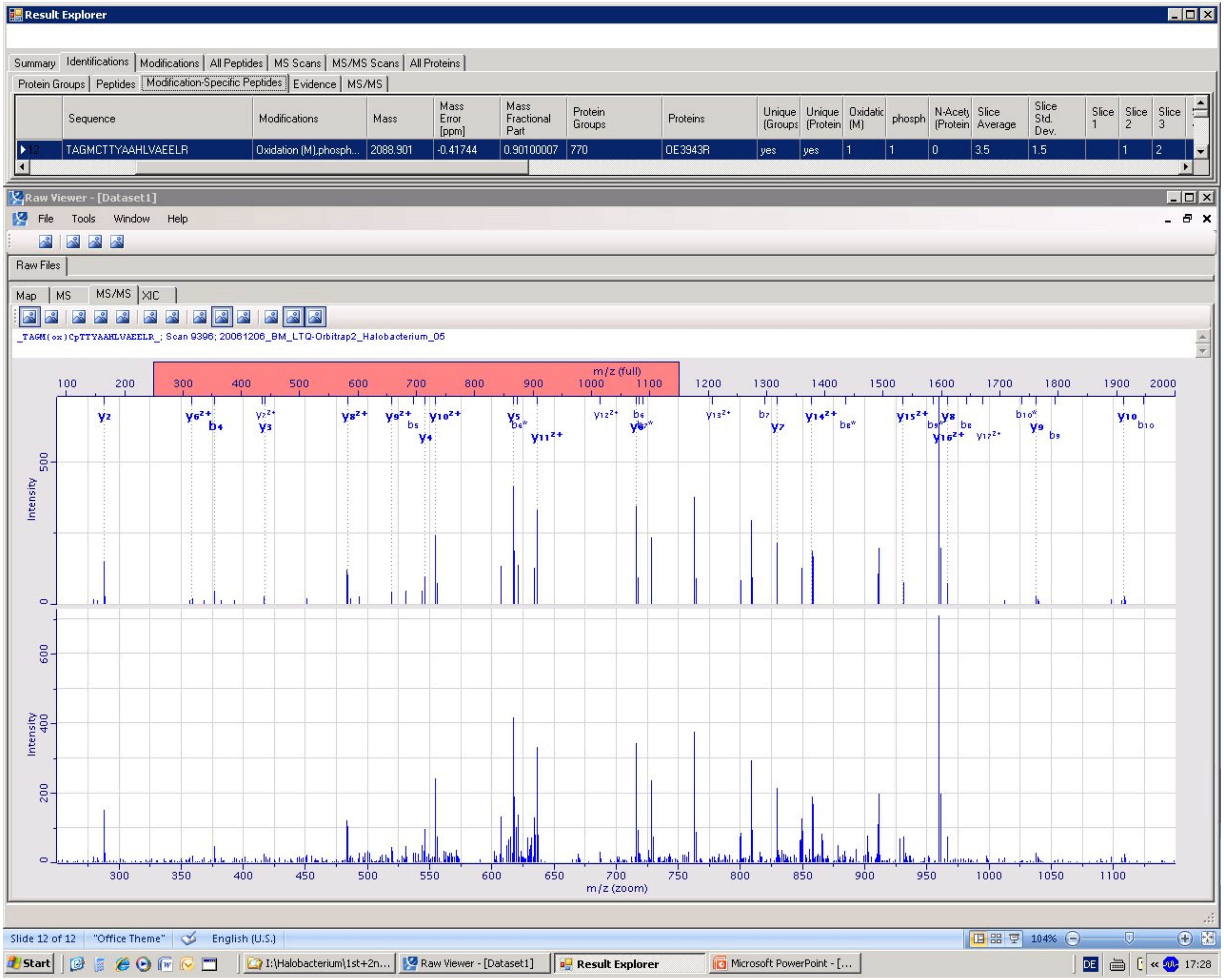
Result Explorer
Summary | Identifications | Modifications | All Peptides | MS Scans | MS/MS Scans | All Proteins
Protein Groups | Peptides | Modification-Specific Peptides | Evidence | MS/MS
Sequence | Modifications | Mass | Mass Error [ppm] | Mass Fractional Part | Protein Groups | Proteins | Unique (Groups | Unique (Protein | Oxidatic (M) | phosph | N-Acety (Protein | Slice Average | Slice Std. Dev. | Slice 1 | Slice 2 | Slice 3
TAGMCTTYAAHLVAEELR | Oxidation (M),phosph... | 2088.901 | -0.41744 | 0.90100007 | 770 | OE3943R | yes | yes | 1 | 1 | 0 | 3.5 | 1.5 | | 1 | 2
Raw Viewer - [Dataset1]
File Tools Window Help
Raw Files
Map MS MS/MS XIC
TAGM(ox)CpTTYAAHLVAEELR; Scan 9396; 20061206_BM_LTQ-Orbitrap2_Halobacterium_05
m/z (full)
Intensity
m/z (zoom)
Slide 12 of 12 "Office Theme" English (U.S.) 104%
Start I:\Halobacterium\1st+2n... Raw Viewer - [Dataset1] Result Explorer Microsoft PowerPoint - [...
DE 17:28

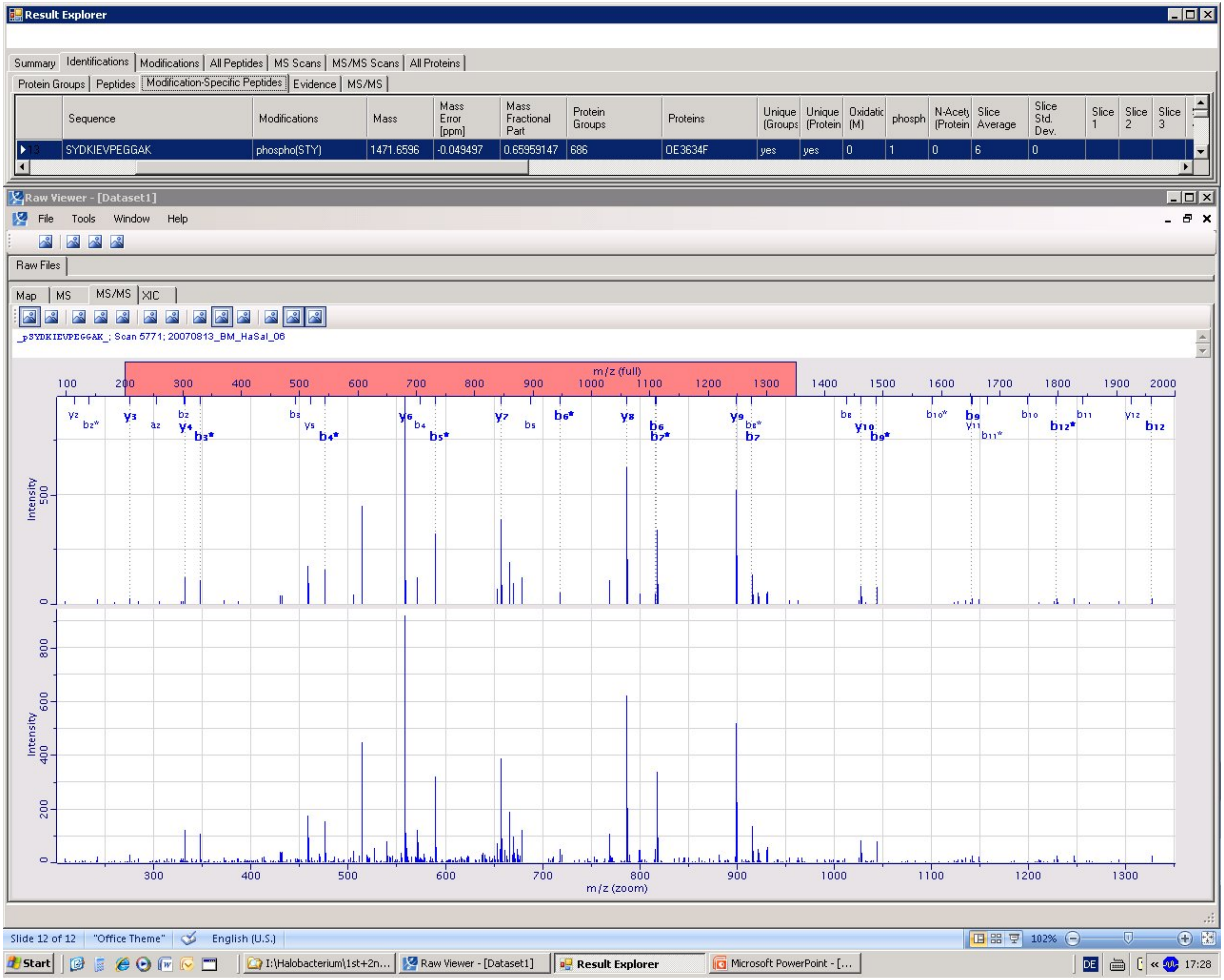
Result Explorer
Summary
Identifications
Modifications
All Peptides
MS Scans
MS/MS Scans
All Proteins
Protein Groups
Peptides
Modification-Specific Peptides
Evidence
MS/MS
Sequence
Modifications
Mass
Mass Error [ppm]
Mass Fractional Part
Protein Groups
Proteins
Unique (Groups
Unique (Protein
Oxidatic (M)
phosph
N-Acety (Protein
Slice Average
Slice Std. Dev.
Slice 1
Slice 2
Slice 3
SYDKIEVPEGGAK
phospho(STY)
1471.6596
-0.049497
0.65959147
686
OE3634F
yes
yes
0
1
0
6
0
Raw Viewer - [Dataset1]
File
Tools
Window
Help
Raw Files
Map
MS
MS/MS
XIC
pSYDKIEVPEGGAK; Scan 5771; 20070813_BM_HaSal_06
m/z (full)
Intensity
m/z (zoom)
Slide 12 of 12
"Office Theme"
English (U.S.)
102%
Start
I:\Halobacterium\1st+2n...
Raw Viewer - [Dataset1]
Result Explorer
Microsoft PowerPoint - [...
DE
17:28

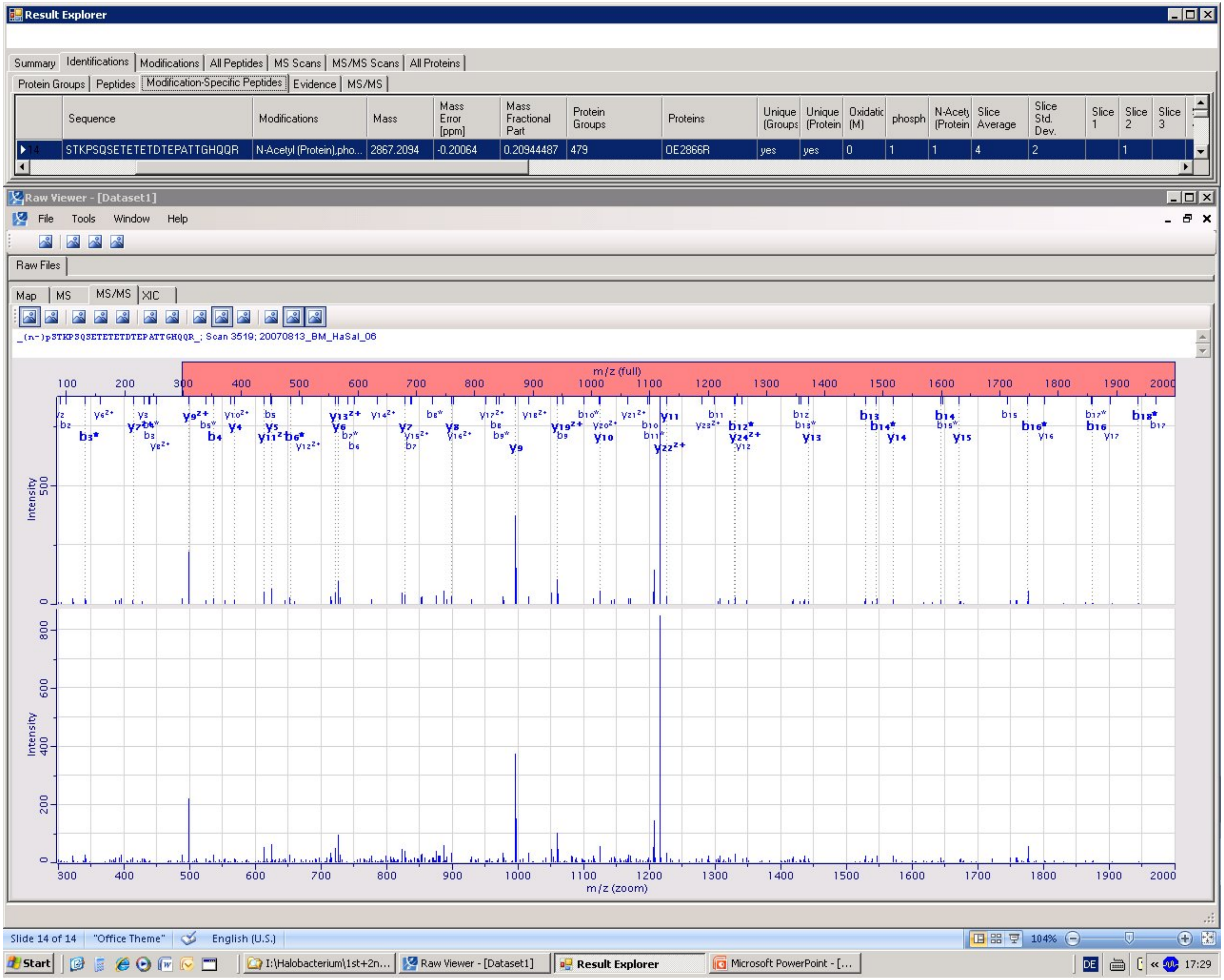
Result Explorer
Summary
Identifications
Modifications
All Peptides
MS Scans
MS/MS Scans
All Proteins
Protein Groups
Peptides
Modification-Specific Peptides
Evidence
MS/MS
Sequence
Modifications
Mass
Mass Error [ppm]
Mass Fractional Part
Protein Groups
Proteins
STKPSQSETETETDTEPATTGHQQR
N-Acetyl (Protein),pho...
2867.2094
-0.20064
0.20944487
479
OE2866R
Raw Viewer - [Dataset1]
File
Tools
Window
Help
Raw Files
Map
MS
MS/MS
XIC
(n-)pSTKPSQSETETETDTEPATTGHQQR; Scan 3519; 20070813_BM_HaSal_06
m/z (full)
Intensity
m/z (zoom)
Slide 14 of 14
"Office Theme"
English (U.S.)
Start
Raw Viewer - [Dataset1]
Result Explorer
17:29

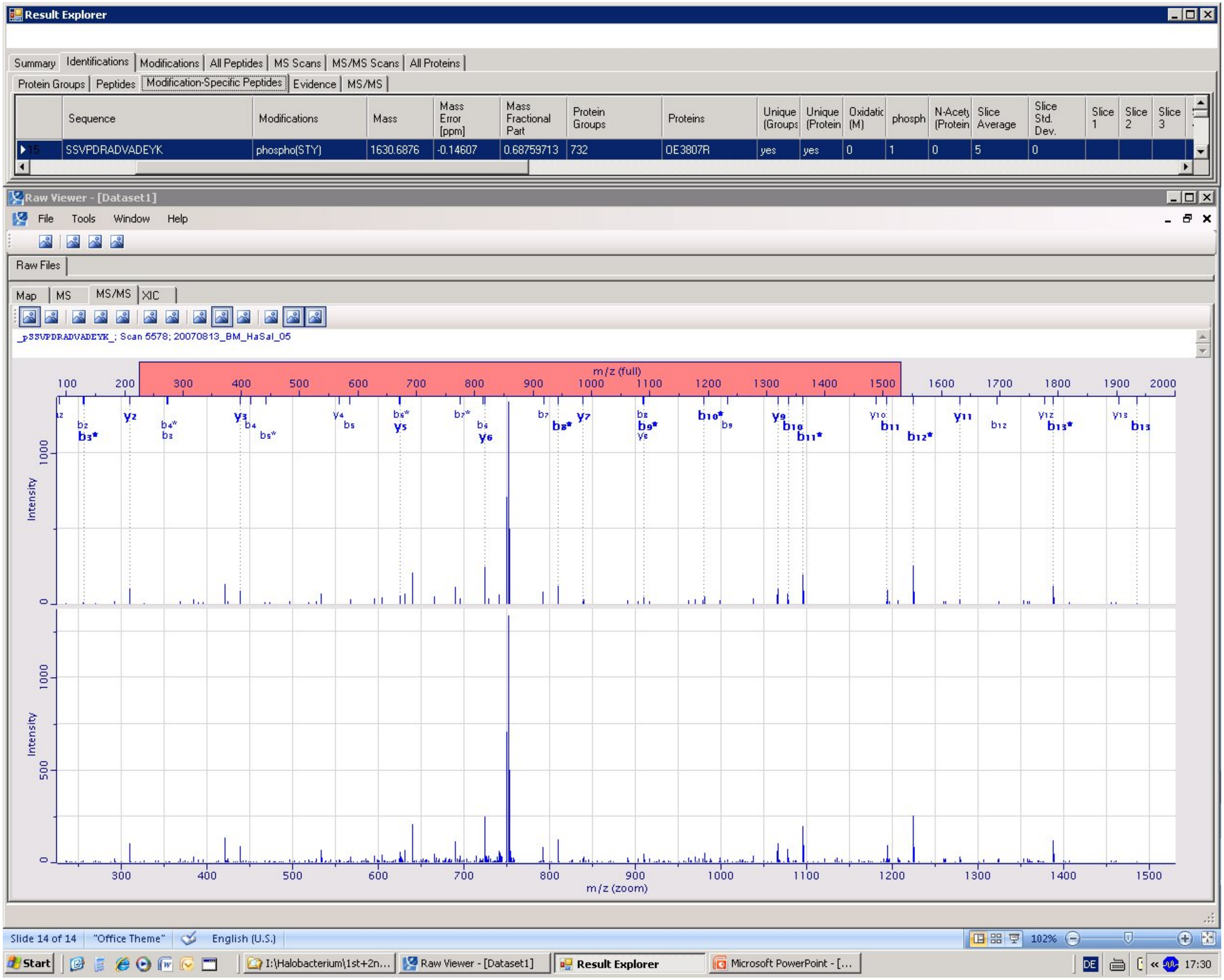
Result Explorer
Summary | Identifications | Modifications | All Peptides | MS Scans | MS/MS Scans | All Proteins
Protein Groups | Peptides | Modification-Specific Peptides | Evidence | MS/MS
Sequence | Modifications | Mass | Mass Error [ppm] | Mass Fractional Part | Protein Groups | Proteins | Unique (Groups | Unique (Protein | Oxidatic (M) | phosph | N-Acety (Protein | Slice Average | Slice Std. Dev. | Slice 1 | Slice 2 | Slice 3
SSVPDRADVADEYK | phospho(STY) | 1630.6876 | -0.14607 | 0.68759713 | 732 | OE3807R | yes | yes | 0 | 1 | 0 | 5 | 0
Raw Viewer - [Dataset1]
File Tools Window Help
Raw Files
Map MS MS/MS XIC
pSSVPDRADVADEYK; Scan 5578; 20070813_BM_HaSal_05
m/z (full)
Intensity
m/z (zoom)
Slide 14 of 14 "Office Theme" English (U.S.)
Start
I:\Halobacterium\1st+2n...
Raw Viewer - [Dataset1]
Result Explorer
Microsoft PowerPoint - [...
17:30

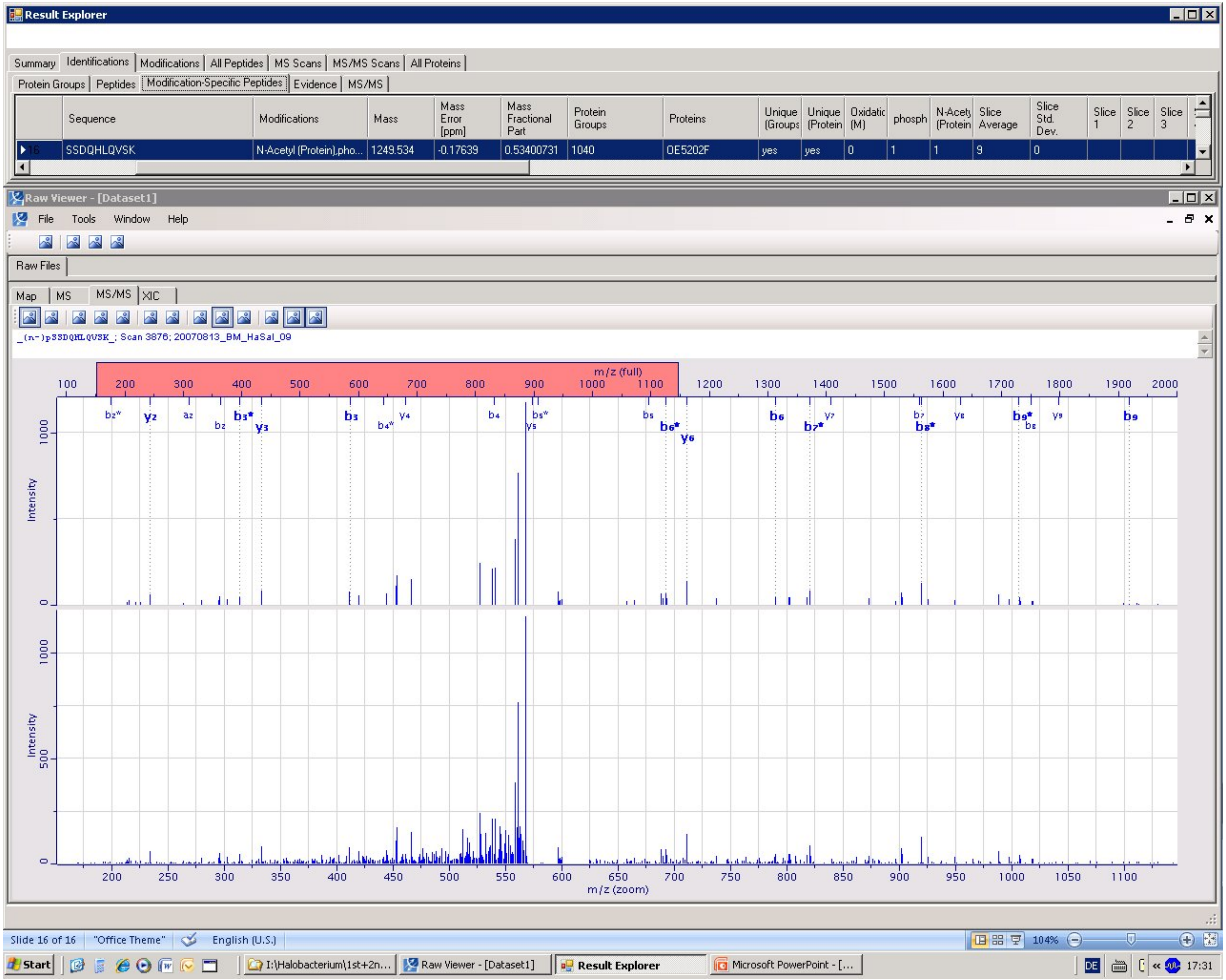
Result Explorer
Summary
Identifications
Modifications
All Peptides
MS Scans
MS/MS Scans
All Proteins
Protein Groups
Peptides
Modification-Specific Peptides
Evidence
MS/MS
Sequence
Modifications
Mass
Mass Error [ppm]
Mass Fractional Part
Protein Groups
Proteins
Unique (Groups
Unique (Protein
Oxidatic (M)
phosph
N-Acety (Protein
Slice Average
Slice Std. Dev.
Slice 1
Slice 2
Slice 3
SSDQHLQVSK
N-Acetyl (Protein),pho...
1249.534
-0.17639
0.53400731
1040
OE5202F
yes
yes
0
1
1
9
0
Raw Viewer - [Dataset1]
File
Tools
Window
Help
Raw Files
Map
MS
MS/MS
XIC
(n-)pSSDQHLQVSK; Scan 3876; 20070813_BM_HaSal_09
m/z (full)
Intensity
m/z (zoom)
Slide 16 of 16
"Office Theme"
English (U.S.)
104%
Start
I:\Halobacterium\1st+2n...
Raw Viewer - [Dataset1]
Result Explorer
Microsoft PowerPoint - [...
DE
17:31

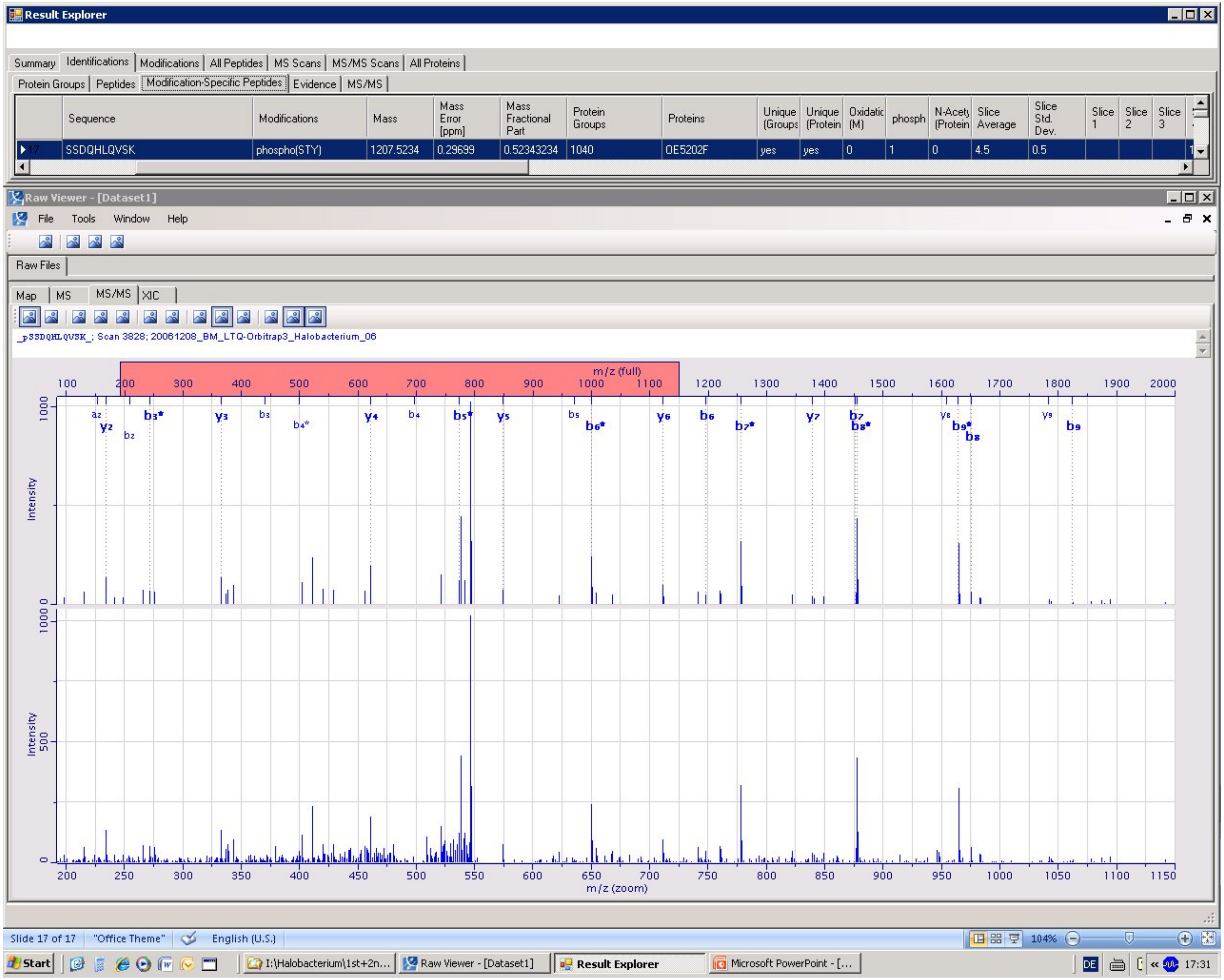
Result Explorer
Summary
Identifications
Modifications
All Peptides
MS Scans
MS/MS Scans
All Proteins
Protein Groups
Peptides
Modification-Specific Peptides
Evidence
MS/MS
Sequence
Modifications
Mass
Mass Error [ppm]
Mass Fractional Part
Protein Groups
Proteins
Unique (Groups
Unique (Protein
Oxidatic (M)
phosph
N-Acety (Protein
Slice Average
Slice Std. Dev.
Slice 1
Slice 2
Slice 3
SSDQHLQVSK
phospho(STY)
1207.5234
0.29699
0.52343234
1040
OE5202F
yes
yes
0
1
0
4.5
0.5
Raw Viewer - [Dataset1]
File
Tools
Window
Help
Raw Files
Map
MS
MS/MS
XIC
pSSDQHLQVSK; Scan 3828; 20061208_BM_LTQ-Orbitrap3_Halobacterium_06
m/z (full)
Intensity
m/z (zoom)
Slide 17 of 17
"Office Theme"
English (U.S.)
104%
Start
I:\Halobacterium\1st+2n...
Raw Viewer - [Dataset1]
Result Explorer
Microsoft PowerPoint - [...
17:31

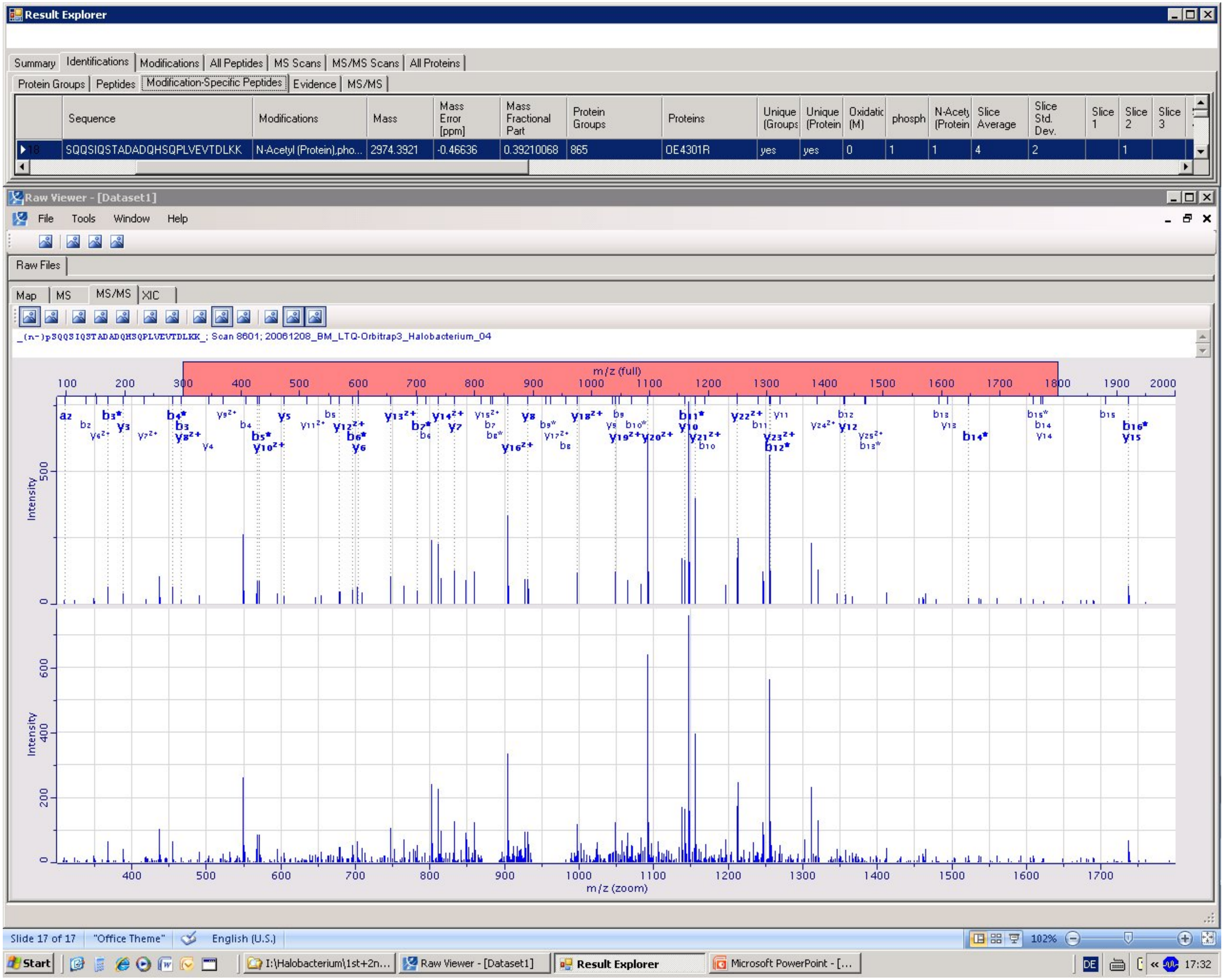
Result Explorer
Summary | Identifications | Modifications | All Peptides | MS Scans | MS/MS Scans | All Proteins
Protein Groups | Peptides | Modification-Specific Peptides | Evidence | MS/MS
Sequence | Modifications | Mass | Mass Error [ppm] | Mass Fractional Part | Protein Groups | Proteins | Unique (Groups | Unique (Protein | Oxidatic (M) | phosph | N-Acety (Protein | Slice Average | Slice Std. Dev. | Slice 1 | Slice 2 | Slice 3
SQQSIQSTADADQHSQPLVEVTDLKK | N-Acetyl (Protein),pho... | 2974.3921 | -0.46636 | 0.39210068 | 865 | OE4301R | yes | yes | 0 | 1 | 1 | 4 | 2 | | 1
Raw Viewer - [Dataset1]
File Tools Window Help
Raw Files
Map MS MS/MS XIC
(n-)pSQQSIQSTADADQHSQPLVEVTDLKK; Scan 8601; 20081208_BM_LTQ-Orbitrap3_Halobacterium_04
m/z (full)
Intensity
m/z (zoom)
Slide 17 of 17 "Office Theme" English (U.S.)
Start
I:\Halobacterium\1st+2n...
Raw Viewer - [Dataset1]
Result Explorer
Microsoft PowerPoint - [...
DE
17:32

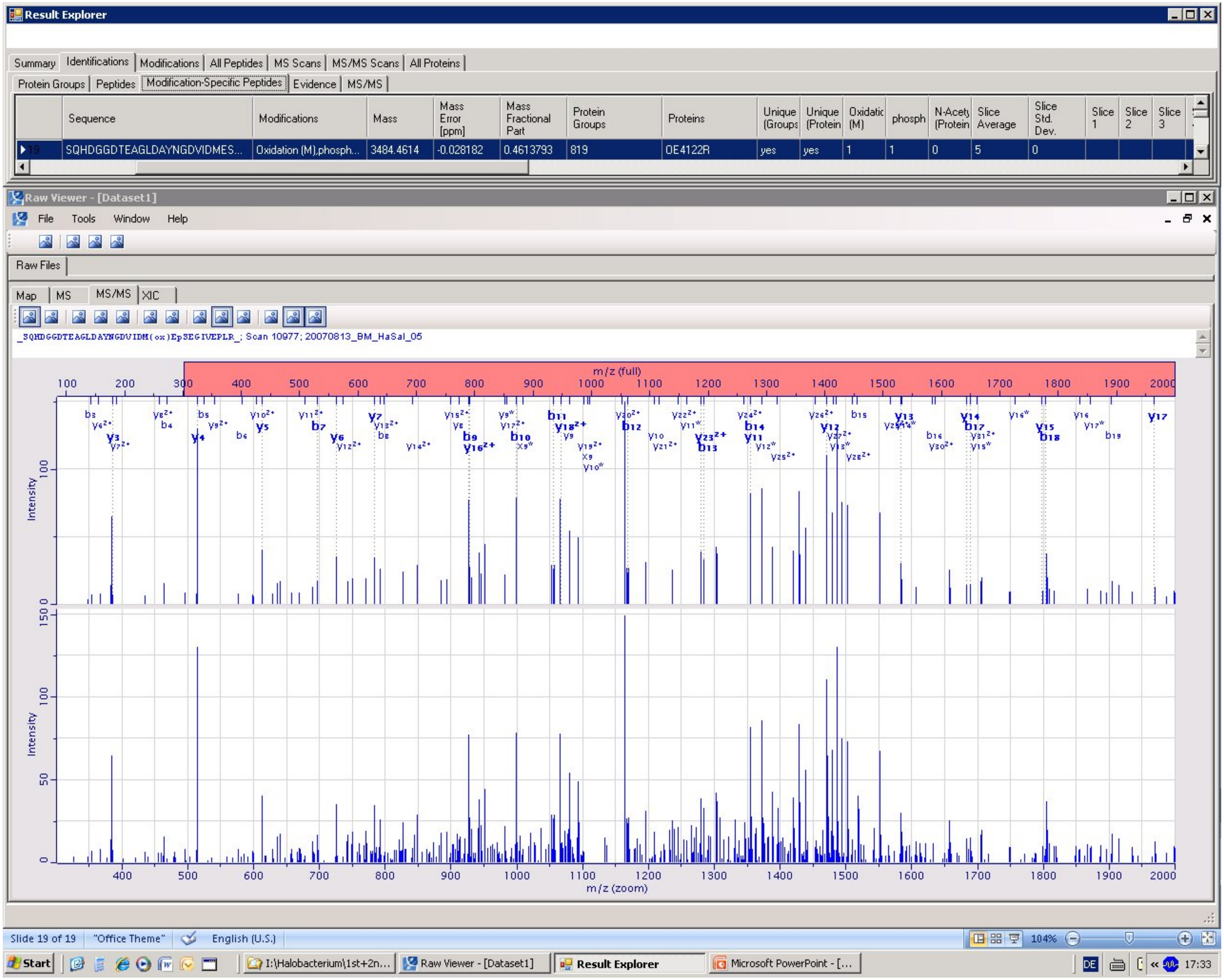
Result Explorer
Summary
Identifications
Modifications
All Peptides
MS Scans
MS/MS Scans
All Proteins
Protein Groups
Peptides
Modification-Specific Peptides
Evidence
MS/MS
Sequence
Modifications
Mass
Mass Error [ppm]
Mass Fractional Part
Protein Groups
Proteins
SQHDGGDTEAGLDAYNGDVIDMES...
Oxidation (M),phosph...
3484.4614
-0.028182
0.4613793
819
OE4122R
Raw Viewer - [Dataset1]
File
Tools
Window
Help
Raw Files
Map
MS
MS/MS
XIC
SQHDGGDTEAGLDAYNGDVIDM(ox)EpSEGIVEPLR; Scan 10977; 20070813_BM_HaSal_05
m/z (full)
Intensity
m/z (zoom)
Slide 19 of 19
"Office Theme"
English (U.S.)
Start
Raw Viewer - [Dataset1]
Result Explorer
17:33

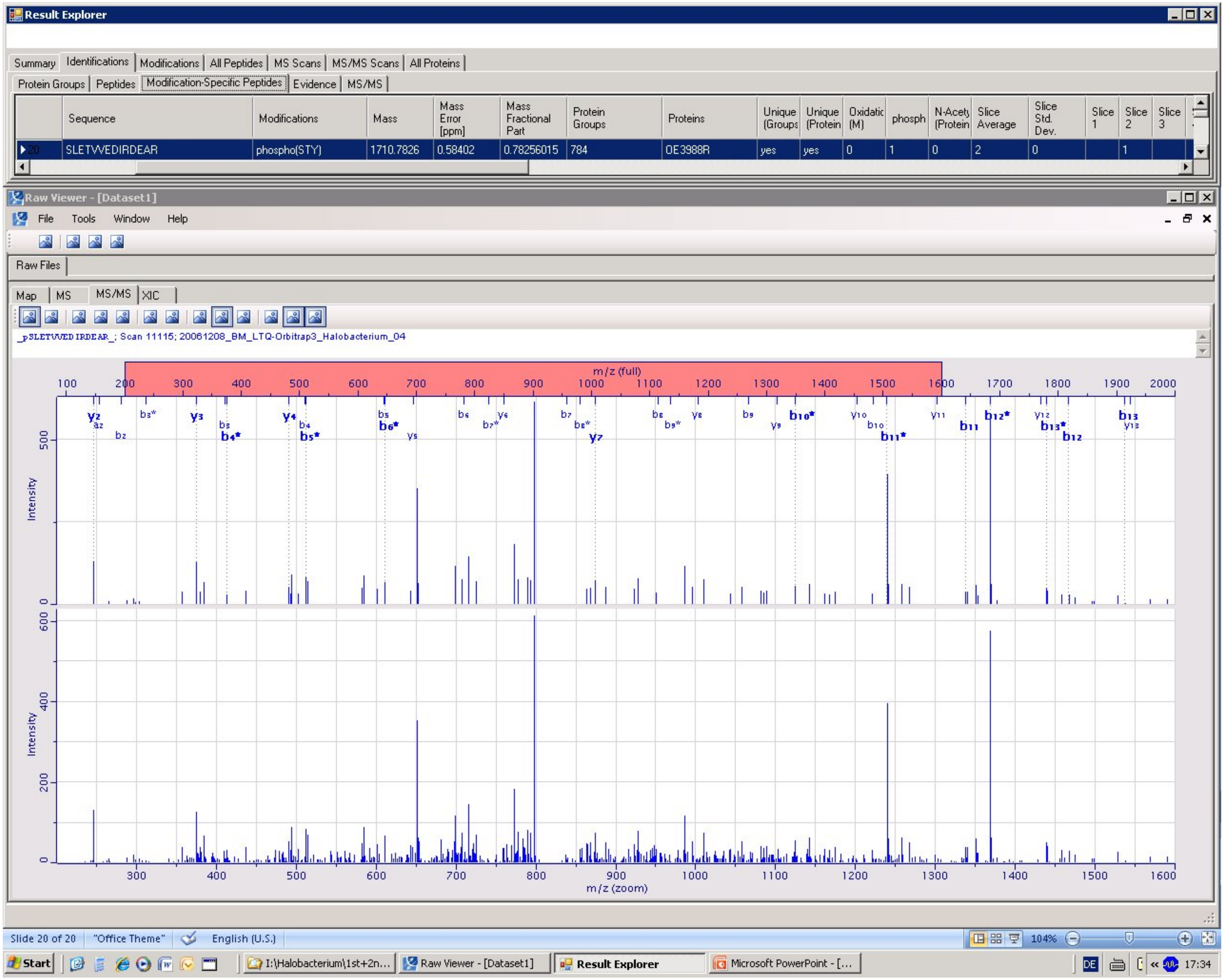
Result Explorer
Summary
Identifications
Modifications
All Peptides
MS Scans
MS/MS Scans
All Proteins
Protein Groups
Peptides
Modification-Specific Peptides
Evidence
MS/MS
Sequence
Modifications
Mass
Mass Error [ppm]
Mass Fractional Part
Protein Groups
Proteins
Unique (Groups
Unique (Protein
Oxidatic (M)
phosph
N-Acety (Protein
Slice Average
Slice Std. Dev.
Slice 1
Slice 2
Slice 3
SLETVVEDIRDEAR
phospho(STY)
1710.7826
0.58402
0.78256015
784
OE3988R
yes
yes
0
1
0
2
0
1
Raw Viewer - [Dataset1]
File
Tools
Window
Help
Raw Files
Map
MS
MS/MS
XIC
pSLETVVEDIRDEAR; Scan 11115; 20061208_BM_LTQ-Orbitrap3_Halobacterium_04
m/z (full)
Intensity
m/z (zoom)
Slide 20 of 20
"Office Theme"
English (U.S.)
104%
Start
I:\Halobacterium\1st+2n...
Raw Viewer - [Dataset1]
Result Explorer
Microsoft PowerPoint - [...
DE
17:34

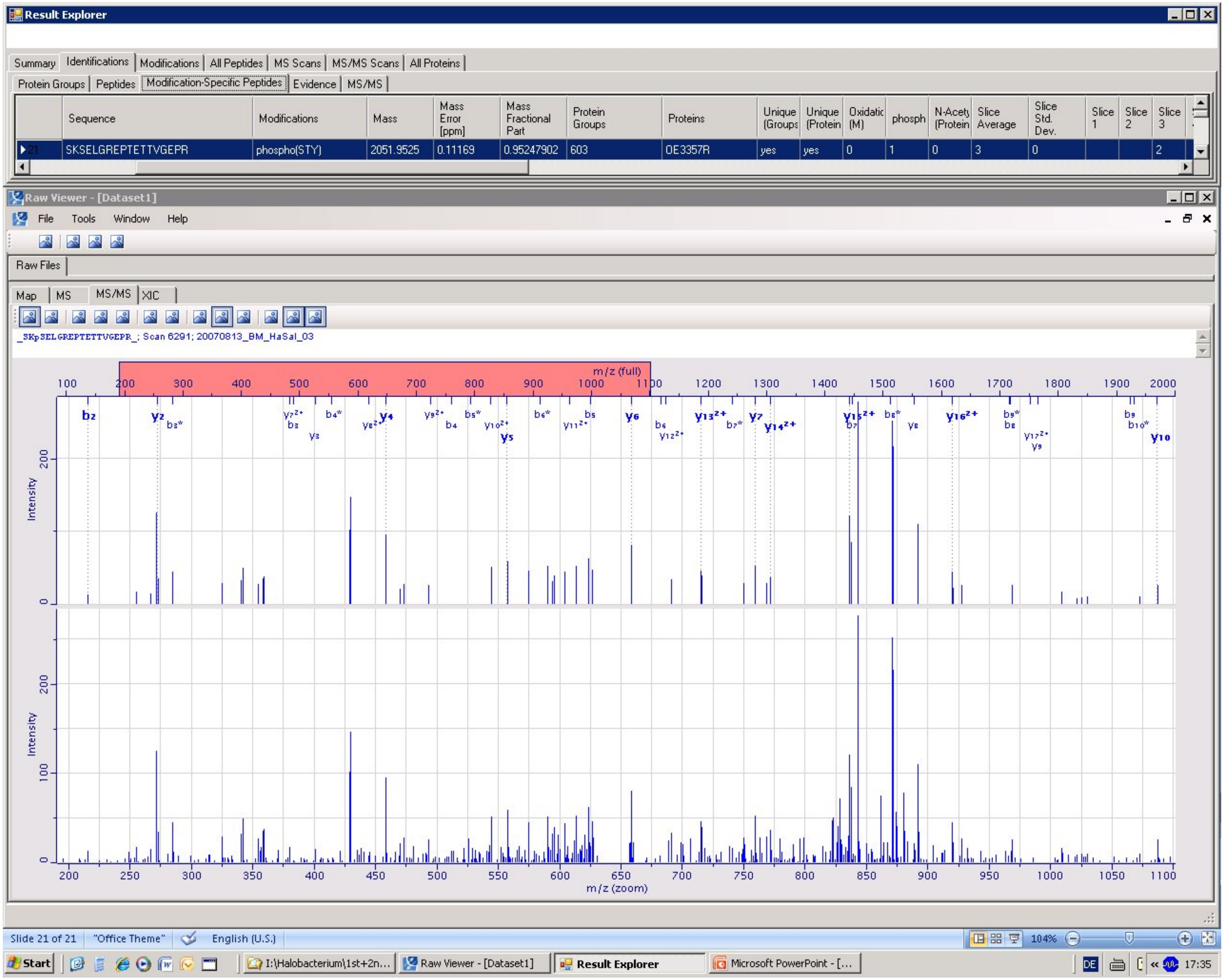
Result Explorer
Summary | Identifications | Modifications | All Peptides | MS Scans | MS/MS Scans | All Proteins
Protein Groups | Peptides | Modification-Specific Peptides | Evidence | MS/MS
Sequence | Modifications | Mass | Mass Error [ppm] | Mass Fractional Part | Protein Groups | Proteins | Unique (Groups | Unique (Protein | Oxidatic (M) | phosph | N-Acety (Protein | Slice Average | Slice Std. Dev. | Slice 1 | Slice 2 | Slice 3
21 | SKSELGREPTETTVGEPR | phospho(STY) | 2051.9525 | 0.11169 | 0.95247902 | 603 | OE3357R | yes | yes | 0 | 1 | 0 | 3 | 0 | | | 2
Raw Viewer - [Dataset1]
File Tools Window Help
Raw Files
Map MS MS/MS XIC
SKpSELGREPTETTVGEPR; Scan 6291; 20070813_BM_HaSal_03
m/z (full)
Intensity
m/z (zoom)
Slide 21 of 21 "Office Theme" English (U.S.) 104%
Start I:\Halobacterium\1st+2n... Raw Viewer - [Dataset1] Result Explorer Microsoft PowerPoint - [... 17:35

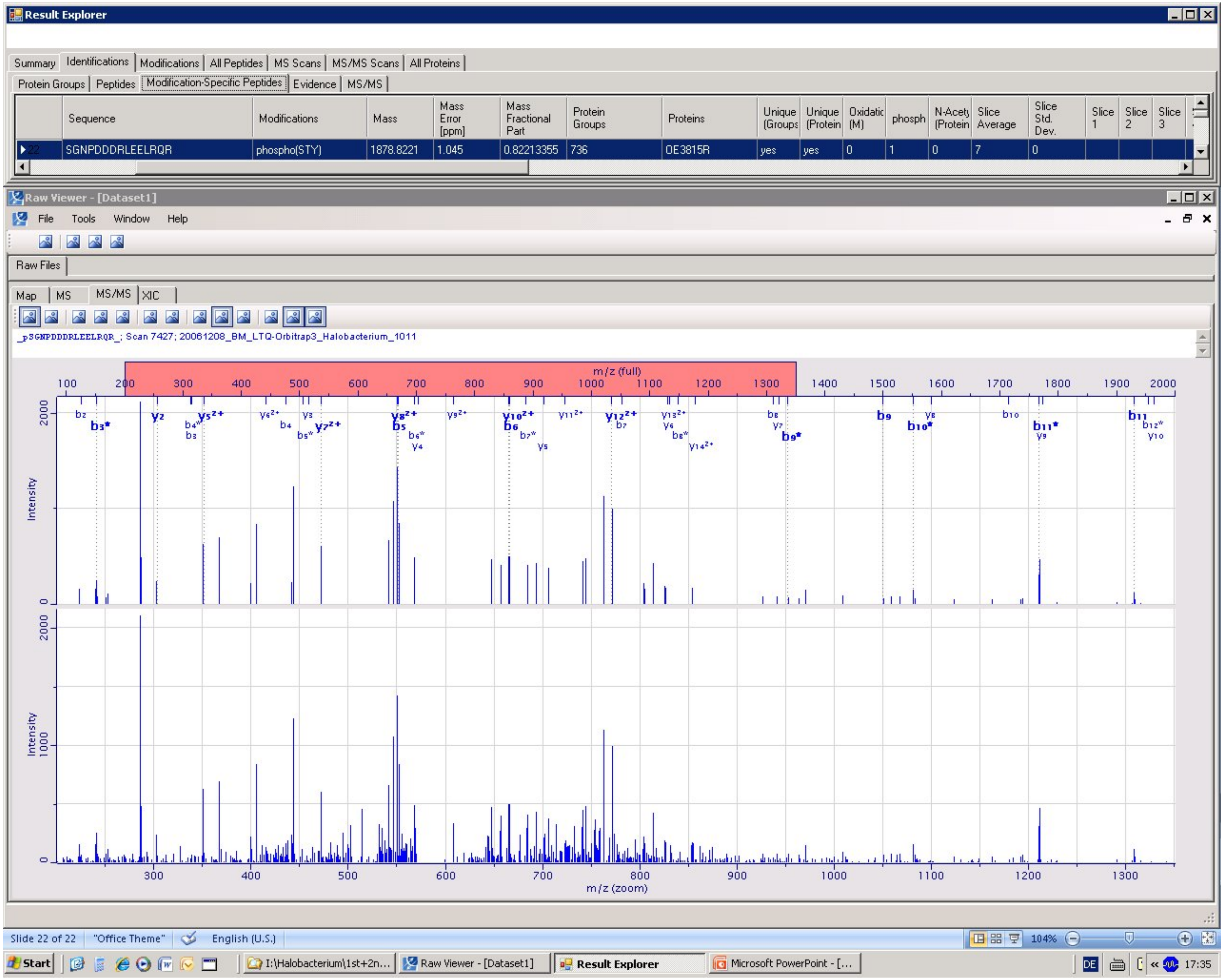
Result Explorer
Summary | Identifications | Modifications | All Peptides | MS Scans | MS/MS Scans | All Proteins
Protein Groups | Peptides | Modification-Specific Peptides | Evidence | MS/MS
Sequence | Modifications | Mass | Mass Error [ppm] | Mass Fractional Part | Protein Groups | Proteins | Unique (Groups | Unique (Protein | Oxidatic (M) | phosph | N-Acety (Protein | Slice Average | Slice Std. Dev. | Slice 1 | Slice 2 | Slice 3
SGNPDDDRLEELRQR | phospho(STY) | 1878.8221 | 1.045 | 0.82213355 | 736 | OE3815R | yes | yes | 0 | 1 | 0 | 7 | 0
Raw Viewer - [Dataset1]
File Tools Window Help
Raw Files
Map MS MS/MS XIC
pSGNPDDDRLEELRQR; Scan 7427; 20061208_BM_LTQ-Orbitrap3_Halobacterium_1011
m/z (full)
Intensity
m/z (zoom)
Slide 22 of 22 "Office Theme" English (U.S.)
Start
Raw Viewer - [Dataset1]
Result Explorer
Microsoft PowerPoint - [...
17:35

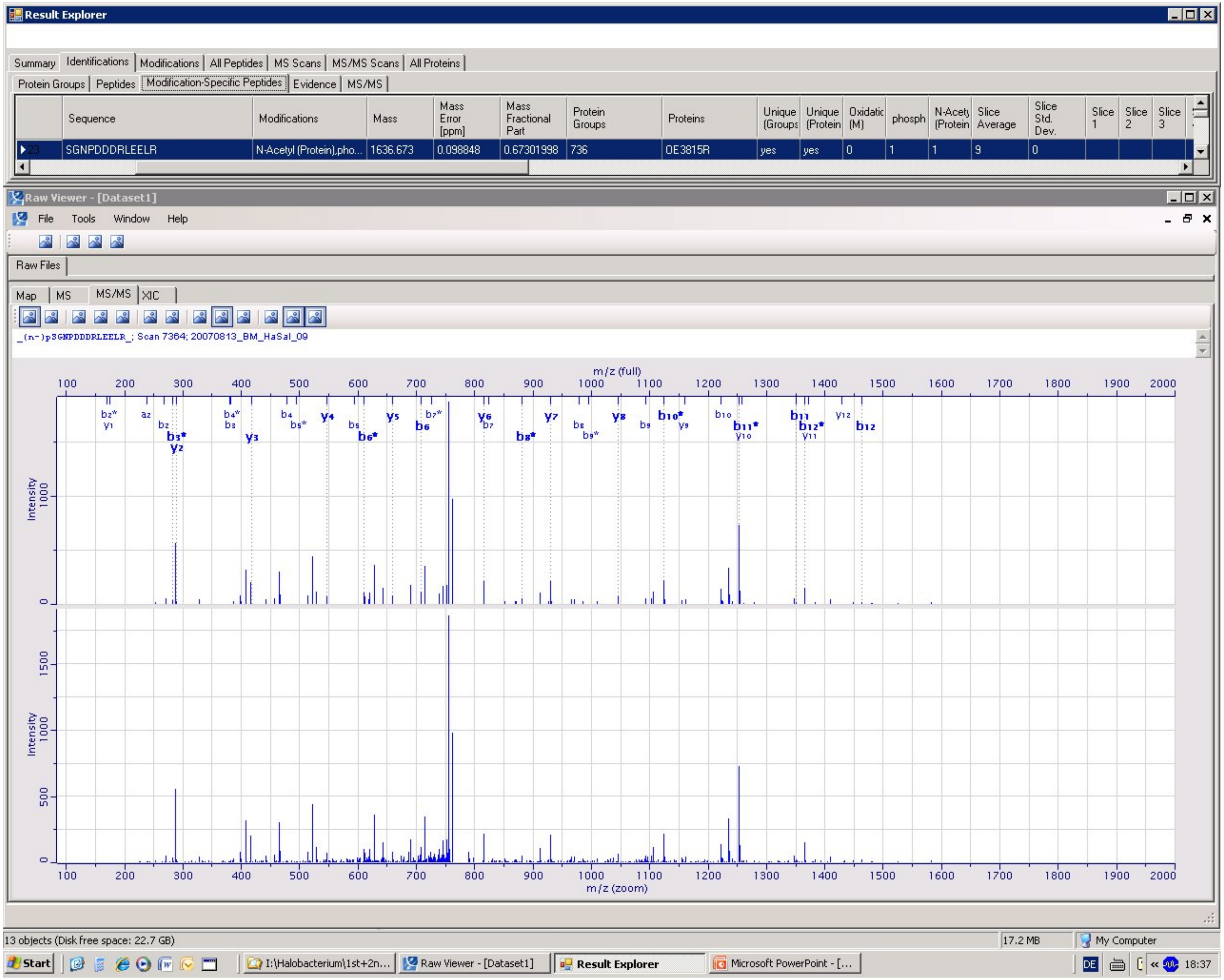
Result Explorer
Summary
Identifications
Modifications
All Peptides
MS Scans
MS/MS Scans
All Proteins
Protein Groups
Peptides
Modification-Specific Peptides
Evidence
MS/MS
Sequence
Modifications
Mass
Mass Error [ppm]
Mass Fractional Part
Protein Groups
Proteins
Unique (Groups
Unique (Protein
Oxidatic (M)
phosph
N-Acety (Protein
Slice Average
Slice Std. Dev.
Slice 1
Slice 2
Slice 3
SGNPDDDRLEELR
N-Acetyl (Protein),pho...
1636.673
0.098848
0.67301998
736
OE3815R
yes
yes
0
1
1
9
0
Raw Viewer - [Dataset1]
File
Tools
Window
Help
Raw Files
Map
MS
MS/MS
XIC
(n-)pSGNPDDDRLEELR; Scan 7364; 20070813_BM_HaSal_09
m/z (full)
Intensity
m/z (zoom)
13 objects (Disk free space: 22.7 GB)
17.2 MB
My Computer
Start
I:\Halobacterium\1st+2n...
Raw Viewer - [Dataset1]
Result Explorer
Microsoft PowerPoint - [...
18:37

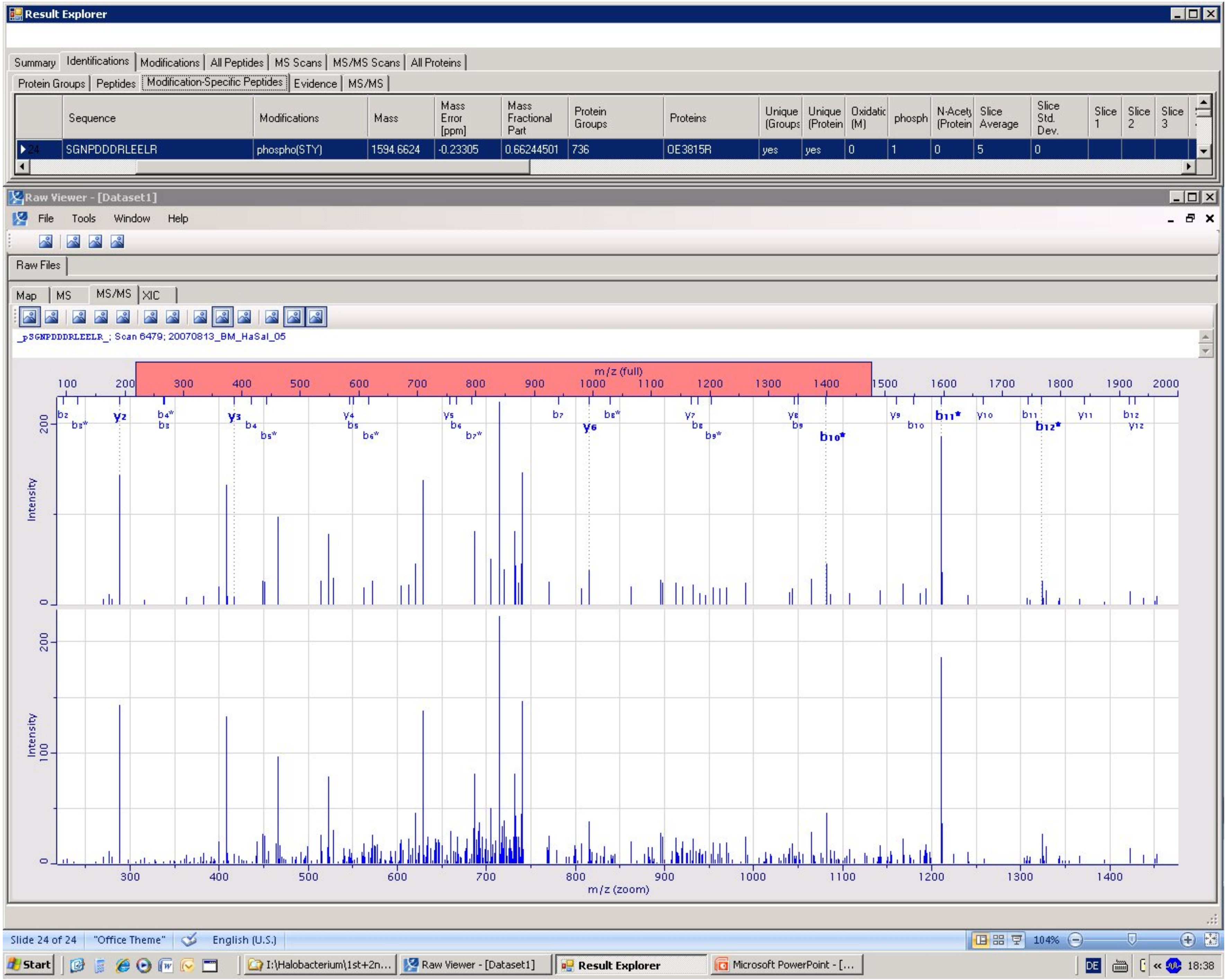

Result Explorer
Summary
Identifications
Modifications
All Peptides
MS Scans
MS/MS Scans
All Proteins
Protein Groups
Peptides
Modification-Specific Peptides
Evidence
MS/MS
Sequence
Modifications
Mass
Mass Error [ppm]
Mass Fractional Part
Protein Groups
Proteins
SGNPDDDRLEELR
phospho(STY)
1594.6624
-0.23305
0.66244501
736
OE3815R
Raw Viewer - [Dataset1]
File
Tools
Window
Help
Raw Files
Map
MS
MS/MS
XIC
pSGNPDDDRLEELR; Scan 6479; 20070813_BM_HaSal_05
m/z (full)
Intensity
m/z (zoom)
Slide 24 of 24
"Office Theme"
English (U.S.)
Start
Raw Viewer - [Dataset1]
Result Explorer
18:38

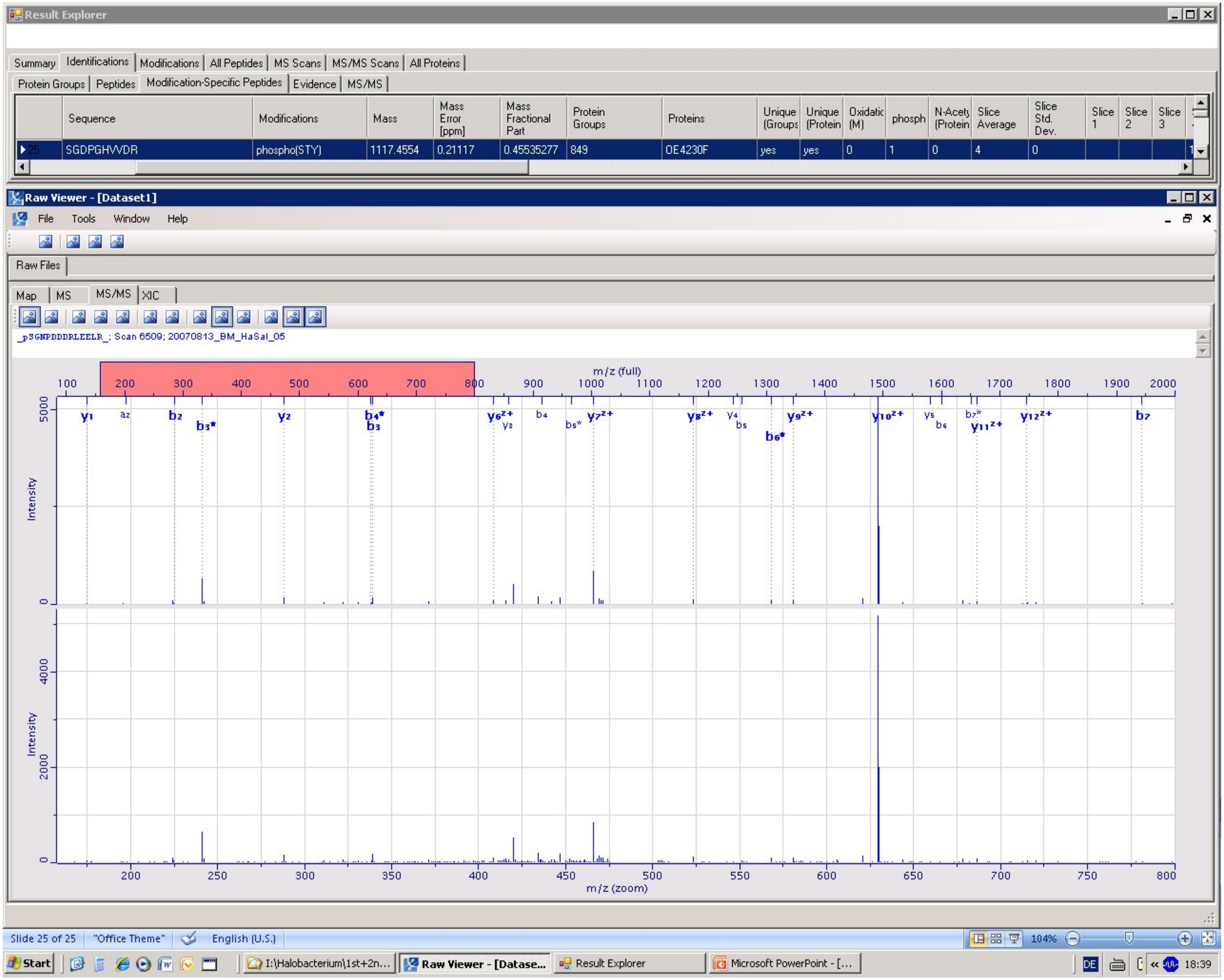
Result Explorer
Summary | Identifications | Modifications | All Peptides | MS Scans | MS/MS Scans | All Proteins
Protein Groups | Peptides | Modification-Specific Peptides | Evidence | MS/MS
Sequence | Modifications | Mass | Mass Error [ppm] | Mass Fractional Part | Protein Groups | Proteins | Unique (Groups | Unique (Protein | Oxidatic (M) | phosph | N-Acety (Protein | Slice Average | Slice Std. Dev. | Slice 1 | Slice 2 | Slice 3
SGDPGHVVDR | phospho(STY) | 1117.4554 | 0.21117 | 0.45535277 | 849 | OE4230F | yes | yes | 0 | 1 | 0 | 4 | 0
Raw Viewer - [Dataset1]
File Tools Window Help
Raw Files
Map MS MS/MS XIC
pSGNPDDDRLEELR; Scan 6509; 20070813_BM_HaSal_05
m/z (full)
Intensity
m/z (zoom)
Slide 25 of 25 "Office Theme" English (U.S.)
Start
I:\Halobacterium\1st+2n...
Raw Viewer - [Datase...
Result Explorer
Microsoft PowerPoint - [...
18:39

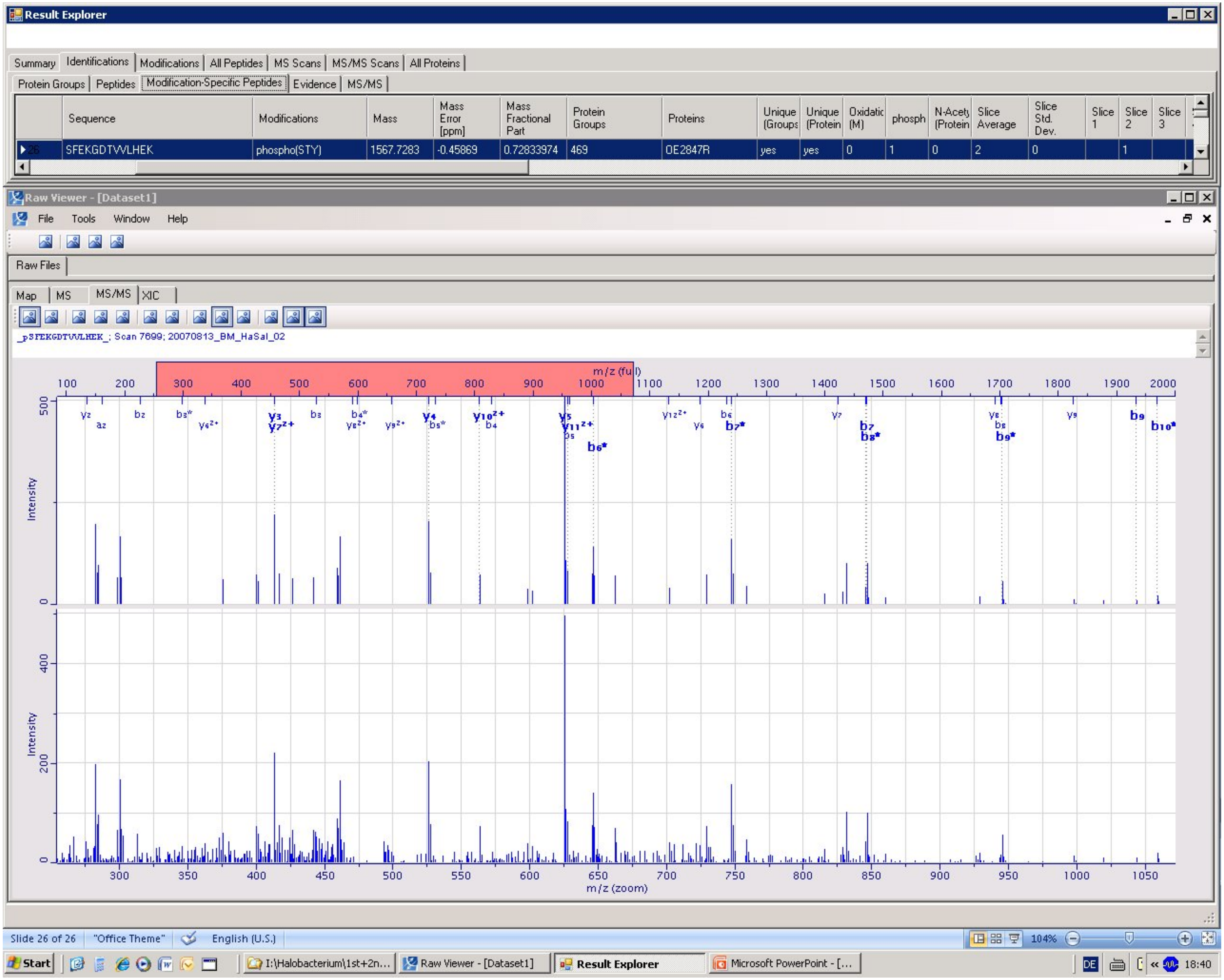

Result Explorer
Summary | Identifications | Modifications | All Peptides | MS Scans | MS/MS Scans | All Proteins
Protein Groups | Peptides | Modification-Specific Peptides | Evidence | MS/MS
Sequence | Modifications | Mass | Mass Error [ppm] | Mass Fractional Part | Protein Groups | Proteins | Unique (Groups | Unique (Protein | Oxidatic (M) | phosph | N-Acety (Protein | Slice Average | Slice Std. Dev. | Slice 1 | Slice 2 | Slice 3
SFEKGDTVVLHEK | phospho(STY) | 1567.7283 | -0.45869 | 0.72833974 | 469 | OE2847R | yes | yes | 0 | 1 | 0 | 2 | 0 | | 1
Raw Viewer - [Dataset1]
File Tools Window Help
Raw Files
Map MS MS/MS XIC
pSFEKGDTVVLHEK; Scan 7699; 20070813_BM_HaSal_02
m/z (full)
Intensity
m/z (zoom)
Slide 26 of 26 "Office Theme" English (U.S.) 104%
Start I:\Halobacterium\1st+2n... Raw Viewer - [Dataset1] Result Explorer Microsoft PowerPoint - [...
18:40

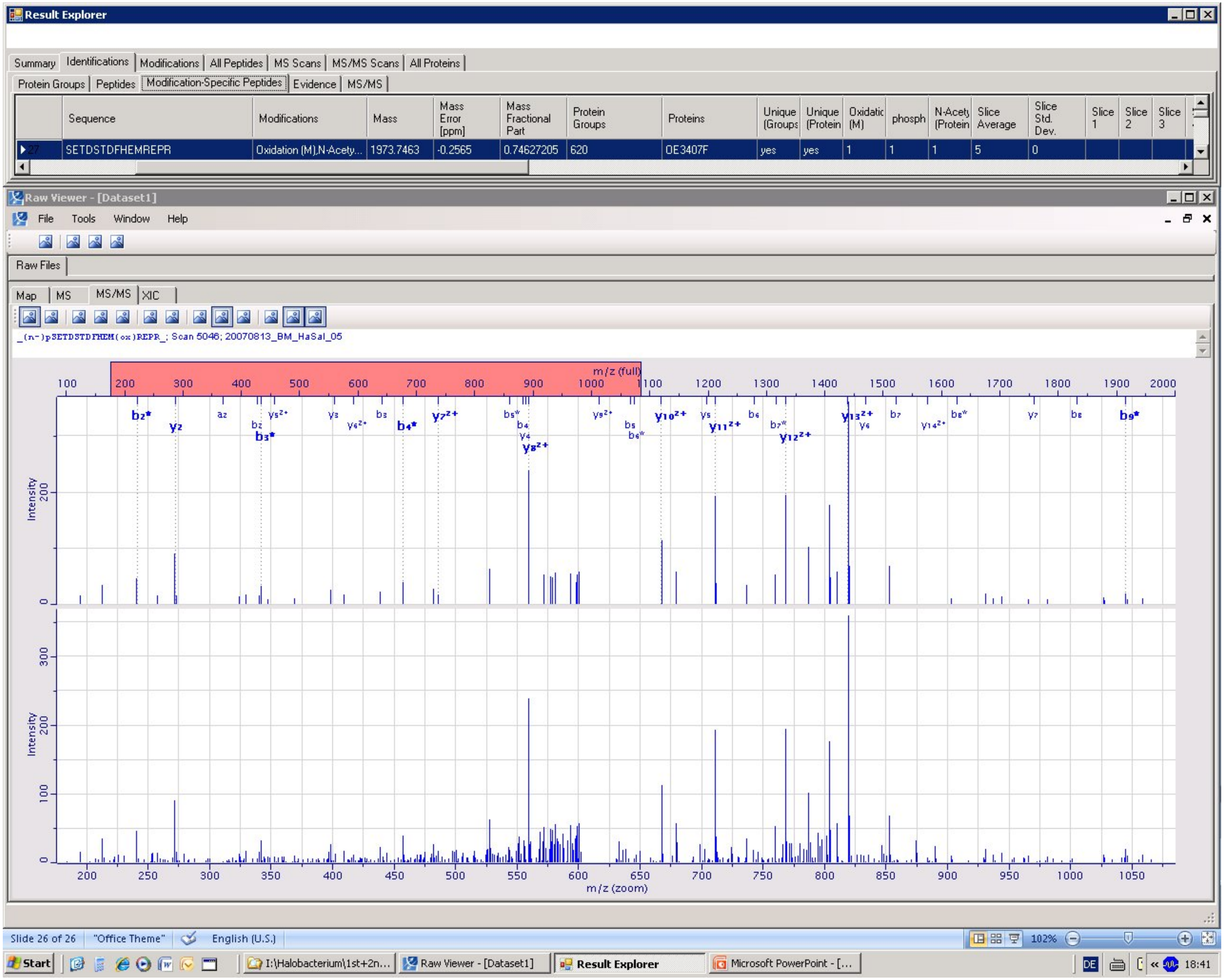
Result Explorer
Summary
Identifications
Modifications
All Peptides
MS Scans
MS/MS Scans
All Proteins
Protein Groups
Peptides
Modification-Specific Peptides
Evidence
MS/MS
Sequence
Modifications
Mass
Mass Error [ppm]
Mass Fractional Part
Protein Groups
Proteins
Unique (Groups
Unique (Protein
Oxidatic (M)
phosph
N-Acety (Protein
Slice Average
Slice Std. Dev.
Slice 1
Slice 2
Slice 3
SETDSTDFHEMREPR
Oxidation (M),N-Acety...
1973.7463
-0.2565
0.74627205
620
OE3407F
yes
yes
1
1
1
5
0
Raw Viewer - [Dataset1]
File
Tools
Window
Help
Raw Files
Map
MS
MS/MS
XIC
(n-)pSETDSTDFHEM(ox)REPR; Scan 5046; 20070813_BM_HaSal_05
m/z (full)
Intensity
m/z (zoom)
Slide 26 of 26
"Office Theme"
English (U.S.)
102%
Start
I:\Halobacterium\1st+2n...
Raw Viewer - [Dataset1]
Result Explorer
Microsoft PowerPoint - [...
DE
18:41

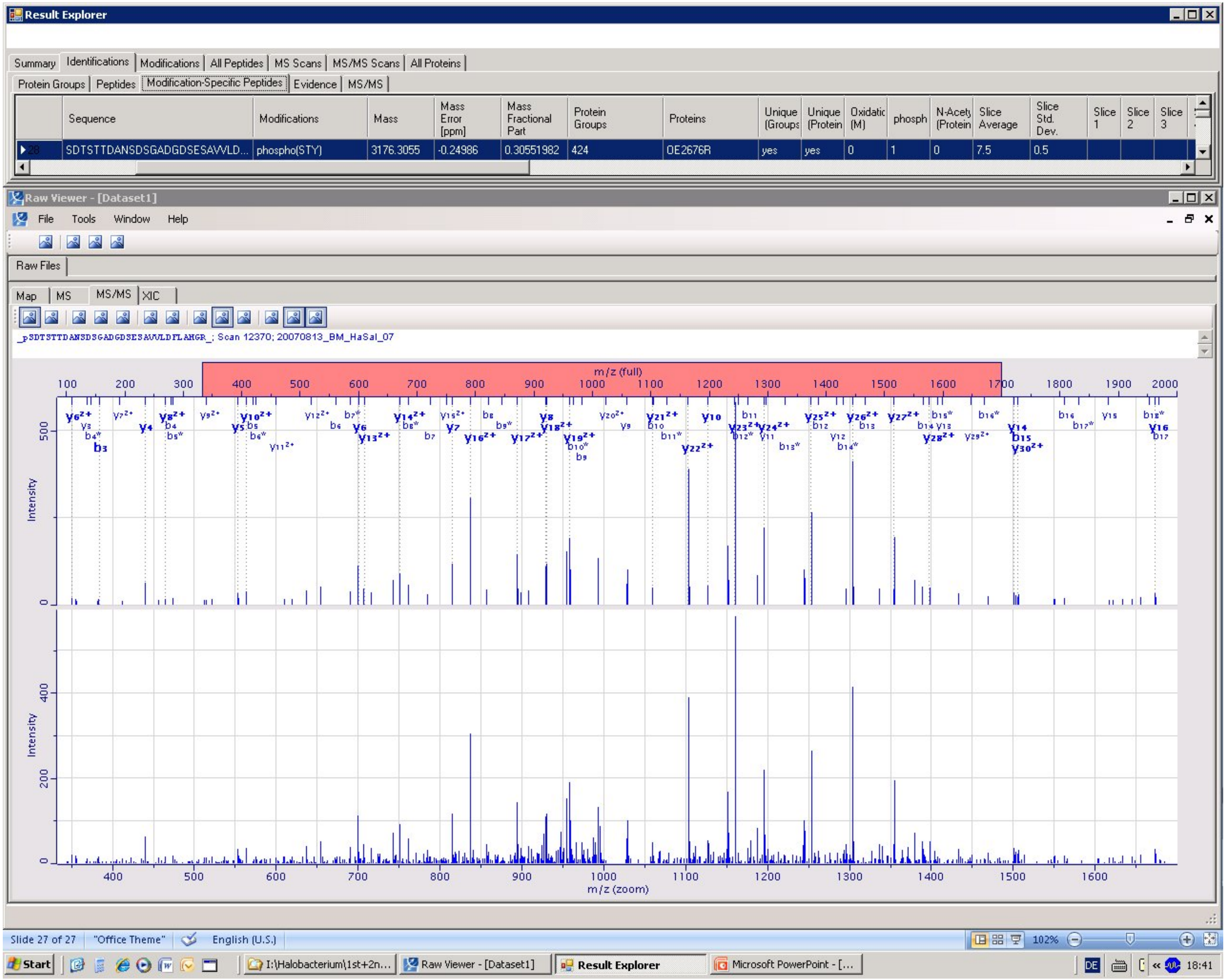
Result Explorer
Summary
Identifications
Modifications
All Peptides
MS Scans
MS/MS Scans
All Proteins
Protein Groups
Peptides
Modification-Specific Peptides
Evidence
MS/MS
Sequence
Modifications
Mass
Mass Error [ppm]
Mass Fractional Part
Protein Groups
Proteins
SDTSTTDANSDSGADGDSESAVVLD...
phospho(STY)
3176.3055
-0.24986
0.30551982
424
OE2676R
Raw Viewer - [Dataset1]
File
Tools
Window
Help
Raw Files
Map
MS
MS/MS
XIC
pSDTSTTDANSDSGADGDSESAVVLDFLAHGR; Scan 12370; 20070813_BM_HaSal_07
m/z (full)
Intensity
m/z (zoom)
Slide 27 of 27
"Office Theme"
English (U.S.)
Start
Raw Viewer - [Dataset1]
Result Explorer
Microsoft PowerPoint - [...
18:41

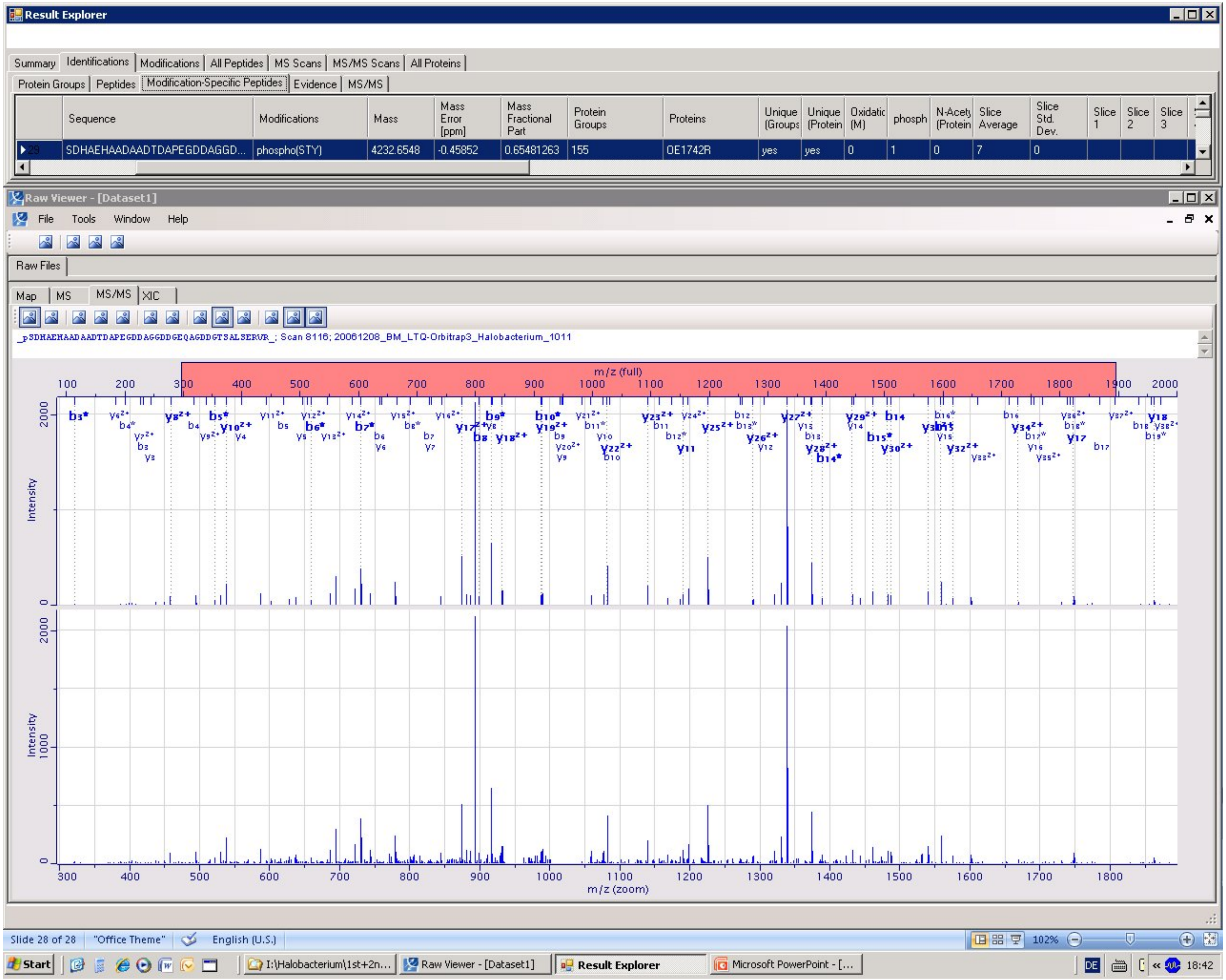
Result Explorer
Summary
Identifications
Modifications
All Peptides
MS Scans
MS/MS Scans
All Proteins
Protein Groups
Peptides
Modification-Specific Peptides
Evidence
MS/MS
Sequence
Modifications
Mass
Mass Error [ppm]
Mass Fractional Part
Protein Groups
Proteins
Unique (Groups
Unique (Protein
Oxidatic (M)
phosph
N-Acety (Protein
Slice Average
Slice Std. Dev.
Slice 1
Slice 2
Slice 3
SDHAEHAADAADTDAPEGDDAGGD...
phospho(STY)
4232.6548
-0.45852
0.65481263
155
OE1742R
yes
yes
0
1
0
7
0
Raw Viewer - [Dataset1]
File
Tools
Window
Help
Raw Files
Map
MS
MS/MS
XIC
pSDHAEHAADAADTDAPEGDDAGGDDGEQAGDDGTSALSERVR; Scan 8116; 20061208_BM_LTQ-Orbitrap3_Halobacterium_1011
m/z (full)
Intensity
m/z (zoom)
Slide 28 of 28
"Office Theme"
English (U.S.)
102%
Start
I:\Halobacterium\1st+2n...
Raw Viewer - [Dataset1]
Result Explorer
Microsoft PowerPoint - [...
DE
18:42

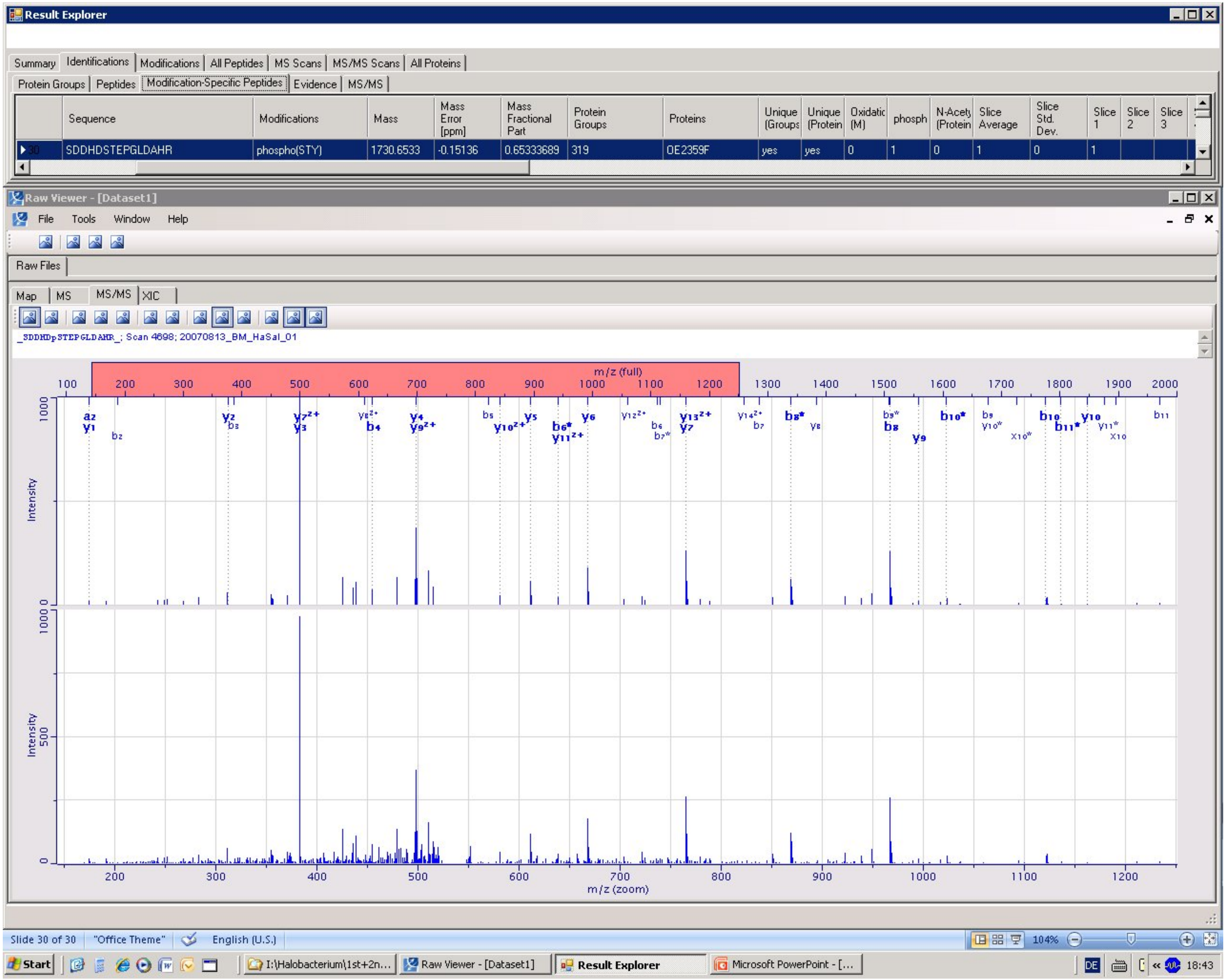
Result Explorer
Summary
Identifications
Modifications
All Peptides
MS Scans
MS/MS Scans
All Proteins
Protein Groups
Peptides
Modification-Specific Peptides
Evidence
MS/MS
Sequence
Modifications
Mass
Mass Error [ppm]
Mass Fractional Part
Protein Groups
Proteins
Unique (Groups
Unique (Protein
Oxidatic (M)
phosph
N-Acety (Protein
Slice Average
Slice Std. Dev.
Slice 1
Slice 2
Slice 3
SDDHDSTEPGLDAHR
phospho(STY)
1730.6533
-0.15136
0.65333689
319
OE2359F
yes
yes
Raw Viewer - [Dataset1]
File
Tools
Window
Help
Raw Files
Map
MS
MS/MS
XIC
SDDHDpSTEPGLDAHR; Scan 4698; 20070813_BM_HaSal_01
m/z (full)
Intensity
m/z (zoom)
Slide 30 of 30
"Office Theme"
English (U.S.)
104%
Start
I:\Halobacterium\1st+2n...
Raw Viewer - [Dataset1]
Result Explorer
Microsoft PowerPoint - [...
18:43

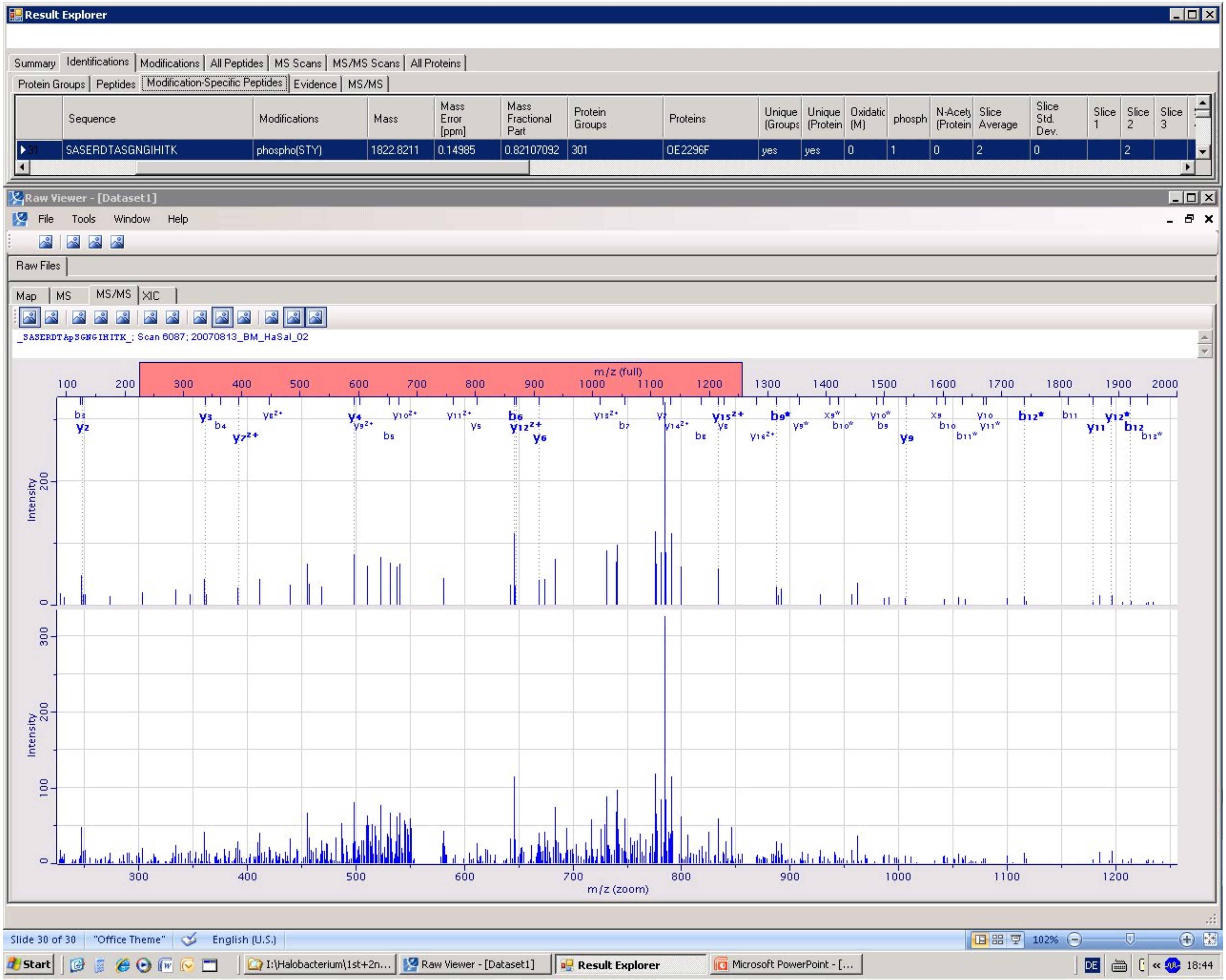
Result Explorer
Summary | Identifications | Modifications | All Peptides | MS Scans | MS/MS Scans | All Proteins
Protein Groups | Peptides | Modification-Specific Peptides | Evidence | MS/MS
Sequence | Modifications | Mass | Mass Error [ppm] | Mass Fractional Part | Protein Groups | Proteins | Unique (Groups | Unique (Protein | Oxidatic (M) | phosph | N-Acety (Protein | Slice Average | Slice Std. Dev. | Slice 1 | Slice 2 | Slice 3
SASERDTASGNGIHITK | phospho(STY) | 1822.8211 | 0.14985 | 0.82107092 | 301 | OE2296F | yes | yes | 0 | 1 | 0 | 2 | 0 | | 2
Raw Viewer - [Dataset1]
File Tools Window Help
Raw Files
Map MS MS/MS XIC
SASERDTApSGNGIHITK; Scan 6087; 20070813_BM_HaSal_02
m/z (full)
Intensity
m/z (zoom)
Slide 30 of 30 "Office Theme" English (U.S.)
Start
Raw Viewer - [Dataset1]
Result Explorer
Microsoft PowerPoint - [...
18:44

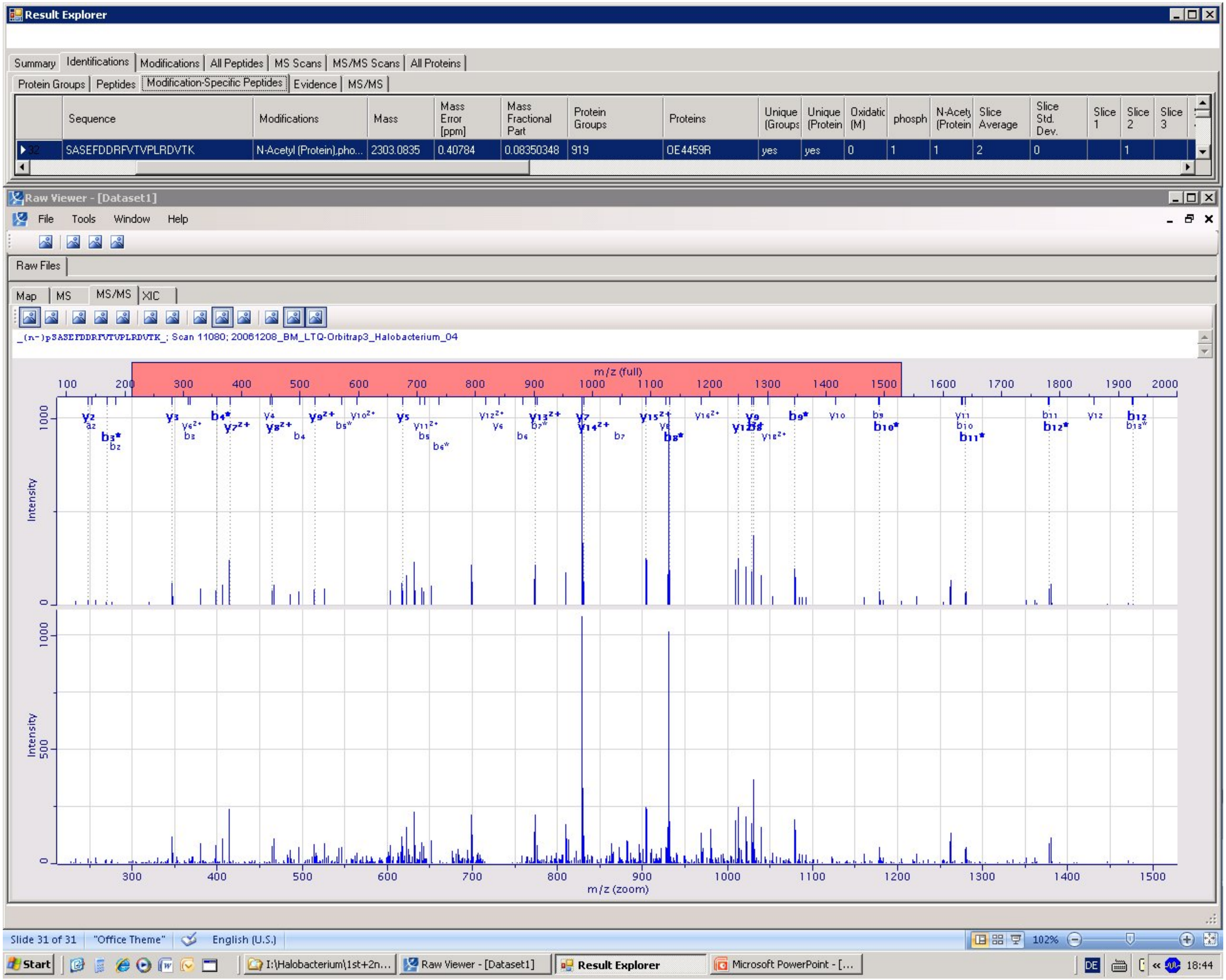
Result Explorer
Summary | Identifications | Modifications | All Peptides | MS Scans | MS/MS Scans | All Proteins
Protein Groups | Peptides | Modification-Specific Peptides | Evidence | MS/MS
Sequence | Modifications | Mass | Mass Error [ppm] | Mass Fractional Part | Protein Groups | Proteins | Unique (Groups | Unique (Protein | Oxidatic (M) | phosph | N-Acety (Protein | Slice Average | Slice Std. Dev. | Slice 1 | Slice 2 | Slice 3
SASEFDDRFVTVPLRDVTK | N-Acetyl (Protein),pho... | 2303.0835 | 0.40784 | 0.08350348 | 919 | OE4459R | yes | yes | 0 | 1 | 1 | 2 | 0 | | 1
Raw Viewer - [Dataset1]
File Tools Window Help
Raw Files
Map MS MS/MS XIC
(n-)pSASEFDDRFVTVPLRDVTK; Scan 11080; 20061208_BM_LTQ-Orbitrap3_Halobacterium_04
m/z (full)
Intensity
m/z (zoom)
Slide 31 of 31 "Office Theme" English (U.S.)
Start
I:\Halobacterium\1st+2n...
Raw Viewer - [Dataset1]
Result Explorer
Microsoft PowerPoint - [...
18:44

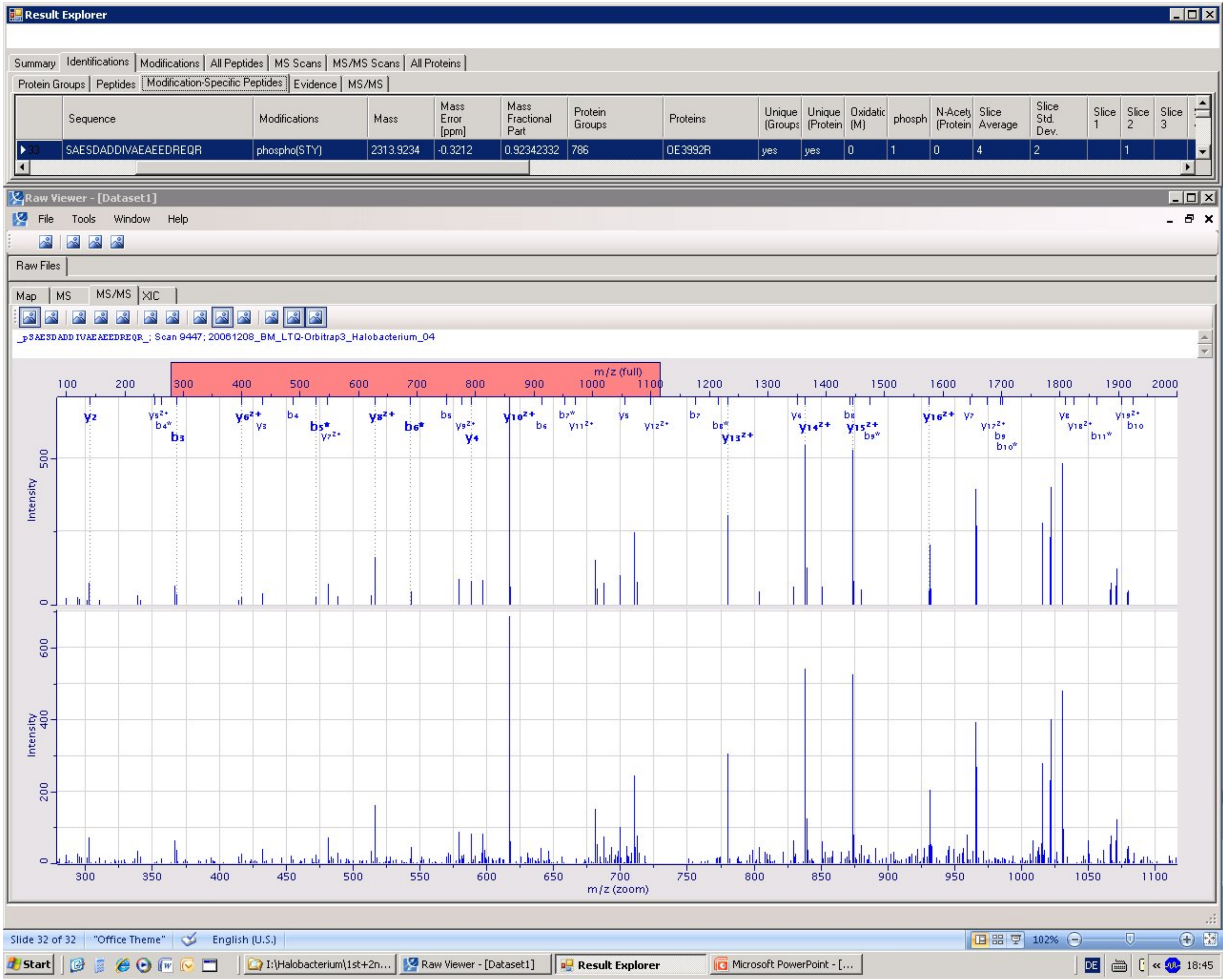

Result Explorer
Summary | Identifications | Modifications | All Peptides | MS Scans | MS/MS Scans | All Proteins
Protein Groups | Peptides | Modification-Specific Peptides | Evidence | MS/MS
Sequence | Modifications | Mass | Mass Error [ppm] | Mass Fractional Part | Protein Groups | Proteins | Unique (Groups | Unique (Protein | Oxidatic (M) | phosph | N-Acety (Protein | Slice Average | Slice Std. Dev. | Slice 1 | Slice 2 | Slice 3
SAESDADDIVAEAEEDREQR | phospho(STY) | 2313.9234 | -0.3212 | 0.92342332 | 786 | OE3992R | yes | yes | 0 | 1 | 0 | 4 | 2 | | 1
Raw Viewer - [Dataset1]
File Tools Window Help
Raw Files
Map MS MS/MS XIC
pSAESDADDIVAEAEEDREQR; Scan 9447; 20061208_BM_LTQ-Orbitrap3_Halobacterium_04
m/z (full)
Intensity
m/z (zoom)
Slide 32 of 32 "Office Theme" English (U.S.) 102%
Start I:\Halobacterium\1st+2n... Raw Viewer - [Dataset1] Result Explorer Microsoft PowerPoint - [...
DE 18:45

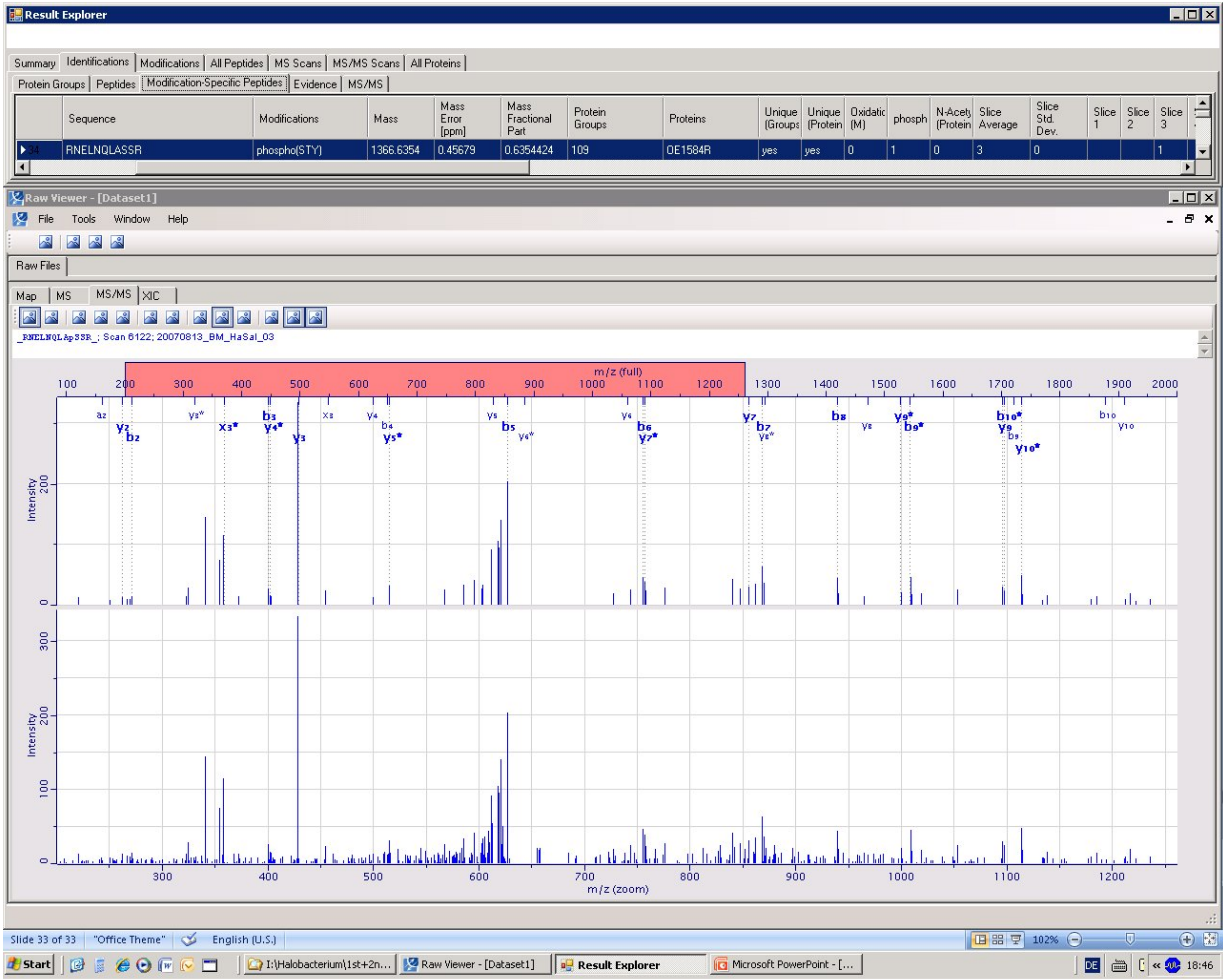
Result Explorer
Summary
Identifications
Modifications
All Peptides
MS Scans
MS/MS Scans
All Proteins
Protein Groups
Peptides
Modification-Specific Peptides
Evidence
MS/MS
Sequence
RNELNQLASSR
phospho(STY)
1366.6354
Raw Viewer - [Dataset1]
RNELNQLApSSR; Scan 6122; 20070813_BM_HaSal_03
m/z (full)
Intensity
m/z (zoom)

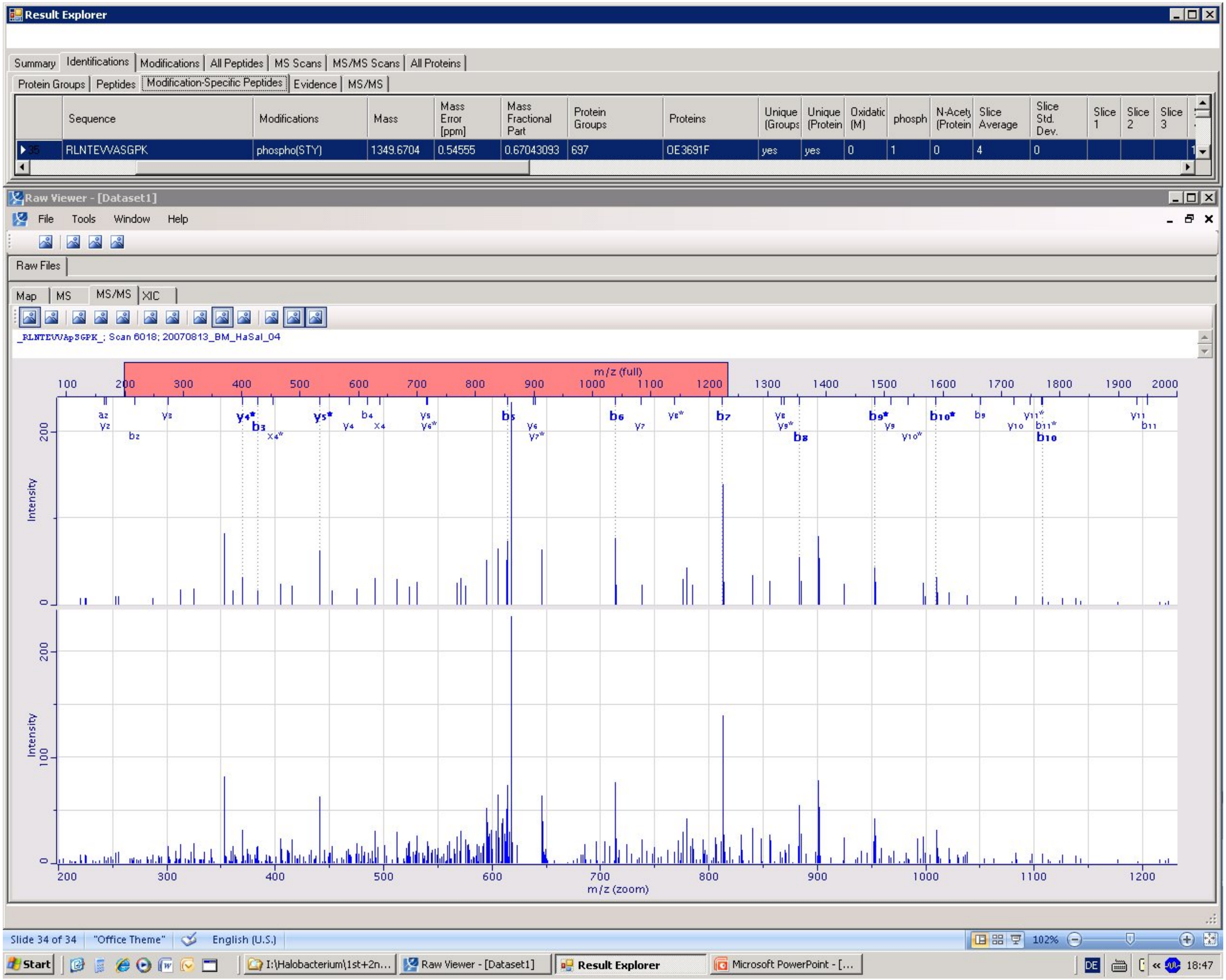
Result Explorer
Summary
Identifications
Modifications
All Peptides
MS Scans
MS/MS Scans
All Proteins
Protein Groups
Peptides
Modification-Specific Peptides
Evidence
MS/MS
Sequence
Modifications
Mass
Mass Error [ppm]
Mass Fractional Part
Protein Groups
Proteins
Unique (Groups
Unique (Protein
Oxidatic (M)
phosph
N-Acety (Protein
Slice Average
Slice Std. Dev.
Slice 1
Slice 2
Slice 3
RLNTEVVASGPK
phospho(STY)
1349.6704
0.54555
0.67043093
697
OE3691F
yes
yes
0
1
0
4
0
Raw Viewer - [Dataset1]
File
Tools
Window
Help
Raw Files
Map
MS
MS/MS
XIC
RLNTEVVApSGPK; Scan 6018; 20070813_BM_HaSal_04
m/z (full)
Intensity
m/z (zoom)
Slide 34 of 34
"Office Theme"
English (U.S.)
102%
Start
I:\Halobacterium\1st+2n...
Raw Viewer - [Dataset1]
Result Explorer
Microsoft PowerPoint - [...
18:47

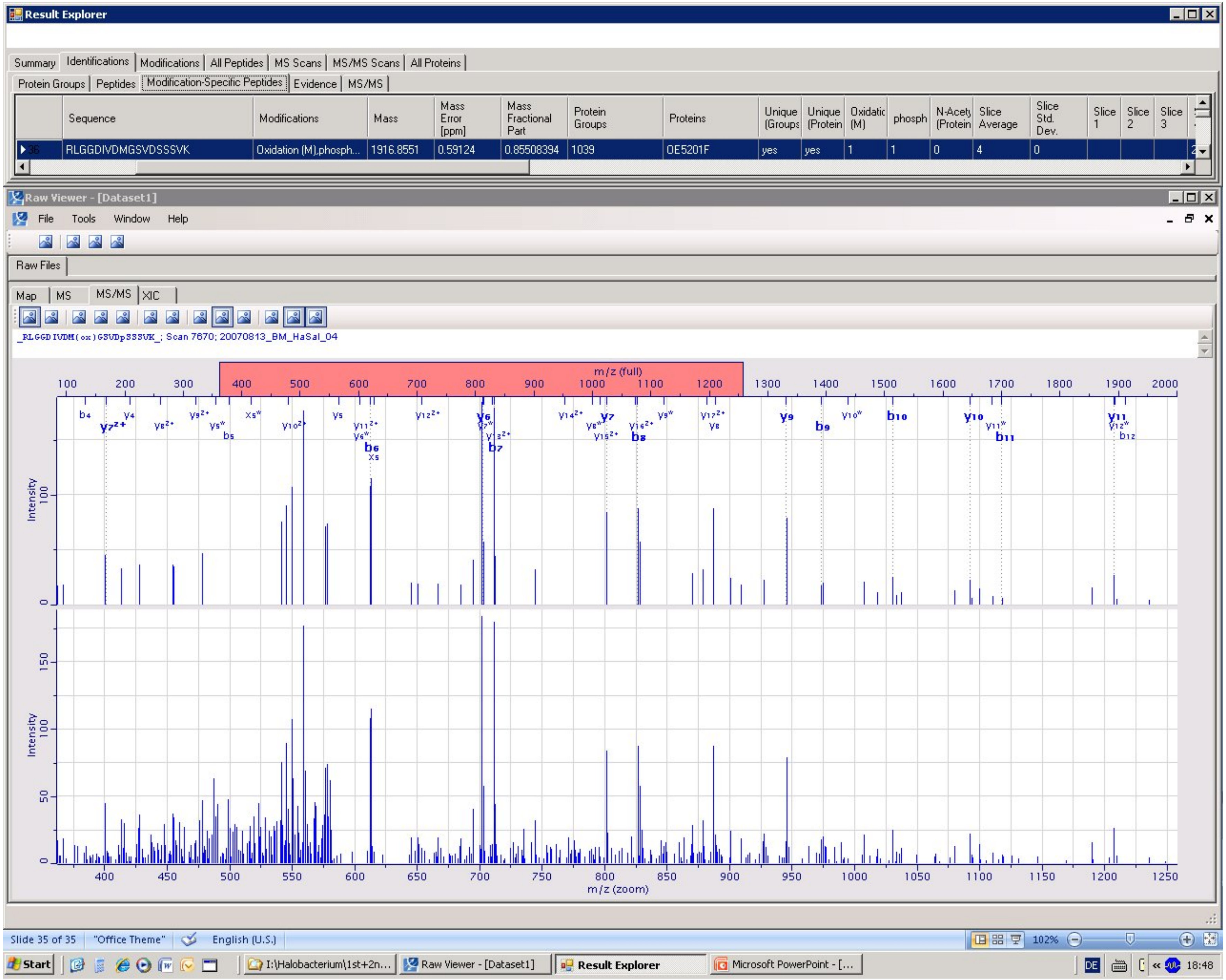
Result Explorer
Summary | Identifications | Modifications | All Peptides | MS Scans | MS/MS Scans | All Proteins
Protein Groups | Peptides | Modification-Specific Peptides | Evidence | MS/MS
Sequence | Modifications | Mass | Mass Error [ppm] | Mass Fractional Part | Protein Groups | Proteins | Unique (Groups | Unique (Protein | Oxidatic (M) | phosph | N-Acety (Protein | Slice Average | Slice Std. Dev. | Slice 1 | Slice 2 | Slice 3
RLGGDIVDMGSVDSSSVK | Oxidation (M),phosph... | 1916.8551 | 0.59124 | 0.85508394 | 1039 | OE5201F | yes | yes | 1 | 1 | 0 | 4 | 0
Raw Viewer - [Dataset1]
File Tools Window Help
Raw Files
Map | MS | MS/MS | XIC
RLGGDIVDM(ox)GSVDpSSSVK; Scan 7670; 20070813_BM_HaSal_04
m/z (full)
Intensity
m/z (zoom)
Slide 35 of 35 "Office Theme" English (U.S.) 102%
Start I:\Halobacterium\1st+2n... Raw Viewer - [Dataset1] Result Explorer Microsoft PowerPoint - [... DE 18:48

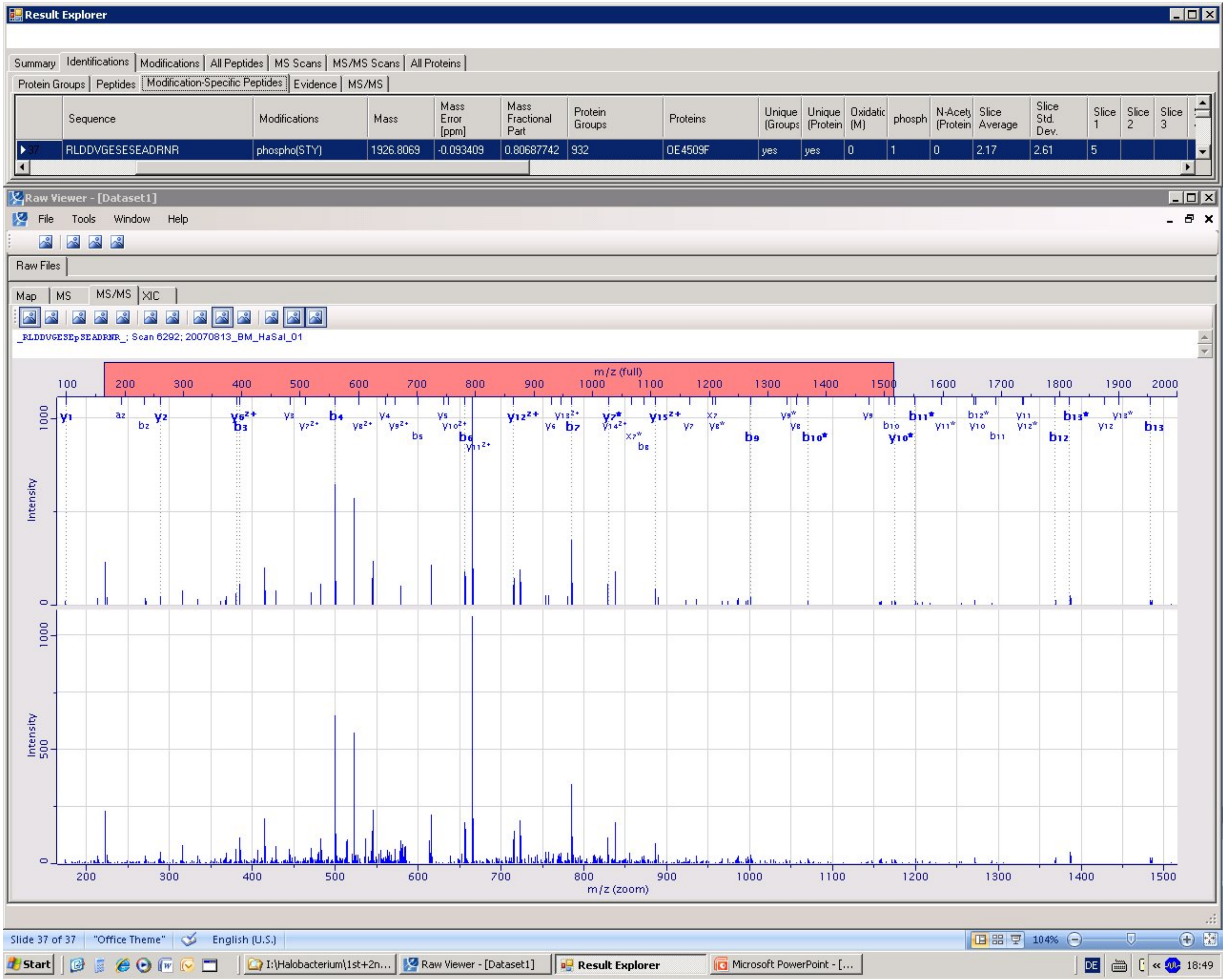
Result Explorer
Summary | Identifications | Modifications | All Peptides | MS Scans | MS/MS Scans | All Proteins
Protein Groups | Peptides | Modification-Specific Peptides | Evidence | MS/MS
Sequence | Modifications | Mass | Mass Error [ppm] | Mass Fractional Part | Protein Groups | Proteins | Unique (Groups) | Unique (Protein) | Oxidation (M) | phosph | N-Acetyl (Protein) | Slice Average | Slice Std. Dev. | Slice 1 | Slice 2 | Slice 3
RLDDVGESESEADRNR | phospho(STY) | 1926.8069 | -0.093409 | 0.80687742 | 932 | OE4509F | yes | yes | 0 | 1 | 0 | 2.17 | 2.61 | 5
Raw Viewer - [Dataset1]
File Tools Window Help
Raw Files
Map | MS | MS/MS | XIC
RLDDVGESEpSEADRNR; Scan 6292; 20070813_BM_HaSal_01
m/z (full)
Intensity
m/z (zoom)
Slide 37 of 37 "Office Theme" English (U.S.)
Start
I:\Halobacterium\1st+2n...
Raw Viewer - [Dataset1]
Result Explorer
Microsoft PowerPoint - [...
18:49

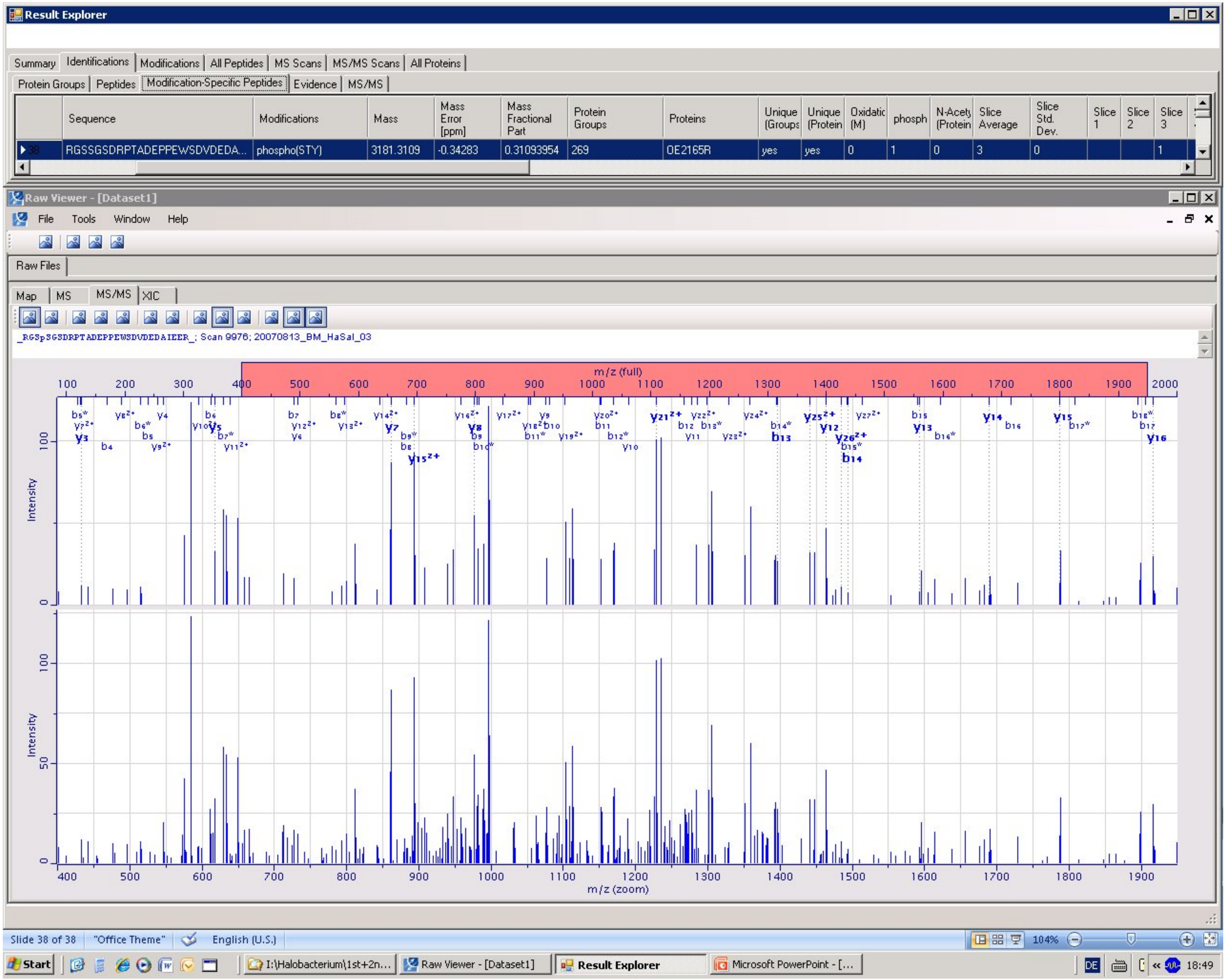
Result Explorer
Summary
Identifications
Modifications
All Peptides
MS Scans
MS/MS Scans
All Proteins
Protein Groups
Peptides
Modification-Specific Peptides
Evidence
MS/MS
Sequence
Modifications
Mass
Mass Error [ppm]
Mass Fractional Part
Protein Groups
Proteins
Unique (Groups
Unique (Protein
Oxidatic (M)
phosph
N-Acety (Protein
Slice Average
Slice Std. Dev.
Slice 1
Slice 2
Slice 3
RGSSGSDRPTADEPPEWSDVDEDA...
phospho(STY)
3181.3109
-0.34283
0.31093954
269
OE2165R
yes
yes
0
1
0
3
0
1
Raw Viewer - [Dataset1]
File
Tools
Window
Help
Raw Files
Map
MS
MS/MS
XIC
RGSpSGSDRPTADEPPEWSDVDEDAIEER; Scan 9976; 20070813_BM_HaSal_03
m/z (full)
Intensity
m/z (zoom)
Slide 38 of 38
"Office Theme"
English (U.S.)
104%
Start
I:\Halobacterium\1st+2n...
Raw Viewer - [Dataset1]
Result Explorer
Microsoft PowerPoint - [...
18:49

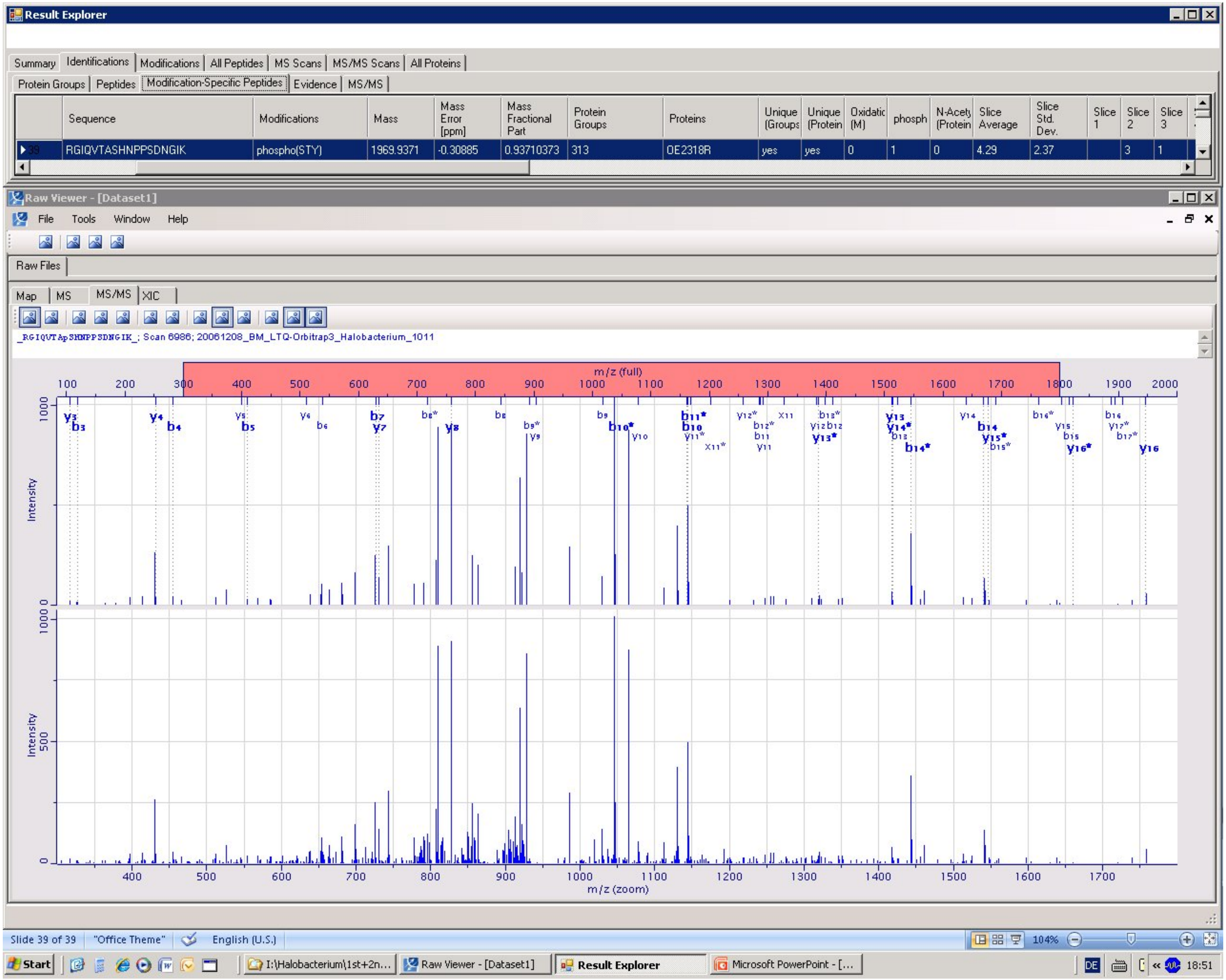
Result Explorer
Summary | Identifications | Modifications | All Peptides | MS Scans | MS/MS Scans | All Proteins
Protein Groups | Peptides | Modification-Specific Peptides | Evidence | MS/MS
Sequence | Modifications | Mass | Mass Error [ppm] | Mass Fractional Part | Protein Groups | Proteins | Unique (Groups | Unique (Protein | Oxidatic (M) | phosph | N-Acety (Protein | Slice Average | Slice Std. Dev. | Slice 1 | Slice 2 | Slice 3
RGIQVTASHNPPSDNGIK | phospho(STY) | 1969.9371 | -0.30885 | 0.93710373 | 313 | OE2318R | yes | yes | 0 | 1 | 0 | 4.29 | 2.37 | | 3 | 1
Raw Viewer - [Dataset1]
File Tools Window Help
Raw Files
Map | MS | MS/MS | XIC
RGIQVTApSHNPPSDNGIK; Scan 6986; 20061208_BM_LTQ-Orbitrap3_Halobacterium_1011
m/z (full)
Intensity
m/z (zoom)
Slide 39 of 39 "Office Theme" English (U.S.)
Start | I:\Halobacterium\1st+2n... | Raw Viewer - [Dataset1] | Result Explorer | Microsoft PowerPoint - [...
18:51

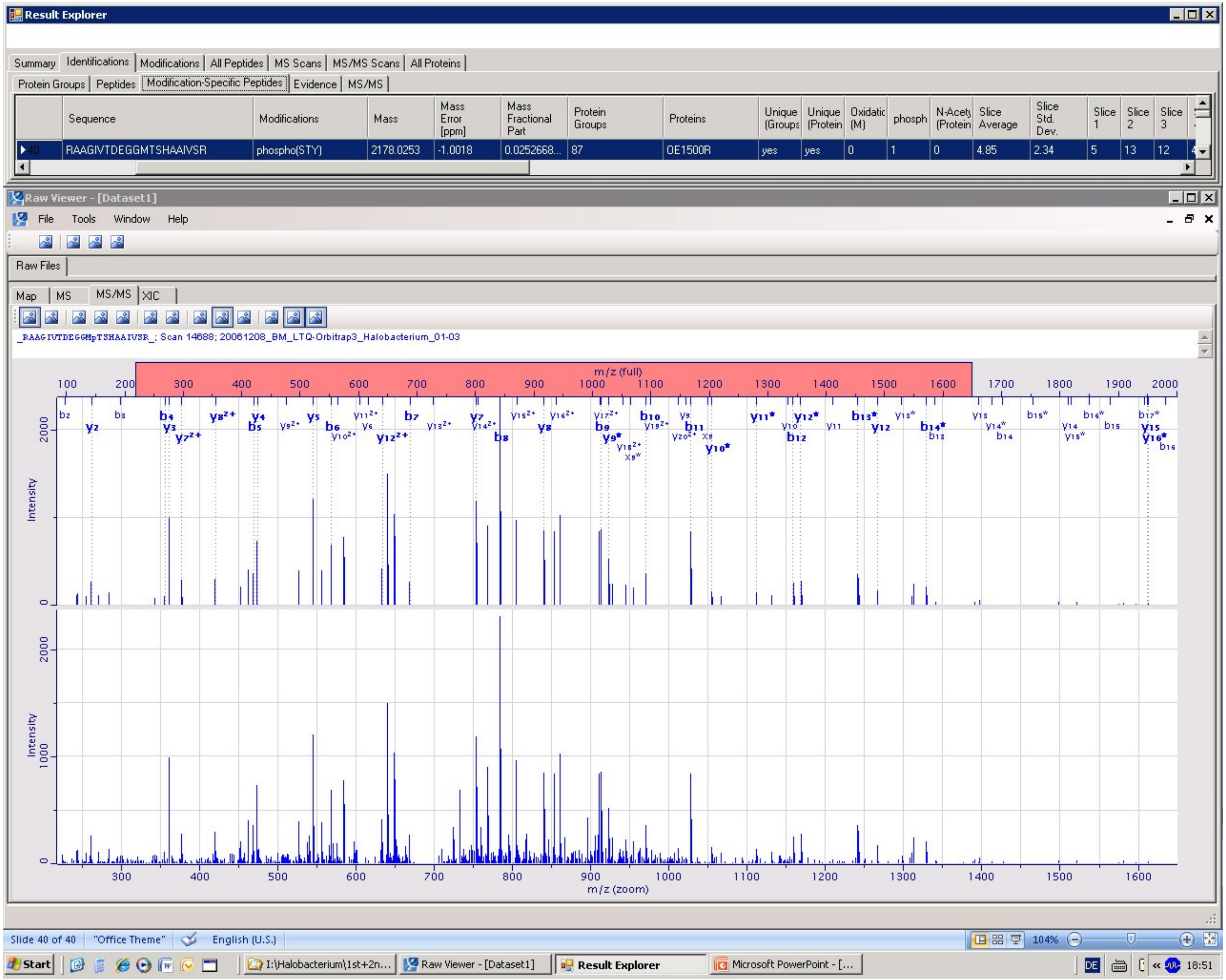
Result Explorer
Summary
Identifications
Modifications
All Peptides
MS Scans
MS/MS Scans
All Proteins
Protein Groups
Peptides
Modification-Specific Peptides
Evidence
MS/MS
Sequence
Modifications
Mass
Mass Error [ppm]
Mass Fractional Part
Protein Groups
Proteins
Slice Average
Slice Std. Dev.
RAAGIVTDEGGMTSHAAIVSR
phospho(STY)
2178.0253
-1.0018
87
OE1500R
yes
4.85
2.34
Raw Viewer - [Dataset1]
File
Tools
Window
Help
Raw Files
Map
MS
MS/MS
XIC
RAAGIVTDEGGMpTSHAAIVSR; Scan 14688; 20061208_BM_LTQ-Orbitrap3_Halobacterium_01-03
m/z (full)
Intensity
m/z (zoom)
Slide 40 of 40
"Office Theme"
English (U.S.)
104%
Start
I:\Halobacterium\1st+2n...
Raw Viewer - [Dataset1]
Result Explorer
Microsoft PowerPoint - [...
18:51

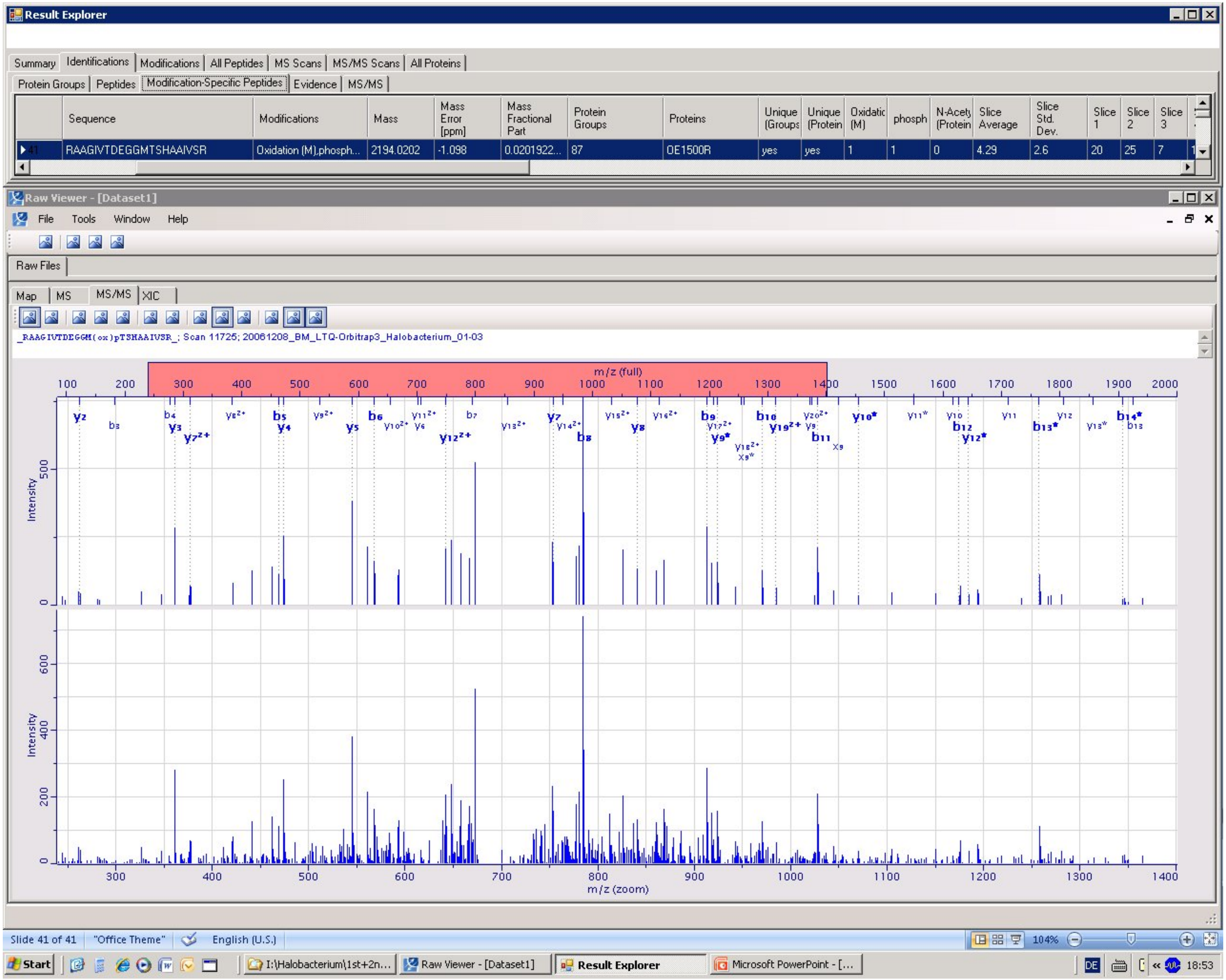
Result Explorer
Summary | Identifications | Modifications | All Peptides | MS Scans | MS/MS Scans | All Proteins
Protein Groups | Peptides | Modification-Specific Peptides | Evidence | MS/MS
Sequence | Modifications | Mass | Mass Error [ppm] | Mass Fractional Part | Protein Groups | Proteins | Unique (Groups | Unique (Protein | Oxidatic (M) | phosph | N-Acety (Protein | Slice Average | Slice Std. Dev. | Slice 1 | Slice 2 | Slice 3
RAAGIVTDEGGMTSHAAIVSR | Oxidation (M),phosph... | 2194.0202 | -1.098 | 0.0201922... | 87 | OE1500R | yes | yes | 1 | 1 | 0 | 4.29 | 2.6 | 20 | 25 | 7
Raw Viewer - [Dataset1]
File Tools Window Help
Raw Files
Map MS MS/MS XIC
RAAGIVTDEGGM(ox)pTSHAAIVSR; Scan 11725; 20061208_BM_LTQ-Orbitrap3_Halobacterium_01-03
m/z (full)
Intensity
m/z (zoom)
Slide 41 of 41 "Office Theme" English (U.S.)
Start
I:\Halobacterium\1st+2n...
Raw Viewer - [Dataset1]
Result Explorer
Microsoft PowerPoint - [...
18:53

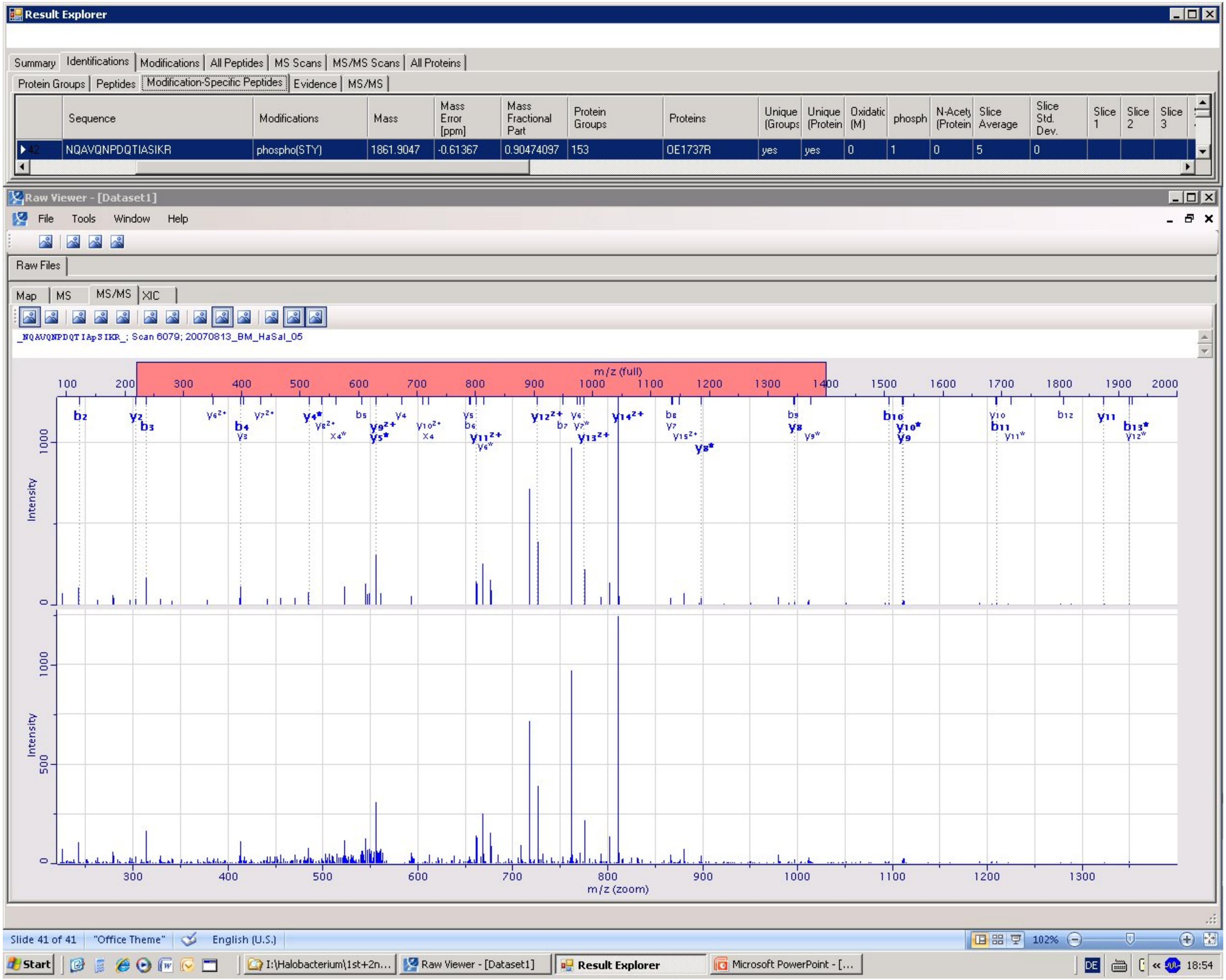
Result Explorer
Summary
Identifications
Modifications
All Peptides
MS Scans
MS/MS Scans
All Proteins
Protein Groups
Peptides
Modification-Specific Peptides
Evidence
MS/MS
Sequence
Modifications
Mass
Mass Error [ppm]
Mass Fractional Part
Protein Groups
Proteins
NQAVQNPDQTIASIKR
phospho(STY)
1861.9047
-0.61367
0.90474097
153
OE1737R
Raw Viewer - [Dataset1]
File
Tools
Window
Help
Raw Files
Map
MS
MS/MS
XIC
NQAVQNPDQTIApSIKR; Scan 6079; 20070813_BM_HaSal_05
m/z (full)
Intensity
m/z (zoom)
Slide 41 of 41
"Office Theme"
English (U.S.)
Start
Raw Viewer - [Dataset1]
Result Explorer
18:54

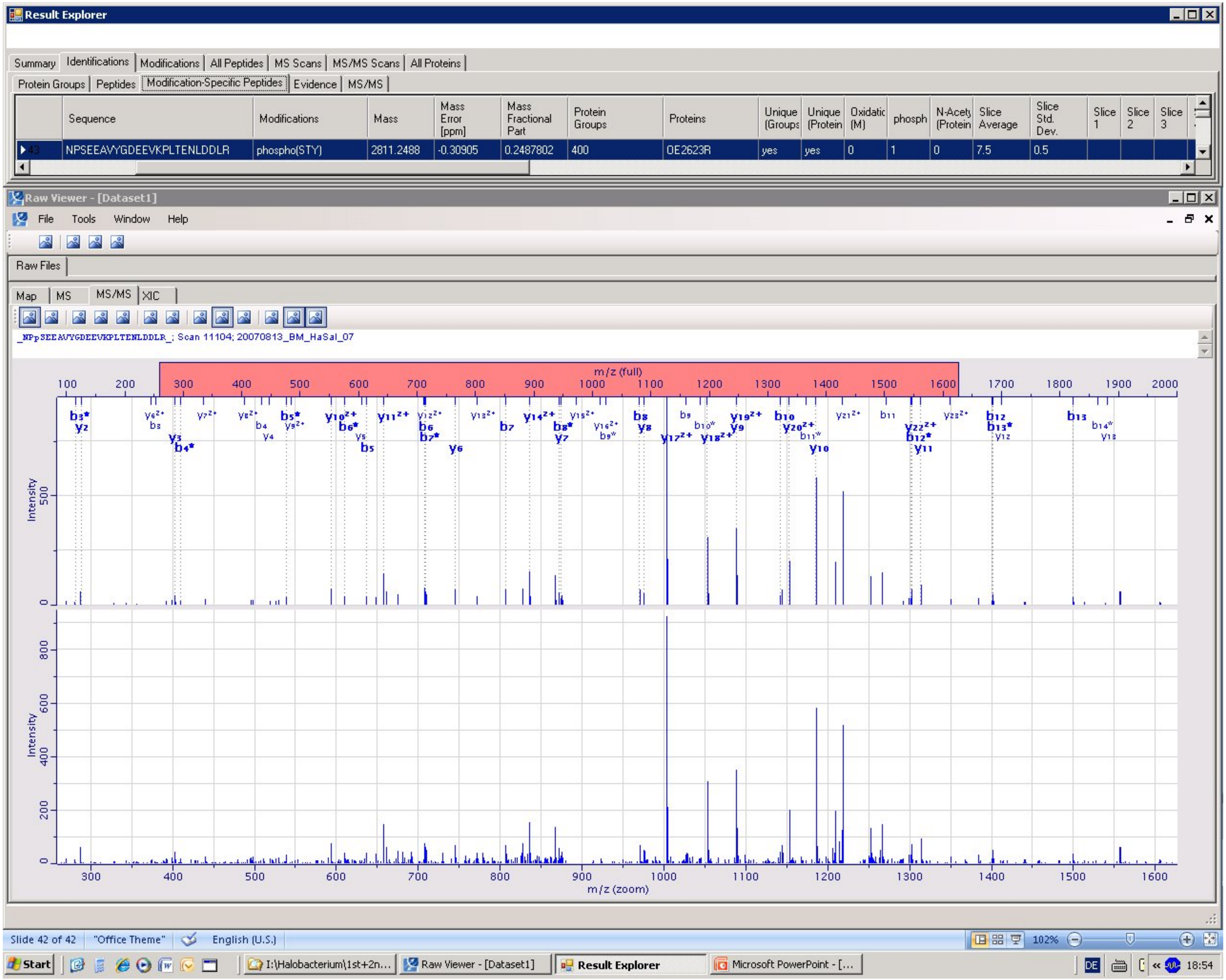
Result Explorer
Summary
Identifications
Modifications
All Peptides
MS Scans
MS/MS Scans
All Proteins
Protein Groups
Peptides
Modification-Specific Peptides
Evidence
MS/MS
Sequence
Modifications
Mass
Mass Error [ppm]
Mass Fractional Part
Protein Groups
Proteins
Unique (Groups
Unique (Protein
Oxidatic (M)
phosph
N-Acety (Protein
Slice Average
Slice Std. Dev.
Slice 1
Slice 2
Slice 3
NPSEEAVYGDEEVKPLTENLDDLR
phospho(STY)
2811.2488
-0.30905
0.2487802
400
OE2623R
yes
yes
0
1
0
7.5
0.5
Raw Viewer - [Dataset1]
File
Tools
Window
Help
Raw Files
Map
MS
MS/MS
XIC
NPpSEEAVYGDEEVKPLTENLDDLR; Scan 11104; 20070813_BM_HaSal_07
m/z (full)
Intensity
m/z (zoom)
Slide 42 of 42
"Office Theme"
English (U.S.)
102%
Start
I:\Halobacterium\1st+2n...
Raw Viewer - [Dataset1]
Result Explorer
Microsoft PowerPoint - [...
DE
18:54

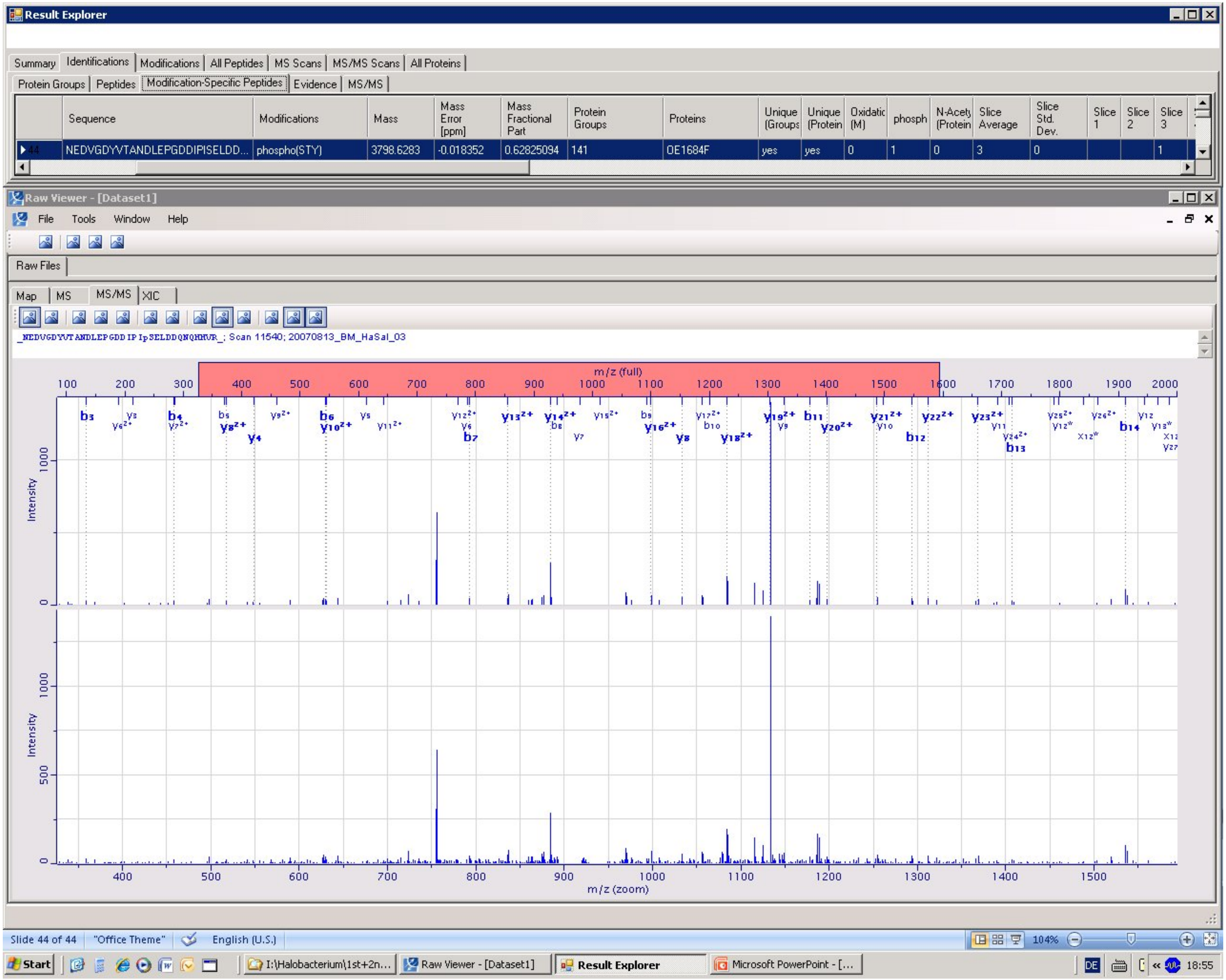
Result Explorer
Summary
Identifications
Modifications
All Peptides
MS Scans
MS/MS Scans
All Proteins
Protein Groups
Peptides
Modification-Specific Peptides
Evidence
MS/MS
Sequence
Modifications
Mass
Mass Error [ppm]
Mass Fractional Part
Protein Groups
Proteins
Slice Average
Slice Std. Dev.
Slice 1
Slice 2
Slice 3
NEDVGDYVTANDLEPGDDIPISELDD...
phospho(STY)
3798.6283
-0.018352
0.62825094
141
OE1684F
yes
yes
Raw Viewer - [Dataset1]
File
Tools
Window
Help
Raw Files
Map
MS
MS/MS
XIC
m/z (full)
Intensity
m/z (zoom)
Slide 44 of 44
"Office Theme"
English (U.S.)
Start
Raw Viewer - [Dataset1]
Result Explorer
18:55

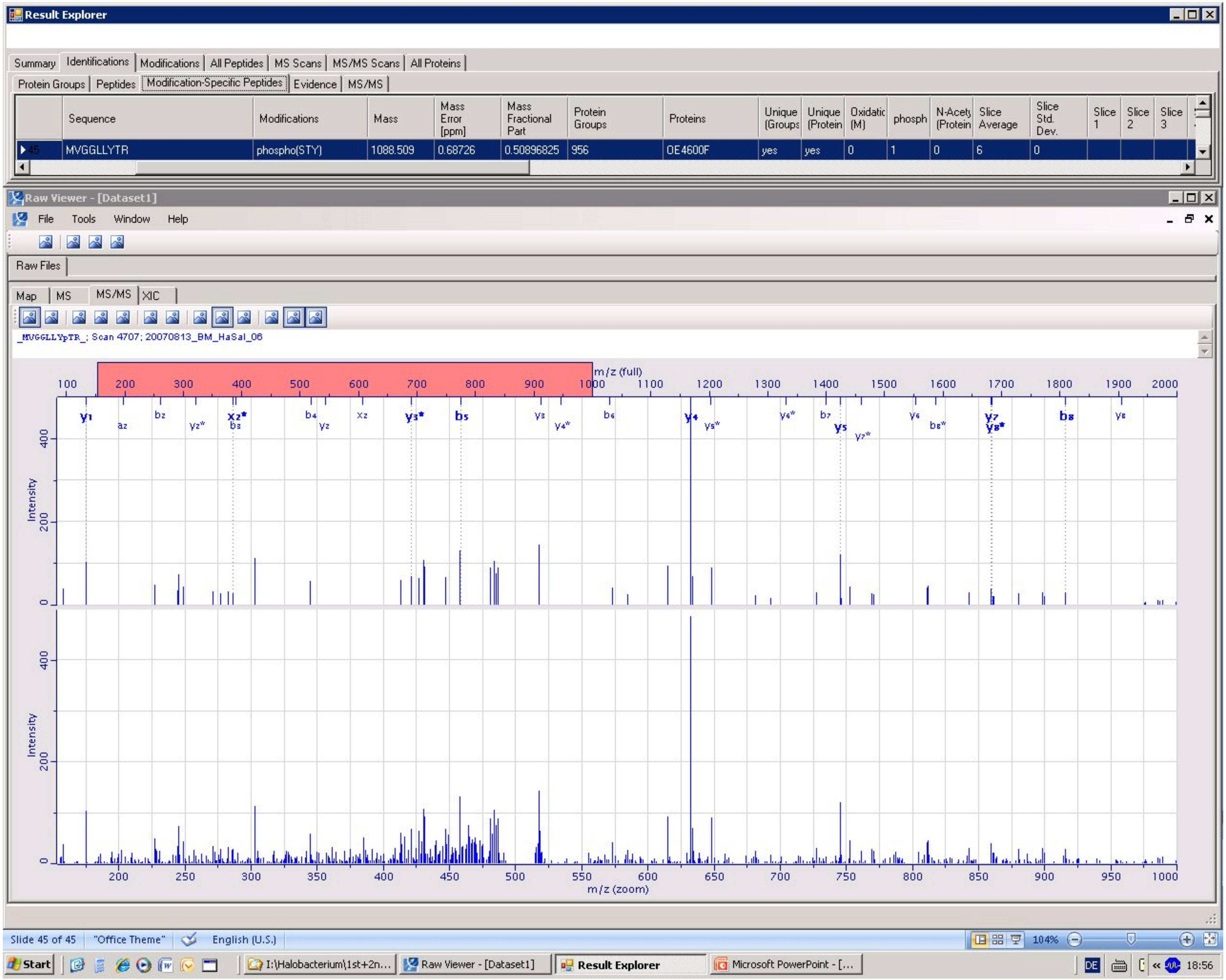
Result Explorer
Summary
Identifications
Modifications
All Peptides
MS Scans
MS/MS Scans
All Proteins
Protein Groups
Peptides
Modification-Specific Peptides
Evidence
MS/MS
Sequence
Modifications
Mass
Mass Error [ppm]
Mass Fractional Part
Protein Groups
Proteins
Unique (Groups
Unique (Protein
Oxidatio (M)
phosph
N-Acety (Protein
Slice Average
Slice Std. Dev.
Slice 1
Slice 2
Slice 3
MVGGLLYTR
phospho(STY)
1088.509
0.68726
0.50896825
956
OE4600F
yes
yes
0
1
0
6
0
Raw Viewer - [Dataset1]
File
Tools
Window
Help
Raw Files
Map
MS
MS/MS
XIC
MVGGLLYpTR; Scan 4707; 20070813_BM_HaSal_06
m/z (full)
Intensity
m/z (zoom)
Slide 45 of 45
"Office Theme"
English (U.S.)
104%
Start
I:\Halobacterium\1st+2n...
Raw Viewer - [Dataset1]
Result Explorer
Microsoft PowerPoint - [...
DE
18:56

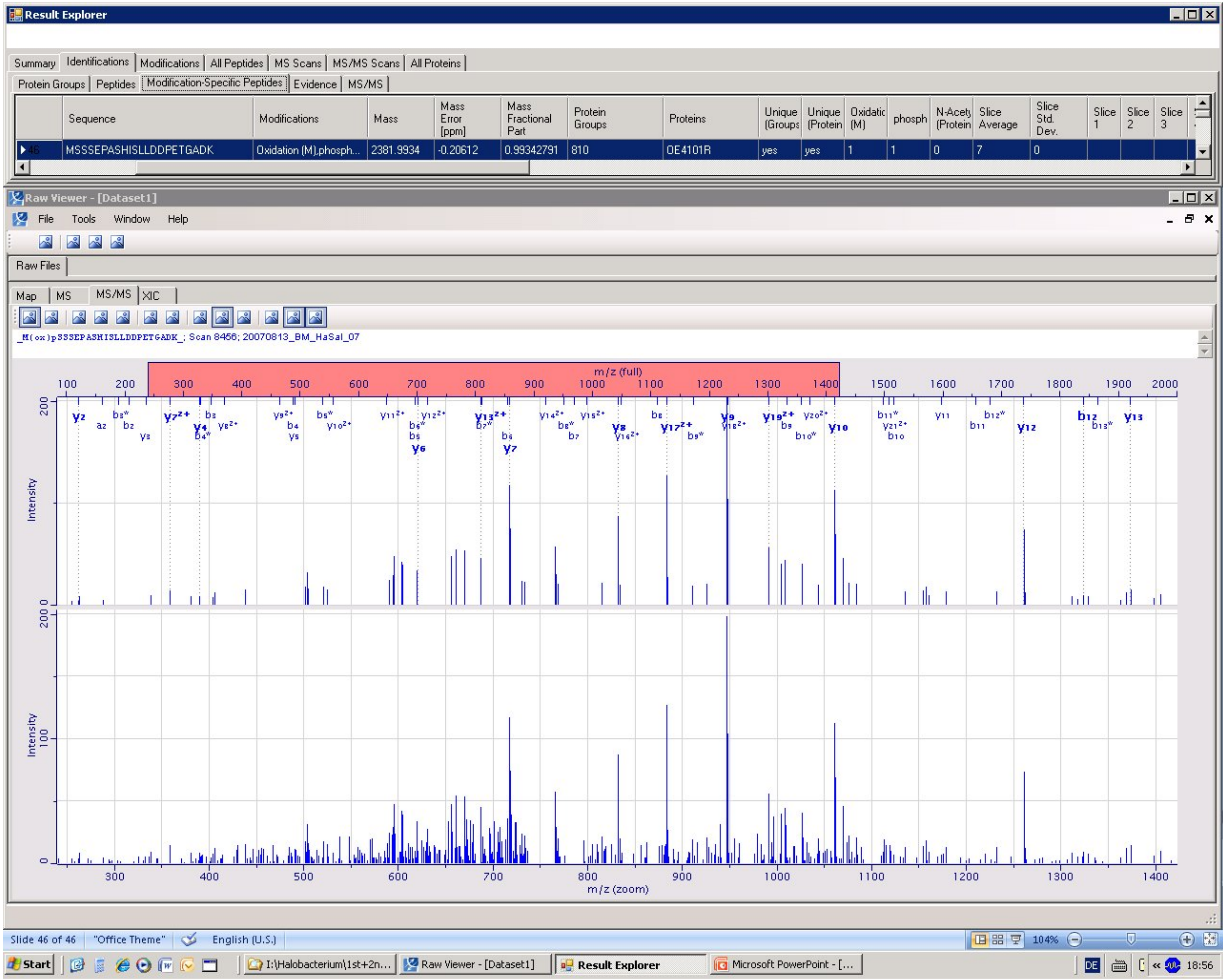
Result Explorer
Summary
Identifications
Modifications
All Peptides
MS Scans
MS/MS Scans
All Proteins
Protein Groups
Peptides
Modification-Specific Peptides
Evidence
MS/MS
Sequence | Modifications | Mass | Mass Error [ppm] | Mass Fractional Part | Protein Groups | Proteins | Unique (Groups) | Unique (Protein) | Oxidation (M) | phosph | N-Acety (Protein) | Slice Average | Slice Std. Dev. | Slice 1 | Slice 2 | Slice 3
MSSSEPASHISLLDDPETGADK | Oxidation (M),phosph... | 2381.9934 | -0.20612 | 0.99342791 | 810 | OE4101R | yes | yes | 1 | 1 | 0 | 7 | 0
Raw Viewer - [Dataset1]
File
Tools
Window
Help
Raw Files
Map
MS
MS/MS
XIC
M(ox)pSSSEPASHISLLDDPETGADK; Scan 8456; 20070813_BM_HaSal_07
m/z (full)
Intensity
m/z (zoom)
Slide 46 of 46
"Office Theme"
English (U.S.)
104%
Start
I:\Halobacterium\1st+2n...
Raw Viewer - [Dataset1]
Result Explorer
Microsoft PowerPoint - [...
DE
18:56

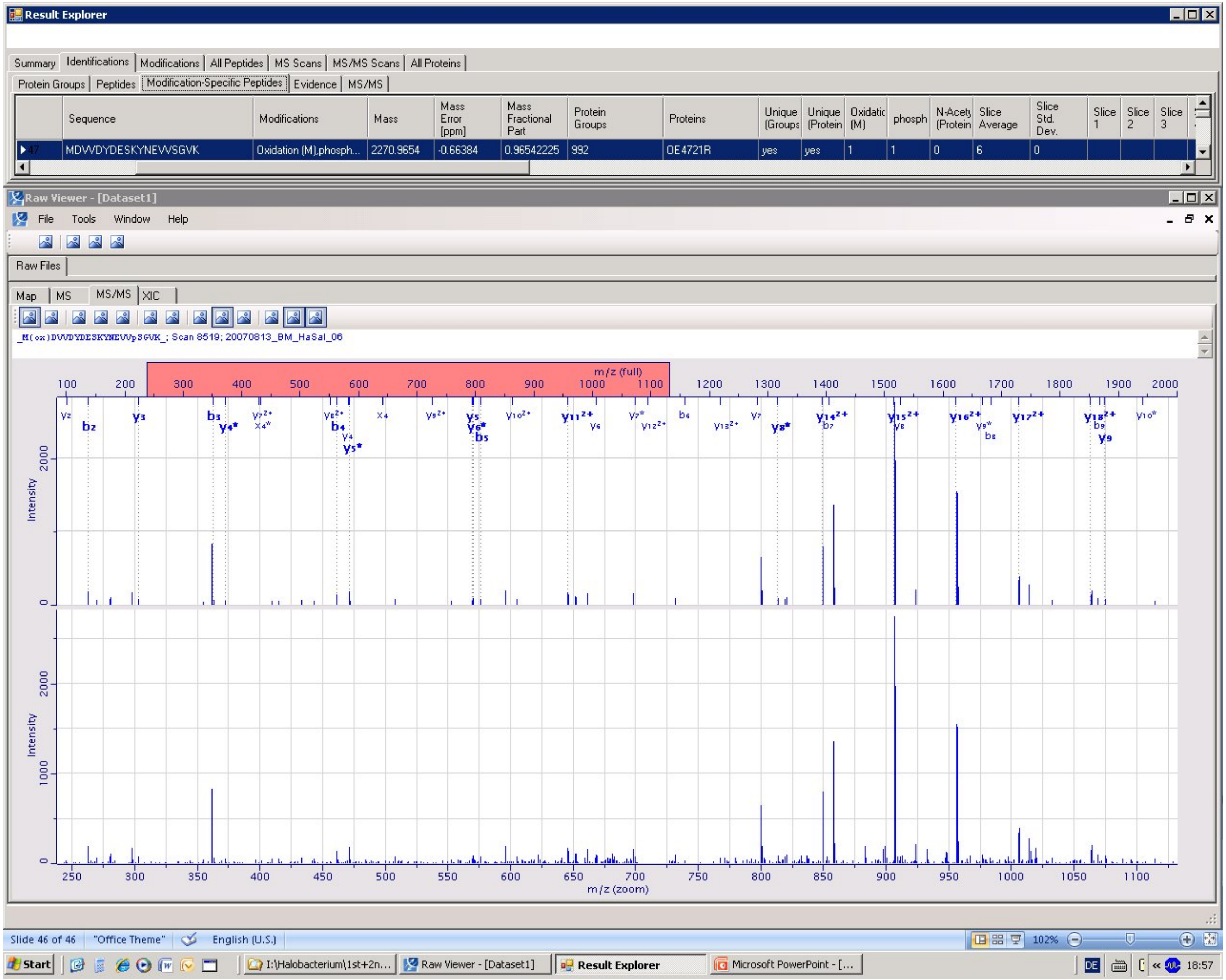
Result Explorer
Summary | Identifications | Modifications | All Peptides | MS Scans | MS/MS Scans | All Proteins
Protein Groups | Peptides | Modification-Specific Peptides | Evidence | MS/MS
Sequence | Modifications | Mass | Mass Error [ppm] | Mass Fractional Part | Protein Groups | Proteins | Unique (Groups | Unique (Protein | Oxidatic (M) | phosph | N-Acety (Protein | Slice Average | Slice Std. Dev. | Slice 1 | Slice 2 | Slice 3
MDVVDYDESKYNEVVSGVK | Oxidation (M),phosph... | 2270.9654 | -0.66384 | 0.96542225 | 992 | OE4721R | yes | yes | 1 | 1 | 0 | 6 | 0
Raw Viewer - [Dataset1]
File Tools Window Help
Raw Files
Map MS MS/MS XIC
M(ox)DVVDYDESKYNEVVpSGVK; Scan 8519; 20070813_BM_HaSal_06
m/z (full)
Intensity
m/z (zoom)
Slide 46 of 46 "Office Theme" English (U.S.) 102%
Start I:\Halobacterium\1st+2n... Raw Viewer - [Dataset1] Result Explorer Microsoft PowerPoint - [... DE 18:57

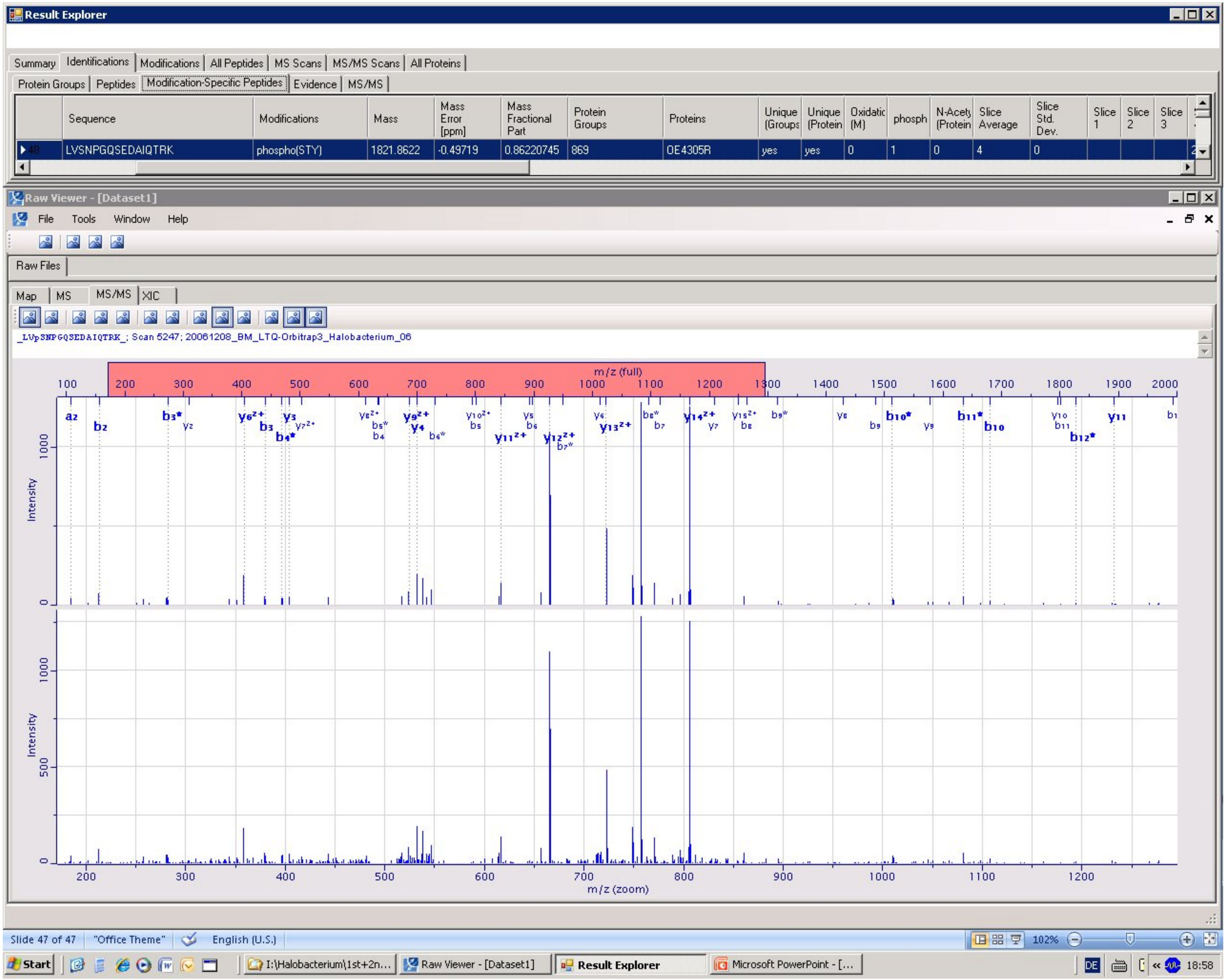
Result Explorer
Summary
Identifications
Modifications
All Peptides
MS Scans
MS/MS Scans
All Proteins
Protein Groups
Peptides
Modification-Specific Peptides
Evidence
MS/MS
Sequence
Modifications
Mass
Mass Error [ppm]
Mass Fractional Part
Protein Groups
Proteins
phosph
Slice Average
Slice Std. Dev.
Slice 1
Slice 2
Slice 3
LVSNPGQSEDAIQTRK
phospho(STY)
1821.8622
-0.49719
0.86220745
869
OE4305R
yes
yes
0
1
0
4
0
Raw Viewer - [Dataset1]
File
Tools
Window
Help
Raw Files
Map
MS
MS/MS
XIC
LVpSNPGQSEDAIQTRK; Scan 5247; 20061208_BM_LTQ-Orbitrap3_Halobacterium_06
m/z (full)
Intensity
m/z (zoom)
Slide 47 of 47
"Office Theme"
English (U.S.)
102%
Start
Raw Viewer - [Dataset1]
Result Explorer
18:58

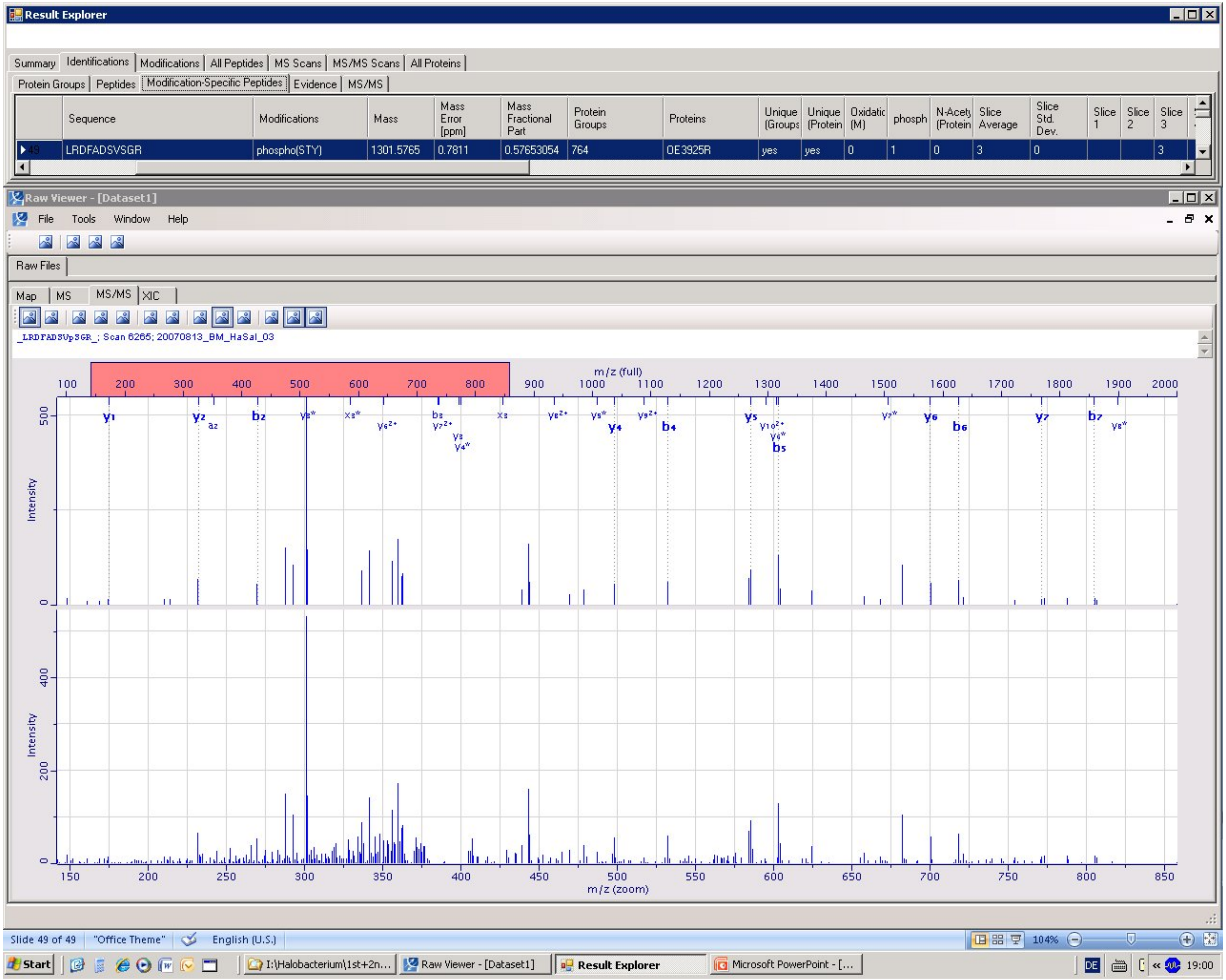
Result Explorer
Summary | Identifications | Modifications | All Peptides | MS Scans | MS/MS Scans | All Proteins
Protein Groups | Peptides | Modification-Specific Peptides | Evidence | MS/MS
Sequence | Modifications | Mass | Mass Error [ppm] | Mass Fractional Part | Protein Groups | Proteins | Unique (Groups | Unique (Protein | Oxidatic (M) | phosph | N-Acety (Protein | Slice Average | Slice Std. Dev. | Slice 1 | Slice 2 | Slice 3
LRDFADSVSGR | phospho(STY) | 1301.5765 | 0.7811 | 0.57653054 | 764 | OE3925R | yes | yes | 0 | 1 | 0 | 3 | 0 | | | 3
Raw Viewer - [Dataset1]
File Tools Window Help
Raw Files
Map | MS | MS/MS | XIC
LRDFADSVpSGR; Scan 6265; 20070813_BM_HaSal_03
m/z (full)
Intensity
m/z (zoom)
Slide 49 of 49 "Office Theme" English (U.S.) 104%
Start I:\Halobacterium\1st+2n... Raw Viewer - [Dataset1] Result Explorer Microsoft PowerPoint - [... DE 19:00

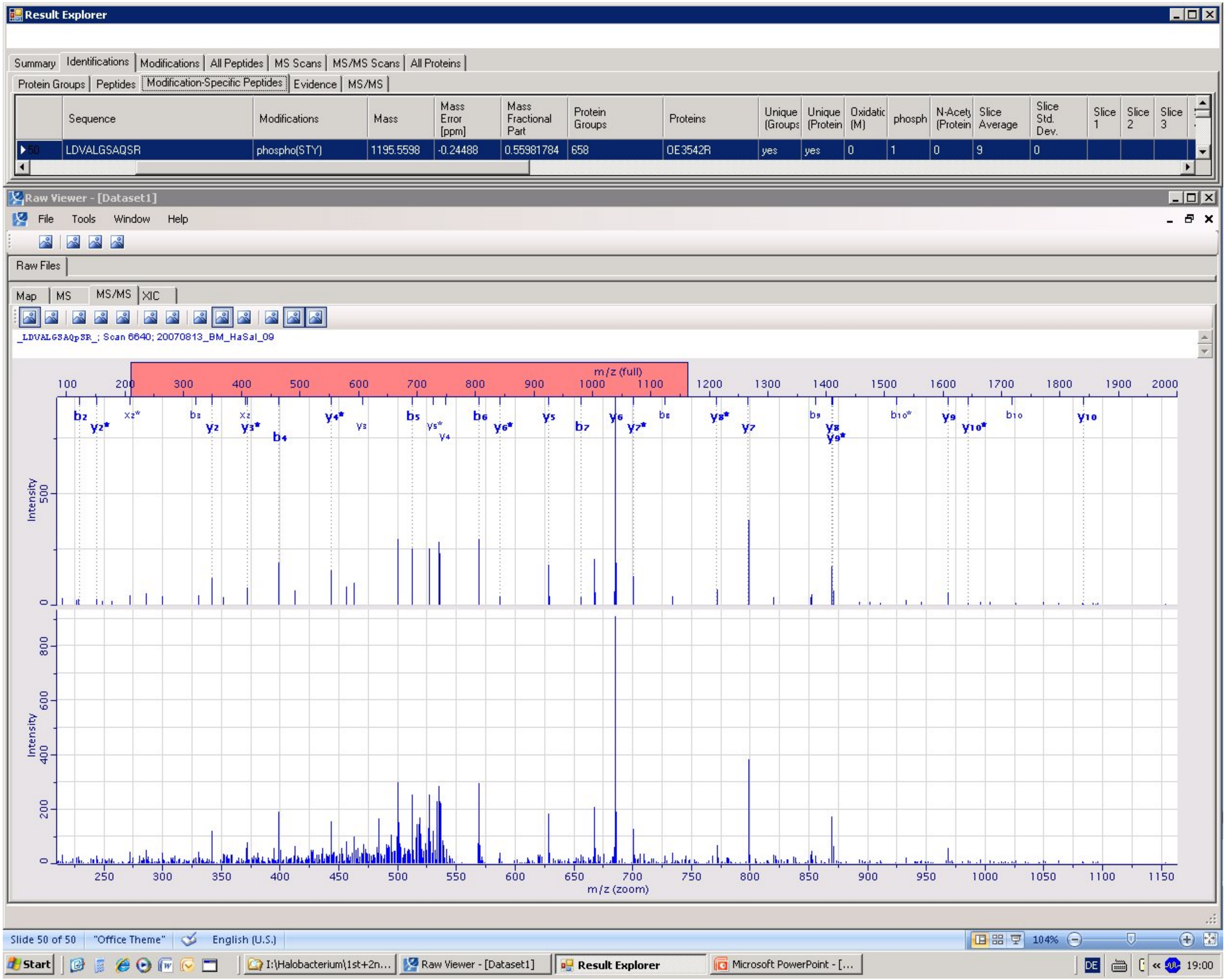
Result Explorer
Summary
Identifications
Modifications
All Peptides
MS Scans
MS/MS Scans
All Proteins
Protein Groups
Peptides
Modification-Specific Peptides
Evidence
MS/MS
Sequence
Modifications
Mass
Mass Error [ppm]
Mass Fractional Part
Protein Groups
Proteins
LDVALGSAQSR
phospho(STY)
1195.5598
-0.24488
0.55981784
658
OE3542R
Raw Viewer - [Dataset1]
File
Tools
Window
Help
Raw Files
Map
MS
MS/MS
XIC
LDVALGSAQpSR; Scan 6640; 20070813_BM_HaSal_09
m/z (full)
Intensity
m/z (zoom)
Slide 50 of 50
"Office Theme"
English (U.S.)
Start
Raw Viewer - [Dataset1]
Result Explorer
19:00

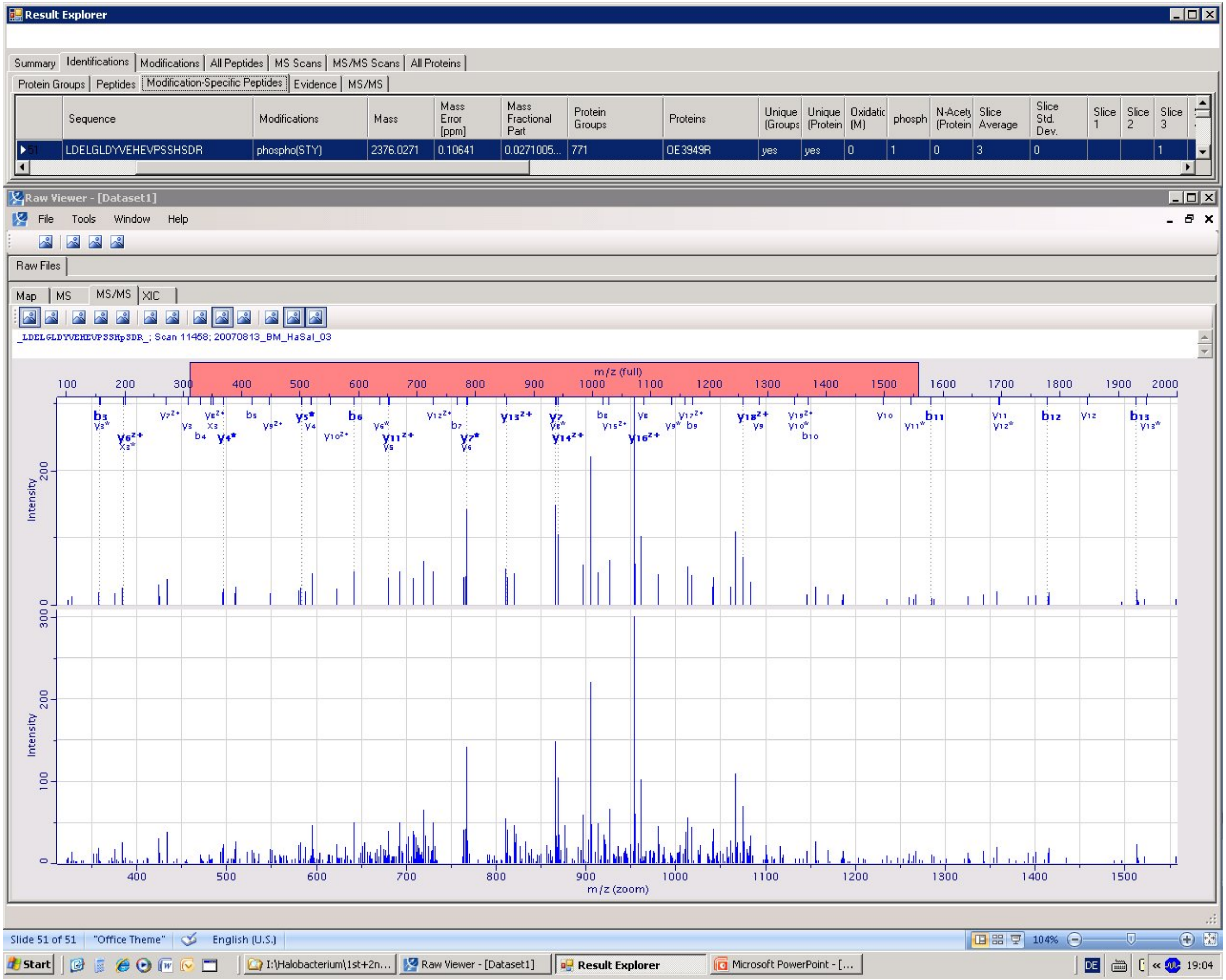
Result Explorer
Summary | Identifications | Modifications | All Peptides | MS Scans | MS/MS Scans | All Proteins
Protein Groups | Peptides | Modification-Specific Peptides | Evidence | MS/MS
Sequence | Modifications | Mass | Mass Error [ppm] | Mass Fractional Part | Protein Groups | Proteins | Unique (Groups) | Unique (Protein) | Oxidation (M) | phosph | N-Acety (Protein) | Slice Average | Slice Std. Dev. | Slice 1 | Slice 2 | Slice 3
LDELGLDYVEHEVPSSHSDR | phospho(STY) | 2376.0271 | 0.10641 | 0.0271005... | 771 | OE3949R | yes | yes | 0 | 1 | 0 | 3 | 0 | | | 1
Raw Viewer - [Dataset1]
File Tools Window Help
Raw Files
Map | MS | MS/MS | XIC
LDELGLDYVEHEVPSSHpSDR; Scan 11458; 20070813_BM_HaSal_03
m/z (full)
Intensity
m/z (zoom)
Slide 51 of 51 "Office Theme" English (U.S.)
Start
Raw Viewer - [Dataset1]
Result Explorer
Microsoft PowerPoint - [...
19:04

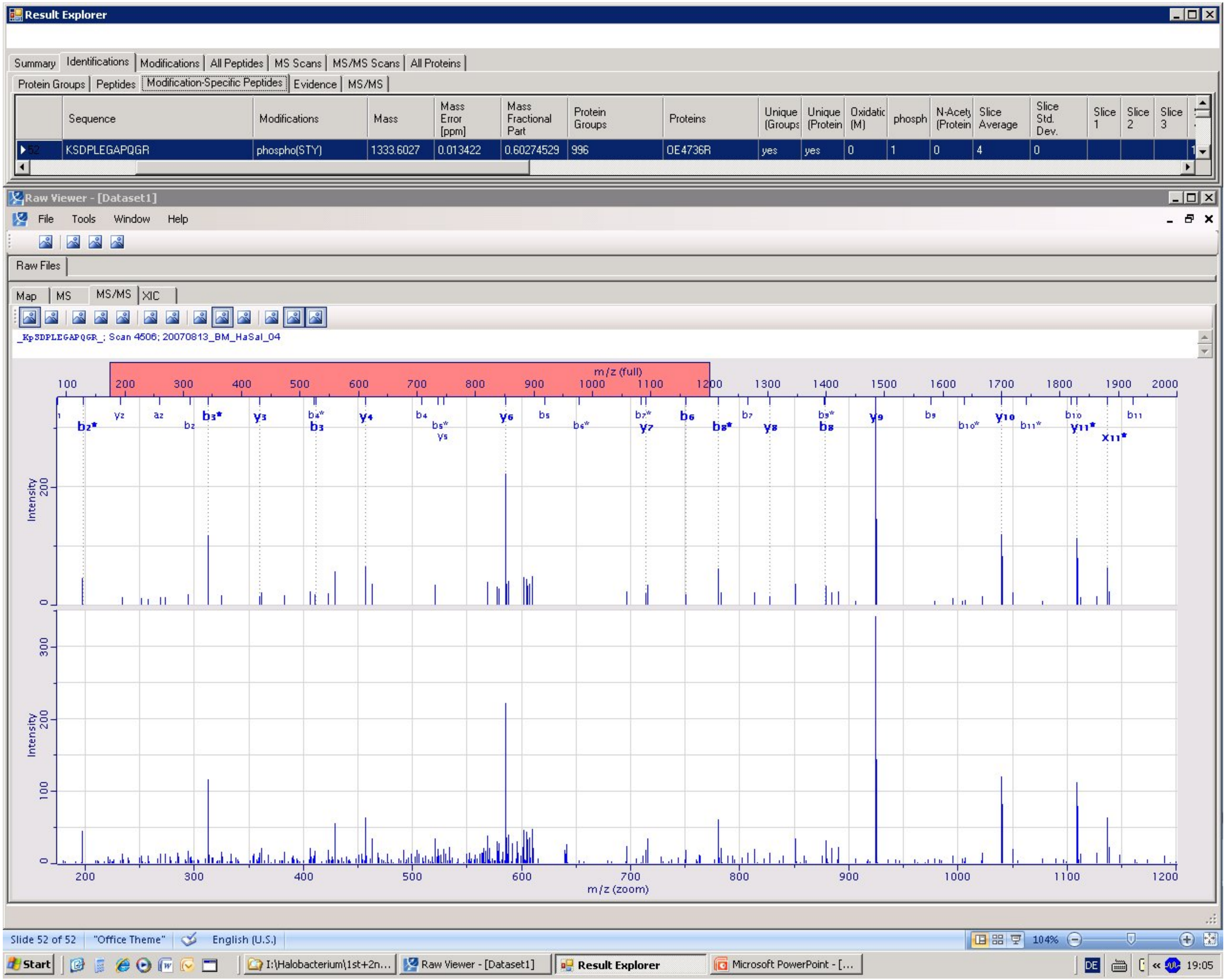
Result Explorer
Summary | Identifications | Modifications | All Peptides | MS Scans | MS/MS Scans | All Proteins
Protein Groups | Peptides | Modification-Specific Peptides | Evidence | MS/MS
Sequence | Modifications | Mass | Mass Error [ppm] | Mass Fractional Part | Protein Groups | Proteins | Unique (Groups | Unique (Protein | Oxidatic (M) | phosph | N-Acety (Protein | Slice Average | Slice Std. Dev. | Slice 1 | Slice 2 | Slice 3
KSDPLEGAPQGR | phospho(STY) | 1333.6027 | 0.013422 | 0.60274529 | 996 | OE4736R | yes | yes | 0 | 1 | 0 | 4 | 0
Raw Viewer - [Dataset1]
File Tools Window Help
Raw Files
Map | MS | MS/MS | XIC
KpSDPLEGAPQGR; Scan 4506; 20070813_BM_HaSal_04
m/z (full)
Intensity
m/z (zoom)
Slide 52 of 52 "Office Theme" English (U.S.) 104%
Start
Raw Viewer - [Dataset1]
Result Explorer
Microsoft PowerPoint - [...
19:05

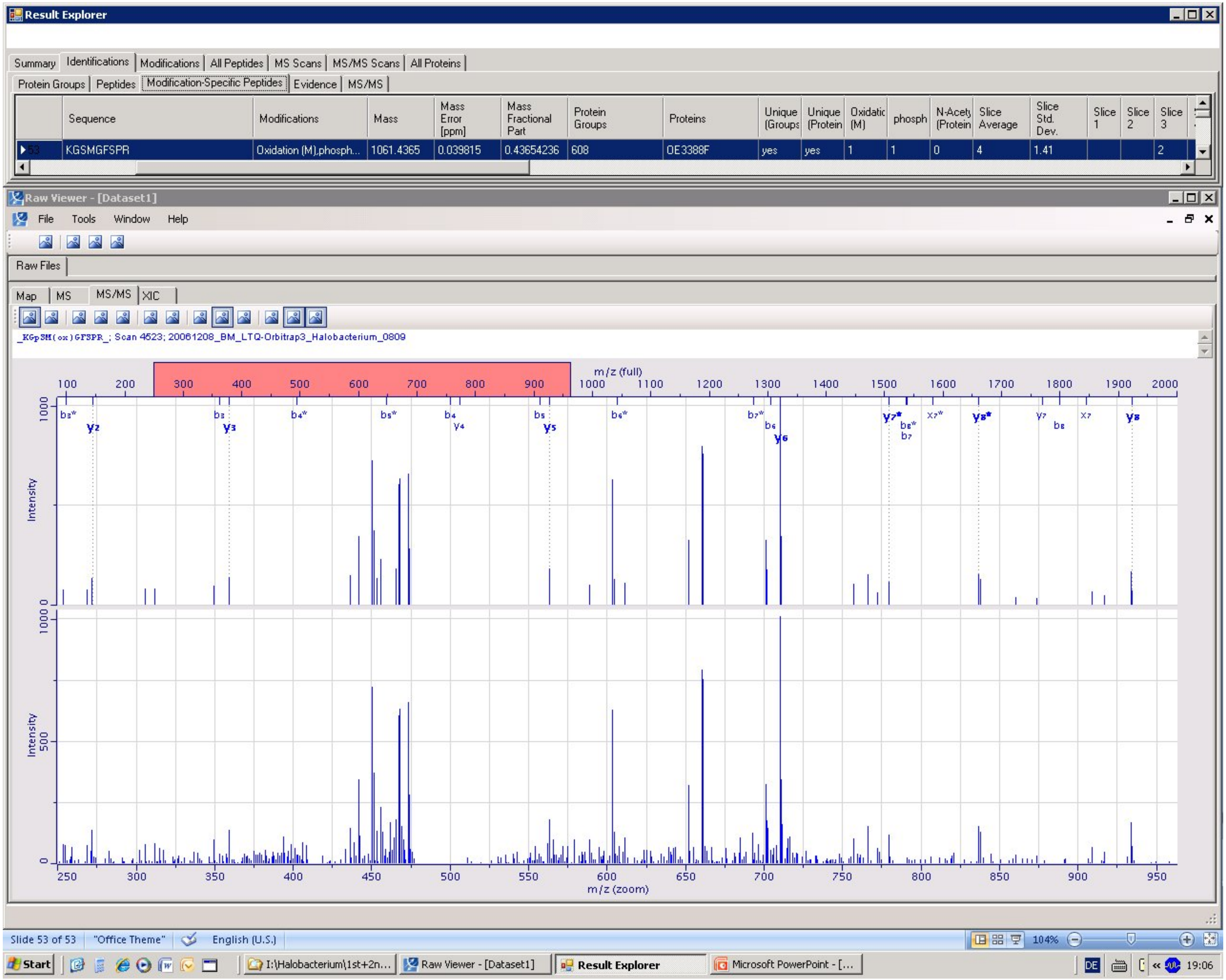
Result Explorer
Summary
Identifications
Modifications
All Peptides
MS Scans
MS/MS Scans
All Proteins
Protein Groups
Peptides
Modification-Specific Peptides
Evidence
MS/MS
Sequence
Modifications
Mass
Mass Error [ppm]
Mass Fractional Part
Protein Groups
Proteins
KGSMGFSPR
Oxidation (M),phosph...
1061.4365
0.039815
0.43654236
608
OE3388F
Raw Viewer - [Dataset1]
File
Tools
Window
Help
Raw Files
Map
MS
MS/MS
XIC
KGpSM(ox)GFSPR; Scan 4523; 20061208_BM_LTQ-Orbitrap3_Halobacterium_0809
m/z (full)
Intensity
m/z (zoom)
Slide 53 of 53
"Office Theme"
English (U.S.)
Start
Raw Viewer - [Dataset1]
Result Explorer
19:06

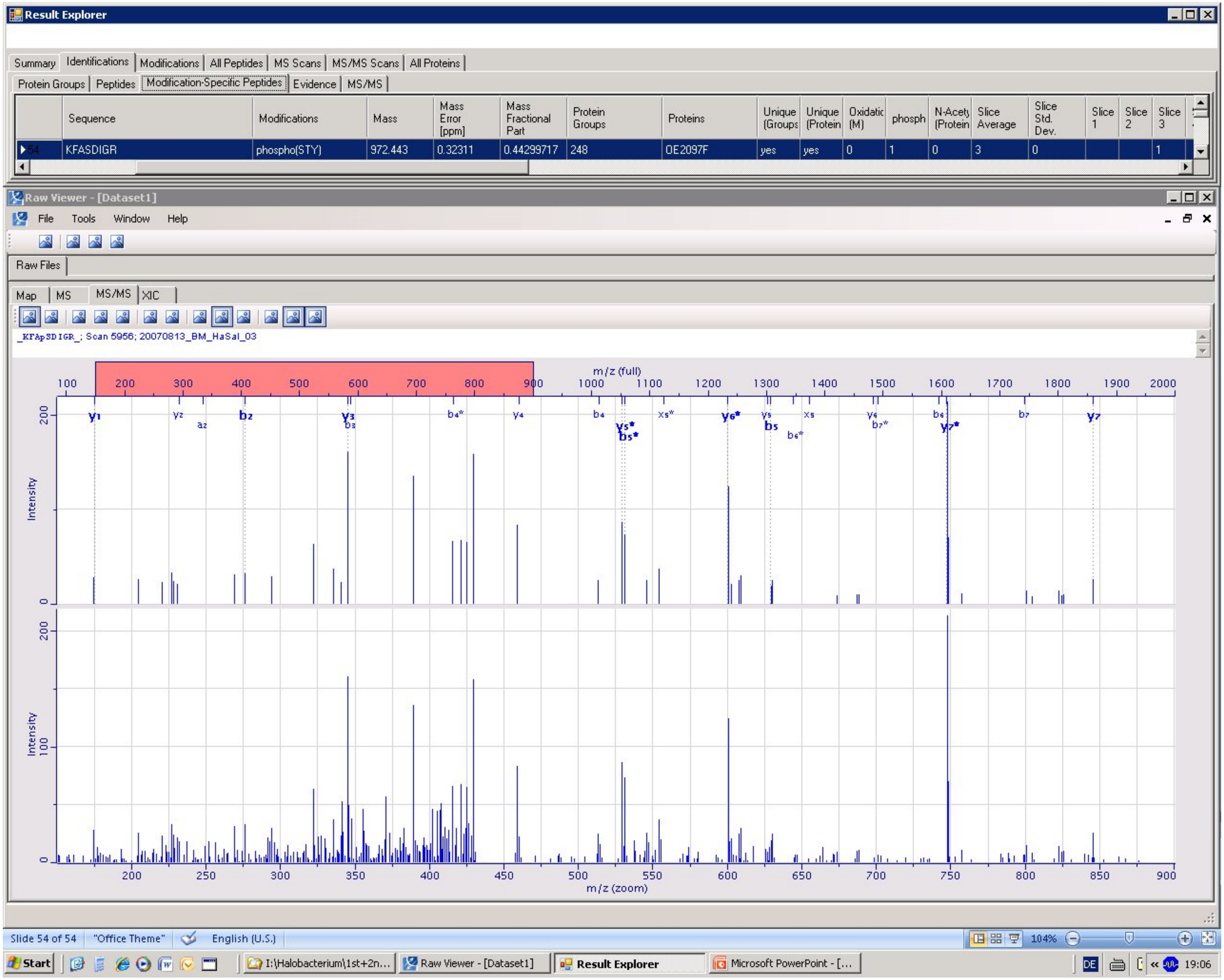
Result Explorer
Summary | Identifications | Modifications | All Peptides | MS Scans | MS/MS Scans | All Proteins
Protein Groups | Peptides | Modification-Specific Peptides | Evidence | MS/MS
Sequence | Modifications | Mass | Mass Error [ppm] | Mass Fractional Part | Protein Groups | Proteins | Unique (Groups | Unique (Protein | Oxidatic (M) | phosph | N-Acety (Protein | Slice Average | Slice Std. Dev. | Slice 1 | Slice 2 | Slice 3
KFASDIGR | phospho(STY) | 972.443 | 0.32311 | 0.44299717 | 248 | OE2097F | yes | yes | 0 | 1 | 0 | 3 | 0 | 1
Raw Viewer - [Dataset1]
File Tools Window Help
Raw Files
Map MS MS/MS XIC
KFApSDIGR; Scan 5956; 20070813_BM_HaSal_03
m/z (full)
Intensity
m/z (zoom)
y1 y2 a2 b2 y3 b3 b4* y4 b4 y5* b5* x5* y6* y5 b5 x5 b6* y6 b7* b6 y7* b7 y7
Slide 54 of 54 "Office Theme" English (U.S.)
Start
I:\Halobacterium\1st+2n...
Raw Viewer - [Dataset1]
Result Explorer
Microsoft PowerPoint - [...
DE
19:06

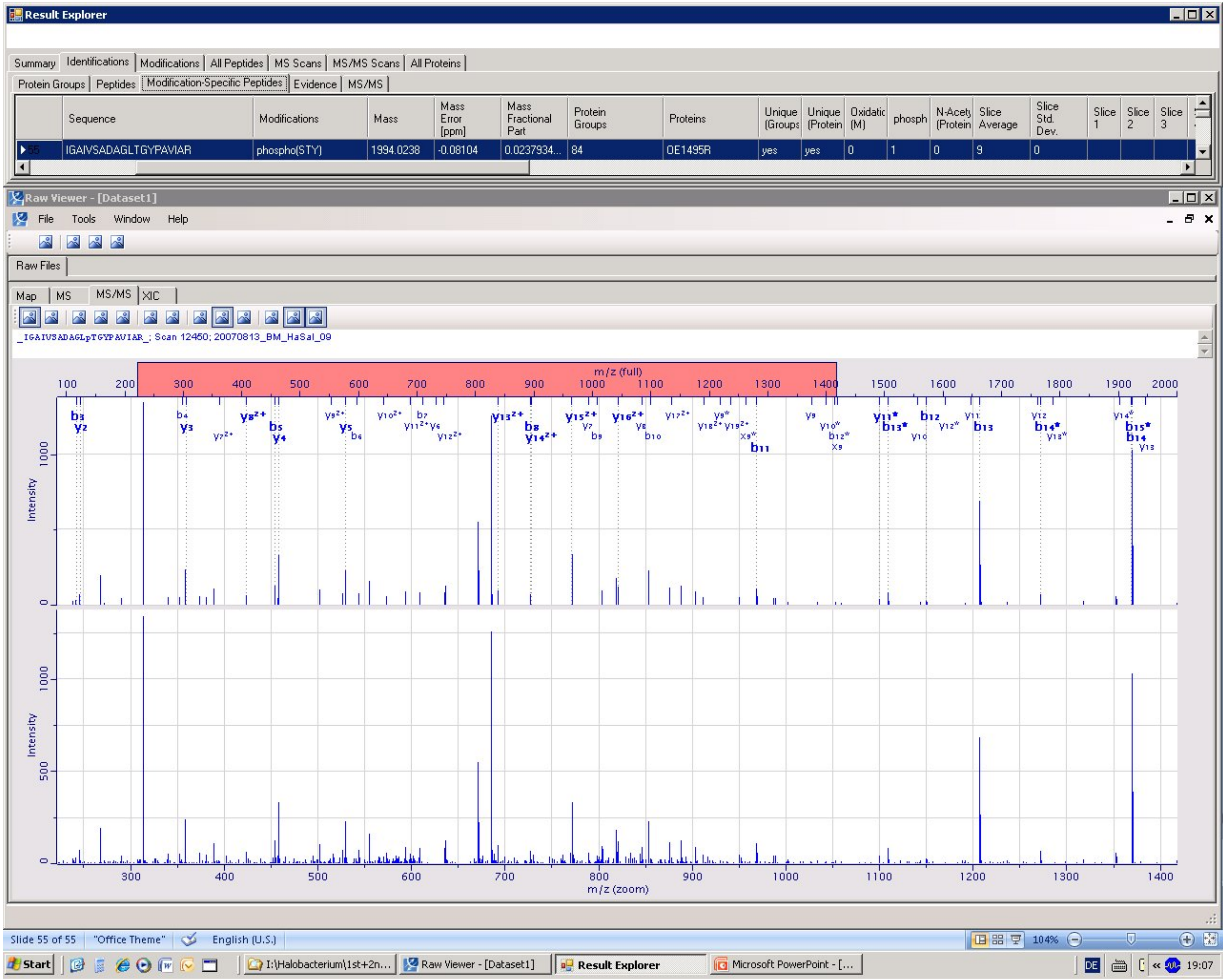
Result Explorer
Summary
Identifications
Modifications
All Peptides
MS Scans
MS/MS Scans
All Proteins
Protein Groups
Peptides
Modification-Specific Peptides
Evidence
MS/MS
Sequence
Modifications
Mass
Mass Error [ppm]
Mass Fractional Part
Protein Groups
Proteins
IGAIVSADAGLTGYPAVIAR
phospho(STY)
1994.0238
-0.08104
0.0237934...
84
OE1495R
Raw Viewer - [Dataset1]
File
Tools
Window
Help
Raw Files
Map
MS
MS/MS
XIC
IGAIVSADAGLpTGYPAVIAR; Scan 12450; 20070813_BM_HaSal_09
m/z (full)
Intensity
m/z (zoom)
Slide 55 of 55
"Office Theme"
English (U.S.)
Start
Raw Viewer - [Dataset1]
Result Explorer
Microsoft PowerPoint - [...
19:07

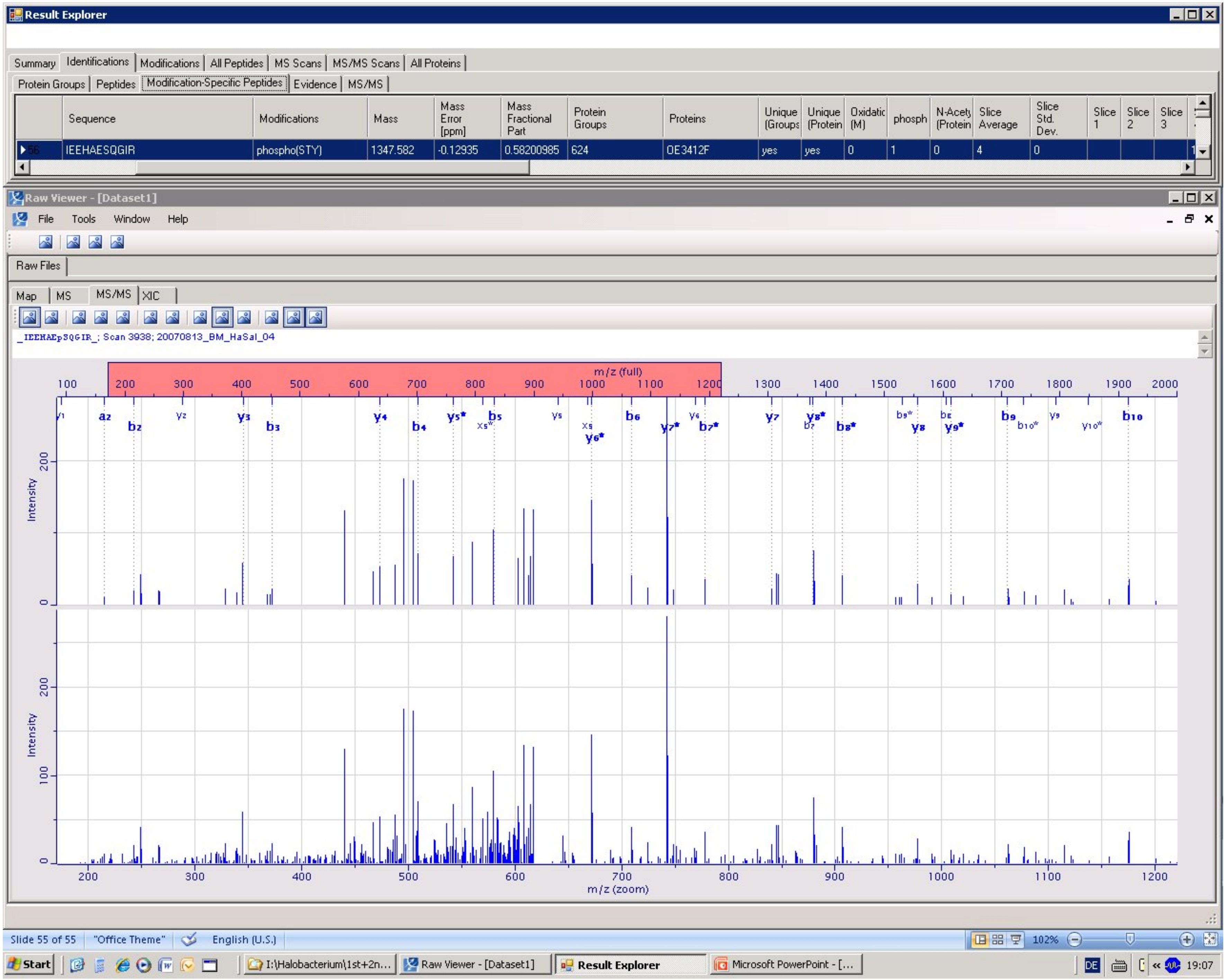
Result Explorer
Summary | Identifications | Modifications | All Peptides | MS Scans | MS/MS Scans | All Proteins
Protein Groups | Peptides | Modification-Specific Peptides | Evidence | MS/MS
Sequence | Modifications | Mass | Mass Error [ppm] | Mass Fractional Part | Protein Groups | Proteins | Unique (Groups | Unique (Protein | Oxidatic (M) | phosph | N-Acety (Protein | Slice Average | Slice Std. Dev. | Slice 1 | Slice 2 | Slice 3
IEEHAESQGIR | phospho(STY) | 1347.582 | -0.12935 | 0.58200985 | 624 | OE3412F | yes | yes | 0 | 1 | 0 | 4 | 0
Raw Viewer - [Dataset1]
File Tools Window Help
Raw Files
Map | MS | MS/MS | XIC
IEEHAEpSQGIR; Scan 3938; 20070813_BM_HaSal_04
m/z (full)
Intensity
m/z (zoom)
Slide 55 of 55 "Office Theme" English (U.S.) 102%
Start I:\Halobacterium\1st+2n... Raw Viewer - [Dataset1] Result Explorer Microsoft PowerPoint - [...
DE 19:07

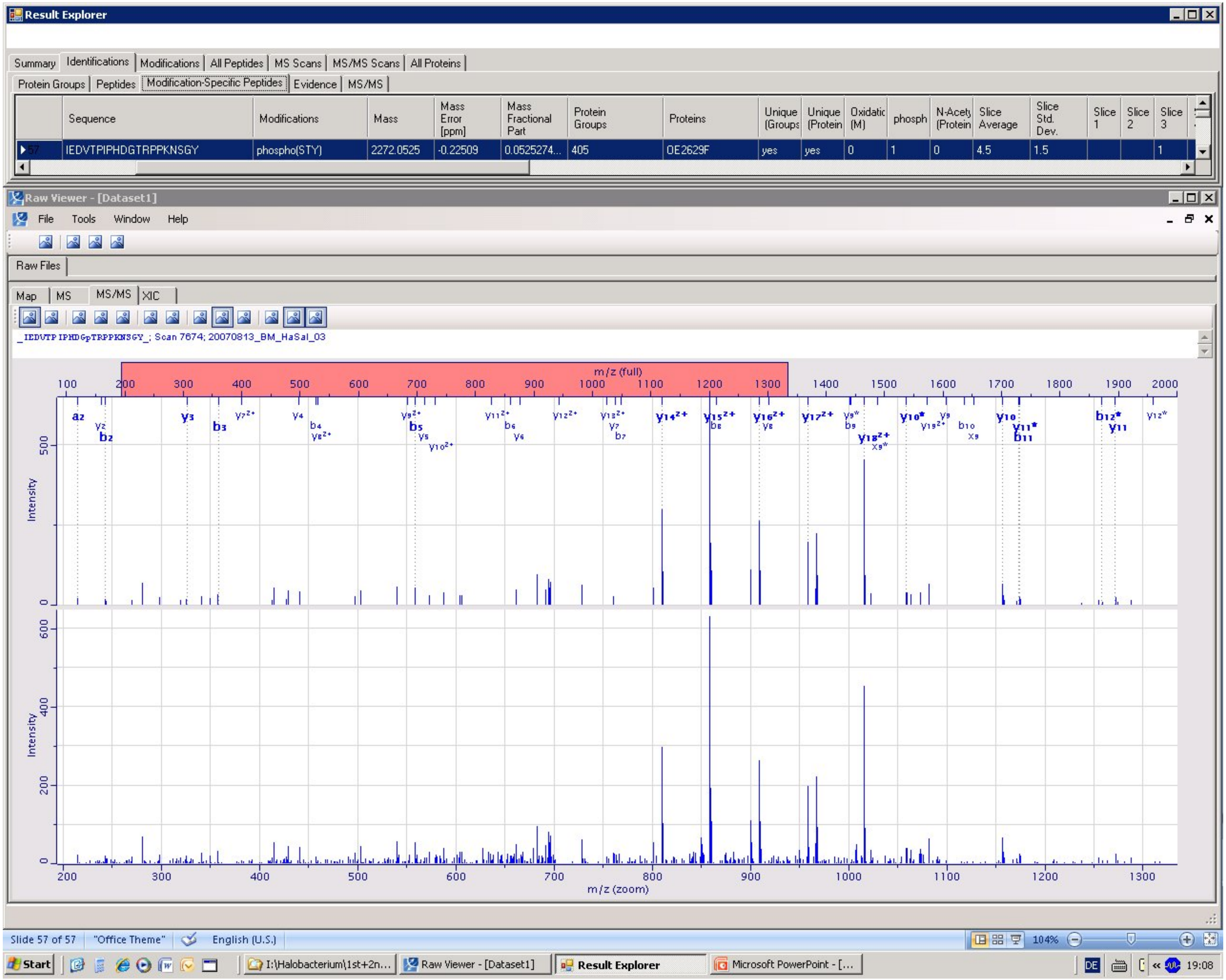
Result Explorer
Summary | Identifications | Modifications | All Peptides | MS Scans | MS/MS Scans | All Proteins
Protein Groups | Peptides | Modification-Specific Peptides | Evidence | MS/MS
Sequence | Modifications | Mass | Mass Error [ppm] | Mass Fractional Part | Protein Groups | Proteins | Unique (Groups | Unique (Protein | Oxidatic (M) | phosph | N-Acety (Protein | Slice Average | Slice Std. Dev. | Slice 1 | Slice 2 | Slice 3
IEDVTPIPHDGTRPPKNSGY | phospho(STY) | 2272.0525 | -0.22509 | 0.0525274... | 405 | OE2629F | yes | yes | 0 | 1 | 0 | 4.5 | 1.5 | 1
Raw Viewer - [Dataset1]
File Tools Window Help
Raw Files
Map MS MS/MS XIC
IEDVTPIPHDGpTRPPKNSGY; Scan 7674; 20070813_BM_HaSal_03
m/z (full)
Intensity
m/z (zoom)
Slide 57 of 57 "Office Theme" English (U.S.) 104%
Start I:\Halobacterium\1st+2n... Raw Viewer - [Dataset1] Result Explorer Microsoft PowerPoint - [... DE 19:08

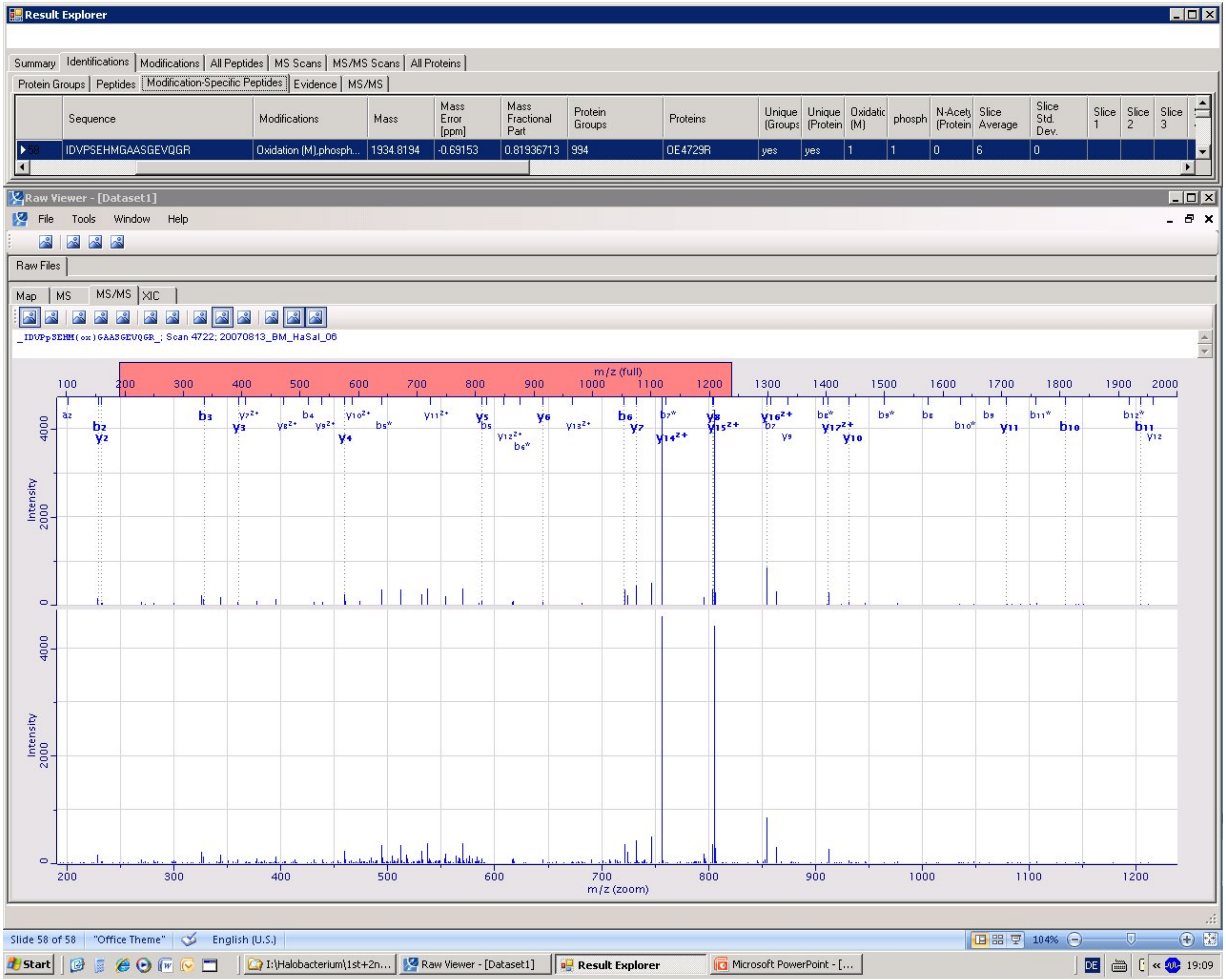
Result Explorer
Summary
Identifications
Modifications
All Peptides
MS Scans
MS/MS Scans
All Proteins
Protein Groups
Peptides
Modification-Specific Peptides
Evidence
MS/MS
Sequence
Modifications
Mass
Mass Error [ppm]
Mass Fractional Part
Protein Groups
Proteins
Slice Average
Slice Std. Dev.
IDVPSEHMGAASGEVQGR
Oxidation (M),phosph...
1934.8194
-0.69153
0.81936713
994
OE4729R
Raw Viewer - [Dataset1]
File
Tools
Window
Help
Raw Files
Map
MS
MS/MS
XIC
m/z (full)
Intensity
m/z (zoom)
Slide 58 of 58
"Office Theme"
English (U.S.)
Start
Raw Viewer - [Dataset1]
Result Explorer
19:09

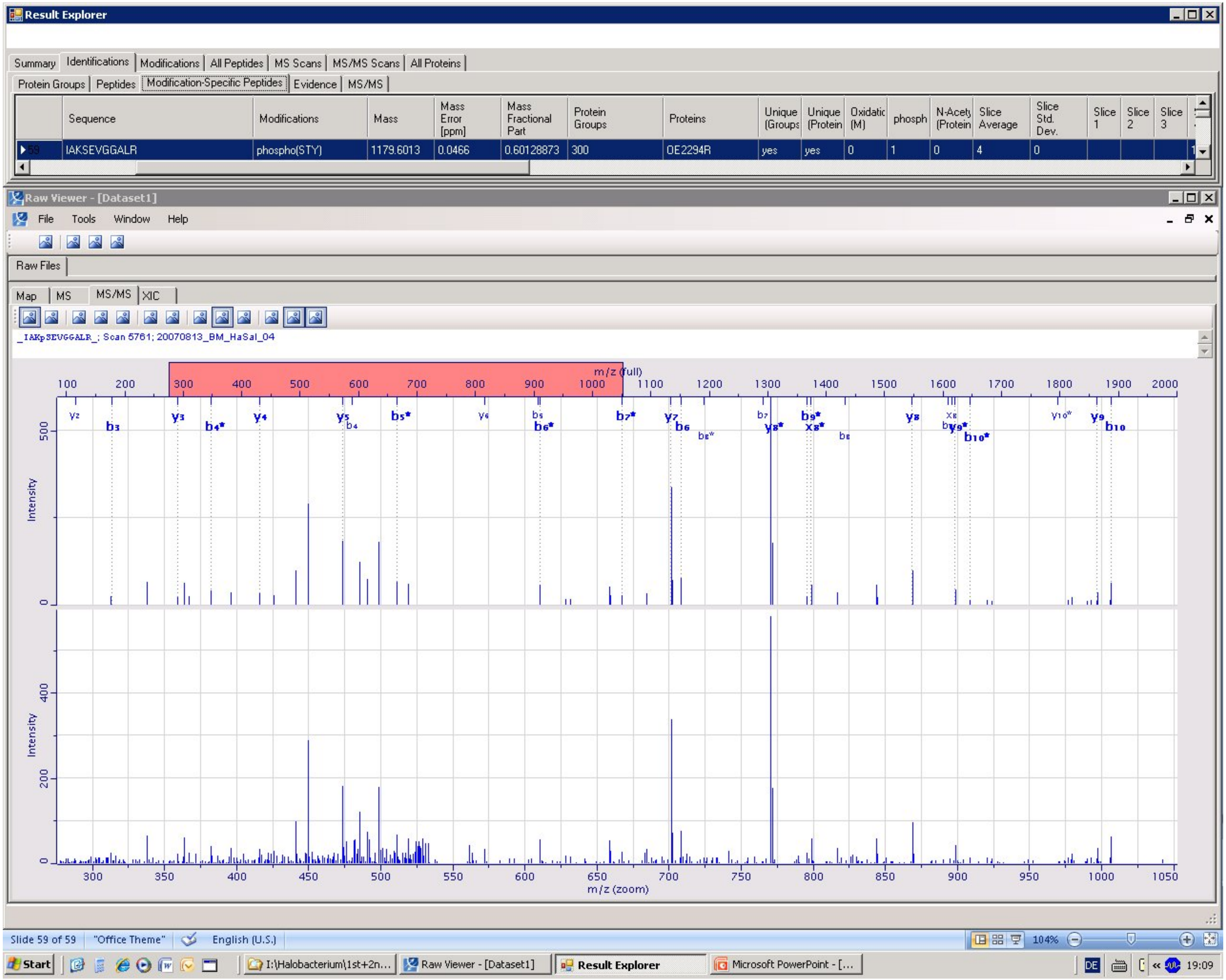

Result Explorer
Summary | Identifications | Modifications | All Peptides | MS Scans | MS/MS Scans | All Proteins
Protein Groups | Peptides | Modification-Specific Peptides | Evidence | MS/MS
Sequence | Modifications | Mass | Mass Error [ppm] | Mass Fractional Part | Protein Groups | Proteins | Unique (Groups | Unique (Protein | Oxidatic (M) | phosph | N-Acety (Protein | Slice Average | Slice Std. Dev. | Slice 1 | Slice 2 | Slice 3
IAKSEVGGALR | phospho(STY) | 1179.6013 | 0.0466 | 0.60128873 | 300 | OE2294R | yes | yes | 0 | 1 | 0 | 4 | 0
Raw Viewer - [Dataset1]
File Tools Window Help
Raw Files
Map | MS | MS/MS | XIC
IAKpSEVGGALR; Scan 5761; 20070813_BM_HaSal_04
m/z (full)
Intensity
m/z (zoom)
Slide 59 of 59 "Office Theme" English (U.S.) 104%
Start | I:\Halobacterium\1st+2n... | Raw Viewer - [Dataset1] | Result Explorer | Microsoft PowerPoint - [...
DE 19:09

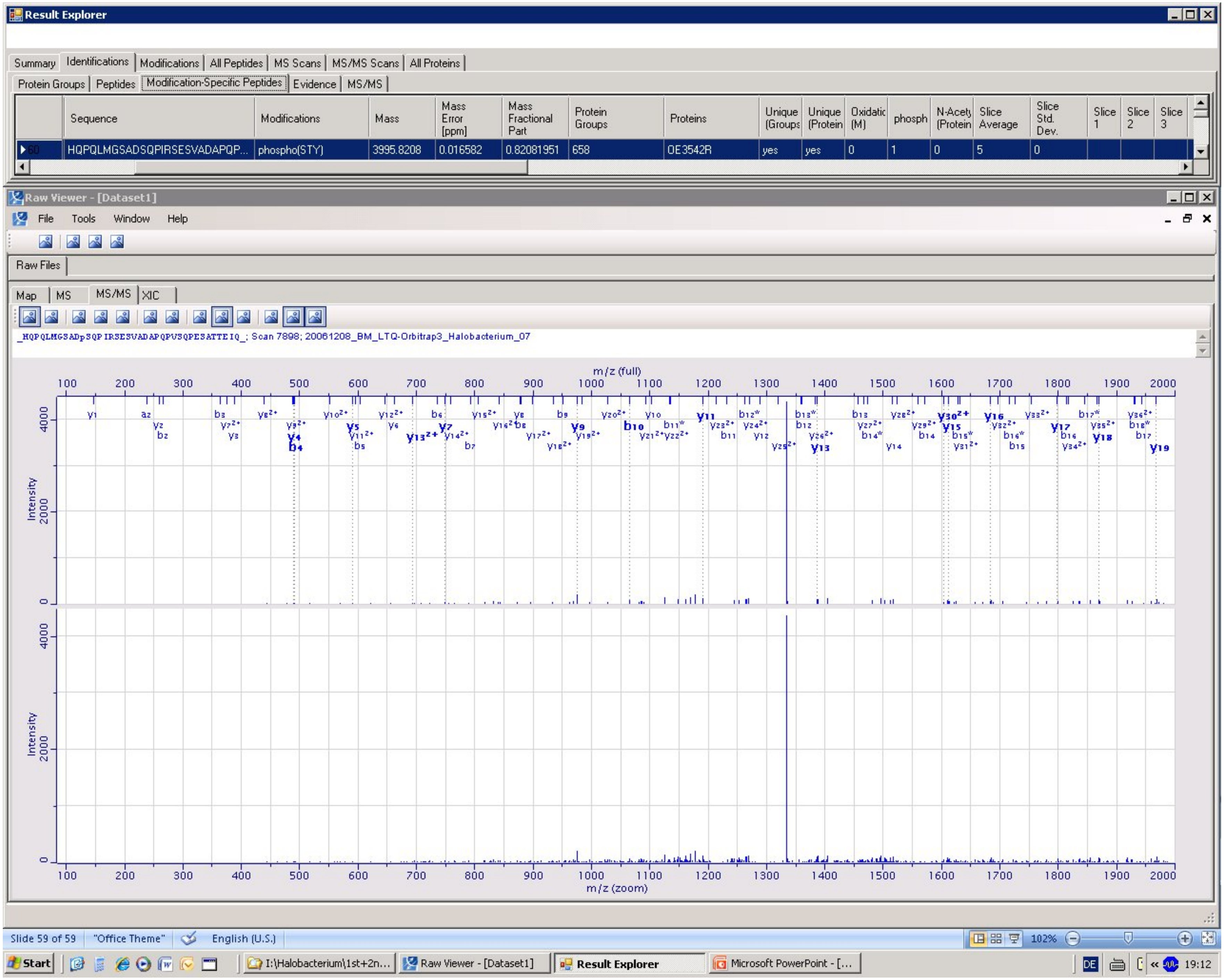
Result Explorer
Summary
Identifications
Modifications
All Peptides
MS Scans
MS/MS Scans
All Proteins
Protein Groups
Peptides
Modification-Specific Peptides
Evidence
MS/MS
Sequence
Modifications
Mass
Mass Error [ppm]
Mass Fractional Part
Protein Groups
Proteins
HQPQLMGSADSQPIRSESVADAPQP...
phospho(STY)
3995.8208
0.016582
0.82081951
658
OE3542R
Raw Viewer - [Dataset1]
File
Tools
Window
Help
Raw Files
Map
MS
MS/MS
XIC
Scan 7898; 20061208_BM_LTQ-Orbitrap3_Halobacterium_07
m/z (full)
Intensity
m/z (zoom)
Slide 59 of 59
"Office Theme"
English (U.S.)
Start
Microsoft PowerPoint - [...
19:12

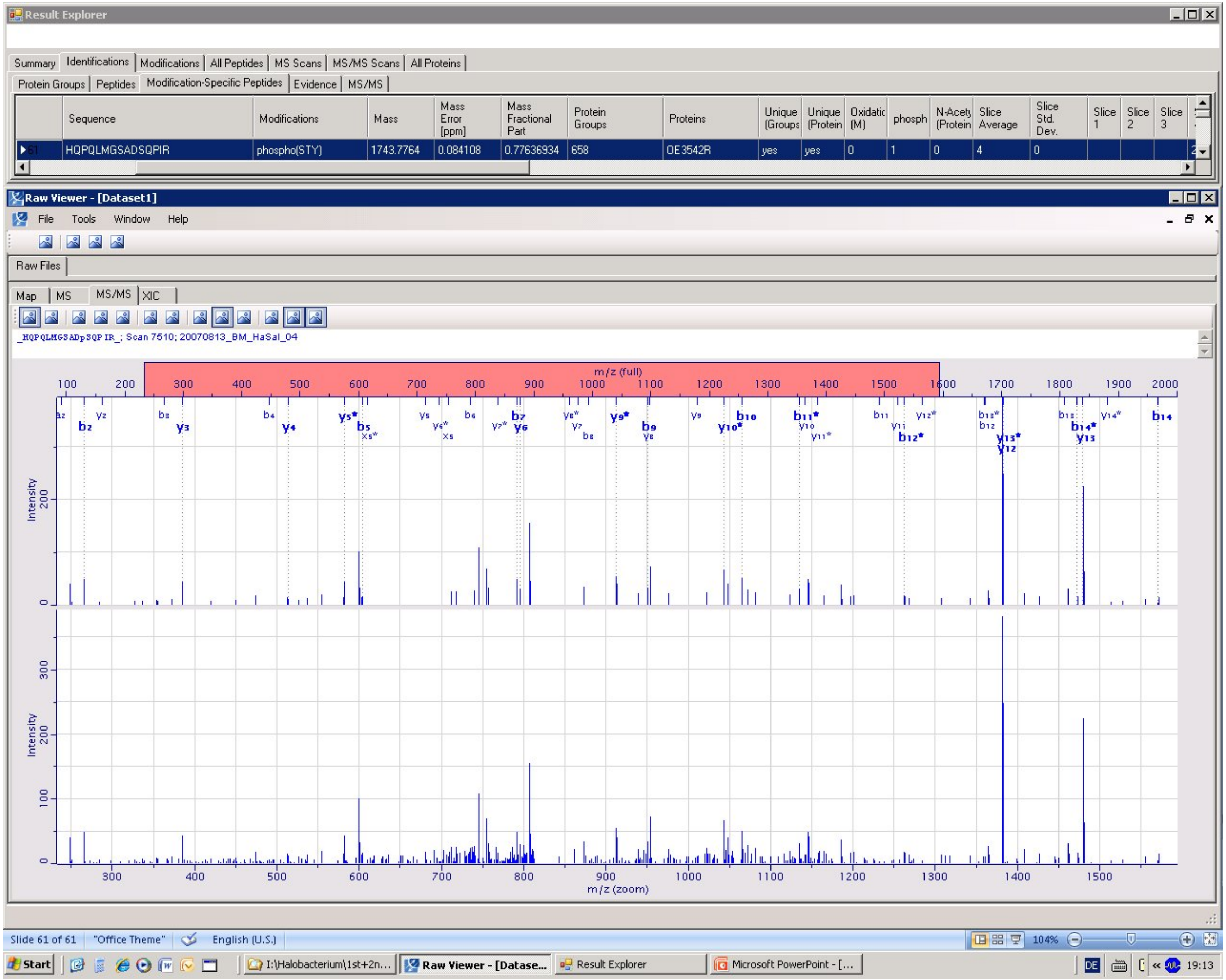
Result Explorer
Summary | Identifications | Modifications | All Peptides | MS Scans | MS/MS Scans | All Proteins
Protein Groups | Peptides | Modification-Specific Peptides | Evidence | MS/MS
Sequence | Modifications | Mass | Mass Error [ppm] | Mass Fractional Part | Protein Groups | Proteins | Unique (Groups | Unique (Protein | Oxidatic (M) | phosph | N-Acety (Protein | Slice Average | Slice Std. Dev. | Slice 1 | Slice 2 | Slice 3
HQPQLMGSADSQPIR | phospho(STY) | 1743.7764 | 0.084108 | 0.77636934 | 658 | OE3542R | yes | yes | 0 | 1 | 0 | 4 | 0
Raw Viewer - [Dataset1]
File Tools Window Help
Raw Files
Map MS MS/MS XIC
HQPQLMGSADpSQPIR; Scan 7510; 20070813_BM_HaSal_04
m/z (full)
Intensity
m/z (zoom)
Slide 61 of 61 "Office Theme" English (U.S.) 104%
Start I:\Halobacterium\1st+2n... Raw Viewer - [Datase... Result Explorer Microsoft PowerPoint - [... DE 19:13

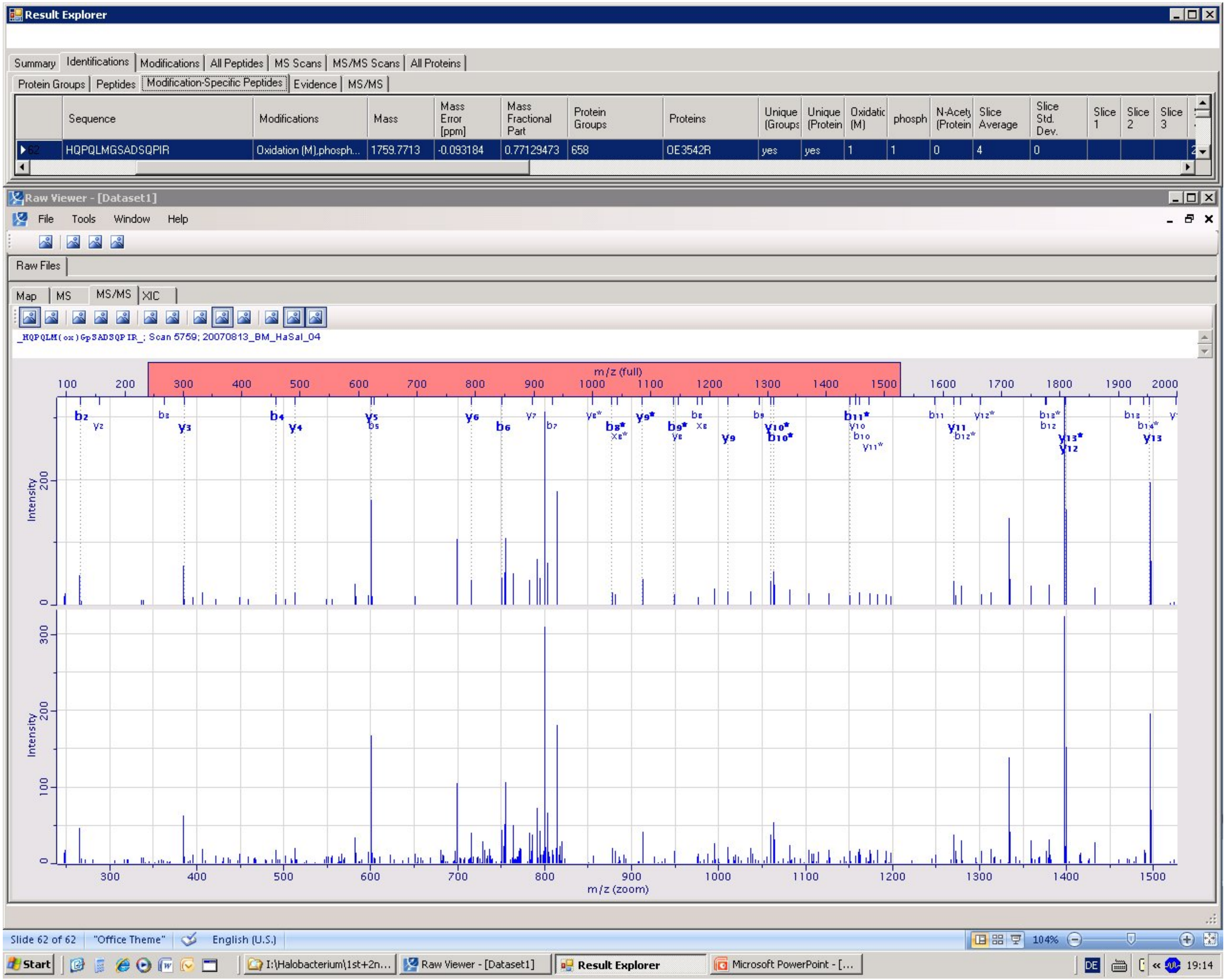
Result Explorer
Summary
Identifications
Modifications
All Peptides
MS Scans
MS/MS Scans
All Proteins
Protein Groups
Peptides
Modification-Specific Peptides
Evidence
MS/MS
Sequence
Modifications
Mass
Mass Error [ppm]
Mass Fractional Part
Protein Groups
Proteins
HQPQLMGSADSQPIR
Oxidation (M),phosph...
1759.7713
-0.093184
0.77129473
658
OE3542R
Raw Viewer - [Dataset1]
File
Tools
Window
Help
Raw Files
Map
MS
MS/MS
XIC
HQPQLM(ox)GpSADSQPIR; Scan 5759; 20070813_BM_HaSal_04
m/z (full)
Intensity
m/z (zoom)
Slide 62 of 62
"Office Theme"
English (U.S.)
Start
Raw Viewer - [Dataset1]
Result Explorer
Microsoft PowerPoint - [...
19:14

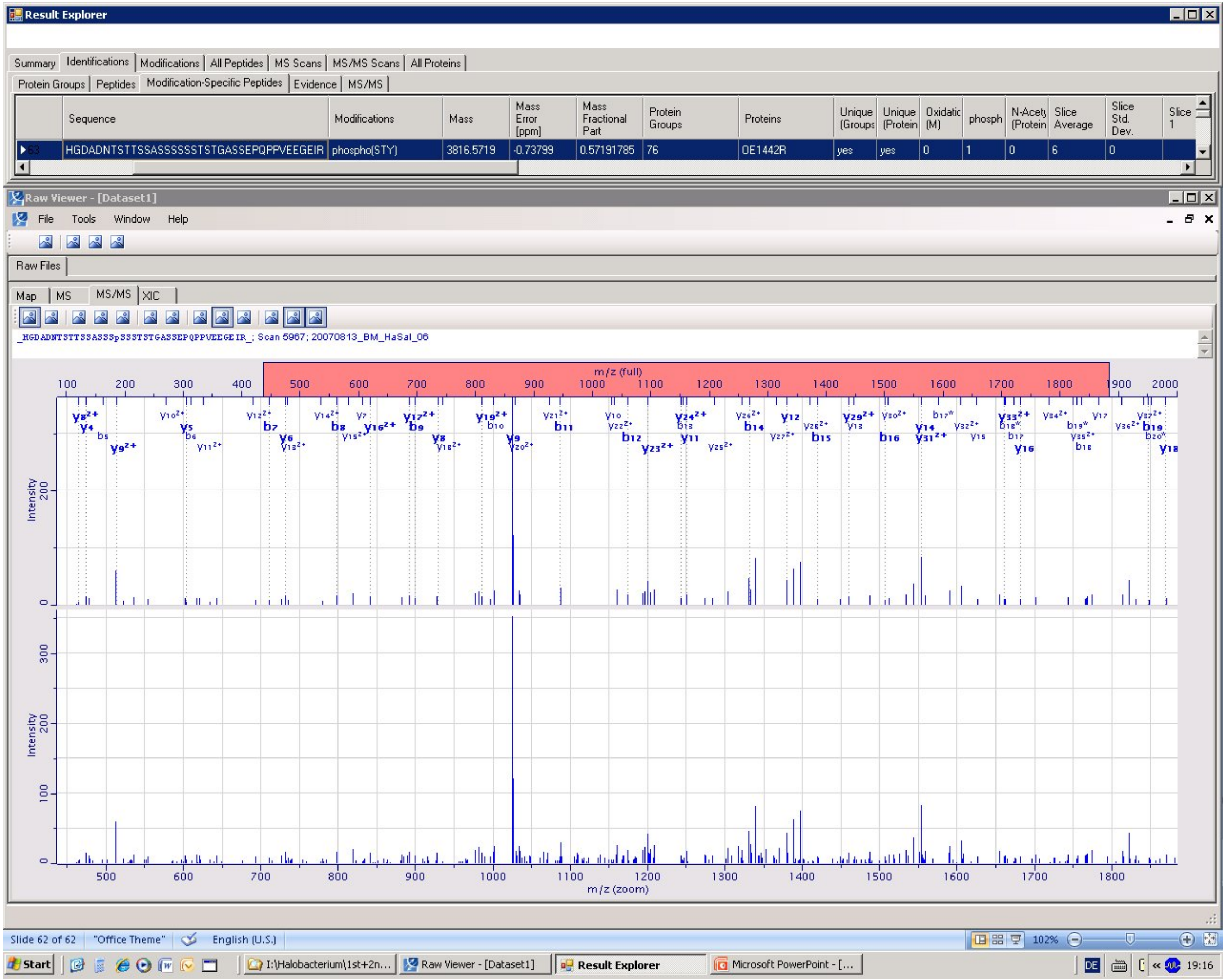
Result Explorer
Summary
Identifications
Modifications
All Peptides
MS Scans
MS/MS Scans
All Proteins
Protein Groups
Peptides
Modification-Specific Peptides
Evidence
MS/MS
Sequence
Modifications
Mass
Mass Error [ppm]
Mass Fractional Part
Protein Groups
Proteins
Unique (Groups
Unique (Protein
Oxidatic (M)
phosph
N-Acety (Protein
Slice Average
Slice Std. Dev.
Slice 1
HGDADNTSTTSSASSSSSSTSTGASSEPQPPVEEGEIR
phospho(STY)
3816.5719
-0.73799
0.57191785
76
OE1442R
yes
yes
0
1
0
6
0
Raw Viewer - [Dataset1]
File
Tools
Window
Help
Raw Files
Map
MS
MS/MS
XIC
HGDADNTSTTSSASSSpSSSTSTGASSEPQPPVEEGEIR; Scan 5967; 20070813_BM_HaSal_06
m/z (full)
Intensity
m/z (zoom)
Slide 62 of 62
"Office Theme"
English (U.S.)
102%
Start
I:\Halobacterium\1st+2n...
Raw Viewer - [Dataset1]
Result Explorer
Microsoft PowerPoint - [...
DE
19:16

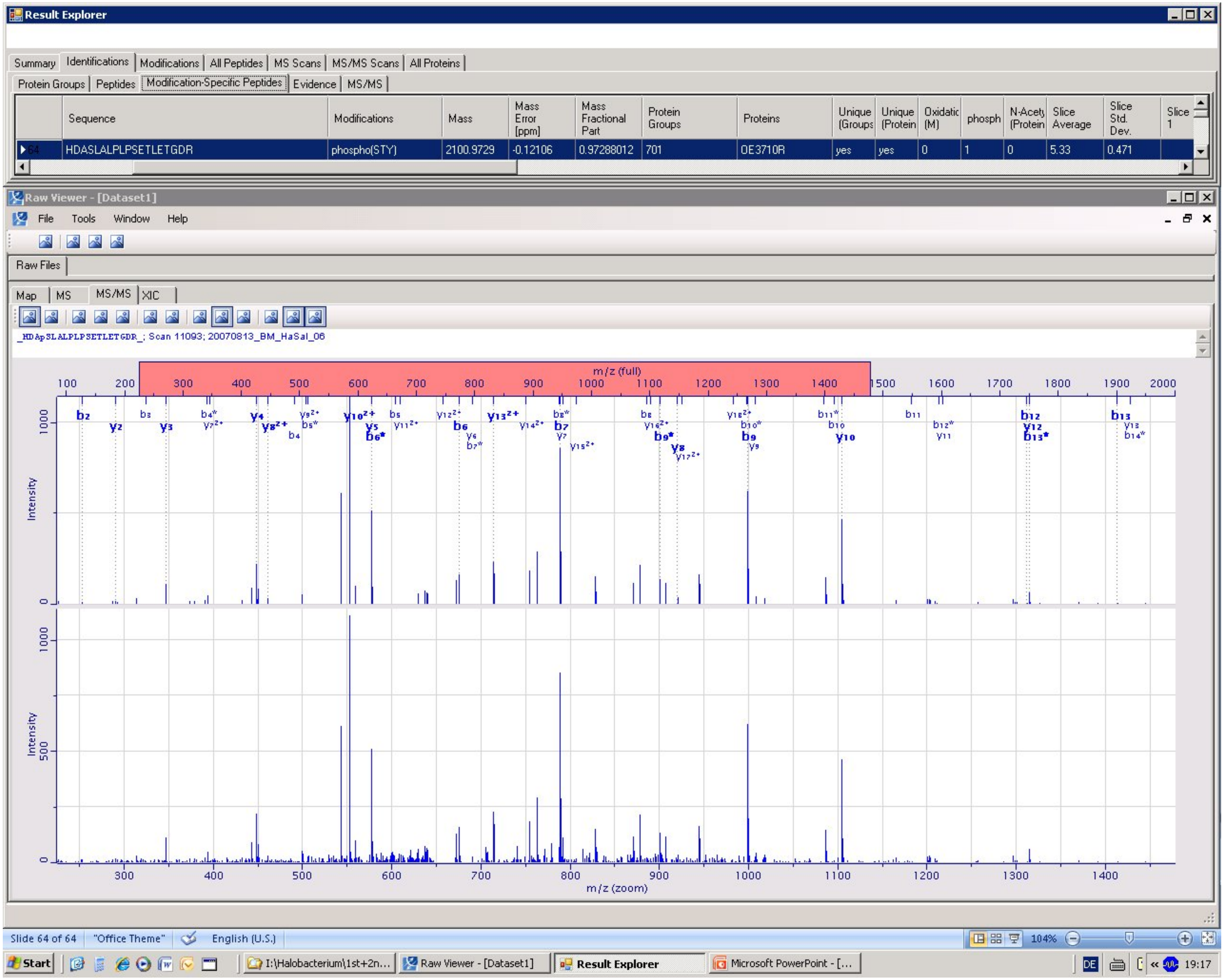

Result Explorer
Summary | Identifications | Modifications | All Peptides | MS Scans | MS/MS Scans | All Proteins
Protein Groups | Peptides | Modification-Specific Peptides | Evidence | MS/MS
Sequence | Modifications | Mass | Mass Error [ppm] | Mass Fractional Part | Protein Groups | Proteins | Unique (Groups | Unique (Protein | Oxidatic (M) | phosph | N-Acety (Protein | Slice Average | Slice Std. Dev. | Slice 1
HDASLALPLPSETLETGDR | phospho(STY) | 2100.9729 | -0.12106 | 0.97288012 | 701 | OE3710R | yes | yes | 0 | 1 | 0 | 5.33 | 0.471
Raw Viewer - [Dataset1]
File Tools Window Help
Raw Files
Map | MS | MS/MS | XIC
HDApSLALPLPSETLETGDR; Scan 11093; 20070813_BM_HaSal_06
m/z (full)
Intensity
m/z (zoom)
Slide 64 of 64 "Office Theme" English (U.S.)
Start
I:\Halobacterium\1st+2n...
Raw Viewer - [Dataset1]
Result Explorer
Microsoft PowerPoint - [...
19:17

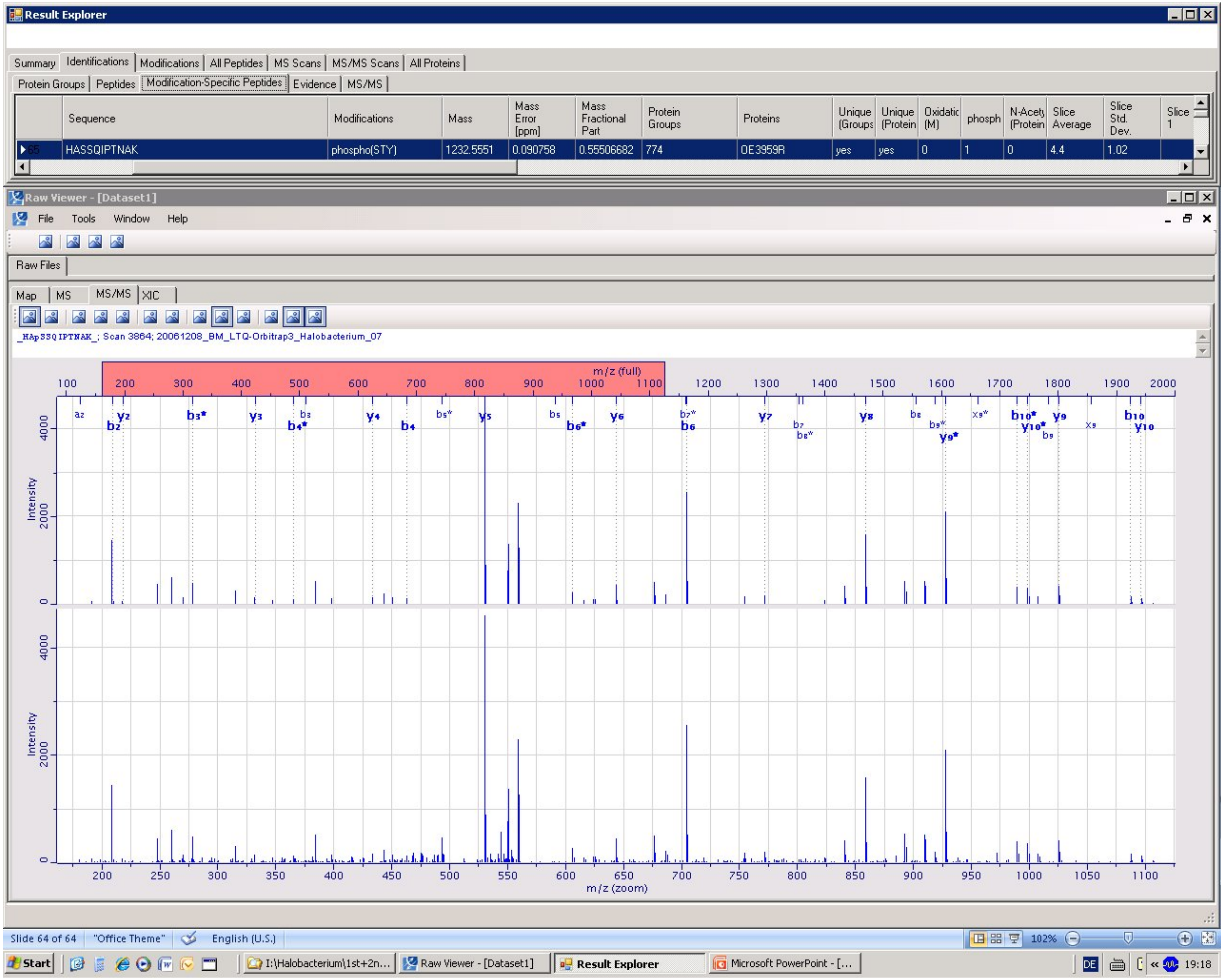
Result Explorer
Summary
Identifications
Modifications
All Peptides
MS Scans
MS/MS Scans
All Proteins
Protein Groups
Peptides
Modification-Specific Peptides
Evidence
MS/MS
Sequence
Modifications
Mass
Mass Error [ppm]
Mass Fractional Part
Protein Groups
Proteins
HASSQIPTNAK
phospho(STY)
1232.5551
0.090758
0.55506682
774
OE3959R
Raw Viewer - [Dataset1]
File
Tools
Window
Help
Raw Files
Map
MS
MS/MS
XIC
HApSSQIPTNAK; Scan 3864; 20061208_BM_LTQ-Orbitrap3_Halobacterium_07
m/z (full)
Intensity
m/z (zoom)
Slide 64 of 64
"Office Theme"
English (U.S.)
Start
Raw Viewer - [Dataset1]
Result Explorer
19:18

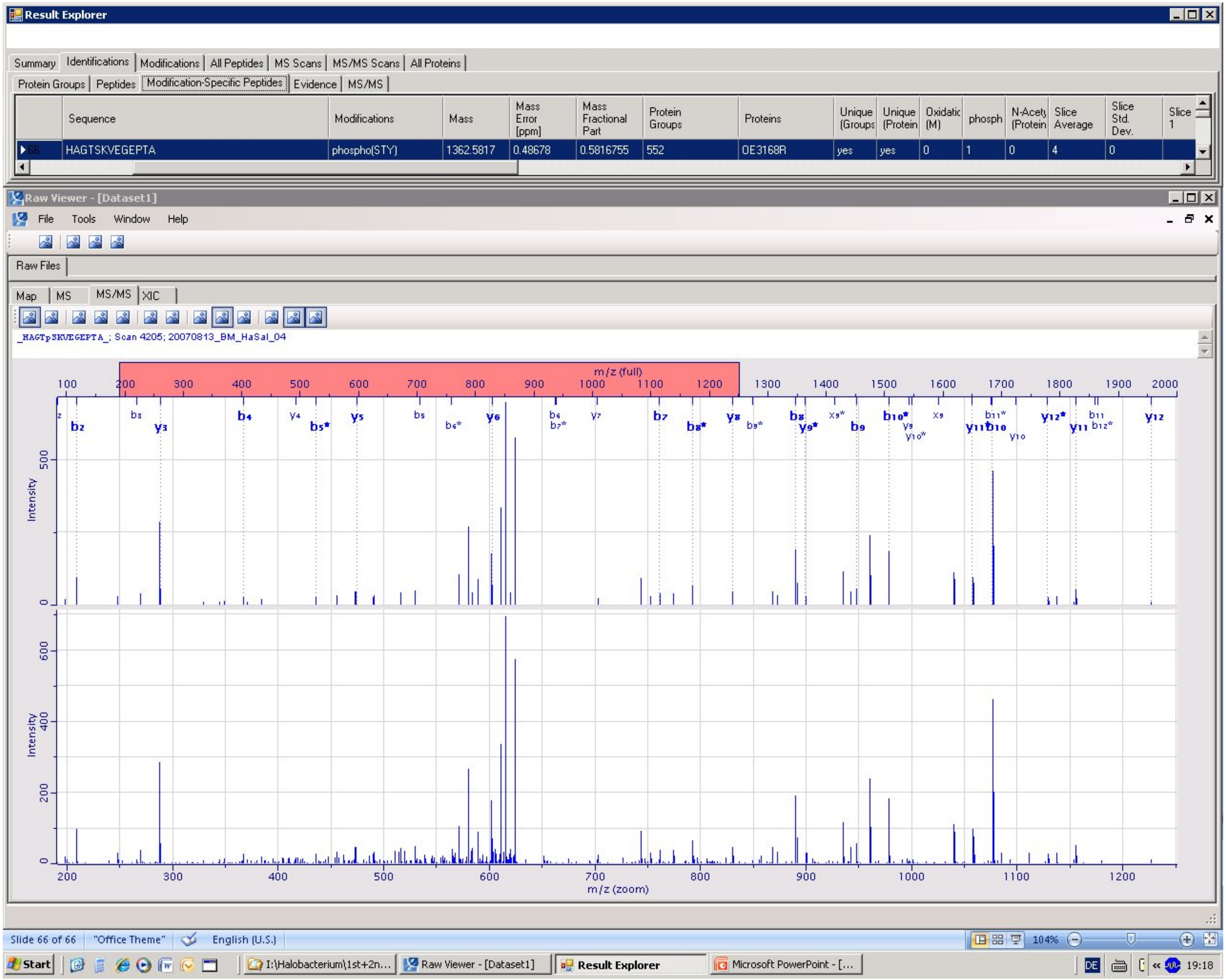
Result Explorer
Summary
Identifications
Modifications
All Peptides
MS Scans
MS/MS Scans
All Proteins
Protein Groups
Peptides
Modification-Specific Peptides
Evidence
MS/MS
Sequence
Modifications
Mass
Mass Error [ppm]
Mass Fractional Part
Protein Groups
Proteins
phosph
Slice Average
Slice Std. Dev.
HAGTSKVEGEPTA
phospho(STY)
1362.5817
0.48678
0.5816755
552
OE3168R
yes
yes
0
1
0
4
0
Raw Viewer - [Dataset1]
File
Tools
Window
Help
Raw Files
Map
MS
MS/MS
XIC
HAGTpSKVEGEPTA; Scan 4205; 20070813_BM_HaSal_04
m/z (full)
Intensity
m/z (zoom)
Slide 66 of 66
"Office Theme"
English (U.S.)
104%
Start
I:\Halobacterium\1st+2n...
Raw Viewer - [Dataset1]
Result Explorer
Microsoft PowerPoint - [...
DE
19:18

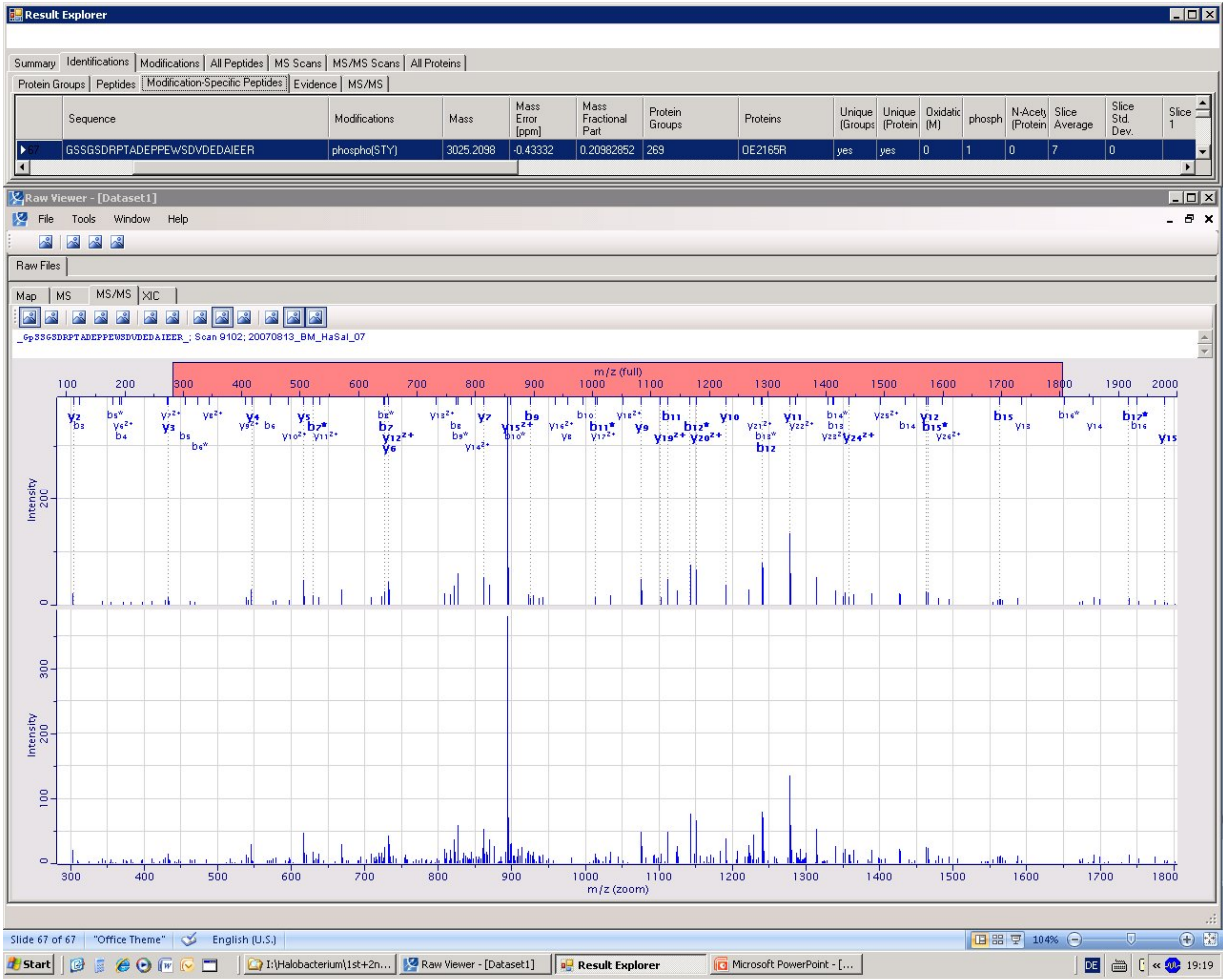
Result Explorer
Summary
Identifications
Modifications
All Peptides
MS Scans
MS/MS Scans
All Proteins
Protein Groups
Peptides
Modification-Specific Peptides
Evidence
MS/MS
Sequence
Modifications
Mass
Mass Error [ppm]
Mass Fractional Part
Protein Groups
Proteins
Unique (Groups
Unique (Protein
Oxidatic (M)
phosph
N-Acety (Protein
Slice Average
Slice Std. Dev.
Slice 1
GSSGSDRPTADEPPEWSDVDEDAIEER
phospho(STY)
3025.2098
-0.43332
0.20982852
269
OE2165R
yes
yes
0
1
0
7
0
Raw Viewer - [Dataset1]
File
Tools
Window
Help
Raw Files
Map
MS
MS/MS
XIC
GpSSGSDRPTADEPPEWSDVDEDAIEER; Scan 9102; 20070813_BM_HaSal_07
m/z (full)
Intensity
m/z (zoom)
Slide 67 of 67
"Office Theme"
English (U.S.)
104%
Start
I:\Halobacterium\1st+2n...
Raw Viewer - [Dataset1]
Result Explorer
Microsoft PowerPoint - [...
DE
19:19

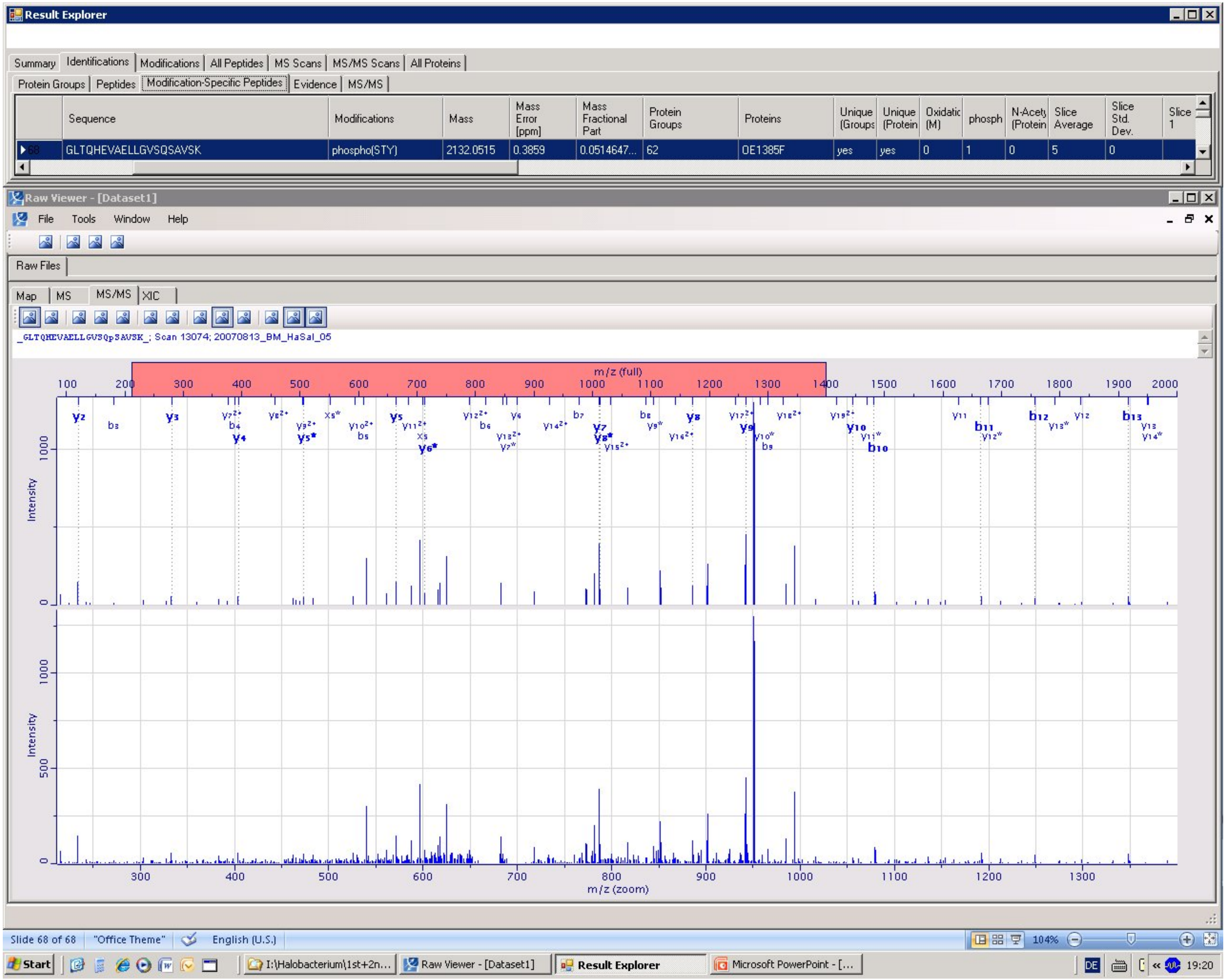
Result Explorer
Summary | Identifications | Modifications | All Peptides | MS Scans | MS/MS Scans | All Proteins
Protein Groups | Peptides | Modification-Specific Peptides | Evidence | MS/MS
Sequence | Modifications | Mass | Mass Error [ppm] | Mass Fractional Part | Protein Groups | Proteins | Unique (Groups) | Unique (Protein) | Oxidation (M) | phosph | N-Acety (Protein) | Slice Average | Slice Std. Dev. | Slice 1
GLTQHEVAELLGVSQSAVSK | phospho(STY) | 2132.0515 | 0.3859 | 0.0514647... | 62 | OE1385F | yes | yes | 0 | 1 | 0 | 5 | 0
Raw Viewer - [Dataset1]
File Tools Window Help
Raw Files
Map MS MS/MS XIC
GLTQHEVAELLGVSQpSAVSK; Scan 13074; 20070813_BM_HaSal_05
m/z (full)
Intensity
m/z (zoom)
Slide 68 of 68 "Office Theme" English (U.S.)
Start I:\Halobacterium\1st+2n... Raw Viewer - [Dataset1] Result Explorer Microsoft PowerPoint - [... 19:20

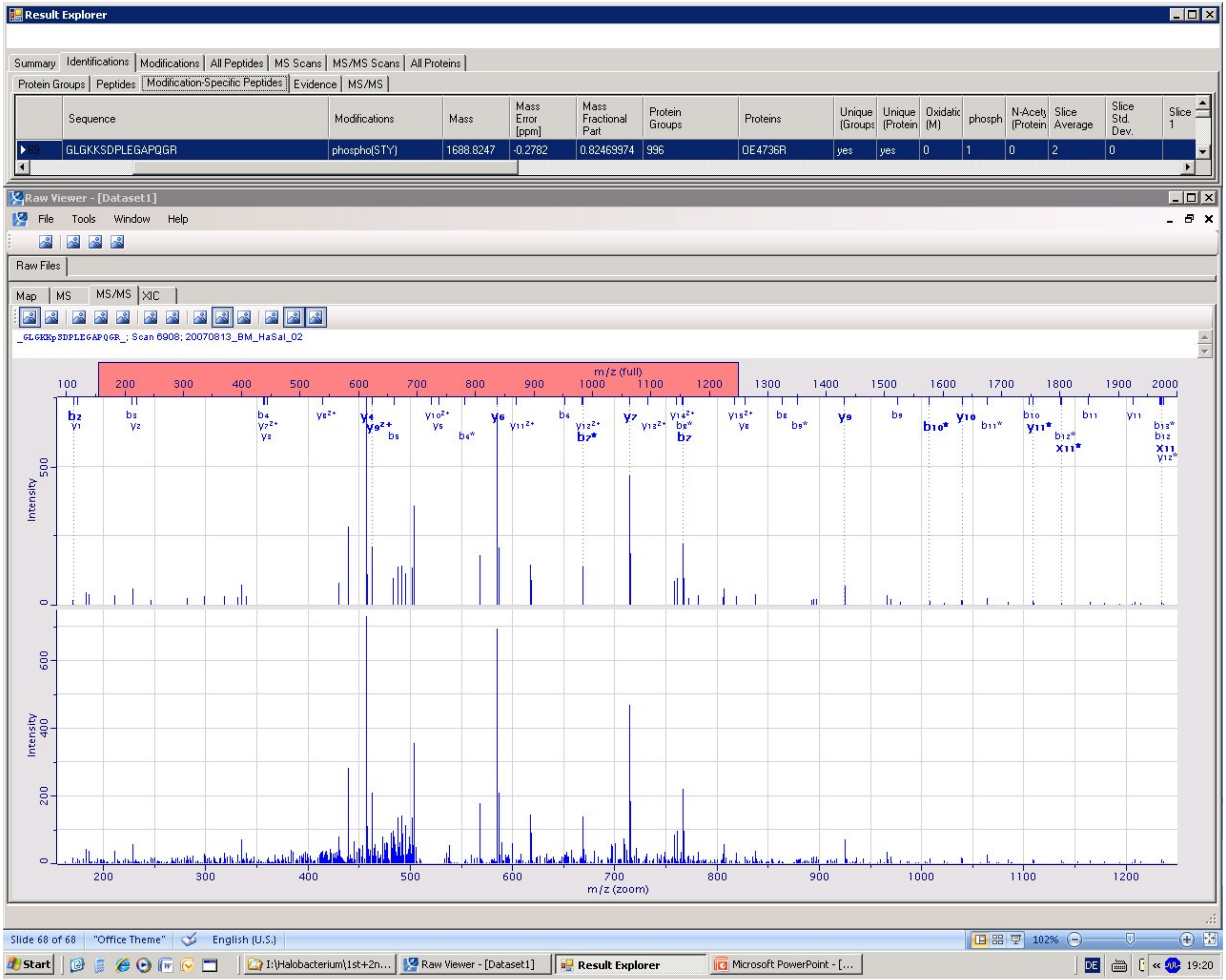
Result Explorer
Summary
Identifications
Modifications
All Peptides
MS Scans
MS/MS Scans
All Proteins
Protein Groups
Peptides
Modification-Specific Peptides
Evidence
MS/MS
Sequence
Modifications
Mass
Mass Error [ppm]
Mass Fractional Part
Protein Groups
Proteins
Unique (Groups
Unique (Protein
Oxidatic (M)
phosph
N-Acety (Protein
Slice Average
Slice Std. Dev.
Slice 1
GLGKKSDPLEGAPQGR
phospho(STY)
1688.8247
-0.2782
0.82469974
996
OE4736R
yes
yes
0
1
0
2
0
Raw Viewer - [Dataset1]
File
Tools
Window
Help
Raw Files
Map
MS
MS/MS
XIC
GLGKKpSDPLEGAPQGR; Scan 6908; 20070813_BM_HaSal_02
m/z (full)
Intensity
m/z (zoom)
Slide 68 of 68
"Office Theme"
English (U.S.)
102%
Start
I:\Halobacterium\1st+2n...
Raw Viewer - [Dataset1]
Result Explorer
Microsoft PowerPoint - [...
19:20

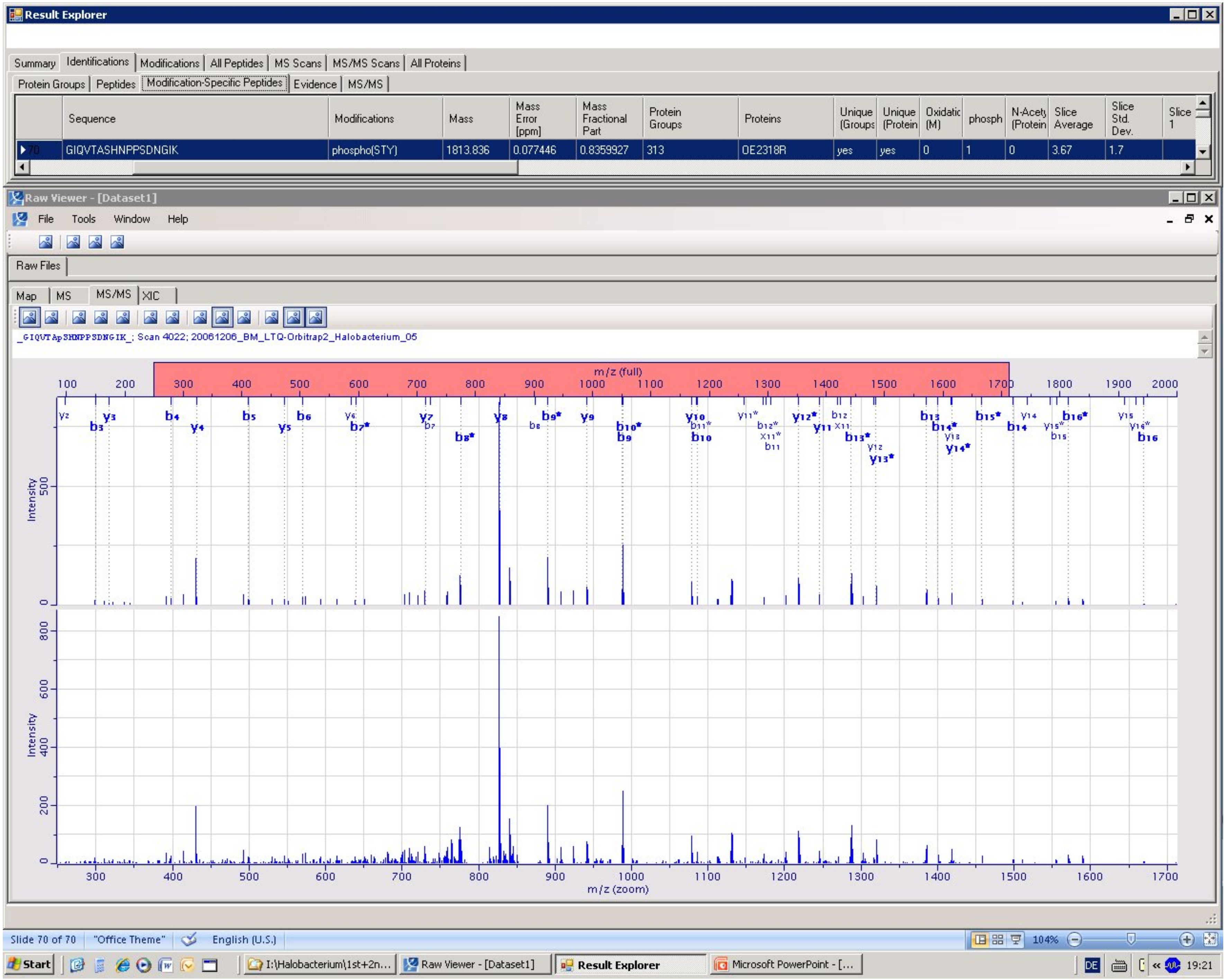
Result Explorer
Summary
Identifications
Modifications
All Peptides
MS Scans
MS/MS Scans
All Proteins
Protein Groups
Peptides
Modification-Specific Peptides
Evidence
MS/MS
Sequence
Modifications
Mass
Mass Error [ppm]
Mass Fractional Part
Protein Groups
Proteins
GIQVTASHNPPSDNGIK
phospho(STY)
1813.836
0.077446
0.8359927
313
OE2318R
yes
yes
3.67
1.7
Raw Viewer - [Dataset1]
File
Tools
Window
Help
Raw Files
Map
MS
MS/MS
XIC
Scan 4022; 20061206_BM_LTQ-Orbitrap2_Halobacterium_05
m/z (full)
Intensity
m/z (zoom)
Slide 70 of 70
"Office Theme"
English (U.S.)
104%
Start
Raw Viewer - [Dataset1]
Result Explorer
19:21

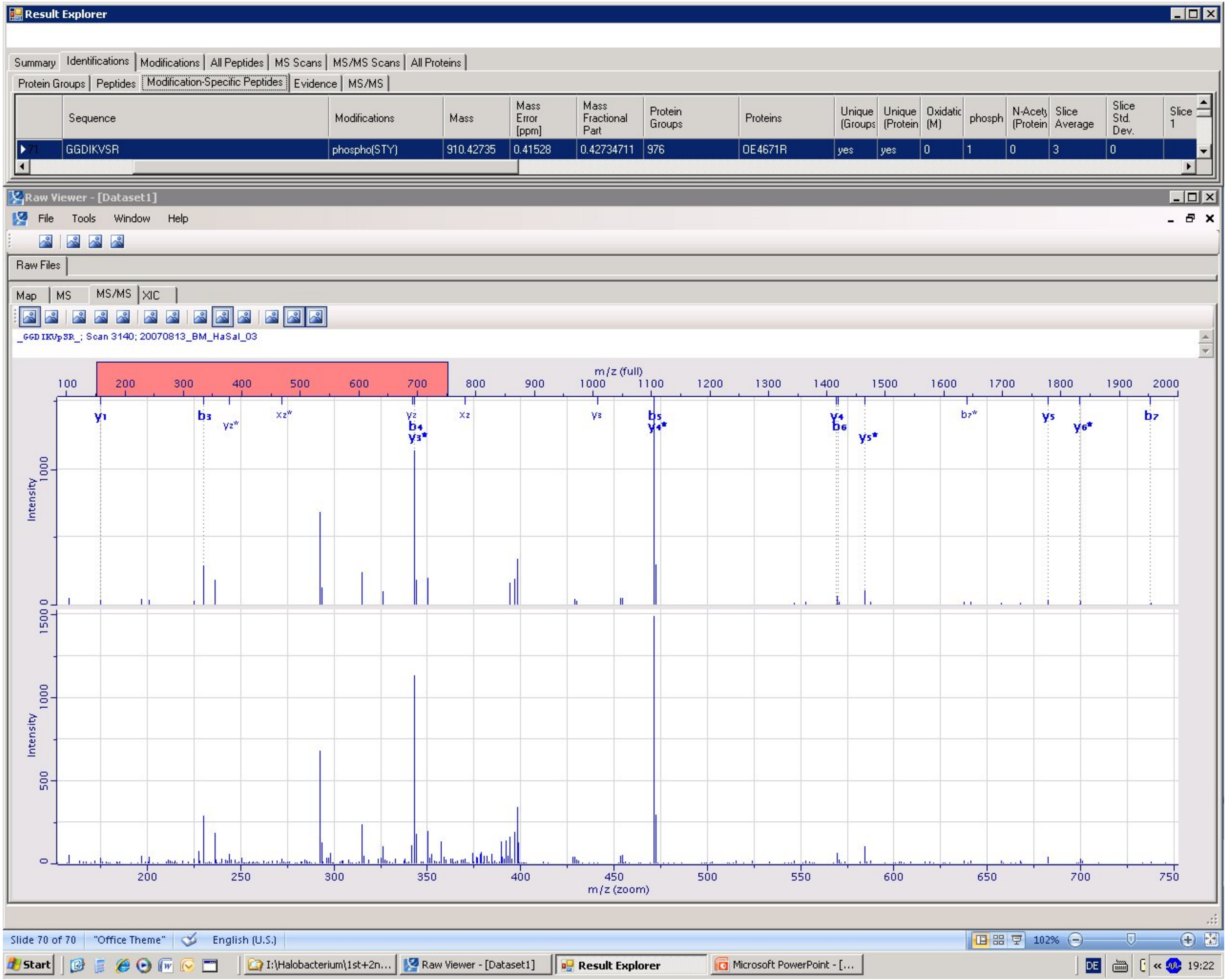
Result Explorer
Summary | Identifications | Modifications | All Peptides | MS Scans | MS/MS Scans | All Proteins
Protein Groups | Peptides | Modification-Specific Peptides | Evidence | MS/MS
Sequence | Modifications | Mass | Mass Error [ppm] | Mass Fractional Part | Protein Groups | Proteins | Unique (Groups | Unique (Protein | Oxidatic (M) | phosph | N-Acety (Protein | Slice Average | Slice Std. Dev. | Slice 1
GGDIKVSR | phospho(STY) | 910.42735 | 0.41528 | 0.42734711 | 976 | OE4671R | yes | yes | 0 | 1 | 0 | 3 | 0
Raw Viewer - [Dataset1]
File Tools Window Help
Raw Files
Map MS MS/MS XIC
GGDIKVpSR; Scan 3140; 20070813_BM_HaSal_03
m/z (full)
Intensity
m/z (zoom)
Slide 70 of 70 "Office Theme" English (U.S.)
Start
I:\Halobacterium\1st+2n...
Raw Viewer - [Dataset1]
Result Explorer
Microsoft PowerPoint - [...
19:22

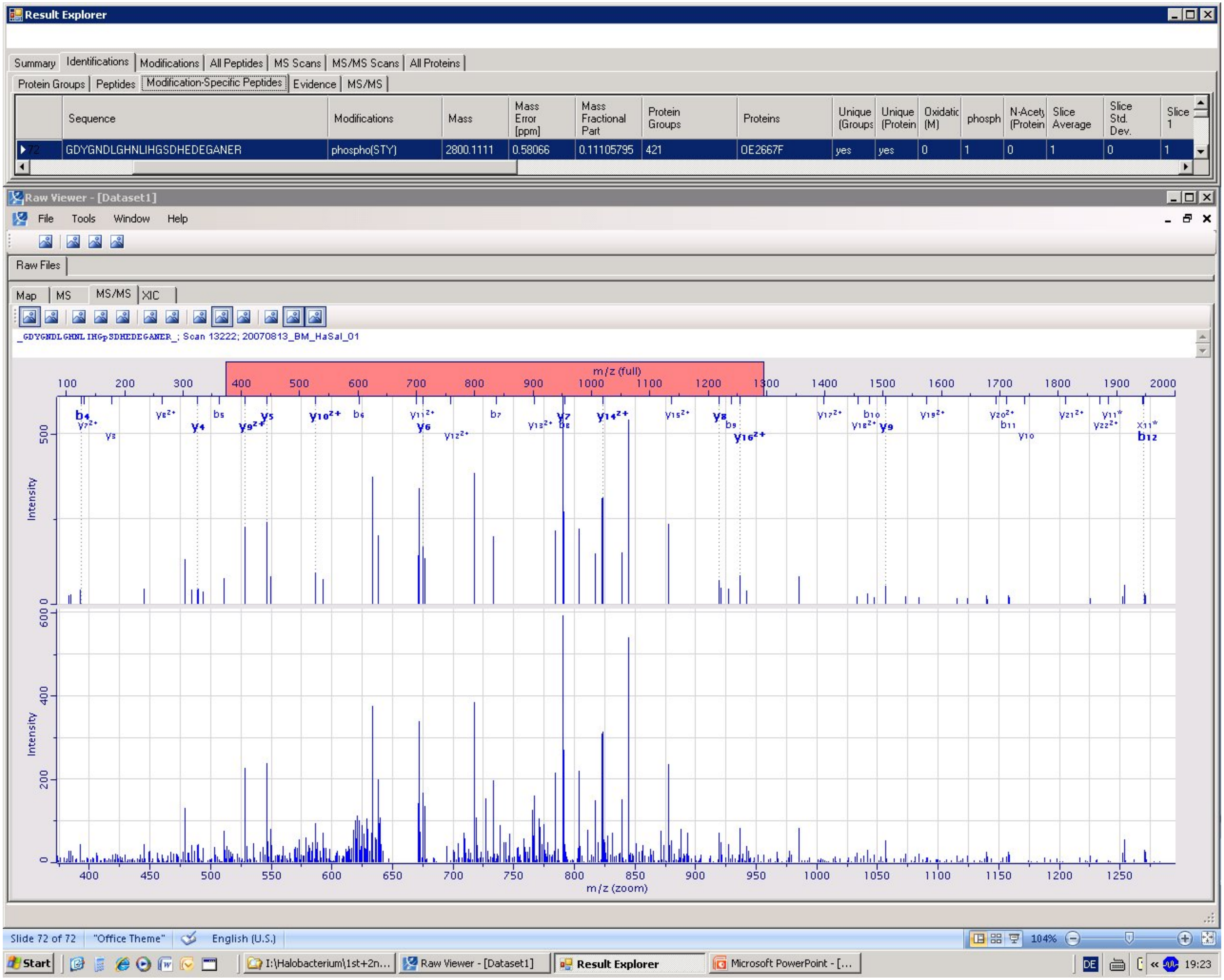
Result Explorer
Summary
Identifications
Modifications
All Peptides
MS Scans
MS/MS Scans
All Proteins
Protein Groups
Peptides
Modification-Specific Peptides
Evidence
MS/MS
Sequence
Modifications
Mass
Mass Error [ppm]
Mass Fractional Part
Protein Groups
Proteins
Unique (Groups
Unique (Protein
Oxidatic (M)
phosph
N-Acety (Protein
Slice Average
Slice Std. Dev.
Slice 1
GDYGNDLGHNLIHGSDHEDEGANER
phospho(STY)
2800.1111
0.58066
0.11105795
421
OE2667F
yes
yes
0
1
0
1
0
1
Raw Viewer - [Dataset1]
File
Tools
Window
Help
Raw Files
Map
MS
MS/MS
XIC
GDYGNDLGHNLIHGpSDHEDEGANER; Scan 13222; 20070813_BM_HaSal_01
m/z (full)
Intensity
m/z (zoom)
Slide 72 of 72
"Office Theme"
English (U.S.)
104%
Start
I:\Halobacterium\1st+2n...
Raw Viewer - [Dataset1]
Result Explorer
Microsoft PowerPoint - [...
DE
19:23

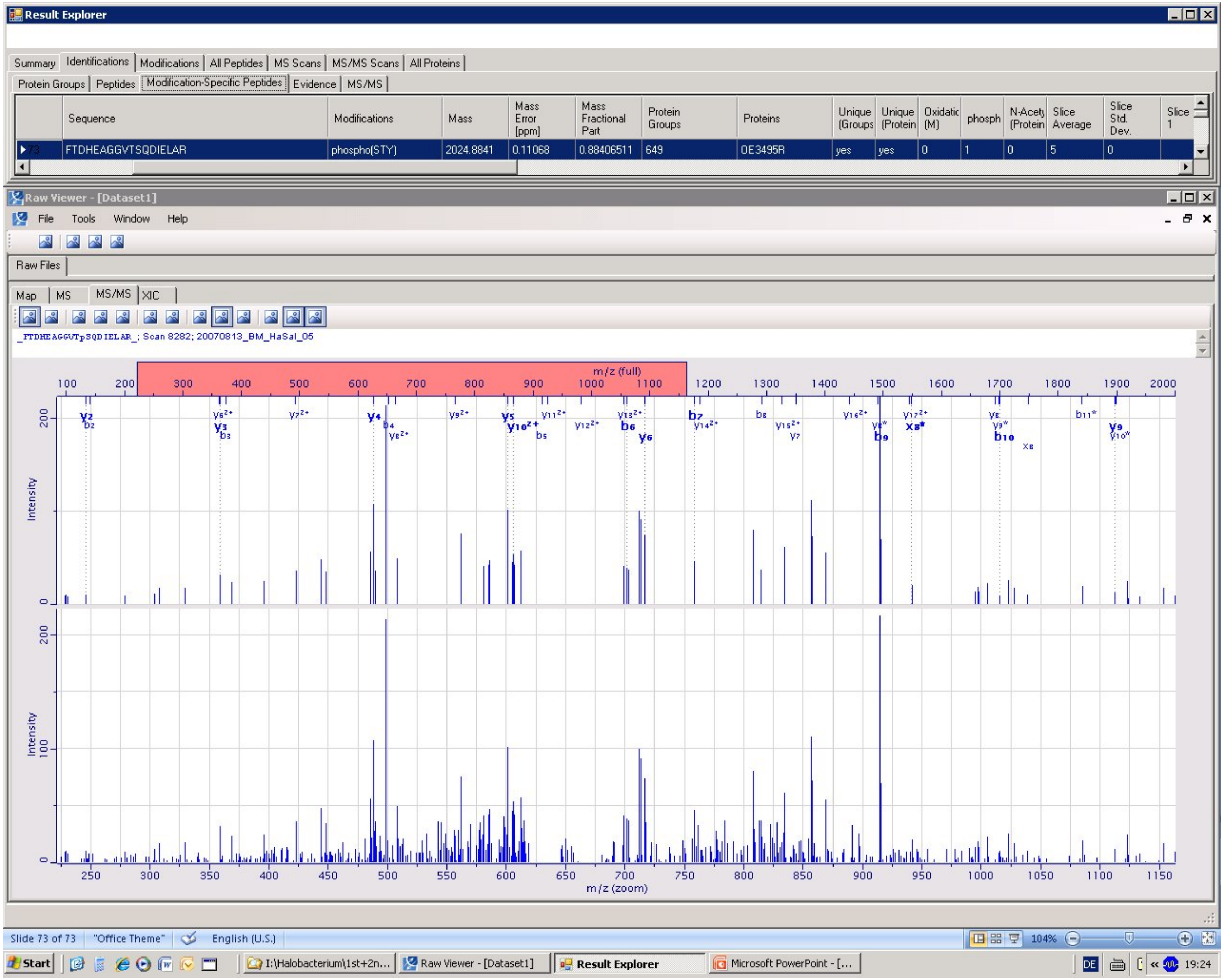
Result Explorer
Summary | Identifications | Modifications | All Peptides | MS Scans | MS/MS Scans | All Proteins
Protein Groups | Peptides | Modification-Specific Peptides | Evidence | MS/MS
Sequence | Modifications | Mass | Mass Error [ppm] | Mass Fractional Part | Protein Groups | Proteins | Unique (Groups | Unique (Protein | Oxidatic (M) | phosph | N-Acety (Protein | Slice Average | Slice Std. Dev. | Slice 1
FTDHEAGGVTSQDIELAR | phospho(STY) | 2024.8841 | 0.11068 | 0.88406511 | 649 | OE3495R | yes | yes | 0 | 1 | 0 | 5 | 0
Raw Viewer - [Dataset1]
File Tools Window Help
Raw Files
Map MS MS/MS XIC
FTDHEAGGVTpSQDIELAR; Scan 8282; 20070813_BM_HaSal_05
m/z (full)
Intensity
m/z (zoom)
Slide 73 of 73 "Office Theme" English (U.S.)
Start
I:\Halobacterium\1st+2n...
Raw Viewer - [Dataset1]
Result Explorer
Microsoft PowerPoint - [...
19:24

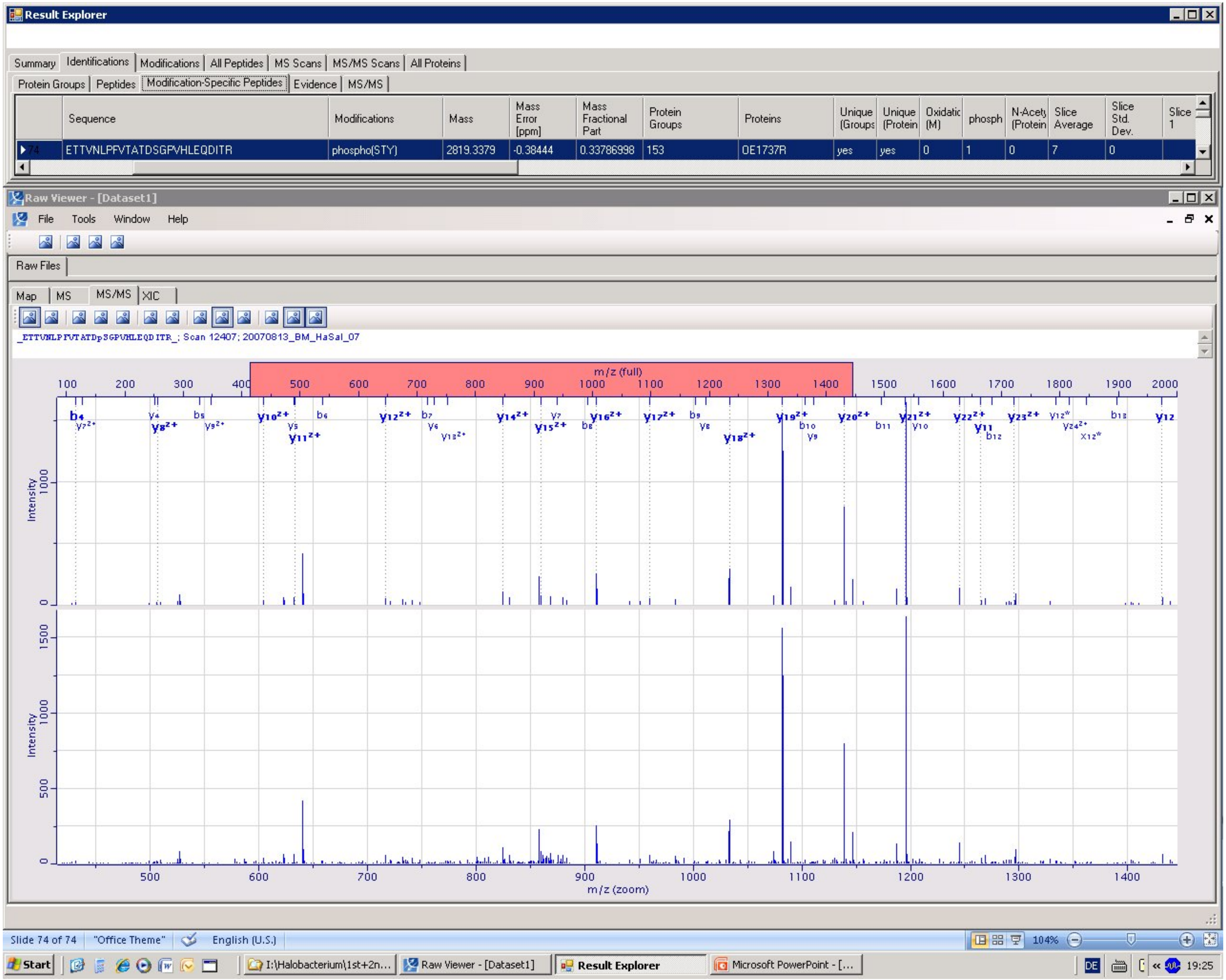
Result Explorer
Summary
Identifications
Modifications
All Peptides
MS Scans
MS/MS Scans
All Proteins
Protein Groups
Peptides
Modification-Specific Peptides
Evidence
MS/MS
Sequence
Modifications
Mass
Mass Error [ppm]
Mass Fractional Part
Protein Groups
Proteins
Unique (Groups
Unique (Protein
Oxidatio (M)
phosph
N-Acety (Protein
Slice Average
Slice Std. Dev.
Slice 1
ETTVNLPFVTATDSGPVHLEQDITR
phospho(STY)
2819.3379
-0.38444
0.33786998
153
OE1737R
yes
yes
0
1
0
7
0
Raw Viewer - [Dataset1]
File
Tools
Window
Help
Raw Files
Map
MS
MS/MS
XIC
ETTVNLPFVTATDpSGPVHLEQDITR; Scan 12407; 20070813_BM_HaSal_07
m/z (full)
Intensity
m/z (zoom)
Slide 74 of 74
"Office Theme"
English (U.S.)
104%
Start
I:\Halobacterium\1st+2n...
Raw Viewer - [Dataset1]
Result Explorer
Microsoft PowerPoint - [...
DE
19:25

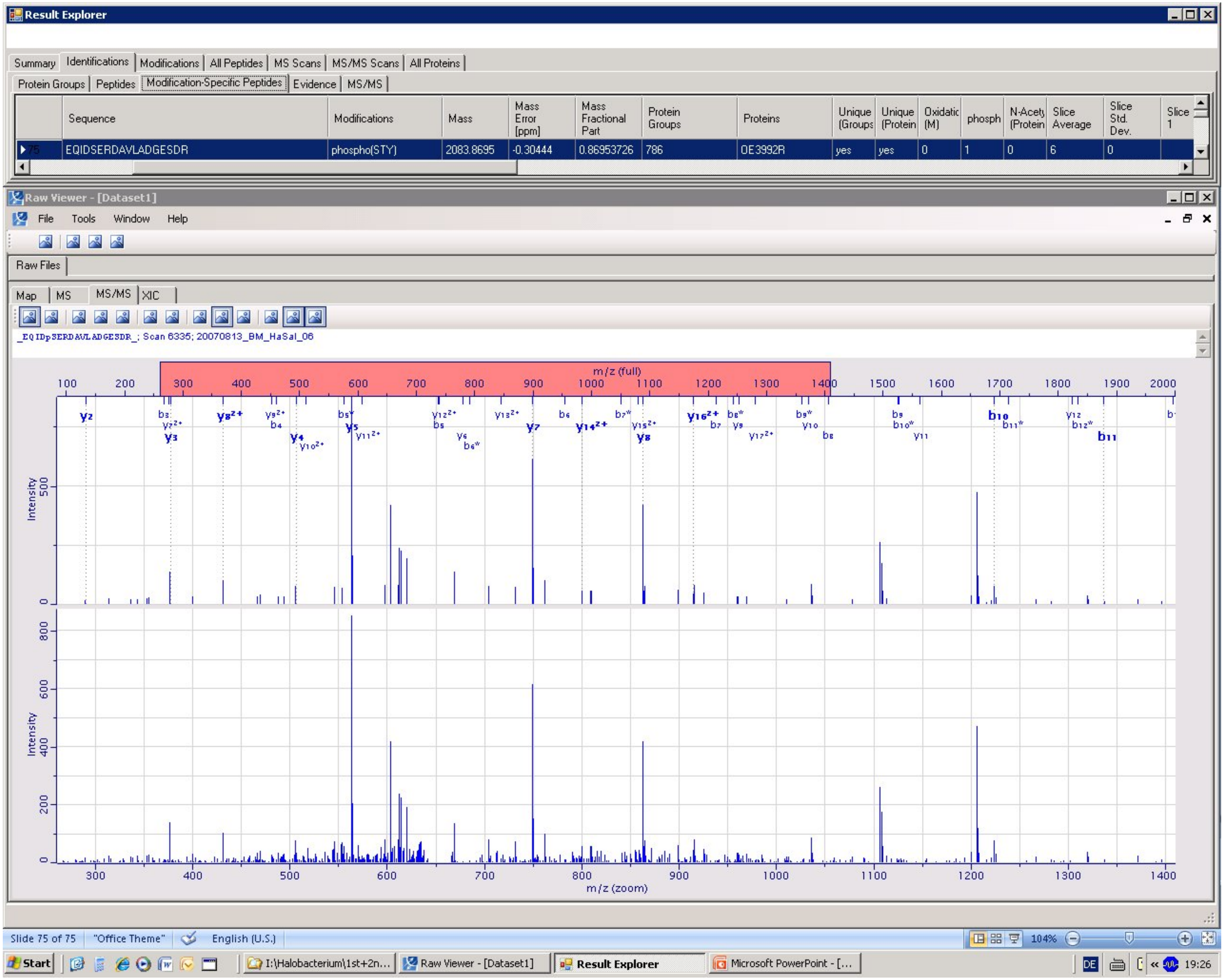
Result Explorer
Summary
Identifications
Modifications
All Peptides
MS Scans
MS/MS Scans
All Proteins
Protein Groups
Peptides
Modification-Specific Peptides
Evidence
MS/MS
Sequence
Modifications
Mass
Mass Error [ppm]
Mass Fractional Part
Protein Groups
Proteins
phosph
Slice Average
EQIDSERDAVLADGESDR
phospho(STY)
2083.8695
-0.30444
0.86953726
786
OE3992R
yes
yes
Raw Viewer - [Dataset1]
File
Tools
Window
Help
Raw Files
Map
MS
MS/MS
XIC
EQIDpSERDAVLADGESDR; Scan 6335; 20070813_BM_HaSal_06
m/z (full)
Intensity
m/z (zoom)
Slide 75 of 75
"Office Theme"
English (U.S.)
Start
Raw Viewer - [Dataset1]
Result Explorer
19:26

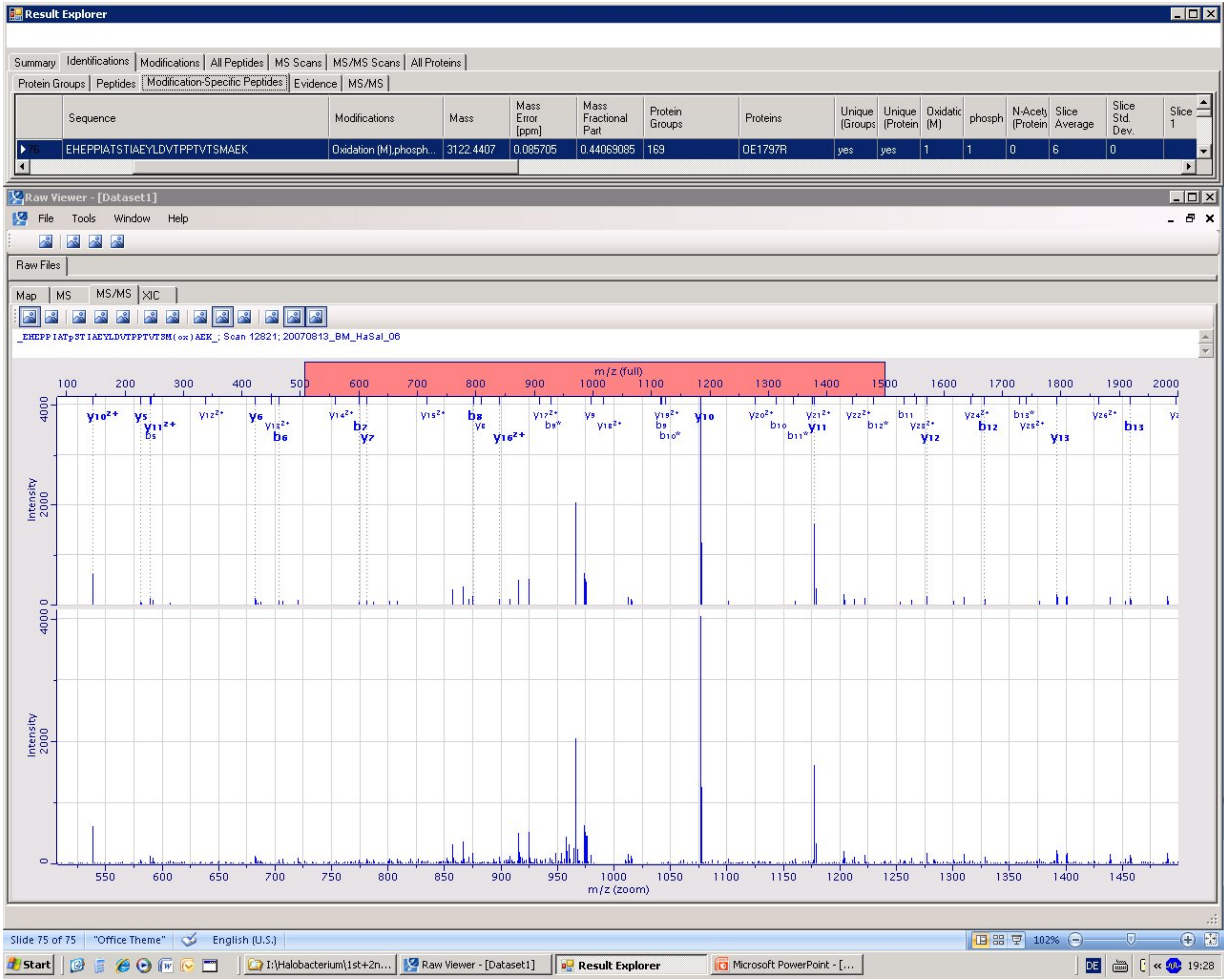
Result Explorer
Summary
Identifications
Modifications
All Peptides
MS Scans
MS/MS Scans
All Proteins
Protein Groups
Peptides
Modification-Specific Peptides
Evidence
MS/MS
Sequence
Modifications
Mass
Mass Error [ppm]
Mass Fractional Part
Protein Groups
Proteins
EHEPPIATSTIAEYLDVTPPTVTSMAEK
Oxidation (M),phosph...
3122.4407
0.085705
0.44069085
169
OE1797R
yes
yes
Raw Viewer - [Dataset1]
File
Tools
Window
Help
Raw Files
Map
MS
MS/MS
XIC
EHEPPIATpSTIAEYLDVTPPTVTSM(ox)AEK; Scan 12821; 20070813_BM_HaSal_06
m/z (full)
Intensity
m/z (zoom)
Slide 75 of 75
"Office Theme"
English (U.S.)
Start
Raw Viewer - [Dataset1]
Result Explorer
Microsoft PowerPoint - [...
19:28

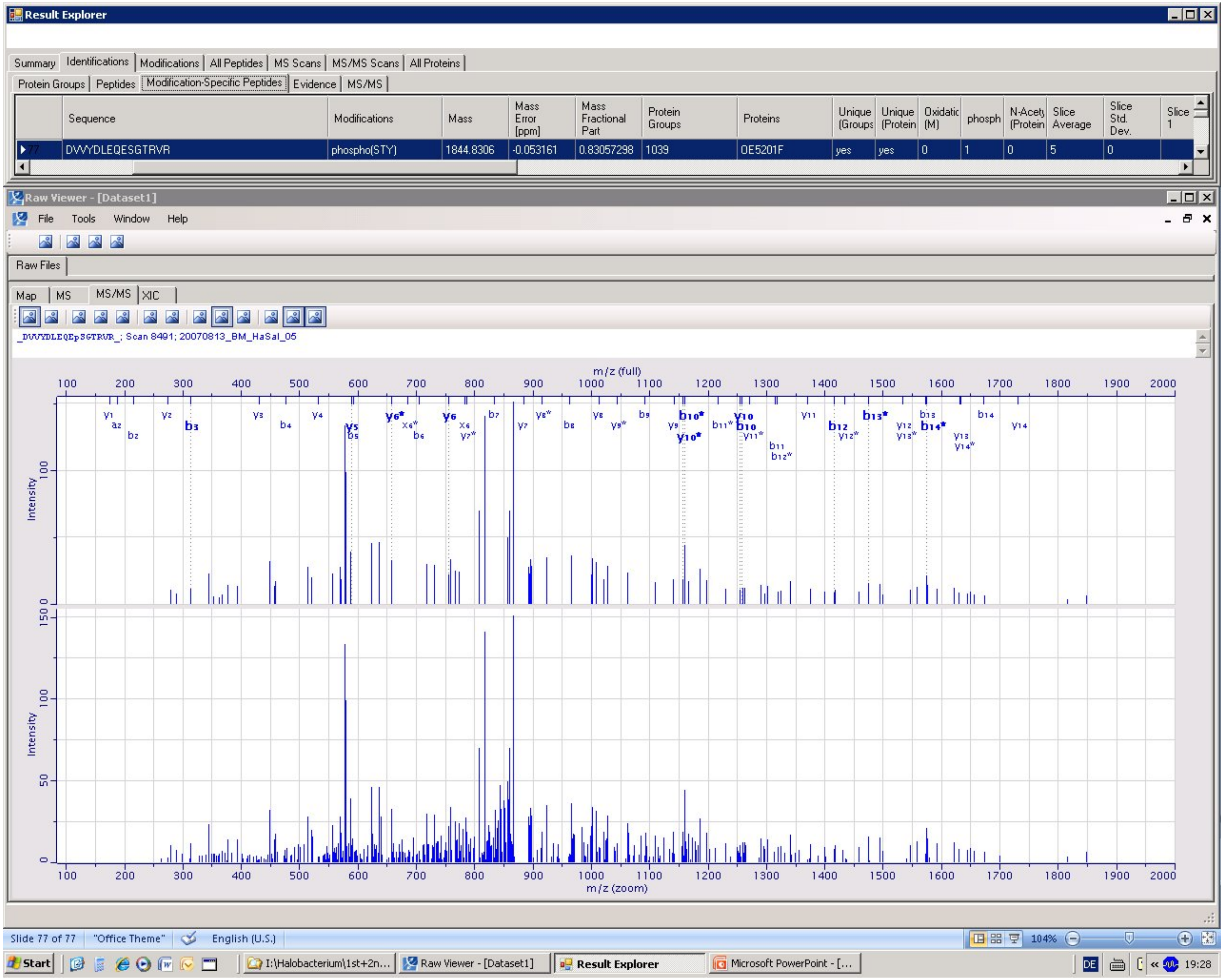
Result Explorer
Summary
Identifications
Modifications
All Peptides
MS Scans
MS/MS Scans
All Proteins
Protein Groups
Peptides
Modification-Specific Peptides
Evidence
MS/MS
Sequence
Modifications
Mass
Mass Error [ppm]
Mass Fractional Part
Protein Groups
Proteins
Unique (Groups
Unique (Protein
Oxidatic (M)
phosph
N-Acety (Protein
Slice Average
Slice Std. Dev.
Slice 1
DVVYDLEQESGTRVR
phospho(STY)
1844.8306
-0.053161
0.83057298
1039
OE5201F
yes
yes
0
1
0
5
0
Raw Viewer - [Dataset1]
File
Tools
Window
Help
Raw Files
Map
MS
MS/MS
XIC
DVVYDLEQEpSGTRVR; Scan 8491; 20070813_BM_HaSal_05
m/z (full)
Intensity
m/z (zoom)
Slide 77 of 77
"Office Theme"
English (U.S.)
104%
Start
I:\Halobacterium\1st+2n...
Raw Viewer - [Dataset1]
Result Explorer
Microsoft PowerPoint - [...
DE
19:28

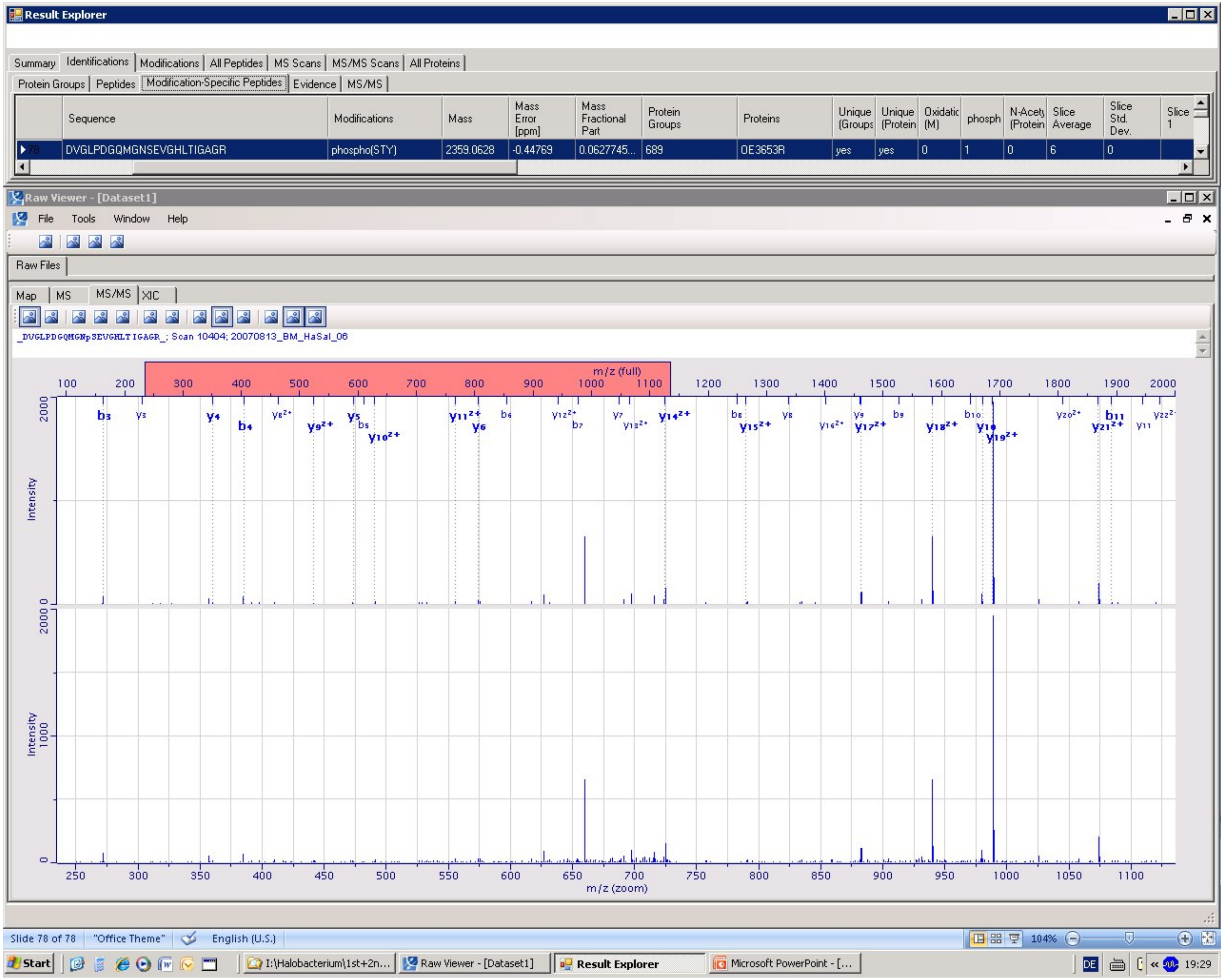
Result Explorer
Summary
Identifications
Modifications
All Peptides
MS Scans
MS/MS Scans
All Proteins
Protein Groups
Peptides
Modification-Specific Peptides
Evidence
MS/MS
Sequence
Modifications
Mass
Mass Error [ppm]
Mass Fractional Part
Protein Groups
Proteins
DVGLPDGQMGNSEVGHLTIGAGR
phospho(STY)
2359.0628
-0.44769
0.0627745...
689
OE3653R
yes
yes
Raw Viewer - [Dataset1]
File
Tools
Window
Help
Raw Files
Map
MS
MS/MS
XIC
DVGLPDGQMGNpSEVGHLTIGAGR; Scan 10404; 20070813_BM_HaSal_06
m/z (full)
Intensity
m/z (zoom)
Slide 78 of 78
"Office Theme"
English (U.S.)
Start
Raw Viewer - [Dataset1]
Result Explorer
19:29

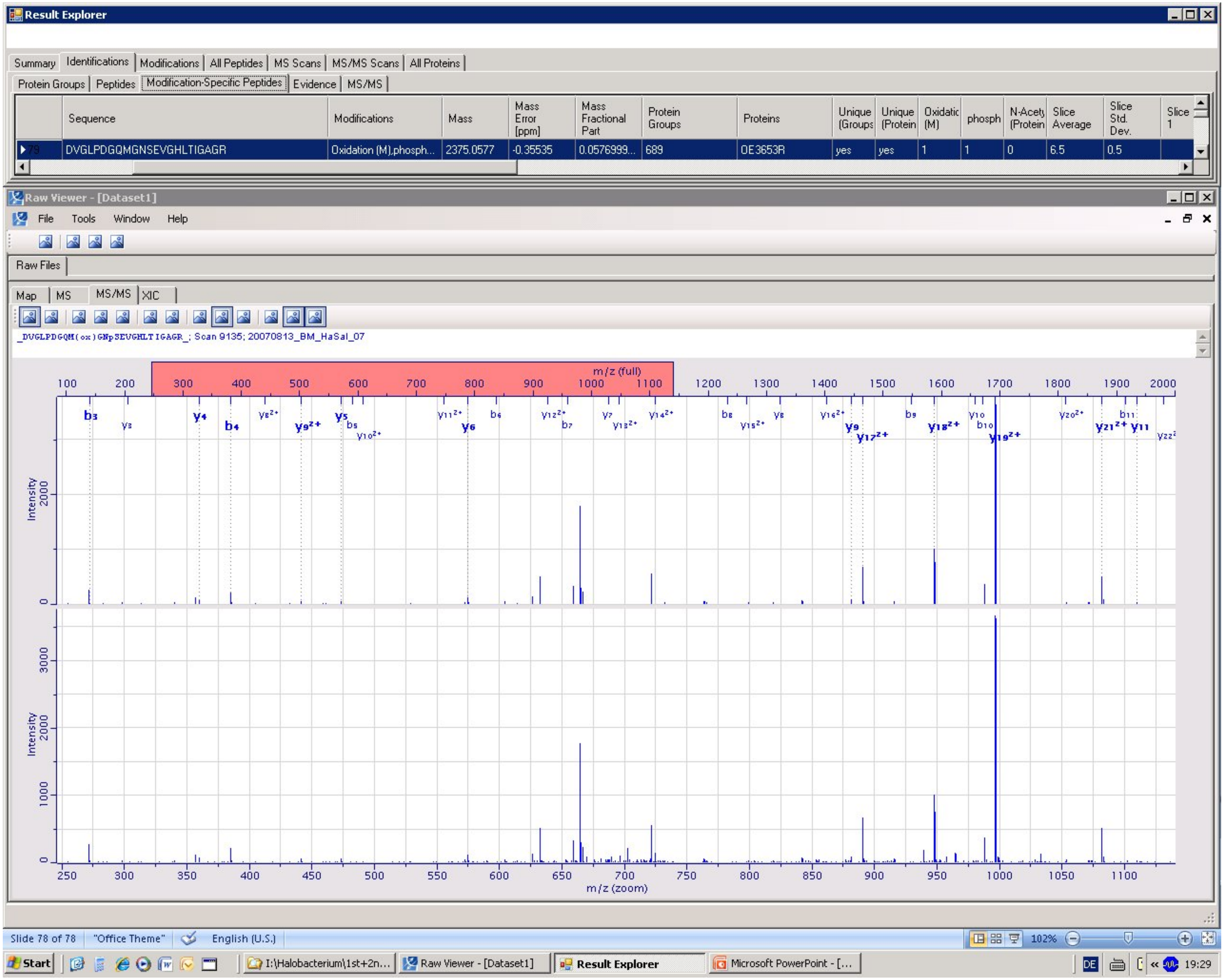
Result Explorer
Summary | Identifications | Modifications | All Peptides | MS Scans | MS/MS Scans | All Proteins
Protein Groups | Peptides | Modification-Specific Peptides | Evidence | MS/MS
Sequence | Modifications | Mass | Mass Error [ppm] | Mass Fractional Part | Protein Groups | Proteins | Unique (Groups | Unique (Protein | Oxidatic (M) | phosph | N-Acety (Protein | Slice Average | Slice Std. Dev. | Slice 1
DVGLPDGQMGNSEVGHLTIGAGR | Oxidation (M),phosph... | 2375.0577 | -0.35535 | 0.0576999... | 689 | OE3653R | yes | yes | 1 | 1 | 0 | 6.5 | 0.5
Raw Viewer - [Dataset1]
File Tools Window Help
Raw Files
Map MS MS/MS XIC
DVGLPDGQM(ox)GNpSEVGHLTIGAGR; Scan 9135; 20070813_BM_HaSal_07
m/z (full)
Intensity
m/z (zoom)
Slide 78 of 78 "Office Theme" English (U.S.)
102%
Start
I:\Halobacterium\1st+2n...
Raw Viewer - [Dataset1]
Result Explorer
Microsoft PowerPoint - [...
DE
19:29

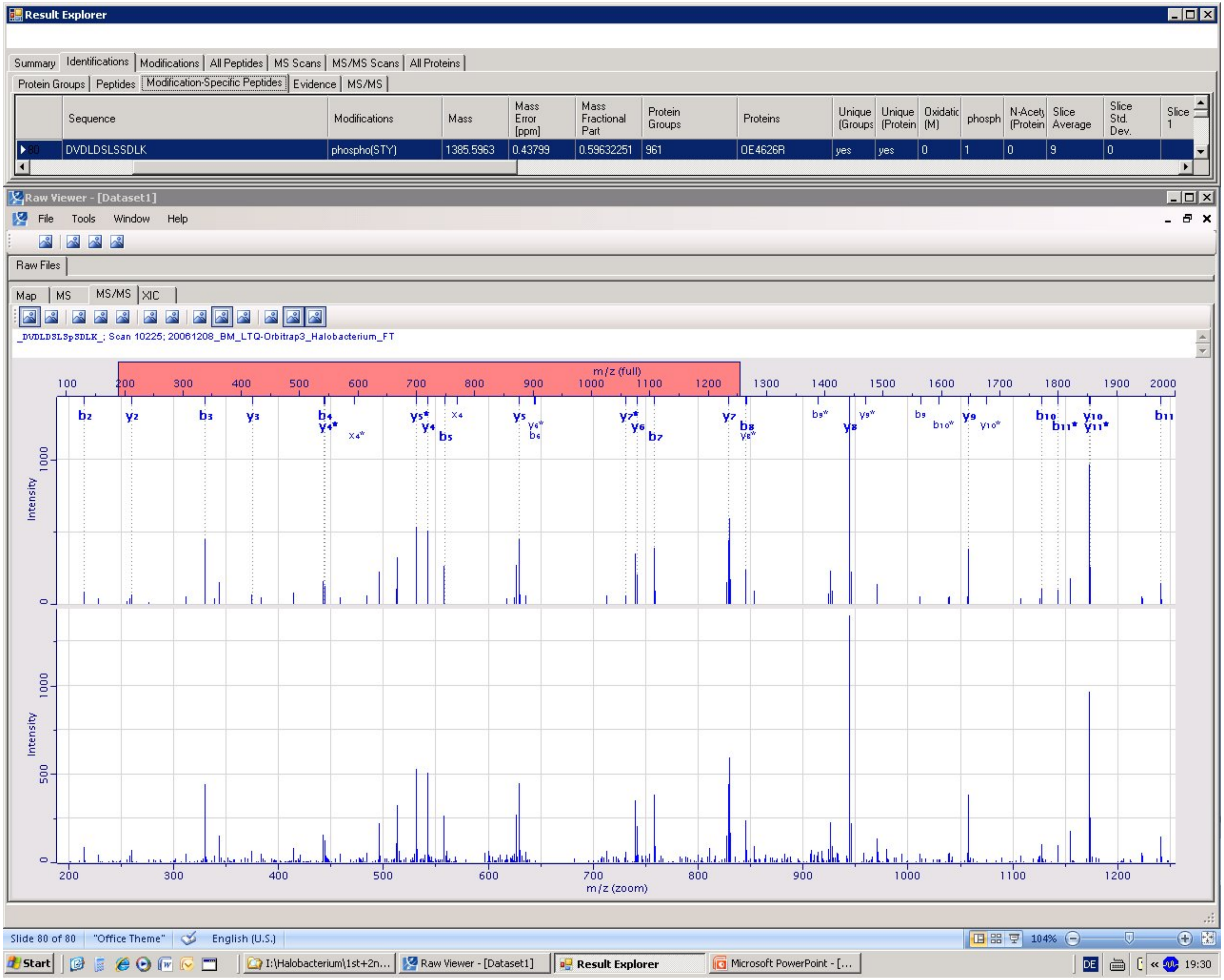
Result Explorer
Summary
Identifications
Modifications
All Peptides
MS Scans
MS/MS Scans
All Proteins
Protein Groups
Peptides
Modification-Specific Peptides
Evidence
MS/MS
Sequence
Modifications
Mass
Mass Error [ppm]
Mass Fractional Part
Protein Groups
Proteins
Unique (Groups
Unique (Protein
Oxidatio (M)
phosph
N-Acety (Protein
Slice Average
Slice Std. Dev.
Slice 1
DVDLDSLSSDLK
phospho(STY)
1385.5963
0.43799
0.59632251
961
OE4626R
yes
yes
0
1
0
9
0
Raw Viewer - [Dataset1]
File
Tools
Window
Help
Raw Files
Map
MS
MS/MS
XIC
DVDLDSLSpSDLK; Scan 10225; 20061208_BM_LTQ-Orbitrap3_Halobacterium_FT
m/z (full)
Intensity
m/z (zoom)
Slide 80 of 80
"Office Theme"
English (U.S.)
104%
Start
I:\Halobacterium\1st+2n...
Raw Viewer - [Dataset1]
Result Explorer
Microsoft PowerPoint - [...
DE
19:30

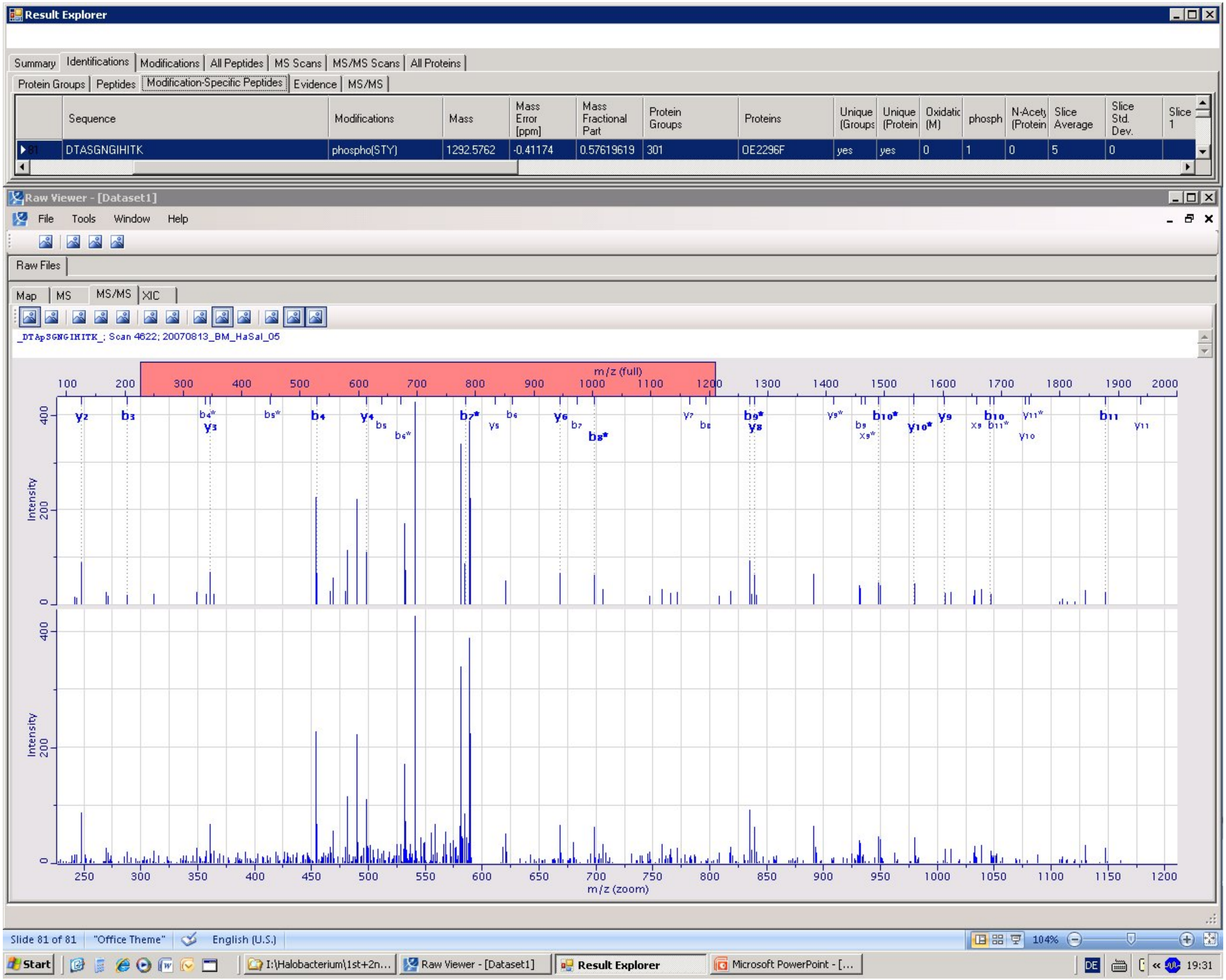
Result Explorer
Summary | Identifications | Modifications | All Peptides | MS Scans | MS/MS Scans | All Proteins
Protein Groups | Peptides | Modification-Specific Peptides | Evidence | MS/MS
Sequence | Modifications | Mass | Mass Error [ppm] | Mass Fractional Part | Protein Groups | Proteins | Unique (Groups | Unique (Protein | Oxidatic (M) | phosph | N-Acety (Protein | Slice Average | Slice Std. Dev. | Slice 1
DTASGNGIHITK | phospho(STY) | 1292.5762 | -0.41174 | 0.57619619 | 301 | OE2296F | yes | yes | 0 | 1 | 0 | 5 | 0
Raw Viewer - [Dataset1]
File Tools Window Help
Raw Files
Map | MS | MS/MS | XIC
DTApSGNGIHITK; Scan 4622; 20070813_BM_HaSal_05
m/z (full)
Intensity
m/z (zoom)
Slide 81 of 81 "Office Theme" English (U.S.) 104%
Start | I:\Halobacterium\1st+2n... | Raw Viewer - [Dataset1] | Result Explorer | Microsoft PowerPoint - [... | 19:31

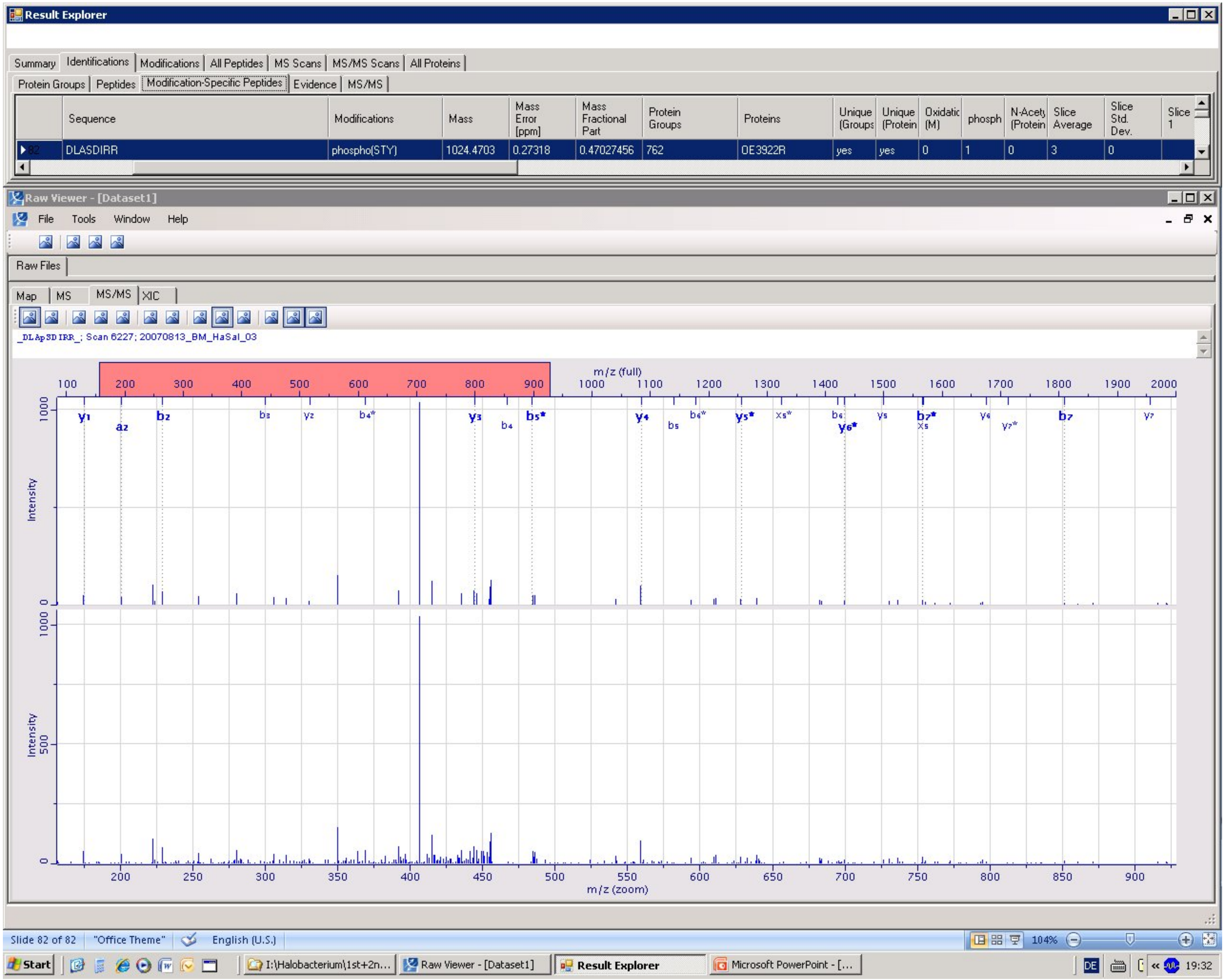
Result Explorer
Summary | Identifications | Modifications | All Peptides | MS Scans | MS/MS Scans | All Proteins
Protein Groups | Peptides | Modification-Specific Peptides | Evidence | MS/MS
Sequence | Modifications | Mass | Mass Error [ppm] | Mass Fractional Part | Protein Groups | Proteins | Unique (Groups | Unique (Protein | Oxidatic (M) | phosph | N-Acety (Protein | Slice Average | Slice Std. Dev. | Slice 1
DLASDIRR | phospho(STY) | 1024.4703 | 0.27318 | 0.47027456 | 762 | OE3922R | yes | yes | 0 | 1 | 0 | 3 | 0
Raw Viewer - [Dataset1]
File Tools Window Help
Raw Files
Map | MS | MS/MS | XIC
DLApSDIRR; Scan 6227; 20070813_BM_HaSal_03
m/z (full)
Intensity
y1 a2 b2 b3 y2 b4* y3 b4 b5* y4 b5 b6* y5* x5* b6 y6* y5 b7* x5 y6 y7* b7 y7
m/z (zoom)
Slide 82 of 82 "Office Theme" English (U.S.)
Start I:\Halobacterium\1st+2n... Raw Viewer - [Dataset1] Result Explorer Microsoft PowerPoint - [...
19:32

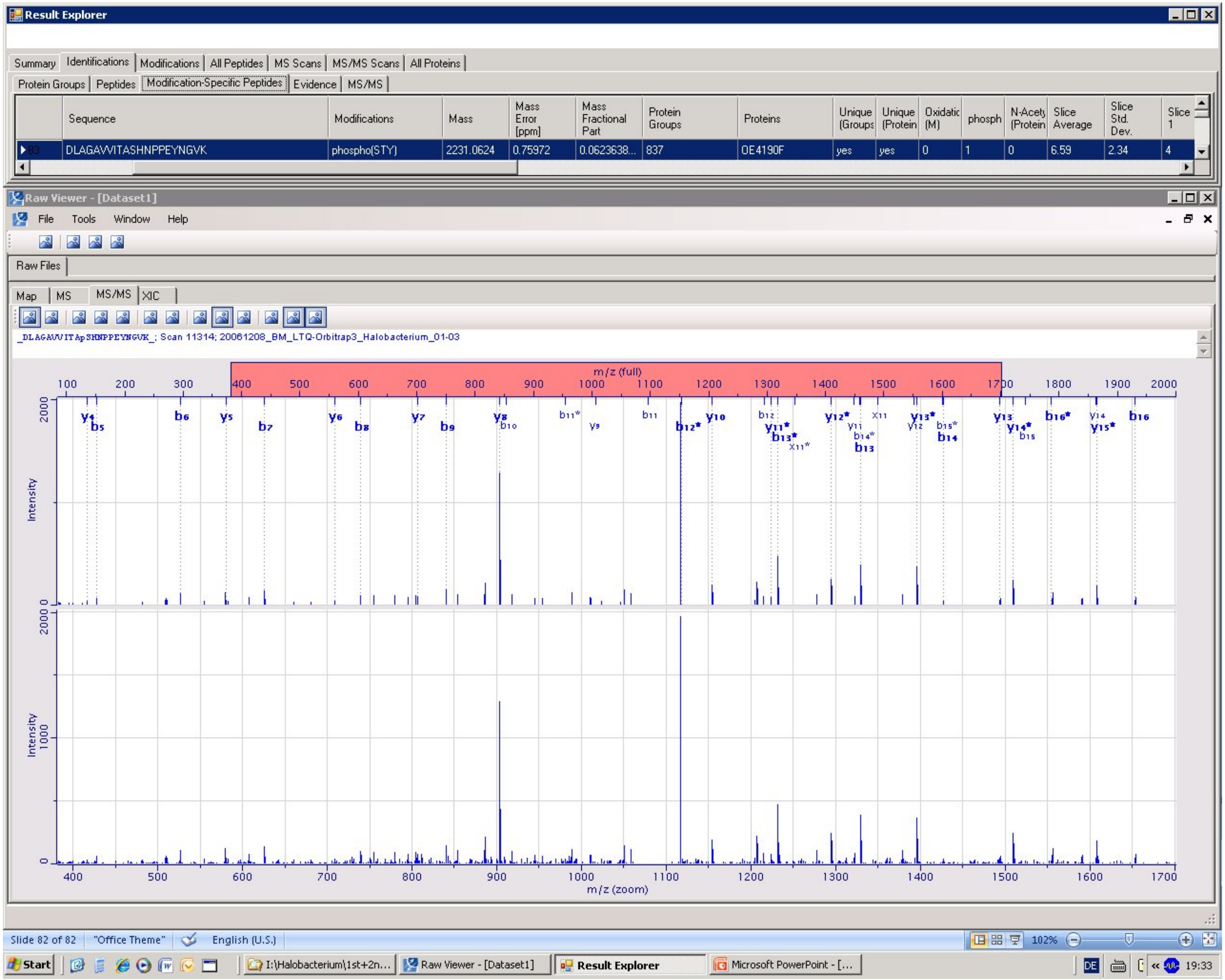
Result Explorer
Summary
Identifications
Modifications
All Peptides
MS Scans
MS/MS Scans
All Proteins
Protein Groups
Peptides
Modification-Specific Peptides
Evidence
MS/MS
Sequence
Modifications
Mass
Mass Error [ppm]
Mass Fractional Part
Protein Groups
Proteins
Unique (Groups
Unique (Protein
Oxidatic (M)
phosph
N-Acety (Protein
Slice Average
Slice Std. Dev.
Slice 1
DLAGAVVITASHNPPEYNGVK
phospho(STY)
2231.0624
0.75972
0.0623638...
837
OE4190F
yes
yes
0
1
0
6.59
2.34
4
Raw Viewer - [Dataset1]
File
Tools
Window
Help
Raw Files
Map
MS
MS/MS
XIC
DLAGAVVITApSHNPPEYNGVK; Scan 11314; 20061208_BM_LTQ-Orbitrap3_Halobacterium_01-03
m/z (full)
Intensity
m/z (zoom)
Slide 82 of 82
"Office Theme"
English (U.S.)
102%
Start
I:\Halobacterium\1st+2n...
Raw Viewer - [Dataset1]
Result Explorer
Microsoft PowerPoint - [...
DE
19:33

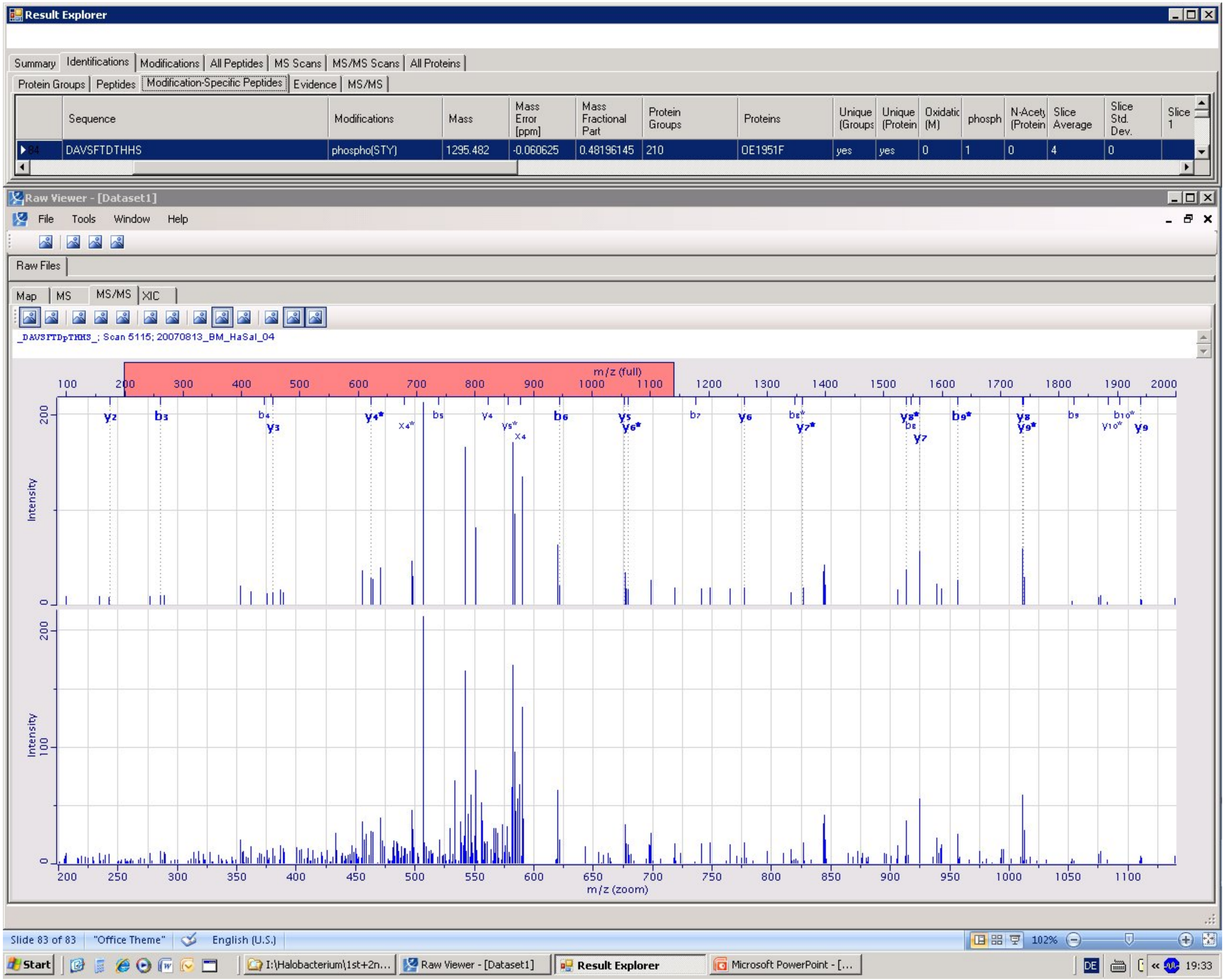
Result Explorer
Summary | Identifications | Modifications | All Peptides | MS Scans | MS/MS Scans | All Proteins
Protein Groups | Peptides | Modification-Specific Peptides | Evidence | MS/MS
Sequence | Modifications | Mass | Mass Error [ppm] | Mass Fractional Part | Protein Groups | Proteins | Unique (Groups) | Unique (Protein) | Oxidation (M) | phosph | N-Acety (Protein) | Slice Average | Slice Std. Dev. | Slice 1
DAVSFTDTHHS | phospho(STY) | 1295.482 | -0.060625 | 0.48196145 | 210 | OE1951F | yes | yes | 0 | 1 | 0 | 4 | 0
Raw Viewer - [Dataset1]
File Tools Window Help
Raw Files
Map | MS | MS/MS | XIC
DAVSFTDpTHHS; Scan 5115; 20070813_BM_HaSal_04
m/z (full)
Intensity
m/z (zoom)
Slide 83 of 83 "Office Theme" English (U.S.)
Start
Raw Viewer - [Dataset1]
Result Explorer
Microsoft PowerPoint - [...
19:33

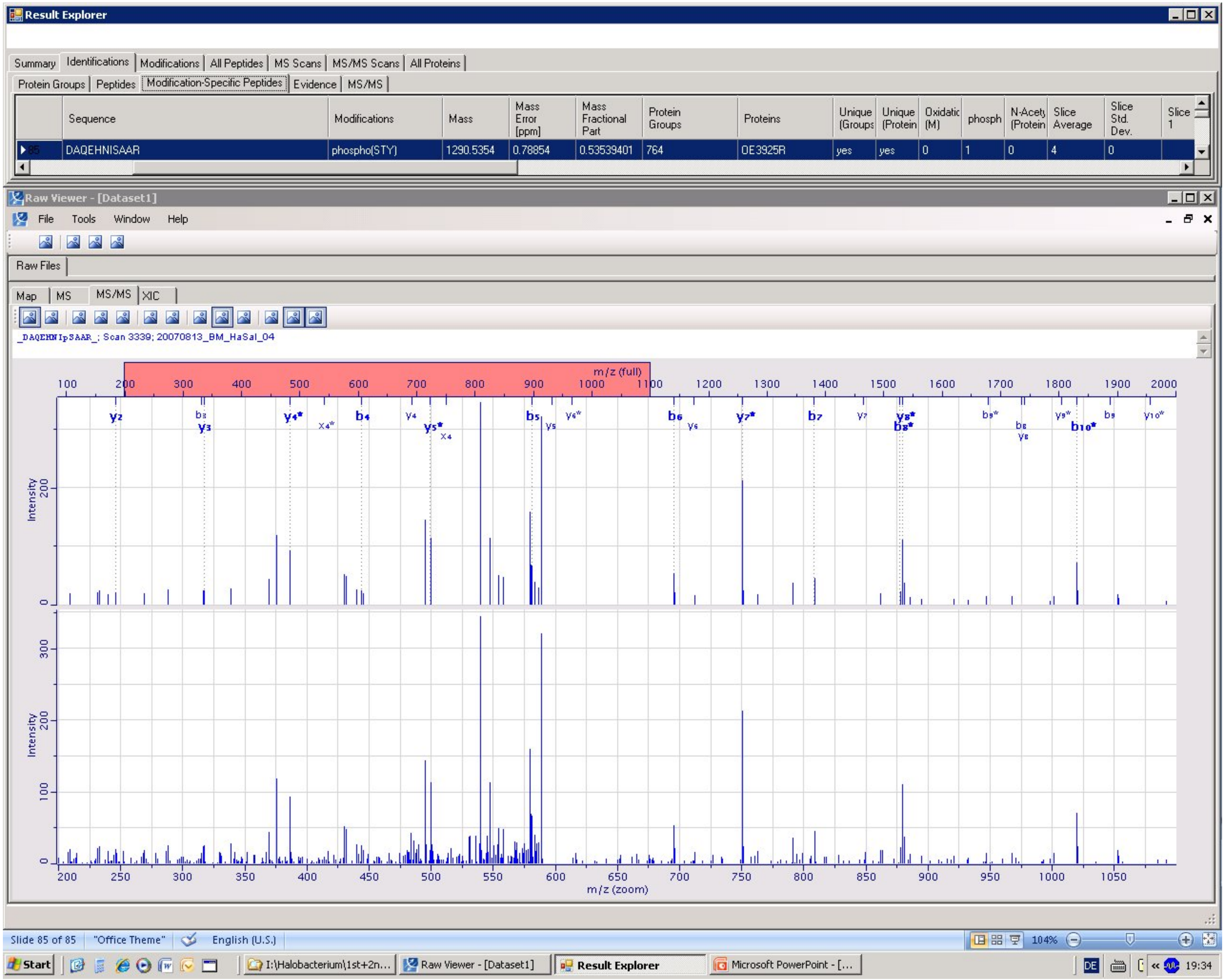
Result Explorer
Summary
Identifications
Modifications
All Peptides
MS Scans
MS/MS Scans
All Proteins
Protein Groups
Peptides
Modification-Specific Peptides
Evidence
MS/MS
Sequence
Modifications
Mass
Mass Error [ppm]
Mass Fractional Part
Protein Groups
Proteins
Unique (Groups
Unique (Protein
Oxidatic (M)
phosph
N-Acety (Protein
Slice Average
Slice Std. Dev.
Slice 1
DAQEHNISAAR
phospho(STY)
1290.5354
0.78854
0.53539401
764
OE3925R
yes
yes
0
1
0
4
0
Raw Viewer - [Dataset1]
File
Tools
Window
Help
Raw Files
Map
MS
MS/MS
XIC
DAQEHNIpSAAR; Scan 3339; 20070813_BM_HaSal_04
m/z (full)
Intensity
m/z (zoom)
Slide 85 of 85
"Office Theme"
English (U.S.)
104%
Start
I:\Halobacterium\1st+2n...
Raw Viewer - [Dataset1]
Result Explorer
Microsoft PowerPoint - [...
DE
19:34

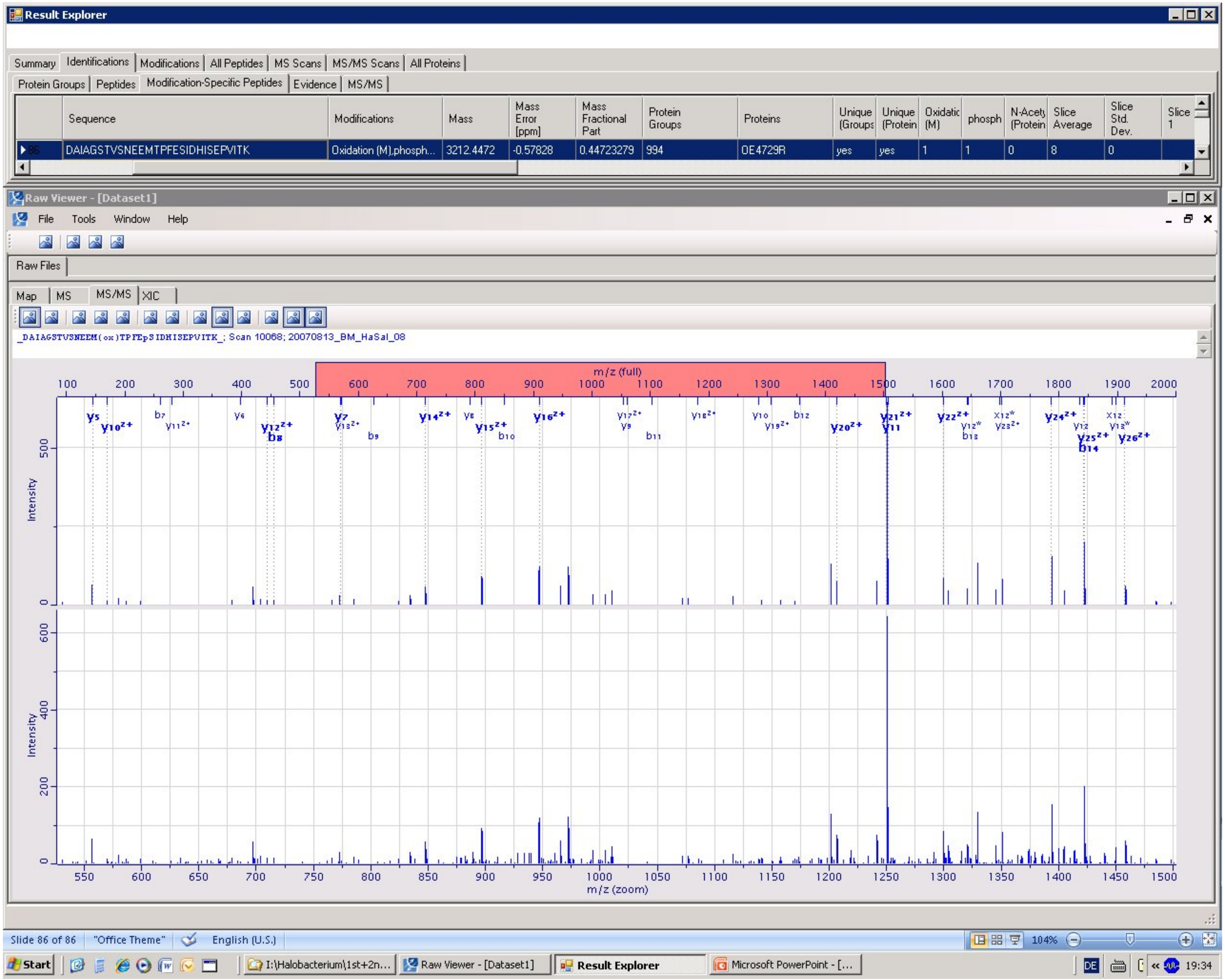
Result Explorer
Summary | Identifications | Modifications | All Peptides | MS Scans | MS/MS Scans | All Proteins
Protein Groups | Peptides | Modification-Specific Peptides | Evidence | MS/MS
Sequence | Modifications | Mass | Mass Error [ppm] | Mass Fractional Part | Protein Groups | Proteins | Unique (Groups) | Unique (Protein) | Oxidation (M) | phosph | N-Acety (Protein) | Slice Average | Slice Std. Dev. | Slice 1
DAIAGSTVSNEEMTPFESIDHISEPVITK | Oxidation (M),phosph... | 3212.4472 | -0.57828 | 0.44723279 | 994 | OE4729R | yes | yes | 1 | 1 | 0 | 8 | 0
Raw Viewer - [Dataset1]
File Tools Window Help
Raw Files
Map MS MS/MS XIC
DAIAGSTVSNEEM(ox)TPFEpSIDHISEPVITK; Scan 10068; 20070813_BM_HaSal_08
m/z (full)
Intensity
m/z (zoom)
Slide 86 of 86 "Office Theme" English (U.S.)
Start
I:\Halobacterium\1st+2n...
Raw Viewer - [Dataset1]
Result Explorer
Microsoft PowerPoint - [...
19:34

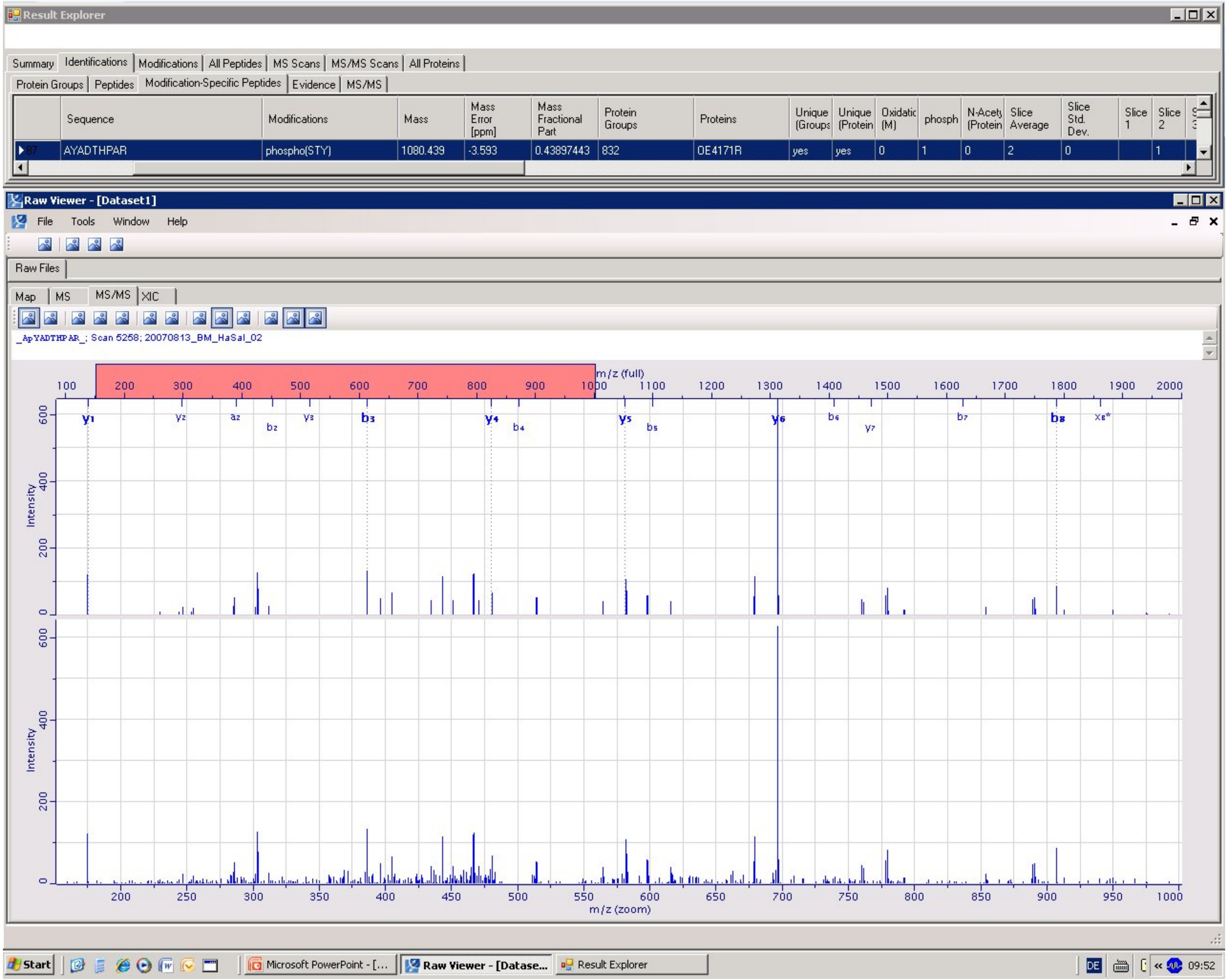
Result Explorer
Summary
Identifications
Modifications
All Peptides
MS Scans
MS/MS Scans
All Proteins
Protein Groups
Peptides
Modification-Specific Peptides
Evidence
MS/MS
Sequence
Modifications
Mass
Mass Error [ppm]
Mass Fractional Part
Protein Groups
Proteins
Unique (Groups
Unique (Protein
Oxidatic (M)
phosph
N-Acety (Protein
Slice Average
Slice Std. Dev.
Slice 1
Slice 2
AYADTHPAR
phospho(STY)
1080.439
-3.593
0.43897443
832
OE4171R
yes
yes
0
1
0
2
0
1
Raw Viewer - [Dataset1]
File
Tools
Window
Help
Raw Files
Map
MS
MS/MS
XIC
ApYADTHPAR; Scan 5258; 20070813_BM_HaSal_02
m/z (full)
y1
y2
a2
b2
y3
b3
y4
b4
y5
b5
y6
b6
y7
b7
b8
x8*
Intensity
m/z (zoom)
Start
Microsoft PowerPoint - [...
Raw Viewer - [Datase...
Result Explorer
DE
09:52

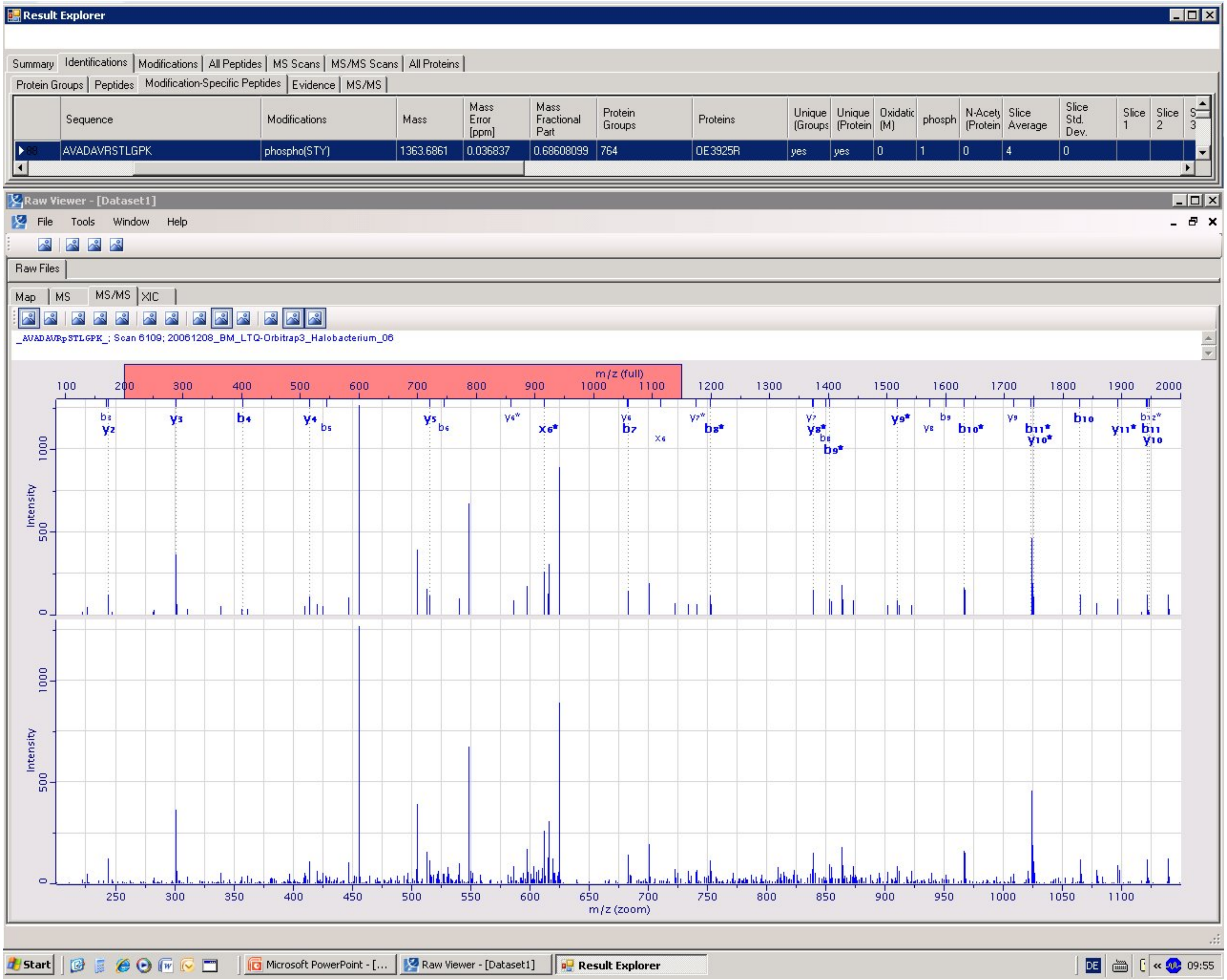
Result Explorer
Summary | Identifications | Modifications | All Peptides | MS Scans | MS/MS Scans | All Proteins
Protein Groups | Peptides | Modification-Specific Peptides | Evidence | MS/MS
Sequence | Modifications | Mass | Mass Error [ppm] | Mass Fractional Part | Protein Groups | Proteins | Unique (Groups | Unique (Protein | Oxidatic (M) | phosph | N-Acety (Protein | Slice Average | Slice Std. Dev. | Slice 1 | Slice 2
AVADAVRSTLGPK | phospho(STY) | 1363.6861 | 0.036837 | 0.68608099 | 764 | OE3925R | yes | yes | 0 | 1 | 0 | 4 | 0
Raw Viewer - [Dataset1]
File Tools Window Help
Raw Files
Map | MS | MS/MS | XIC
AVADAVRpSTLGPK; Scan 6109; 20061208_BM_LTQ-Orbitrap3_Halobacterium_06
m/z (full)
Intensity
m/z (zoom)
Start
Microsoft PowerPoint - [...
Raw Viewer - [Dataset1]
Result Explorer
DE
09:55

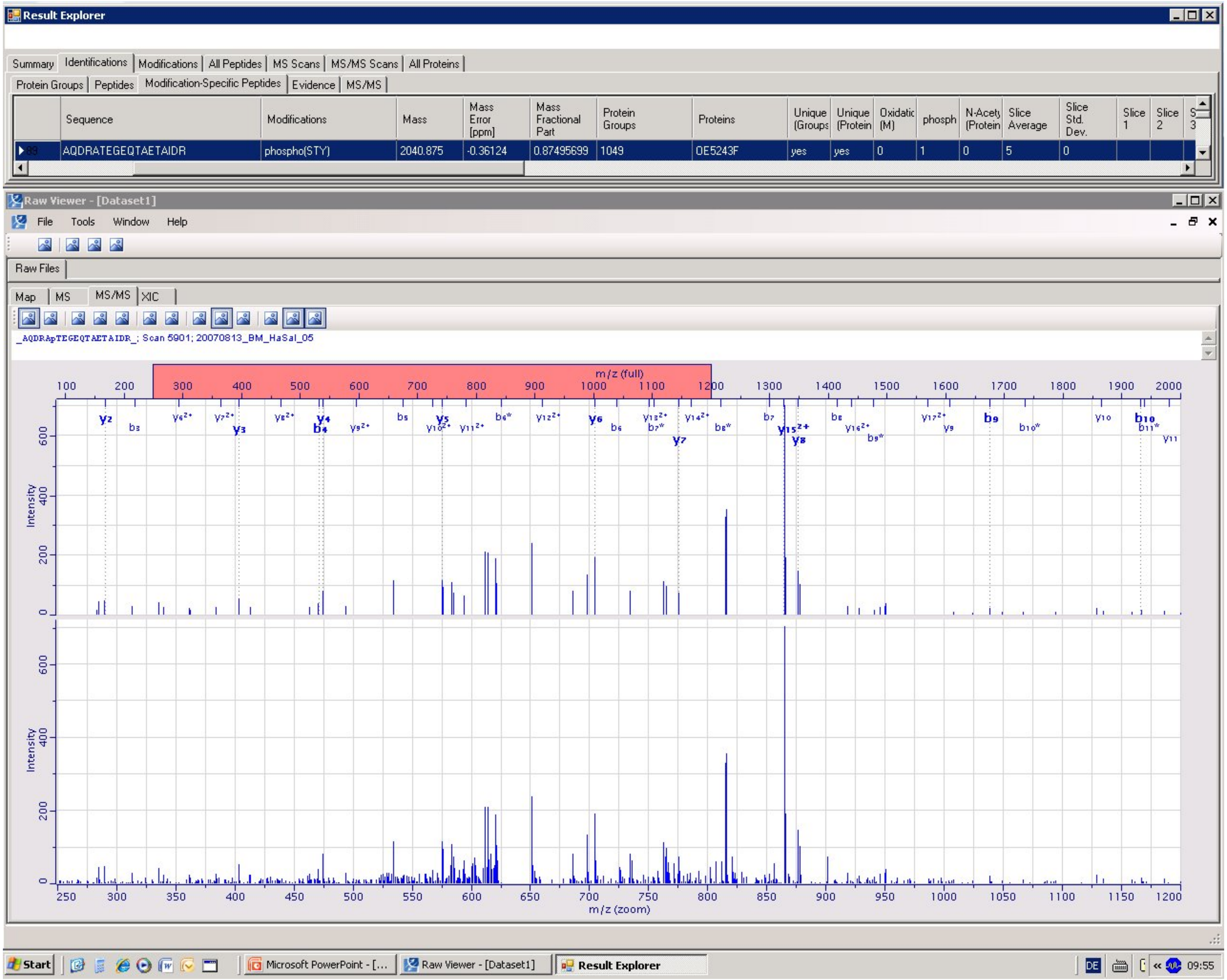
Result Explorer
Summary
Identifications
Modifications
All Peptides
MS Scans
MS/MS Scans
All Proteins
Protein Groups
Peptides
Modification-Specific Peptides
Evidence
MS/MS
Sequence
Modifications
Mass
Mass Error [ppm]
Mass Fractional Part
Protein Groups
Proteins
Unique (Groups
Unique (Protein
Oxidatic (M)
phosph
N-Acety (Protein
Slice Average
Slice Std. Dev.
Slice 1
Slice 2
AQDRATEGEQTAETAIDR
phospho(STY)
2040.875
-0.36124
0.87495699
1049
0E5243F
yes
yes
0
1
0
5
0
Raw Viewer - [Dataset1]
File
Tools
Window
Help
Raw Files
Map
MS
MS/MS
XIC
AQDRApTEGEQTAETAIDR; Scan 5901; 20070813_BM_HaSal_05
m/z (full)
Intensity
m/z (zoom)
Start
Microsoft PowerPoint - [...
Raw Viewer - [Dataset1]
Result Explorer
DE
09:55

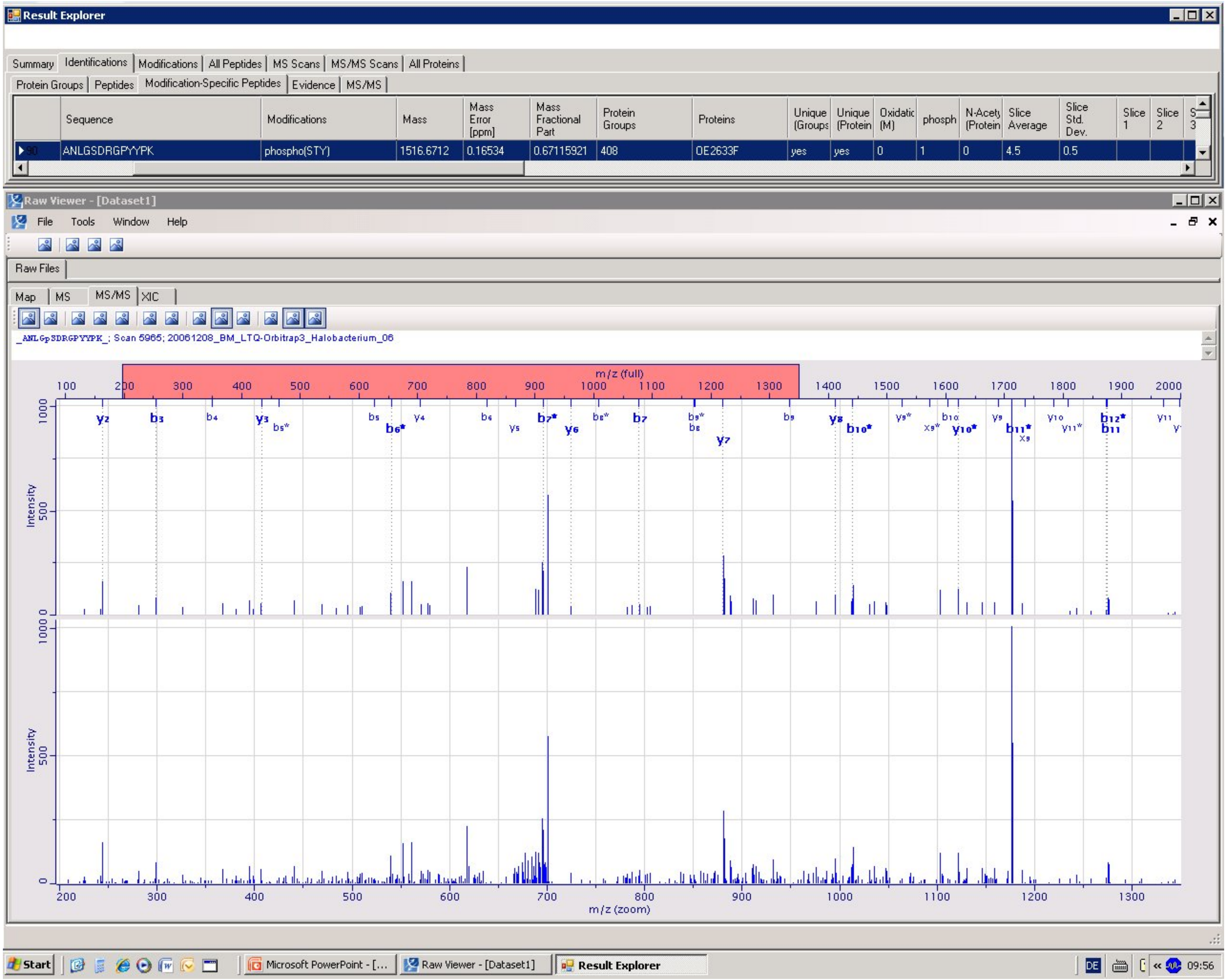
Result Explorer
Summary | Identifications | Modifications | All Peptides | MS Scans | MS/MS Scans | All Proteins
Protein Groups | Peptides | Modification-Specific Peptides | Evidence | MS/MS
Sequence | Modifications | Mass | Mass Error [ppm] | Mass Fractional Part | Protein Groups | Proteins | Unique (Groups | Unique (Protein | Oxidatic (M) | phosph | N-Acety (Protein | Slice Average | Slice Std. Dev. | Slice 1 | Slice 2
ANLGSDRGPYYPK | phospho(STY) | 1516.6712 | 0.16534 | 0.67115921 | 408 | OE2633F | yes | yes | 0 | 1 | 0 | 4.5 | 0.5
Raw Viewer - [Dataset1]
File Tools Window Help
Raw Files
Map | MS | MS/MS | XIC
ANLGpSDRGPYYPK; Scan 5965; 20061208_BM_LTQ-Orbitrap3_Halobacterium_06
m/z (full)
Intensity
m/z (zoom)
Start
Microsoft PowerPoint - [...
Raw Viewer - [Dataset1]
Result Explorer
09:56

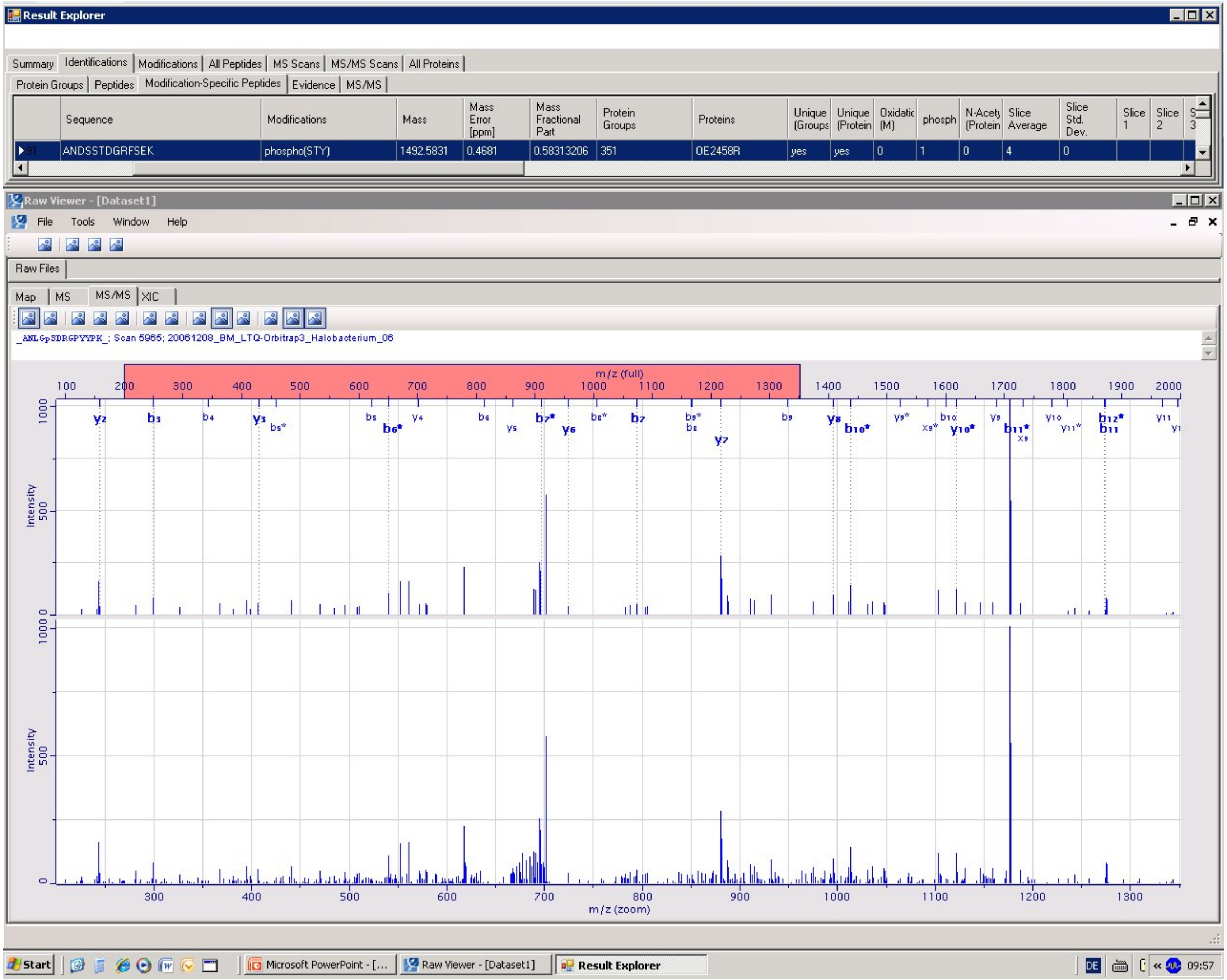
Result Explorer
Summary
Identifications
Modifications
All Peptides
MS Scans
MS/MS Scans
All Proteins
Protein Groups
Peptides
Modification-Specific Peptides
Evidence
MS/MS
Sequence
Modifications
Mass
Mass Error [ppm]
Mass Fractional Part
Protein Groups
Proteins
Unique (Groups
Unique (Protein
Oxidatic (M)
phosph
N-Acety (Protein
Slice Average
Slice Std. Dev.
Slice 1
Slice 2
ANDSSTDGRFSEK
phospho(STY)
1492.5831
0.4681
0.58313206
351
OE2458R
yes
yes
0
1
0
4
0
Raw Viewer - [Dataset1]
File
Tools
Window
Help
Raw Files
Map
MS
MS/MS
XIC
ANLGpSDRGPYYPK; Scan 5965; 20061208_BM_LTQ-Orbitrap3_Halobacterium_06
m/z (full)
Intensity
m/z (zoom)
Start
Microsoft PowerPoint - [...
Raw Viewer - [Dataset1]
Result Explorer
DE
09:57

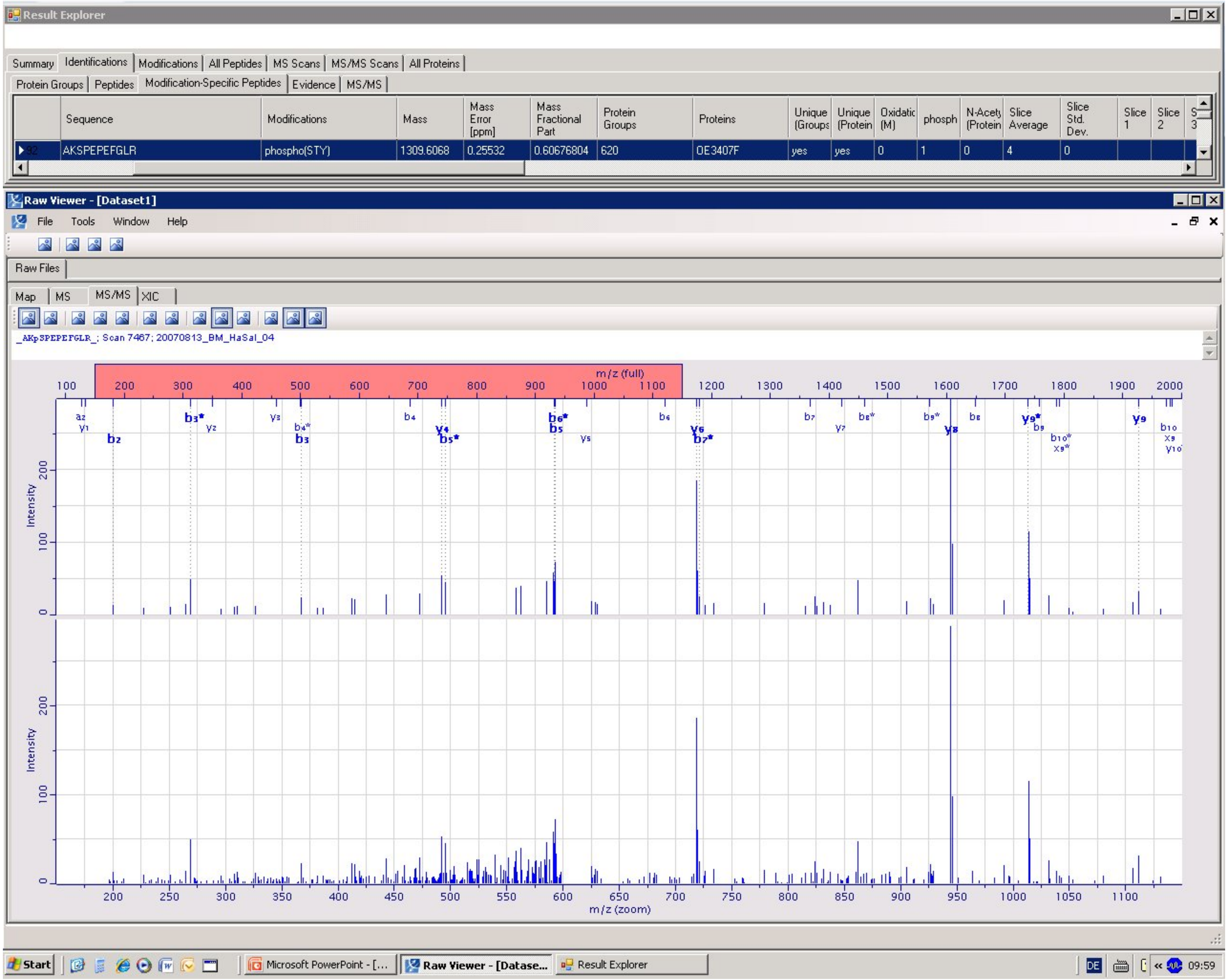
Result Explorer
Summary
Identifications
Modifications
All Peptides
MS Scans
MS/MS Scans
All Proteins
Protein Groups
Peptides
Modification-Specific Peptides
Evidence
MS/MS
Sequence
Modifications
Mass
Mass Error [ppm]
Mass Fractional Part
Protein Groups
Proteins
AKSPEPEFGLR
phospho(STY)
1309.6068
0.25532
0.60676804
620
0E3407F
Raw Viewer - [Dataset1]
File
Tools
Window
Help
Raw Files
Map
MS
MS/MS
XIC
AKpSPEPEFGLR; Scan 7467; 20070813_BM_HaSal_04
m/z (full)
Intensity
m/z (zoom)
Start
Microsoft PowerPoint - [...
Raw Viewer - [Datase...
Result Explorer
09:59

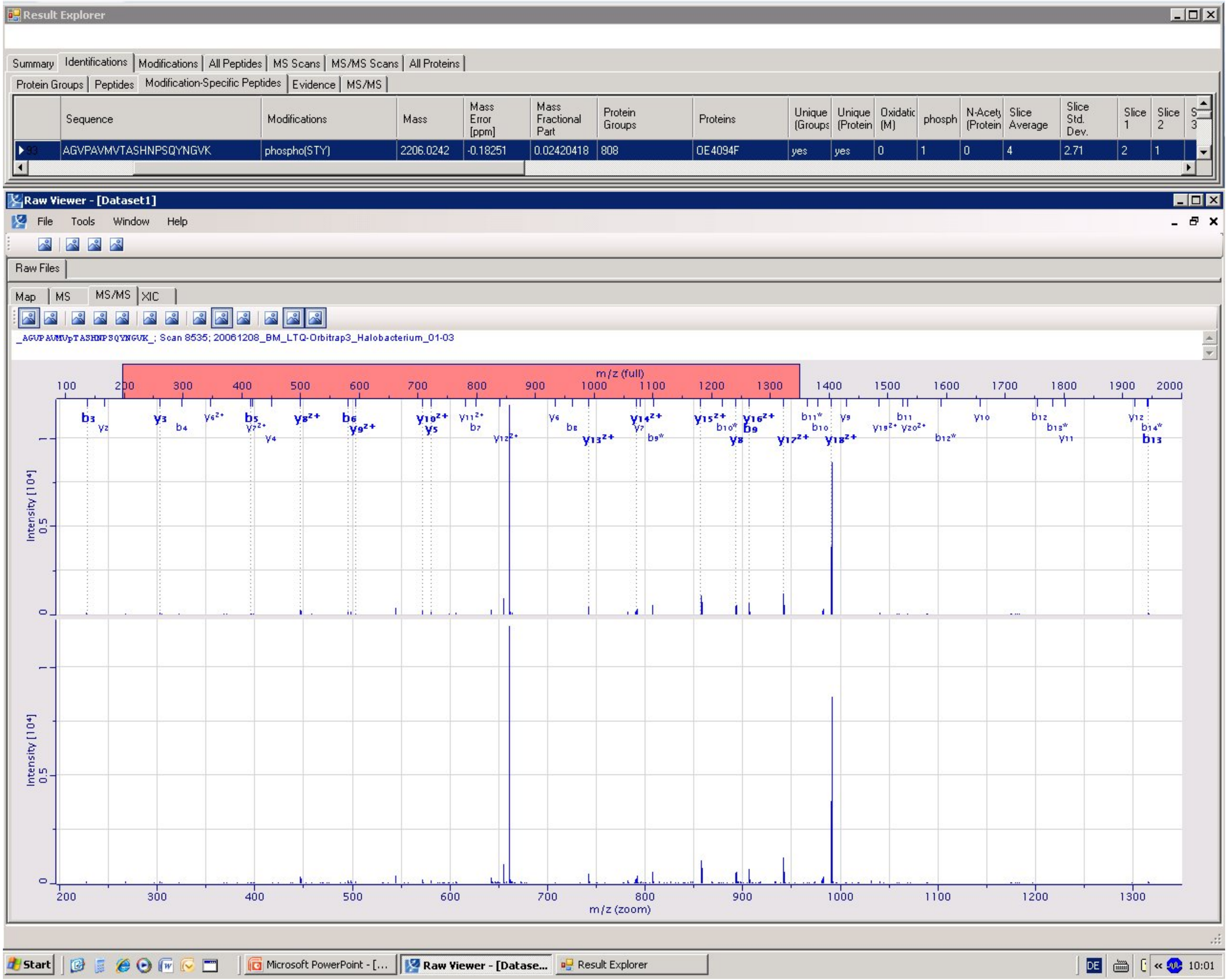
Result Explorer
Summary
Identifications
Modifications
All Peptides
MS Scans
MS/MS Scans
All Proteins
Protein Groups
Peptides
Modification-Specific Peptides
Evidence
MS/MS
Sequence
Modifications
Mass
Mass Error [ppm]
Mass Fractional Part
Protein Groups
Proteins
AGVPAVMVTASHNPSQYNGVK
phospho(STY)
2206.0242
-0.18251
0.02420418
808
OE4094F
Raw Viewer - [Dataset1]
File
Tools
Window
Help
Raw Files
Map
MS
MS/MS
XIC
m/z (full)
Intensity [10⁴]
m/z (zoom)
Start
Microsoft PowerPoint - [...
Raw Viewer - [Datase...
Result Explorer
10:01

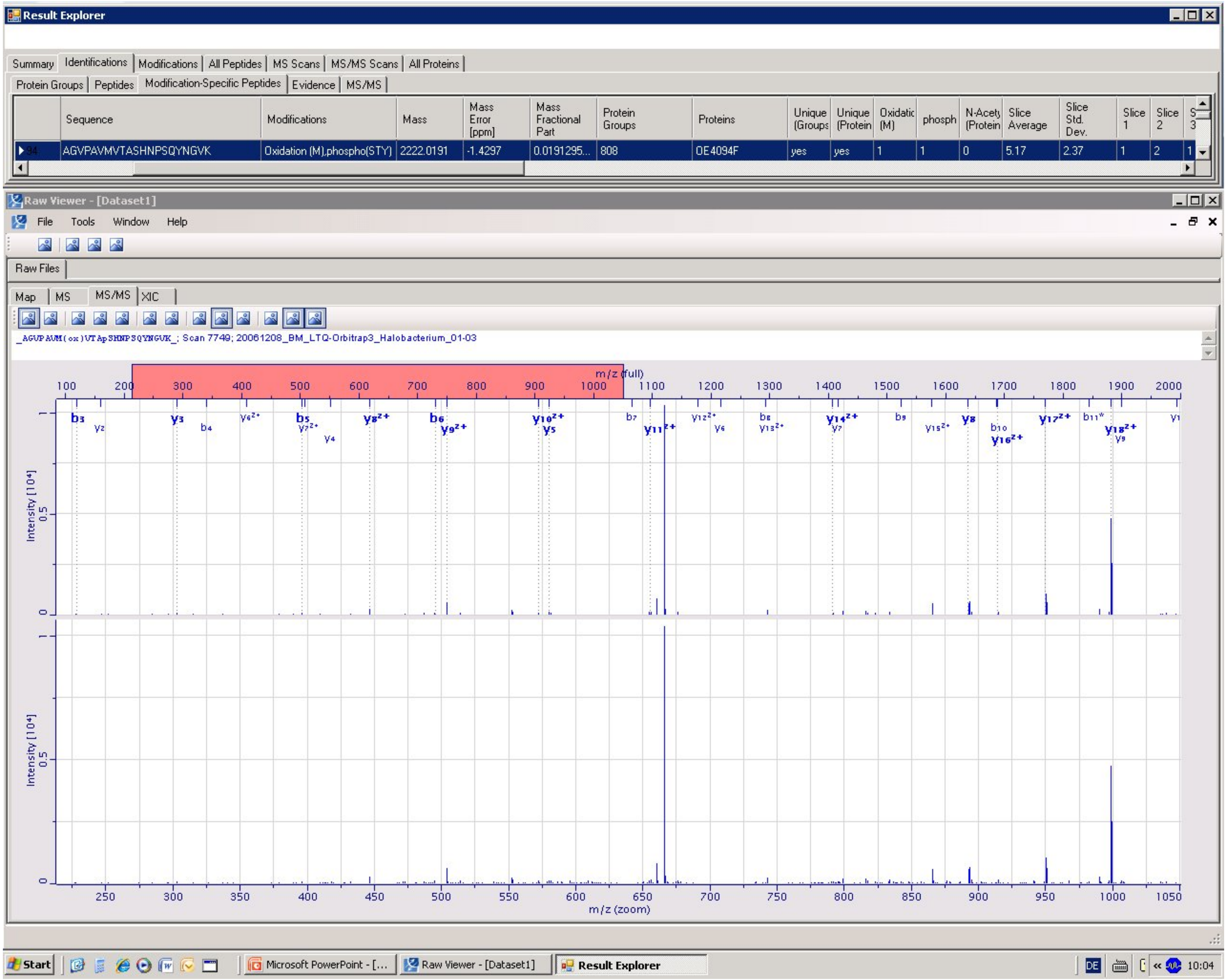
Result Explorer
Summary
Identifications
Modifications
All Peptides
MS Scans
MS/MS Scans
All Proteins
Protein Groups
Peptides
Modification-Specific Peptides
Evidence
MS/MS
Sequence
Modifications
Mass
Mass Error [ppm]
Mass Fractional Part
Protein Groups
Proteins
Unique (Groups
Unique (Protein
Oxidatic (M)
phosph
N-Acety (Protein
Slice Average
Slice Std. Dev.
Slice 1
Slice 2
AGVPAVMVTASHNPSQYNGVK
Oxidation (M),phospho(STY)
2222.0191
-1.4297
0.0191295...
808
OE4094F
yes
yes
1
1
0
5.17
2.37
1
2
Raw Viewer - [Dataset1]
File
Tools
Window
Help
Raw Files
Map
MS
MS/MS
XIC
AGVPAVM(ox)VTApSHNPSQYNGVK; Scan 7749; 20061208_BM_LTQ-Orbitrap3_Halobacterium_01-03
m/z (full)
Intensity [10⁴]
m/z (zoom)
Start
Microsoft PowerPoint - [...
Raw Viewer - [Dataset1]
Result Explorer
10:04

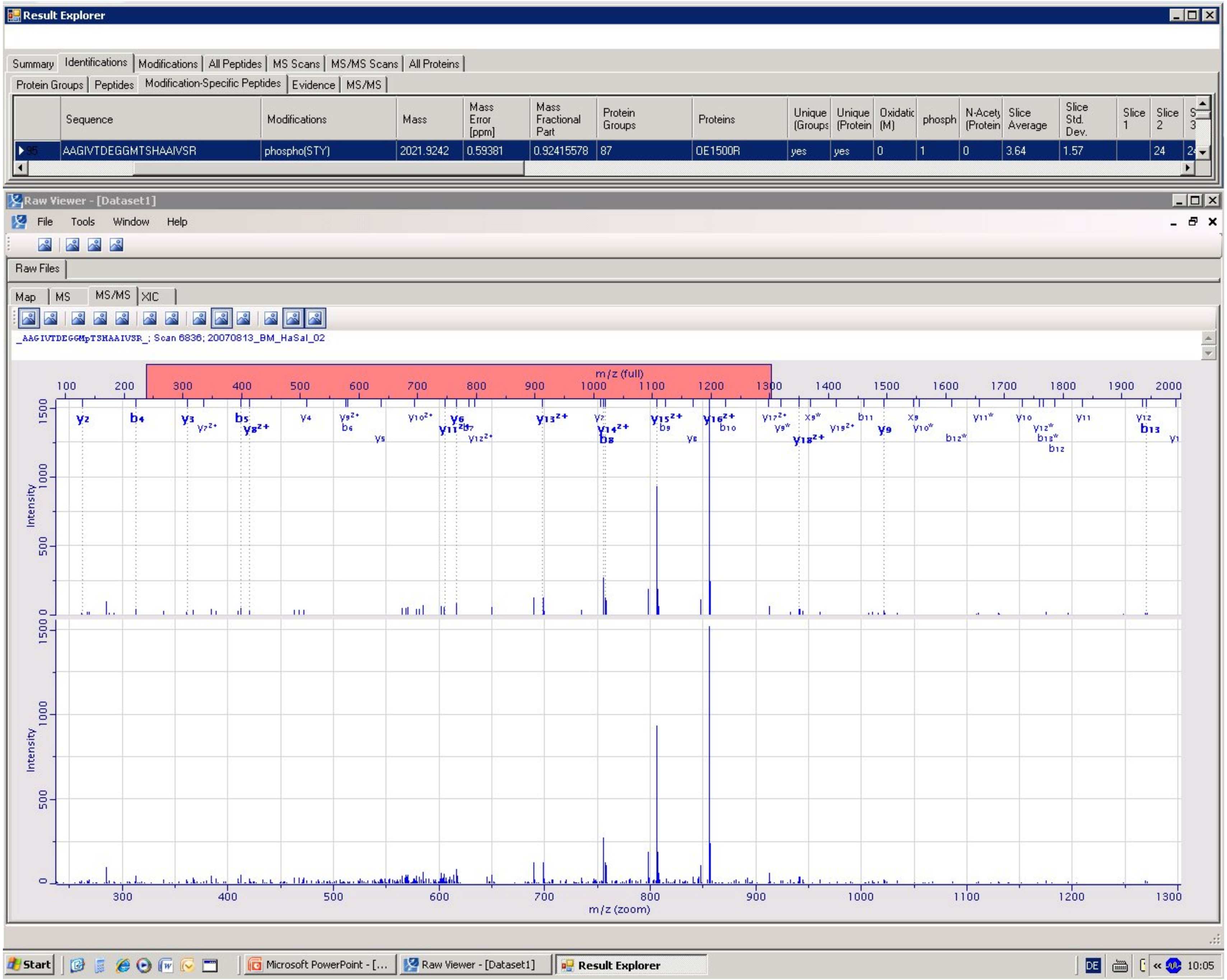
Result Explorer
Summary
Identifications
Modifications
All Peptides
MS Scans
MS/MS Scans
All Proteins
Protein Groups
Peptides
Modification-Specific Peptides
Evidence
MS/MS
Sequence
Modifications
Mass
Mass Error [ppm]
Mass Fractional Part
Protein Groups
Proteins
Slice Average
Slice Std. Dev.
AAGIVTDEGGMTSHAAIVSR
phospho(STY)
2021.9242
0.59381
0.92415578
87
OE1500R
3.64
1.57
Raw Viewer - [Dataset1]
File
Tools
Window
Help
Raw Files
Map
MS
MS/MS
XIC
AAGIVTDEGGMpTSHAAIVSR; Scan 6836; 20070813_BM_HaSal_02
m/z (full)
Intensity
m/z (zoom)
Start
Microsoft PowerPoint - [...
Raw Viewer - [Dataset1]
Result Explorer
10:05

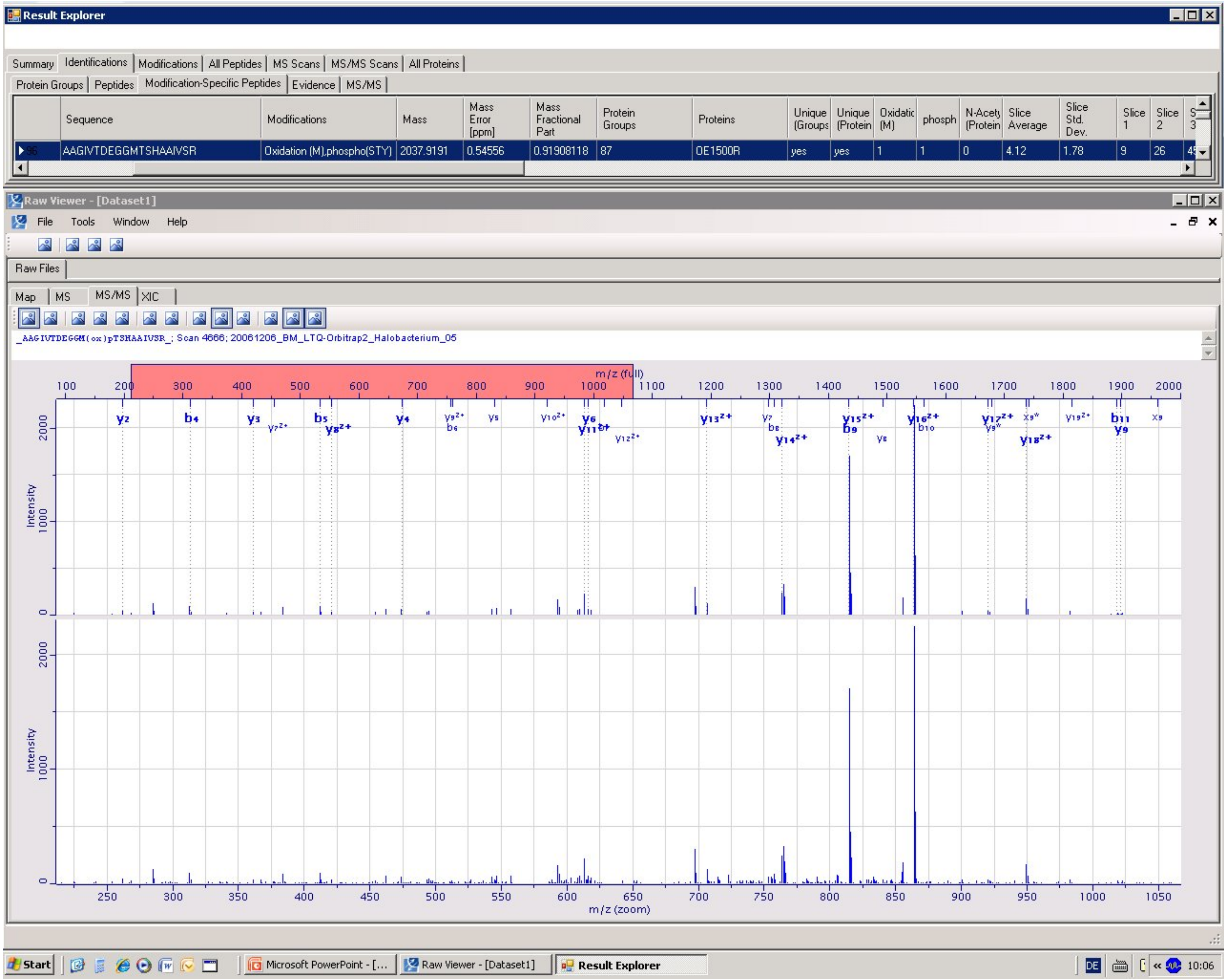
Result Explorer
Summary | Identifications | Modifications | All Peptides | MS Scans | MS/MS Scans | All Proteins
Protein Groups | Peptides | Modification-Specific Peptides | Evidence | MS/MS
Sequence | Modifications | Mass | Mass Error [ppm] | Mass Fractional Part | Protein Groups | Proteins | Unique (Groups) | Unique (Protein) | phosph | Slice Average | Slice Std. Dev. | Slice 1 | Slice 2
AAGIVTDEGGMTSHAAIVSR | Oxidation (M),phospho(STY) | 2037.9191 | 0.54556 | 0.91908118 | 87 | OE1500R | yes | yes | 1 | 1 | 0 | 4.12 | 1.78 | 9 | 26
Raw Viewer - [Dataset1]
File Tools Window Help
Raw Files
Map | MS | MS/MS | XIC
AAGIVTDEGGM(ox)pTSHAAIVSR; Scan 4666; 20061206_BM_LTQ-Orbitrap2_Halobacterium_05
m/z (full)
Intensity
m/z (zoom)
Start
Microsoft PowerPoint - [...
Raw Viewer - [Dataset1]
Result Explorer
10:06
